# Deciphering phylogenetic relationships and delimiting species boundaries using a Bayesian coalescent approach in protists: A case study of the ciliate genus *Spirostomum* (Ciliophora, Heterotrichea)

**DOI:** 10.1038/s41598-019-52722-4

**Published:** 2019-11-08

**Authors:** Shahed Uddin Ahmed Shazib, Peter Vďačný, Marek Slovák, Eleni Gentekaki, Mann Kyoon Shin

**Affiliations:** 1 Department of Biological Science, University of Ulsan, Ulsan, 44610 South Korea; 2 Department of Zoology, Comenius University in Bratislava, 842 15 Bratislava, Slovakia; 3 Plant Science and Biodiversity Centre, Institute of Botany, Slovak Academy of Sciences, 845 23 Bratislava, Slovakia; 4 Department of Botany, Charles University, 128 01 Prague, Czech Republic; 5 School of Science, Mae Fah Luang University, Chiang Rai, 57100 Thailand

**Keywords:** Biodiversity, Phylogenetics, Taxonomy, Microbiology

## Abstract

The ciliate genus *Spirostomum* comprises eight morphospecies, inhabiting diverse aquatic environments worldwide, where they can be used as water quality indicators. Although *Spirostomum* species are relatively easily identified using morphological methods, the previous nuclear rDNA-based phylogenies indicated several conflicts in morphospecies delineation. Moreover, the single locus phylogenies and previous analytical approaches could not unambiguously resolve phylogenetic relationships among *Spirostomum* morphospecies. Here, we attempt to investigate species boundaries and evolutionary history of *Spirostomum* taxa, using 166 new sequences from multiple populations employing one mitochondrial locus (CO1 gene) and two nuclear loci (rRNA operon and alpha-tubulin gene). In accordance with previous studies, relationships among the eight *Spirostomum* morphospecies were poorly supported statistically in individual gene trees. To overcome this problem, we utilised for the first time in ciliates the Bayesian coalescent approach, which accounts for ancestral polymorphisms, incomplete lineage sorting, and recombination. This strategy enabled us to robustly resolve deep relationships between *Spirostomum* species and to support the hypothesis that taxa with compact macronucleus and taxa with moniliform macronucleus each form a distinct lineage. Bayesian coalescent-based delimitation analyses strongly statistically supported the traditional morphospecies concept but also indicated that there are two *S. minus*-like cryptic species and *S. teres* is non-monophyletic. *Spirostomum teres* was very likely defined by a set of ancestral features of lineages that also gave rise to *S. yagiui* and *S. dharwarensis*. However, molecular data from type populations of the morphospecies *S. minus* and *S. teres* are required to unambiguously resolve the taxonomic problems.

## Introduction

Ciliates are common inhabitants of various environments worldwide. They are considered to be the top consumers of prokaryotic microbes in aquatic biofilms and they also play essential roles in different aspects of biological applications^1,2^. Since the discovery of the first ciliate *Paramecium* species by John Hill in 1752, more than 8,000 ciliate species have been described^3^. With the aid of advancements in molecular biology, the evolutionary history and relationships among ciliates have been studied at levels ranging from genera to classes. However, comparatively few studies have focused on integrated systematics at the species level and among closely related species^4–17^.

The heterotrichean ciliate genus *Spirostomum* Ehrenberg, 1834 represents an appropriate model for studying ciliate evolution at the subgeneric level, due to the relatively easy morphological identification of its species. *Spirostomum* includes vermiform, large ciliates (150–4000 µm long) characterised by a long collecting canal of the contractile vacuole, extending from the posterior to the anterior body end along the dorsal cell side. The ciliary rows of *Spirostomum* become spiral during cell contraction. Another characteristic diagnostic trait of this genus includes a continuous paroral membrane distinctly thickened at its proximal end after silver staining^3,6,12,18–22^. The morphological taxonomy of *Spirostomum* is mostly based on body shape and size, macronuclear pattern (i.e., shape, size, and number of macronuclear nodules), and the number of ciliary rows and cortical granule rows between them^6,12,23^. *Spirostomum* ciliates are relatively common in fresh and brackish water environments^5,24,25^, where they can be used as water quality indicators^23,26–29^. *Spirostomum* species are mostly found in microaerophilic or anaerobic conditions^23,30^ and according to transcriptome analyses they are able to respire under anoxic conditions^31^.

Recently, considerable research effort has focused on the morphology and molecular phylogeny of *Spirostomum* species^6,12,19,20,22,31–34^. Boscaro *et al*.^6^ markedly improved *Spirostomum* systematics using both morphological and gene sequence data, but species boundaries were not unambiguously identified at the molecular level. Shazib *et al*.^12^ used secondary structure information of the nuclear internal transcribed spacer 2 (ITS2) to delimit species boundaries, an approach commonly utilised for species identification and improvement of molecular phylogenetic reconstruction in various groups of organisms^35–43^. However, both primary and secondary sequence information merged several morphologically distinct species (i.e., *S. teres*, *S. yagiui* and *S. dharwarensis*) into a single group, and only a few relations were robustly resolved. All previous phylogenetic studies were limited to the nuclear rRNA gene sequences and suggested the presence of several cryptic species, especially, in *S. minus* and *S. teres*. Interrelationships among *Spirostomum* species were mostly left unclear.

In this study, 86 *Spirostomum* populations belonging to eight morphospecies were investigated, using three ribosomal markers and two protein-coding genes. This diverse dataset enabled us for the first time to explore the effectiveness of these molecular markers in species delimitation and to better understand evolutionary relationships among *Spirostomum* species. To complement the traditional tree building methods, the Bayesian coalescent approach and species network analysis were also utilised, which account for ancestral polymorphisms and incomplete lineage sorting. Finally, our aim was to compare the congruence of morphology and molecules in studying species boundaries.

## Results

### Sequence variation and genetic divergence analyses

In total, 166 new *Spirostomum* gene sequences were obtained during the course of this study, including 32 new 18S rRNA gene sequences, 33 new ITS1-5.8S-ITS2 region sequences, 33 new D1D2 region sequences of the 28S rRNA gene, 31 new alpha-tubulin gene sequences, and 37 new mitochondrial cytochrome oxidase subunit 1 (CO1) gene sequences. In addition, one new alpha-tubulin and CO1 gene sequence from *Anigstenia* sp. were also obtained. All sequences were deposited in the NCBI GenBank database (https://www.ncbi.nlm.nih.gov/nucleotide/). The corresponding GenBank accession numbers, length and GC content were summarised in Supplementary Table S1.

The intra- and inter-specific genetic distances are collated for all alignments in Supplementary Tables S2–20. To summarise, the 18S rRNA gene was the slowest evolving marker, while the mitochondrial CO1 gene was the fastest. The insert size of the CO1 barcode region was 285–288 nucleotides and 95–96 amino acids long. In the CO1 gene sequence alignment, one additional codon was commonly found in populations of *S. caudatum*, *S. minus*, *S. teres* and *S. yagiui* (SKS787), and was located in the insert region (Supplementary Fig. S1). The alpha-tubulin and CO1 gene sequences had greater mean genetic distances than the three rRNA gene sequences. *S. ambiguum* had low nucleotide variability in the rRNA locus and the alpha-tubulin gene, but markedly high diversity in the insert region of the CO1 gene. A similar pattern was also observed in *S. subtilis* and *S. yagiui*, but diversity in the CO1 gene was not so pronounced. The intra-specific genetic diversity in the morphospecies *S. minus* was comparatively higher in the ITS region than in the two other rRNA loci, but the mean intra-specific divergence was significantly lower in both protein coding genes, possibly due to the limited population sampling. The morphospecies *S. teres* showed markedly high mean intra-specific nucleotide diversities both in the rRNA locus and the CO1 gene.

### Gene trees

**Figure 1:**
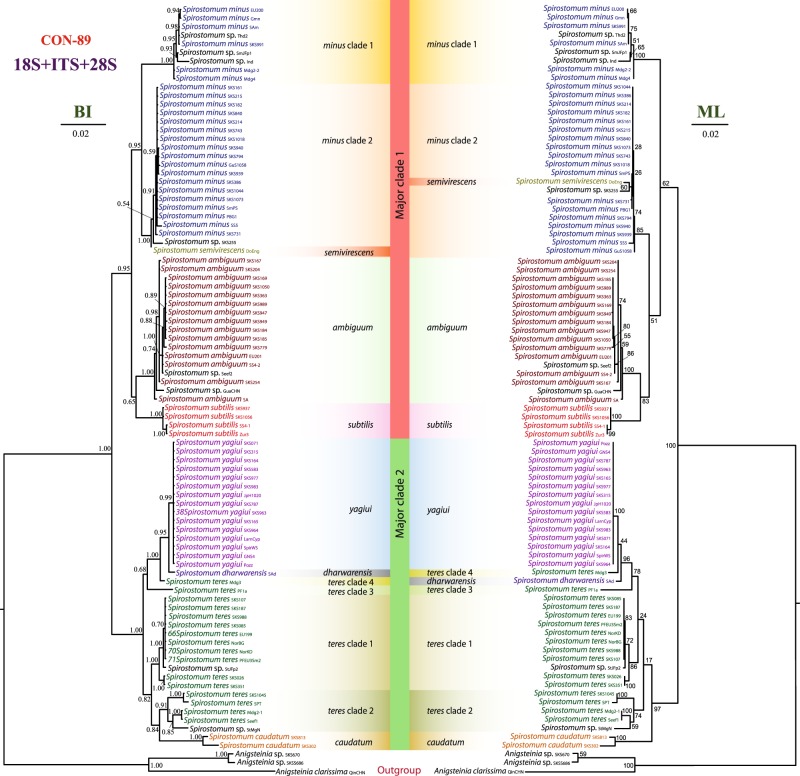
Phylogenetic relationships among 86 *Spirostomum* populations inferred from the 18S rRNA-ITS-28S rRNA concatenated dataset (CON-89). The Bayesian Inference (BI) tree is on the left and the Maximum Likelihood (ML) tree is on the right. Scale bars correspond to the number of nucleotide substitutions.

**Figure 2:**
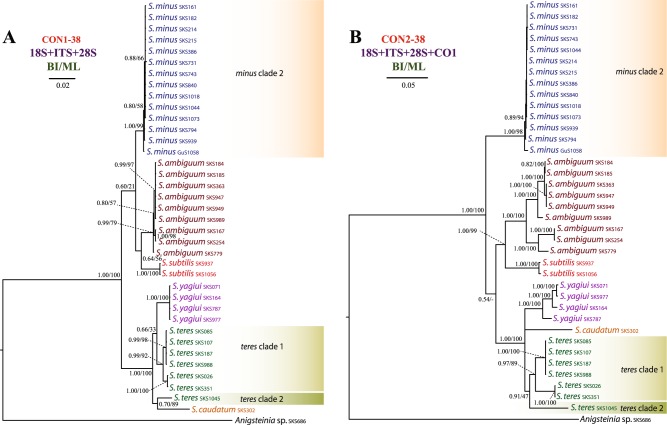
Phylogenetic relationships among 37 *Spirostomum* populations inferred from the CON1-38 and CON2-38 datasets. Results from the maximum likelihood (ML) bootstrap analyses were mapped onto the Bayesian Inference (BI) tree. Scale bars correspond to the number of nucleotide substitutions.

**Figure 3:**
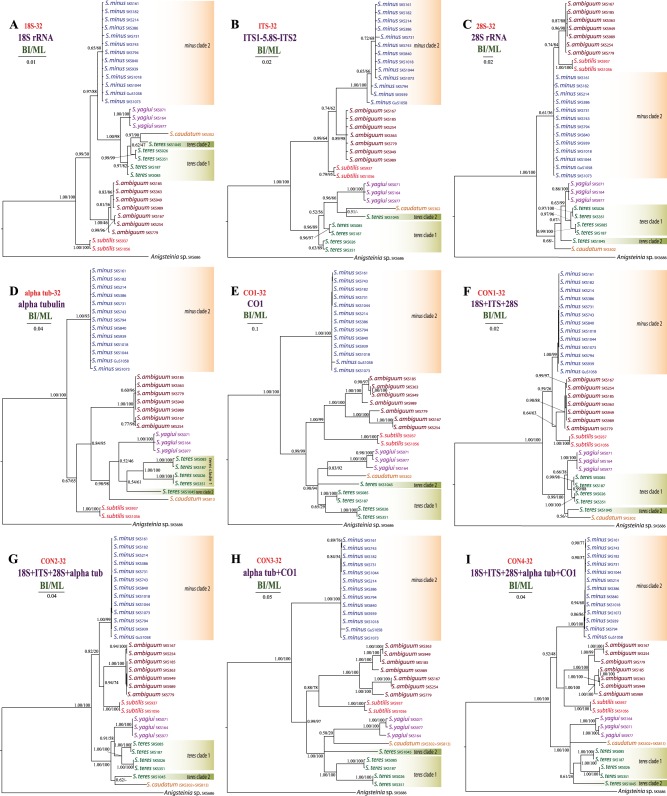
Phylogenetic relationships among 31 *Spirostomum* populations inferred from nine different datasets. Results from the maximum likelihood (ML) bootstrap analyses were mapped onto the Bayesian Inference (BI) tree. Scale bars correspond to the number of nucleotide substitutions.

We used different datasets to investigate the effect of taxa sampling and masking on phylogenetic inferences. In almost all resultant phylogenetic trees (Figs 1–3), *Spirostomum* species were divided into two major clades. Clade 1 included *S. ambiguum*, *S. minus*, *S. semivirescens*, and *S. subtilis*, while clade 2 was comprised of *S. caudatum*, *S. dharwarensis*, *S. teres*, and *S. yagiui*. However, *S. semivirescens* and *S. dharwarensis* were present only in the 89 taxa dataset (Fig. 1). Single gene trees were, in general, congruent with concatenated trees, especially with regards to the well-supported monophyletic lineages. Although the branching pattern of phylogenetic trees inferred from the ribosomal locus (18S rRNA, ITS, 28S rRNA) and from the protein-coding gene sequences (alpha tubulin, CO1) was inconsistent, the conflicting topologies were not statistically supported. The most significant disagreement was the placement of the *S. ambiguum* + *S. subtilis* clade. However, its discordant phylogenetic position was also not statistically supported.

*Spirostomum minus*, *S. semivirescens*, *S. ambiguum* and *S. subtilis* formed a monophyletic group with variable support in the majority of trees (Figs 1–3). Their interrelationships were, however, inconsistent across analyses. *Spirostomum ambiguum* and *S. subtilis* were grouped in a sister position to *S. minus* in the concatenated trees, i.e. CON1-38 BI, ML trees (Fig. 2A), CON2-38 ML tree (Fig. 2B), CON1-32 BI, ML trees (Fig. 3F), CON2-32 BI, ML trees (Fig. 3G), and CON4-32 BI, ML trees (Fig. 3I), while they were separated from *S. minus* and grouped with major clade 2 in the protein-coding gene trees (Fig. 3D,E,H). Nonetheless, this conflicting relationship received no statistical support. *Spirostomum minus* was not monophyletic and its populations formed two well supported clades in trees inferred from the 89 taxa dataset, whereby *S. semivirescens* was integrated into a clade along with *S. minus* clade 2 and *Spirostomum* sp. SKS255 in both BI and ML trees (Fig. 1). However, *S. minus* was recovered as a monophyletic lineage in trees based on the 32 and 38 taxa datasets (Figs 2 and 3), very likely due to the limited population sampling. *Spirostomum ambiguum* and *S. subtilis* were consistently grouped together in all concatenated analyses with low to strong support (Figs 1–3).

*Spirostomum teres*, *S. yagiui*, *S. caudatum*, and *S. dharwarensis* formed a strongly statistically supported clade in most concatenated analyses. However, their interrelationships varied depending on the molecular markers and phylogenetic technique used. This instability was also reflected in the weak statistical support for the majority of the clusters. The CON-89 dataset indicated that *S. caudatum* might have branched off first in major clade 2 (ML tree) but bootstrap support for this position was very weak (17%). In the BI tree, it was depicted as a sister taxon of the *S. teres* clade 2 (Fig. 1). Its position was also inconsistent in trees inferred from other datasets (Figs 2 and 3). *Spirostomum teres* remained non-monophyletic both in the single gene and the concatenated analyses based on four or more molecular markers (CON2-32 in Fig. 3G) but was recovered as a monophyletic lineage in the datasets CON2-38 and CON4-32 (Figs 2B and 3I) and the alpha-tubulin gene tree (Fig. 3D) with insignificant statistical support. This may be due to the limited population sampling.

All statistical tree topology tests did not reject the monophyletic origin of *Spirostomum* species having a moniliform macronucleus (i.e., clade 1) and monophyly of *Spirostomum* species with a compact macronucleus (i.e., clade 2). Regarding the CON-89 alignment, all statistical tests refuted monophyly of the four *S. teres* clades, but did not exclude monophyly of *S. teres* clades 1, 2, and 3. *Spirostomum teres* might be monophyletic when the single *S. teres* clade 4 population (Mdg3) is excluded. The monophyly of the two *S. minus* clades was firmly rejected by the AU test (*p* = 0.036) conducted on the concatenated 89 taxa alignment (Supplementary Table S21).

### Species trees and multispecies coalescent analyses

**Figure 4:**
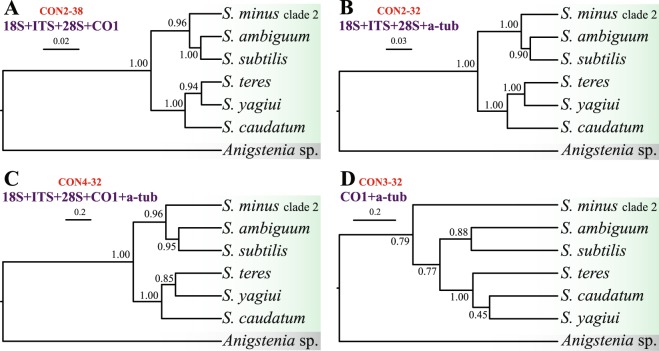
Phylogenetic relationships among six *Spirostomum* species inferred from four different datasets. Species trees were estimated using the Bayesian multispecies coalescent method. Values at nodes represent posterior probabilities. Scale bar corresponds to the number of nucleotide substitutions.

**Figure 5:**
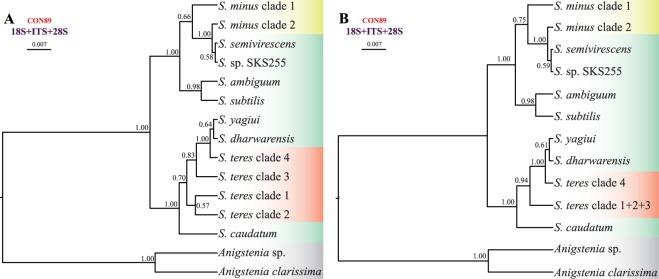
Phylogenetic relationships among *Spirostomum* clades inferred from the CON-89 dataset. Species trees were estimated using the Bayesian multispecies coalescent method. Two models were considered, with 15 (**A**) and 13 (**B**) separate lineages. Values at nodes represent posterior probabilities. Scale bar corresponds to the number of nucleotide substitutions.

Species trees were built from five datasets, as specified in Figs 4 and 5. All trees, except those inferred from the two protein-coding genes (Fig. 4D), consistently recognised two main clades within the genus *Spirostomum*. These clades matched those depicted in the majority of gene trees but they received high or full statistical support in multispecies coalescent analyses (cp. Figs 1–5). The branching pattern within the first main clade was robustly resolved in four and five marker trees: *S. minus* clade 2 branched off first and *S. ambiguum* and *S. subtilis* were sister taxa (Fig. 4). In species trees based on three markers, the two *S. minus* lineages did not group together but the *S. minus* clade 2 was placed in a sister position to the *S. semivirescens* + *Spirostomum* sp. SKS255 clade (Fig. 5), as also indicated in gene trees (Fig. 1). The grouping of *S. semivirescens* + *Spirostomum* sp. SKS255 was, however, left statistically unsupported in species trees. As concerns the branching pattern within the second main *Spirostomum* clade, results depended on the dataset analysed. Nevertheless, coalescent trees indicated that *S. caudatum* might have branched off first and *S. teres*, *S. yagiui*, and *S. dharwarensis* might cluster together. Relationships between *S. teres* clades 1–3 were left unresolved and, therefore, it cannot be excluded that they might belong to the same species (Fig. 5A). *Spirostomum teres* clade 4 was consistently classified with full statistical support in a clade along with *S. yagiui* and *S. dharwarensis* (Fig. 5A,B). This indicates that the *S. teres* clade 4 population (Mdg3) might represent a distinct species.

Altogether, six Bayesian species delimitation analyses were conducted, as specified in Table 1. Analyses based on the datasets CON2-38, CON2-32, and CON4-32 were fully consistent and recognised all lineages depicted in coalescent trees as distinct species with a posterior probability of 1.00. Thus, multiple molecular markers statistically corroborated very well the morphospecies concept of the genus *Spirostomum* and supported the validity of *S. ambiguum*, *S. subtilis*, *S. minus* (clade 2), *S. caudatum*, *S. teres* (clades 1 + 2), and *S. yagiui*. A similar result was obtained from the dataset CON3-32. The single exceptions were *S. caudatum* and *S. yagiui*, which were not statistically significantly delimited as distinct species, indicating alpha-tubulin and CO1 genes do not harbour enough phylogenetic signal to delimit *Spirostomum* species within the second main clade. With regard to the CON-89 dataset, two scenarios were considered given the results of statistical topology tests (Supplementary Table S21): altogether 15 lineages (i.e., four distinct clades were assumed in *S. teres*) were analysed in the first model and only 13 lineages (i.e., *S. teres* clades 1–3 were merged and only clade 4 was left separated) in the second model. Respective Bayesian delimitation analyses consistently recognised all assumed lineages, except for *Spirostomum* sp. SKS255 and *S. semivirescens* which were not statistically significantly delimited as distinct taxa, indicating they might be conspecific (Table 1). However, the distinctness of both *S. minus* clades was robustly supported and the morphospecies *S. teres* was depicted as non-monophyletic.

**Table 1:** Posterior probabilities for the number of delimited lineages/species, using different priors for model parameters.

Dataset	Prior	Posterior probability for number of delimited lineages	Conclusion
CON2-38	*θ* ~ G(2, 1000), *τ* ~ G(2, 2000)	*P*_7_ = 1.00	Morphospecies concept statistically supported
	*θ* ~ G(2, 100), *τ* ~ G(2, 200)	*P*_7_ = 1.00	Morphospecies concept statistically supported
	*θ* ~ G(2, 100), *τ* ~ G(2, 2000)	*P*_7_ = 1.00	Morphospecies concept statistically supported
	*θ* ~ G(2, 1000), *τ* ~ G(2, 200)	*P*_7_ = 1.00	Morphospecies concept statistically supported
CON2-32	*θ* ~ G(2, 1000), *τ* ~ G(2, 2000)	*P*_7_ = 1.00	Morphospecies concept statistically supported
	*θ* ~ G(2, 100), *τ* ~ G(2, 200)	*P*_7_ = 1.00	Morphospecies concept statistically supported
	*θ* ~ G(2, 100), *τ* ~ G(2, 2000)	*P*_7_ = 1.00	Morphospecies concept statistically supported
	*θ* ~ G(2, 1000), *τ* ~ G(2, 200)	*P*_7_ = 1.00	Morphospecies concept statistically supported
CON4-32	*θ* ~ G(2, 1000), *τ* ~ G(2, 2000)	*P*_7_ = 1.00	Morphospecies concept statistically supported
	*θ* ~ G(2, 100), *τ* ~ G(2, 200)	*P*_7_ = 1.00	Morphospecies concept statistically supported
	*θ* ~ G(2, 100), *τ* ~ G(2, 2000)	*P*_7_ = 1.00	Morphospecies concept statistically supported
	*θ* ~ G(2, 1000), *τ* ~ G(2, 200)	*P*_7_ = 1.00	Morphospecies concept statistically supported
CON3-32	*θ* ~ G(2, 1000), *τ* ~ G(2, 2000)	*P*_7_ = 0.84, *P*_6_ = 0.13	*S. caudatum* and *S. yagiui* not statistically significantly delimited as distinct species
	*θ* ~ G(2, 100), *τ* ~ G(2, 200)	*P*_7_ = 0.89, *P*_6_ = 0.10	*S. caudatum* and *S. yagiui* not statistically significantly delimited as distinct species
	*θ* ~ G(2, 100), *τ* ~ G(2, 2000)	*P*_7_ = 0.78, *P*_6_ = 0.20	*S. caudatum* and *S. yagiui* not statistically significantly delimited as distinct species
	*θ* ~ G(2, 1000), *τ* ~ G(2, 200)	*P*_7_ = 0.94, *P*_6_ = 0.05	*S. caudatum* and *S. yagiui* not statistically significantly delimited as distinct species
CON-89	Assuming 15 lineages		
	*θ* ~ G(2, 1000), *τ* ~ G(2, 2000)	*P*_15_ = 0.73, *P*_14_ = 0.27	*Spirostomum* sp. SKS255 and *S. semivirescens* not statistically significantly delimited as distinct species
	*θ* ~ G(2, 100), *τ* ~ G(2, 200)	*P*_15_ = 0.44, *P*_14_ = 0.56	*Spirostomum* sp. SKS255 and *S. semivirescens* not statistically significantly delimited as distinct species
	*θ* ~ G(2, 100), *τ* ~ G(2, 2000)	*P*_15_ = 0.68, *P*_14_ = 0.32	*Spirostomum* sp. SKS255 and *S. semivirescens* not statistically significantly delimited as distinct species
	*θ* ~ G(2, 1000), *τ* ~ G(2, 200)	*P*_15_ = 0.58, *P*_14_ = 0.42	*Spirostomum* sp. SKS255 and *S. semivirescens* not statistically significantly delimited as distinct species
CON-89	Assuming 13 lineages		
	*θ* ~ G(2, 1000), *τ* ~ G(2, 2000)	*P*_13_ = 0.71, *P*_12_ = 0.29	*Spirostomum* sp. SKS255 and *S. semivirescens* not statistically significantly delimited as distinct species
	*θ* ~ G(2, 100), *τ* ~ G(2, 200)	*P*_13_ = 0.46, *P*_12_ = 0.54	*Spirostomum* sp. SKS255 and *S. semivirescens* not statistically significantly delimited as distinct species
	*θ* ~ G(2, 100), *τ* ~ G(2, 2000)	*P*_13_ = 0.63, *P*_12_ = 0.36	*Spirostomum* sp. SKS255 and *S. semivirescens* not statistically significantly delimited as distinct species
	*θ* ~ G(2, 1000), *τ* ~ G(2, 200)	*P*_13_ = 0.55, *P*_12_ = 0.45	*Spirostomum* sp. SKS255 and *S. semivirescens* not statistically significantly delimited as distinct species

For the CON-89 dataset, two scenarios were considered given the results of statistical topology tests (Supplementary Table S21): altogether 15 lineages (i.e., four distinct clades were assumed in *S. teres*) were analysed in the first model, while only 13 lineages (i.e., *S. teres* clades 1–3 were merged and only clade 4 was left separated) in the second model. For species trees and lineages, see Figs 4 and 5.

**Figure 6:**
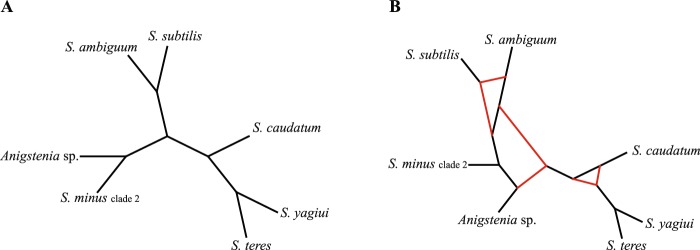
Species networks inferred from the 18S rRNA-ITS-28S rRNA, CO1 and alpha-tubulin Bayesian consensus trees based on the CON1-32, CO1-32, and alpha tub-32 datasets, respectively. Network retrieved with a maximum of zero (**A**) and ten (**B**) reticulation nodes allowed.

Species network analyses brought additional insights into conflicts between gene trees and poor statistical supports at some nodes. Networks computed with a maximum of zero reticulation nodes had topologies similar to the coalescent species trees (pseudo-likelihoods from − 3188.94 to − 3409.56), except for the position of *S. minus* (clade 2), which was depicted as the deepest branching species (Fig. 6A). The highest pseudo-likelihood (− 2296.43) was obtained in an analysis limited to 10 reticulation nodes, whereby the resulting network had six reticulation nodes (Fig. 6B). The pattern of the species network most likely reflects a very deep incomplete lineage sorting, ancestral polymorphism, and/or population substructure along the branches of the phylogeny.

### Selection tests

Branch-site Unrestricted Statistical Test for Episodic Diversification (BUSTED) was conducted to test for positive selection and to reveal the proportion of neutral, negative, and positively selected codon positions in the alpha-tubulin and CO1 genes of *Spirostomum* species. The null constrained model disallowing positive selection was not rejected either for the alpha-tubulin (likelihood ratio test LRT, *p*-value = 0.987) or the CO1 (LRT, *p*-value = 0.861) gene. Thus, both protein-coding genes very likely did not experience positive selection during the *Spirostomum* evolution.

**Figure 7:**
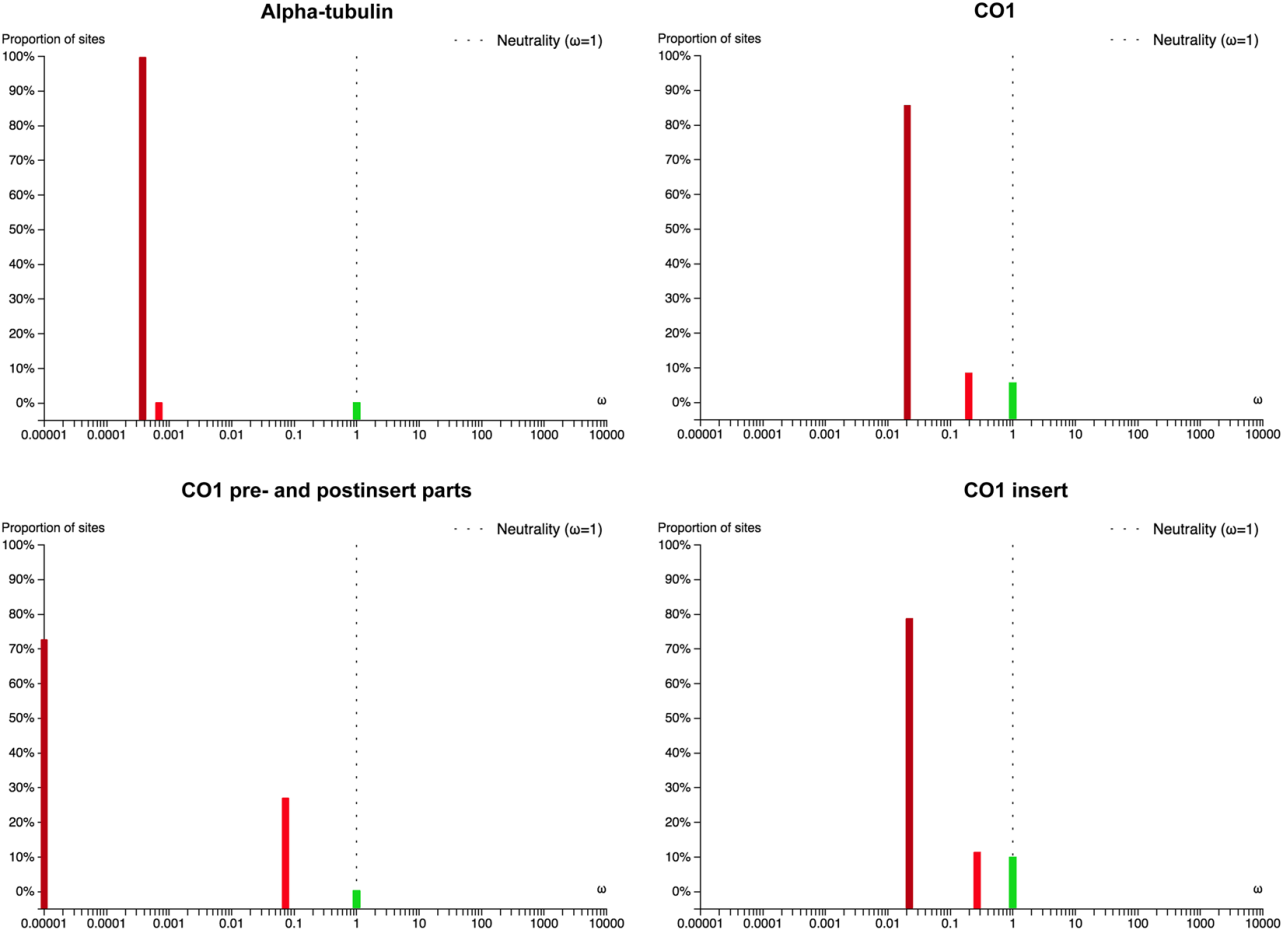
Proportion of sites belonging to three *ω* classes (*ω*_1_ ≤ *ω*_2_ ≤ *ω*_3_ and *ω*_3_ = 1) in alpha-tubulin and CO1 estimated with BUSTED.

The rate ratio of non-synonymous/synonymous changes *ω* = d_N_/d_S_ was smaller than 0.001 in 99.88% of the codon positions, documenting very strong negative selection acting on alpha-tubulin. Only 0.12% of the codon positions in alpha-tubulin evolved neutrally. In the case of CO1, the *ω* ratio was below 0.02 in 85.77% and below 0.20 in 8.57% of the codon positions. Only 5.66% of the codon positions in CO1 evolved neutrally. According to the BUSTED analyses, a higher proportion of codon positions were under strong negative selection in the pre- and post-insert parts of CO1 than in the CO1 insert part (Fig. 7). Thus, only 0.29% of the codon positions evolved neutrally in the pre- and post-insert parts of CO1, while 9.93% of the codon positions evolved neutrally in the CO1 insert part. Strong negative selection might have generated signal that is different from species ancestry in both protein-coding genes during the *Spirostomum* evolution.

## Discussion

### Utility of genetic markers in species delimitation and molecular phylogeny

In the present paper, we analysed the utility of five genetic markers in tree inferences and species delimitation: the mitochondrial cytochrome oxidase 1 (CO1) gene, the nuclear alpha-tubulin gene as well as the nuclear 18S rRNA gene, the ITS1-5.8S-ITS2 region, and the D1D2 domains of the 28S rRNA gene. These markers are commonly employed in reconstruction of phylogenetic relationships among closely related ciliate species^5–12,14–17,33,44–51^. Among the five genetic markers analysed, CO1 gene sequences showed the highest genetic divergence between morphospecies of the genus *Spirostomum* (Supplementary Table S2). However, rRNA gene sequences including the fast evolving ITS region and the D1D2 domains of the 28S rRNA gene also showed some degree of variability inside the same morphospecies. This variability was suitable for discriminating individual species, since only rRNA genes brought results consistent with coalescent trees based on three, four, and five gene datasets as well as with morphological evolutionary scenarios. This suggests that *Spirostomum* species with a moniliform macronucleus cluster together and species with a compact macronucleus form a separated sister clade^12^. In contrast, trees inferred solely from individual protein-coding genes or their combination displayed different topologies, which might reflect purifying selection rather than speciation processes. Indeed, the BUSTED analyses revealed that strong purifying negative selection might have acted on the alpha-tubulin and CO1 gene (Fig. 7). Selection pressure might have generated signal that is different from species ancestry^52,53^. The significance of protein-coding genes in discriminating species boundaries was also questioned by the present Bayesian species delimitation based only on the alpha-tubulin and CO1 genes. In the coalescent species tree inferred solely from these two protein-coding genes, *S. yagiui* and *S. caudatum* were depicted as sister taxa and the model where their ancestral node is collapsed was favoured in Bayesian delimitation analyses. This result indicated that both species might represent a single species in the light of alpha-tubulin and CO1, which is in contradiction with all three, four, and five gene delimitation analyses and also with morphological data^12^.

### Evolutionary relationships among *Spirostomum* species

Phylogenetic relationships among *Spirostomum* species using single or multiple gene trees have not been clearly resolved in previous analyses. Although most gene trees indicated a split of *Spirostomum* into two major clades, statistical support for these clades was usually poor^6,12,31,32^. On the other hand, the species trees presented herein, which were based on the coalescent model taking into account phenomena such as incomplete lineage sorting, ancestral polymorphisms and/or recombination^54^, recovered both major clades with strong statistical support. This finding supports previous morphological evolutionary scenarios, suggesting that *Spirostomum* species with a moniliform macronucleus form a distinct clade that is sister to the clade of *Spirostomum* species with a compact macronucleus^12^. Specifically, all members of major clade 1 (*S. ambiguum*, *S. minus*, *S. semivirescens*, *S. subtilis*) possess a moniliform macronucleus, while species from major clade 2 (*S. caudatum*, *S. dharwarensis*, *S. teres* and *S. yagiui*) exhibit a compact ellipsoidal or elongated curved macronucleus.

The coalescent approach also helped to resolve phylogenetic relationships better within major clade 1 and supported the existence of one morphologically cryptic species that was identified as *S. minus* in the previous studies^6,12^. *Spirostomum minus* clade 2 is closely related to *S. semivirescens* which could not be unambiguously separated from *Spirostomum* sp. SKS255 in Bayesian species delimitation analyses. On the other hand, *S. ambiguum* and *S. subtilis* are consistently depicted as sister species. The latter two species also have large bodies, being 1,000–4,000 µm long in *S. ambiguum* and 700–1,000 µm long in *S. subtilis*. *Spirostomum minus* is much smaller, typically being only 300–400 µm long,^6,19,23,34^ while *S. semivirescens* is typically 600–2000 µm long with numerous symbiotic algae in the cytoplasm^6,31,55^.

Phylogenetic relationships within the second major clade are much more intricate, which might be due to the paucity of phylogenetic signal and/or presence of non-historical signal^56^. Our coalescent network analyses suggest a very deep incomplete lineage sorting in the genus *Spirostomum* (Fig. 6). Hybridization, another source of reticulation in species networks, is highly unlikely since hybrids of even closely related ciliate species are usually not viable^57^. The present coalescent analyses indicate that *S. caudatum* branches off first, *S. teres* is non-monophyletic, and *S. yagiui* and *S. dharwarensis* are sister taxa. The two latter species share an elongated macronucleus, while the two former morphospecies have an ellipsoidal macronucleus, which was very likely an ancestral condition in the main *Spirostomum* clade 2^12^. The present phylogenetic trees support the hypothesis of Boscaro *et al*.^6^ that the morphospecies *S. teres* was very likely defined by a set of ancestral features of lineages that gave also origin to *S. yagiui* and *S. dharwarensis*.

### Taxonomic implications

Boscaro *et al*.^6^ proposed eight valid *Spirostomum* morphospecies. Gene trees have indicated that *S. minus* and *S. teres* very likely represent species complexes and/or include multiple cryptic species^6,12,19^. According to the present Bayesian delimitation analyses, *S. minus* should indeed be split into two species, which supports the previous crypticity hypothesis. The mean genetic divergences among *S. minus* populations were 0.37%, 2.57%, and 1.91% in the 18S rRNA gene, ITS region, and D1D2 domains of the 28S rRNA gene, respectively (89 taxa dataset) (Supplementary Table S2). Very similar genetic distances have also been inferred for the ITS region by Shazib *et al*.^12^. The mitochondrial CO1 sequences suggested a mean nucleotide diversity of 0.44% (from 14 clones), which is lower than the mean nucleotide diversity in the ITS region from the 89 taxa dataset. However, this result needs to be taken with caution because CO1 sequences are not available for members of the *S. minus* clade 1. Boscaro *et al*.^6^ could not find any key morphological characters that would enable reliable discrimination between the two *S. minus* clades. So far, the classification of *S. minus*-like specimens into one of the two clades is based only on molecular information. Although it would be appropriate to create a new formal name for one of the *S. minus* clades, sequences from populations originally studied by Roux^58^ are needed to determine which clade corresponds to the “true” *S. minus*.

As concerns *S. teres*, both single and concatenated gene trees (except for trees inferred from the alpha tub-32 and the CON4-32 dataset; Fig. 3D,I) revealed this morphospecies to be non-monophyletic and its populations were consistently separated into two or four distinct clusters, depending on the dataset. Our previous results based on the primary and secondary structure of the ITS2 molecule could not support the existence of cryptic species in *S. teres*^12^. In this study, we analysed the genetic variability of the mitochondrial CO1 gene sequences at the population level. Our data suggested that the mean genetic diversity is significantly higher in the CO1 gene sequences (10.25% from 7 clones). Nevertheless, this is still below the 18% threshold that is considered as the mean intra-species variability in *Carchesium polypinum*^8^. The intra-specific genetic divergences in *S. teres* reach up to 20.81%, a value above that of *Carchesium* but distinctly below the 26% threshold in some arthropods^59^. Interestingly, about 19% sequence divergences were observed between two morphologically indistinguishable populations of the millipede *Bicoxidens flavicollis*^60^. Nonetheless, the present statistical tree topology tests (Supplementary Table S21) and delimitation analyses (Table 1) suggest that at least the population Mdg3 of the *S. teres* morphospecies might represent a distinct taxon. The erection of a new species is, however, prevented by the lack of type material from the Mdg3 population. As in *S. minus*, molecular data from type population of *S. teres* are needed to determine which populations correspond to the “true” *S. teres*.

In all phylogenetic analyses, *S. ambiguum* populations were grouped together. Specifically, they formed an unstructured cluster in all gene trees, except for the CO1 gene tree where they were classified into two sister lineages (Fig. 3E). Furthermore, the mean genetic divergence among nine *S. ambiguum* isolates was 11.63%, with a maximum of 20.46%, and there were 167 polymorphic nucleotide sites (38 taxa) in the CO1 nucleotide sequences. Such a high intra-specific divergence in CO1 gene sequences is considered as a presence of species complexes or cryptic species^8,61^. However, the high divergence between *S. ambiguum* populations is concentrated mostly in the insert region of the CO1 gene. The quickly evolving ITS region sequences and D1D2 domains of the 28S rRNA gene do not indicate the presence of cryptic species within *S. ambiguum* (Figs 1, 2A, 3A‒D,F,G). Therefore, we consider the nucleotide CO1 sequence variability to mostly reflect synonymous mutations in the insert region of the CO1 gene and not speciation processes. Conjugation experiments and further neutral evolving markers are, however, needed to test if *S. ambiguum* might contain cryptic species or not.

## Materials and Methods

### Sampling, species identification and processing

Samples were collected from different habitats and localities, as summarised in Supplementary Table S1. Collected material was immediately stored in an icebox and brought to the laboratory, where individual ciliate morphospecies were isolated and used to set clonal cultures in Petri dishes. Each clonal culture started from a single individual that was washed in sterile distilled water. Cultures were maintained at 18–24 °C and contained filtrated original medium and/or sterile seawater for marine species and commercial mineral water (Evian, France) for freshwater species. Wheat grains were periodically added to stimulate the growth of prey bacteria.

After one week, specimens from each clonal culture were examined under an optical microscope Zeiss Axio Imager A1 at low (50–400X) and high (1,000X, oil immersion) magnifications, using bright field and differential interference contrast optics. The protargol staining method was used to reveal the ciliary pattern and nuclear apparatus^62^. Species identification was performed according to the following studies: Berger *et al*.^27^, Boscaro *et al*.^6^, Foissner *et al*.^23^, Repak and Isquith^21^ and Shazib *et al*.^12^. Main morphological characters of the studied *Spirostomum* species were summarised in Supplementary Fig. S2 and Table S22.

### DNA extraction, PCR amplification and sequencing

After morphological identification, one or more cells from each identified population were isolated, washed several times in distilled water, and transferred into 10 µl extraction solution buffer (Sigma, St. Louis, MO, USA) in 1.5 ml microtubes. When more cells were collected from a population, they were kept separate and each cell represented a distinct sample. Subsequently, genomic DNA was extracted using the RED Extract-N-Amp Tissue PCR Kit (Sigma, St. Louis, MO), with modifications mentioned by Shazib *et al*.^12^. DNA amplifications were performed with polymerase chain reaction (PCR) using the TaKaRa Ex Taq polymerase kit, which has a higher fidelity than standard Taq polymerase with a mutation rate approximately 4.5 times lower (TaKaRa Bio-medicals, Otsu, Japan). Amplicons containing the 18S rRNA-ITS-28S rRNA region were obtained with the eukaryotic universal forward primer Euk A (5′-AAC CTG GTT GAT CCT GCC AG-3′)^63^ and the reverse primer D1D2-R2 (5′-ACG ATC GAT TTG CAC GTC AG-3′)^64^ under the PCR cycling conditions of Kim *et al*.^65^. The alpha tubulin gene sequences were amplified with the forward TUB-1 primer (5′-AAG GCT CTC TTG GCG TAC AT-3′) and the reverse TUB-2 primer (5′-TGA TGC CTT CAA CAC CTT CTT-3′)^66^. PCR conditions were as follows: 1 cycle for 5 min at 95 °C, 30 cycles for 1 min at 94 °C, 2 min at 60 °C and 2 min at 72 °C and 1 cycle for 10 min at 72 °C. The mitochondrial CO1 gene sequences were amplified using the forward F388dT primer (5′-TGT AAA ACG ACG GCC AGT GGW KCB AAA GAT GTW GC-3′) and the reverse R1184dT primer (5′-CAG GAA ACA GCT ATG ACT ADA CYT CAG GGT GAC CRA AAA ATC A-3′)^61^. We also slightly modified the CO1 primers as follows: 5′-GGN KCN AAA GAT GTW GC-3′ for the forward CO1-F388dT17 primer and 5′-CAG GGT GAC CGA AAA ATC-3′ for the reverse CO1-R1184dT18 primer by aligning all available *Spirostomum* CO1 gene sequences and checking their suitability with polymerase chain reaction. The condition of the PCR cycles for both sets of primers were: 1 cycle for 4 min at 94 °C, 40 cycles for 45 s at 94 °C, 75 s at 47 °C and 90 s at 72 °C and 1 final extension cycle for 10 min at 72 °C. The size of the amplified DNA was confirmed by electrophoresing in 1.2% agarose gel and 1X TAE buffer at 80 V for 50–60 min. PCR products were visualised with the SYBR Green I nucleic acid gel stain (Sigma Aldrich) and UV transillumination. Finally, PCR products were purified and bi-directional sequenced with PCR primers on an ABI 3730 automatic sequencer (Macrogen Inc., Seoul, South Korea). For ribosomal gene sequences, five additional internal primers were used as specified in Shazib *et al*.^12^.

### Sequence processing, datasets and alignment procedures

Sequencing chromatogram files were checked, trimmed, and assembled into contigs using the software Geneious ver. 8.1.7^67^ (http://www.geneious.com). The protein-coding genes were translated into amino acid sequences in Geneious with the ciliate nuclear genetic code for the alpha-tubulin gene and with the protozoan mitochondrial code for the CO1 gene to check for stop codons and frame shifts. Several datasets were constructed to examine the impact of taxonomic sampling on phylogenetic analyses (Table 2). Ribosomal gene sequences were aligned using the MAFFT algorithm and 100 bootstrap repeats on the online server GUIDANCE2^68^ (http://guidance.tau.ac.il/ver2/). The protein-coding genes were aligned based on the predicted amino acid sequences with MEGA ver. 6.06^69^. Unreliable and poorly aligned columns were removed from the final rRNA gene sequence alignments according to the calculated confidence scores suggested by the GUIDANCE2 algorithm. No masking strategy was employed for the alpha-tubulin and CO1 gene sequences, as all columns were aligned unambiguously. The GC content of each sequence was calculated in Geneious. Numbers of parsimony informative (Pi) sites were estimated from each alignment using the software PAUP* ver. 4.0b10^70^.

**Table 2:** Characterisation of the datasets analysed.

Dataset	No. of taxa	Molecular marker(s)	No. of char.	No. of PI char.	Evolutionary substitution model used in Bayesian analyses
18S-32	32	18S rRNA gene (=18S)	1580	63	GTR + I (=0.7750) + G (=0.6340)
ITS-32	32	ITS1-5.8S-ITS2 region (=ITS)	256	43	GTR + G (=0.1940)
28S-32	32	D1D2 domain of 28S rRNA gene (=28S)	495	50	GTR + I (=0.4570) + G (=0.4760)
alpha tub-32	32	Alpha-tubulin	936	203	GTR + I (=0.2560) + G (=0.2110)
CO1-32	32	Cytochrome oxidase subunit 1 (=CO1)	644	306	GTR + I (=0.3430) + G (=0.7290)
CON1-32	32	18S + ITS + 28S	2331	156	GTR + I (=0.6840) + G (=0.4610)
CON2-32	32	18S + ITS + 28S + alpha-tubulin	3267	359	GTR + I (=0.6180) + G (=0.3880)
CON3-32	32	Alpha-tubulin + CO1	1580	509	GTR + I (=0.5600) + G (2.1390)
CON4-32	32	18S + ITS + 28S + alpha-tubulin + CO1	3911	665	GTR + I (=0.5950) + G (=0.5640)
CON1-38	38	18S + ITS + 28S	2317	154	GTR + I (=0.6900) + G (=0.4635)
CON2-38	38	18S + ITS + 28S + CO1	2887	439	GTR + I (=0.6130) + G (=0.4930)
CON-89	89	18S + ITS + 28S	2428	333	GTR + I (=0.6850) + G (=0.5670)

The number of parsimony informative characters (PI char.) was calculated in PAUP* and the best evolutionary substitution model was selected in jModelTest under the Akaike information criterion.

### Distance analyses

Intraspecific as well as interspecific pairwise uncorrected *p*-distances and numbers of nucleotide differences were calculated separately in MEGA ver. 6.06^69^. All alignment positions with gaps were excluded from the distance analysis, using the complete and/or partial deletion option.

### Construction of gene trees

We conducted Bayesian Inference (BI) and Maximum Likelihood (ML) analyses on all alignments. The Akaike information criterion (AIC) calculated in jModelTest ver. 2.0.1^71,72^ was used to evaluate nucleotide substitution models of evolution for each dataset. The best fitting evolutionary models for all alignments are summarised in Table 2. Bayesian analyses were performed in MrBayes ver. 3.2.6^73^ using the best evolutionary model, whereby MCMC chains were one million steps long and every 100^th^ generation was sampled. The first 2,500 sampled trees were discarded as burn-in, leaving 7,500 trees for calculating majority rule consensus trees and posterior probabilities of their branching patterns. ML analyses with 1,000 bootstrap replicates were carried out using RAxML-HPC2 ver. 8.2.10 on the CIPRES Science Gateway ver. 3.3 (http://www.phylo.org/index.php/portal/v33) with the GTRCAT evolutionary model to account for heterogeneity rate^74–77^. Phylogenetic trees were visualised and edited using FigTree ver. 1.4 (http://tree.bio.ed.ac.uk/software/figtree/) and MEGA. In all datasets, *Anigstenia* species were considered as the outgroup taxa for rooting the trees.

### Construction of species trees, networks and Bayesian species delimitation

Species trees were calculated under the Bayesian multispecies coalescent model, using STACEY ver. 1.2.2^78^ implemented in the computer package BEAST ver. 2.4.5^79^. Input files were prepared in BEAUti with the following settings: (i) best evolutionary substitution models as selected by jModelTest for each partition; (ii) four categories for substitution rate heterogeneity; (iii) uncorrelated lognormal clock; (iv) ploidy scalars at 1.0 for the mitochondrial partition and 2.0 for the nuclear partitions; (v) the Yule process model for the species tree prior; and (vi) 200 million generations and a sampling frequency of 20,000 in Markov Chain Monte Carlo analyses. In total, six different datasets were analysed, as detailed in the Results section. The convergence to stationary distribution (effective sample size >200 for all parameters) was checked in Tracer ver. 1.6 for all analyses. The maximum clade credibility trees were summarised in TreeAnnotator ver. 1.8.1^80^ after discarding the first 10% of sampled trees.

A species network was constructed in PhyloNet ver. 3.6.1^81,82^, using the maximum pseudo-likelihood framework. The network was computed with 0, 5, and 10 maximum numbers of reticulation nodes from the 18S + ITS + 28S, CO1 and alpha-tubulin Bayesian consensus trees based on the CON1-32, CO1-32, and alpha tub-32 datasets, respectively. The maximum number of reticulation nodes was determined iteratively. Zero reticulations represented a null model corresponding to a species tree, while a maximum of either 5 or 10 reticulations served to test how many reticulations could be present in the resulting phylogenetic networks. If there were exactly 10 reticulation nodes, another round of analyses with an increased number of reticulations would be needed. However, phylogenetic networks with a maximum of six reticulations were recovered (see the Results section); hence, no further analyses were needed. Each analysis was performed with 10 runs and default settings, generating five optimal networks. The species networks were visualised with Dendroscope ver. 2.7.4^83^.

Bayesian species delimitation was conducted in BP&P ver. 2.2^84^, with the same datasets as used in the construction of species trees. Coalescent species trees obtained with STACEY served as guide trees for species delimitation. Each species delimitation model was assigned equal prior probability. Four different combinations of prior settings for the ancestral population size (*θ*) and root age (*τ*) were tested to examine the robustness of the results: relatively small ancestral population size and shallow divergences (*θ* = G[2, 1000], *τ* = G[2, 2000]), relatively large ancestral population size and deep divergences (*θ* = G[2, 100], *τ* = G[2, 200]), relatively large ancestral population size and shallow divergences (*θ* = G[2, 100], *τ* = G[2, 2000]), and relatively small ancestral population size and deep divergences (*θ* = G[2, 1000], *τ* = G[2, 200])^85^. The rjMCMC analyses were run for 100,000 generations with a sampling frequency of 2 and a burn-in of 10,000. A large fine-tuning parameter (*ε* = 15) was used to guarantee a good mixing in the reversible jump algorithm^84^. All analyses were conducted twice to confirm consistency between runs.

### Statistical tree topology tests

Topology tests were carried out to assess the monophyletic origins of (1) *Spirostomum* species with moniliform macronucleus, (2) *Spirostomum* species with compact macronucleus, (3) two *S. minus* clades, and (4) four *S. teres* clades. The approximately unbiased (AU), the weighted Kishino-Hasegawa (WKH) and the weighted Shimodaira-Hasegawa (WSH) test were conducted, as implemented in CONSEL ver. 0.1^86–88^. The unconstrained and constrained ML trees, and their site-wise likelihoods were calculated in PAUP* ver. 4.0b10^70^ under the best evolutionary models using the ML criterion, heuristic search, TBR branch swapping and 10 random sequence addition replications.

### Selection tests

Gene-wide tests for positive selection acting on the alpha-tubulin and CO1 genes were performed with BUSTED (Branch-site Unrestricted Statistical Test for Episodic Diversification) on the Datamonkey Adaptive Evolution Server^89–91^. BUSTED simultaneously estimates the proportion of sites belonging to each of three *ω* classes. It holds *ω*_1_ ≤ *ω*_2_ ≤ 1 ≤ *ω*_3_ in the unconstrained model, while *ω*_3_ = 1 in the constrained null model disallowing positive selection. If the null hypothesis is rejected, then there is evidence that at least one site has, at least some of the time, experienced positive selection^92^.

## Conclusions

Our analyses strongly statistically supported the following previous hypotheses about the genus *Spirostomum*: (1) taxa with compact macronucleus and taxa with moniliform macronucleus each form a distinct lineage; (2) the morphospecies *S. minus* contains two morphologically cryptic taxa; and (3) the morphospecies *S. teres* is non-monophyletic and defined by a set of ancestral features of lineages that also gave origin to *S. yagiui* and *S. dharwarensis*. Our analyses further revealed that ribosomal RNA genes and their spacers bear phylogenetic signal – which is consistent with species trees – and therefore have the highest phylogenetic informativeness and delimitation power in *Spirostomum*. On the other hand, the protein-coding CO1 and alpha-tubulin genes are useful in population structure analyses but might not have power to resolve phylogenetic relationships among *Spirostomum* species, possibly due to the purifying selection. The problem of purifying selection is especially pronounced in the gene coding for alpha-tubulin whose usage is therefore not recommended in species delimitation analyses of the genus *Spirostomum*, although this gene is highly variable.

## Supplementary information

Supplementary material

## Data Availability

Accession numbers for the newly obtained DNA sequences are 18S rRNA (MK688522 – MK688553); ITS1-5.8S-ITS2 (MK721433 – MK721465); 28S rRNA™(MK713375 – MK713407); alpha-tubulin (MK721466 – MK721497); CO1 (MK721498 – MK721535)  listed in Supplementary Table S1 and are available at https://www.ncbi.nlm.nih.gov/nucleotide/. Results of all analyses are included in this published article and its Supplementary Information files. The datasets generated and/or analysed during the current study are available from the corresponding author on reasonable request.
