# Supplementary material for: Deciphering phylogenetic relationships and delimiting species boundaries using a Bayesian coalescent approach in protists: A case study of the ciliate genus Spirostomum (Ciliophora, Heterotrichea)
Source: Sci Rep. 2019 Nov 8;9:16360. doi: 10.1038/s41598-019-52722-4 (PMC6841689; doi:10.1038/s41598-019-52722-4)
Supplement: Supplementary file 1 — Supplementary material [file 41598_2019_52722_MOESM1_ESM.pdf]

Supplementary material

for DOI : 10.1038/s41598-019-52722-4 in **Scientific Reports**

Deciphering phylogenetic relationships and delimiting species boundaries using a Bayesian coalescent approach in protists: A case study of the ciliate genus *Spirostomum* (Ciliophora, Heterotrichea)

Shahed Uddin Ahmed Shazib[1,a], Peter Vďačný[2,a], Marek Slovák[3,4], Eleni Gentekaki[5] & Mann Kyoon Shin[1*]

[1]Department of Biological Science, University of Ulsan, Ulsan 44610, South Korea. [2]Department of Zoology, Comenius University in Bratislava, 842 15 Bratislava, Slovakia. [3]Plant Science and Biodiversity Centre, Institute of Botany, Slovak Academy of Sciences, 845 23 Bratislava, Slovakia. [4]Department of Botany, Charles University, 128 01 Prague, Czech Republic. [5]School of Science, Mae Fah Luang University, Chiang Rai 57100, Thailand. Correspondence and requests for materials should be addressed to M.K.S. (email: mkshin@ulsan.ac.kr)

[a]Both authors contributed equally.

Pages: 36

Figures: 2

Tables: 22

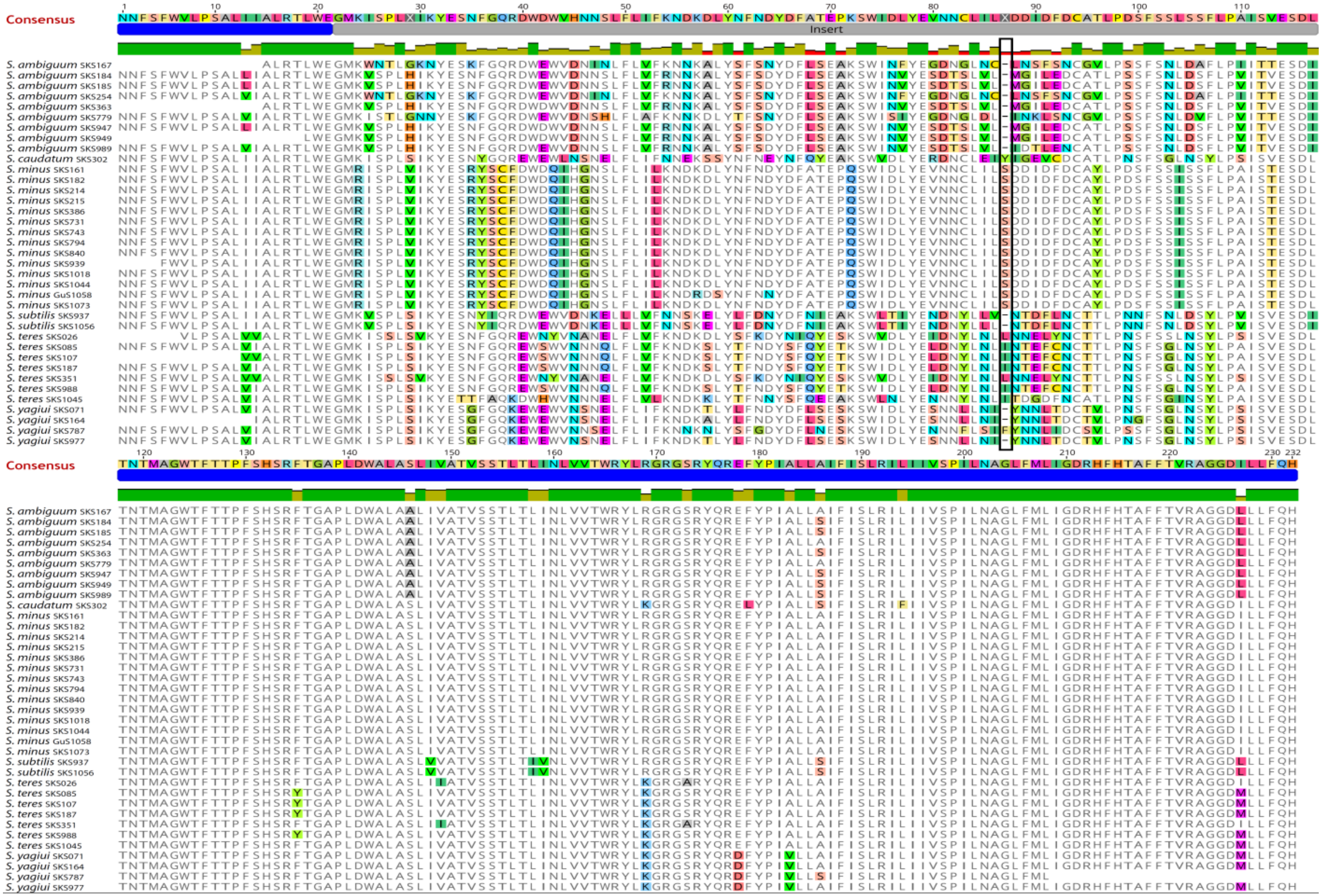

Supplementary Figure S1. Mitochondrial CO1 amino acid sequence alignment of 37 *Spirostomum* populations. An indel position is marked with a box.

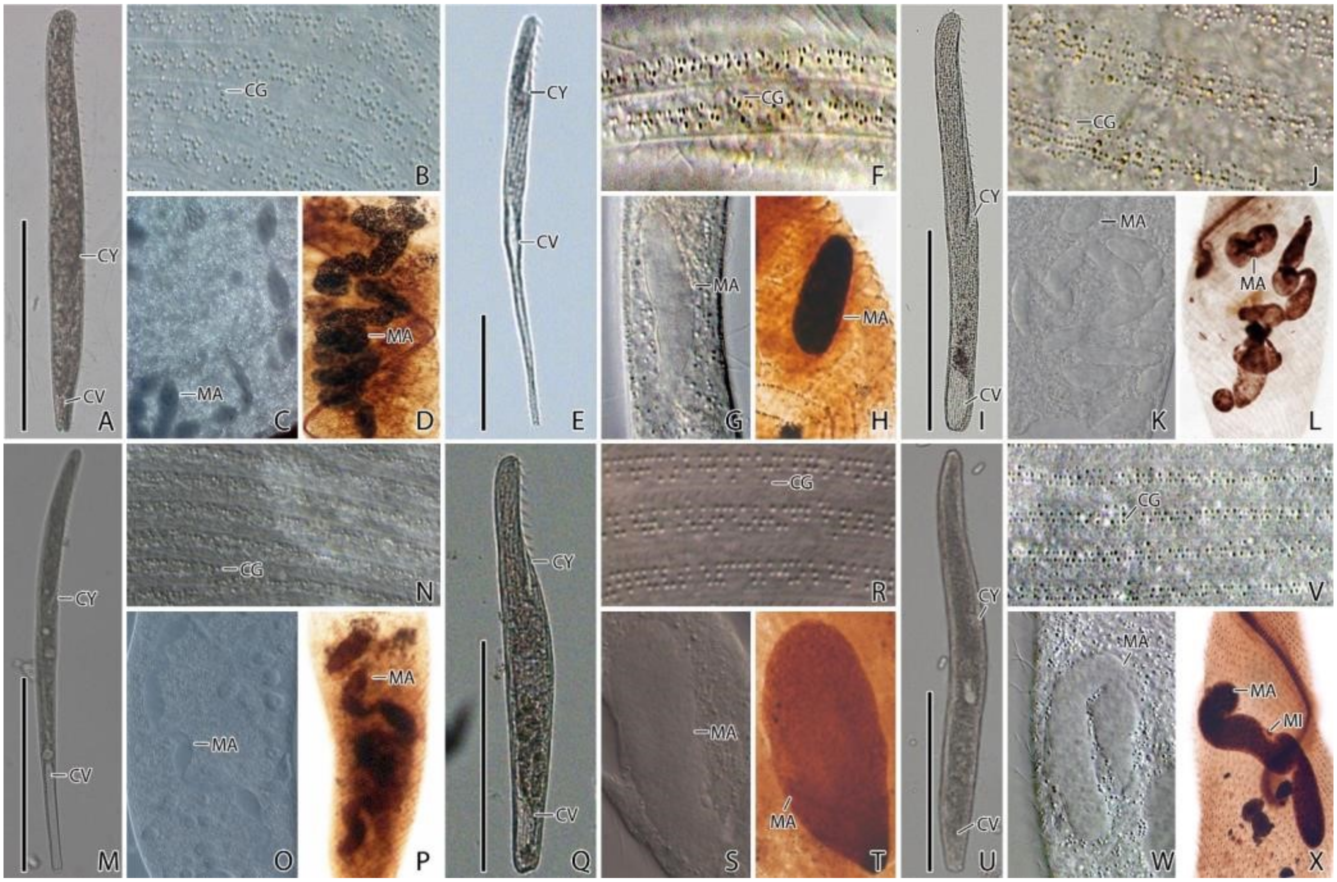

Supplementary Figure S2. Six *Spirostomum* species in vivo and after protargol impregnation investigated in the present study. Overviews showing the body shape (A, E, I, M, Q, U), arrangement of cortical granules (B, F, J, N, R, V), nuclear apparatus in vivo (C, G, K, O, S, W) and after protargol impregnation (D, H, L, P, T, X). (**A–D**) *Spirostomum ambiguum* is characterized by having a peristome occupying about 45–78% of the body length, a moniliform macronucleus, a truncated posterior body end, and 2–5 cortical granule rows between adjacent somatic kineties. **(E–H)** *Spirostomum caudatum* is distinguished by having a peristome occupying about 18–20% of the body length, an ellipsoid macronucleus, a tail-like posterior body end, and 2–3 cortical granule rows between adjacent somatic kineties. **(I–L)** *Spirostomum minus* has a peristome occupying about 40–65% of the body length, a moniliform macronucleus, a truncated posterior body end, and 2–5 cortical granule rows between adjacent somatic kineties. **(M–P)** *Spirostomum subtilis* is characterized by having a peristome occupying about 28–61% of the body length, a moniliform macronucleus, a truncated posterior body end, and 1–4 cortical granule rows between adjacent somatic kineties. **(Q–T)** *Spirostomum teres* has a peristome occupying about 31–56% of the body length, an ellipsoid macronucleus, a truncated posterior body end, and 2–6 cortical granule rows between adjacent somatic kineties. **(U–X)** *Spirostomum yagiui* is characterized by having a peristome occupying about 21–59% of the body length, a curved cylindroid macronucleus, a truncated posterior body end, and 2–6 cortical granule rows between adjacent somatic kineties. CG, cortical granules; CV, contractile vacuole; CY, cytostome; MA, macronucleus; MI, micronucleus. Scale bars: Scale bars: 100 µm (E), 200 µm (I, Q, U), and 500 µm (A, M).

Supplementary Table S1. Sampling sites and characterisation of the 18S rRNA gene, ITS region, D1D2 domain of the 28S rRNA gene, alpha-tubulin gene and mitochondrial CO1 gene for a total of 86 *Spirostomum* populations. Species are arranged alphabetically, and new sequences are in bold face. Sequence information has been updated for the SKS026 and SKS085 populations previously described by Shazib *et al*. (2014).

Population ID	Species name	18S rRNA			ITS1-5.8S-ITS2			28S rRNA			a-tubulin			CO1			Habitat and collection location	Collection date	References
		GB accession number	Length (nt)	GC (%)	GB accession number	Length (nt)	GC (%)	GB accession number	Length (nt)	GC (%)	GB accession number	Length (nt)	GC (%)	GB accession number	Length (nt)	GC (%)			
QinCHN	*Anigsteinia clarissima*	KM222109	1705	46.3	KM222077	263	47.5	KM222166	700	50.3	----	----	----	----	----	----	No. 1 Bathing Beach, Qingdao, China	Not provided	Gao *et al*. (2016)
SKS670	*Anigsteinia* sp.	KU848241	1669	46.5	KR861718	257	48.2	KU848225	792	51.0	----	----	----	----	----	----	Brackish water from Taehwa river, Bangu-dong, Ulsan, South Korea	March 2014	Shazib *et al*. (2016)
SKS686	*Anigsteinia* sp.	KU848242	1676	46.6	KR861719	256	48.8	KU848226	785	51.2	**MK721466**	993	54.5	**MK721498**	744	26.7	Brackish water from Taehwa river, Taehwa-dong, Ulsan, South Korea	March 2014	Shazib *et al*. (2016)
SKS167	*Spirostomum ambiguum*	KJ651819	1598	48.6	KJ651852	257	56.0	KJ651836	686	57.0	**MK721467**	950	59.8	**MK721503**	654	31.2	Freshwater pond (Meonmulkkak pond), Seonheul-ri, Jocheon-eup, Jeju, South Korea	February 2012	Shazib *et al*. (2014)
SKS363	*Spirostomum ambiguum*	KU848236	1670	48.1	KR861713	257	56.0	KU848220	766	55.2	**MK721470**	950	59.5	**MK721505**	654	33.2	Freshwater pond (Jeonggol pond), Mugeo-dong, Ulsan, South Korea	July 2013	Shazib *et al*. (2016)
SKS1050	*Spirostomum ambiguum*	**MK688526**	1670	48.1	**MK721438**	257	56.0	**MK713380**	694	56.3	----	----	----	----	----	----	Freshwater pond (Jeonggol pond), Mugeo-dong, Ulsan, South Korea	March 2016	Present study
SKS947	*Spirostomum ambiguum*	**MK688523**	1624	48.2	**MK721435**	257	56.0	**MK713377**	694	56.3	----	----	----	**MK721501**	696	32.9	Freshwater pond (Jeonggol pond), Mugeo-dong, Ulsan, South Korea	April 2015	Present study
SKS949	*Spirostomum ambiguum*	**MK688524**	1637	48.3	**MK721436**	257	56.0	**MK713378**	694	56.3	**MK721472**	950	59.5	**MK721502**	642	32.6	Freshwater pond (Jeonggol pond), Mugeo-dong, Ulsan, South Korea	April 2015	Present study
EU201	*Spirostomum ambiguum*	AM398201	1701	47.8	AM398201	257	56.0	AM398201	583	54.5	----	----	----	----	----	----	Not provided	Not provided	Schmidt *et al*. (2007)
SS4-2	*Spirostomum ambiguum*	HG939551	1702	47.8	HG939551	257	56.0	HG939551	505	53.1	----	----	----	----	----	----	Freshwater pond inside the Skansen Open Air Museum, Djurgarden, Stockholm, Sweden	August 2013	Boscaro *et al*. (2014)
SA	*Spirostomum ambiguum*	KP970238	1605	48.4	KP970292	258	56.6	KP970266	692	56.5	----	----	----	----	----	----	Freswater pond near the beach in Ilha Grande, Brazil	Not provided	Fernandes *et al*. (2016)
SKS185	*Spirostomum ambiguum*	KU848231	1611	48.3	KR861708	257	56.0	KU848215	721	55.9	**MK721468**	950	59.5	**MK721500**	696	33.0	Freshwater from natural wetland in Ulsan Grand Park, Sinjeong-dong, Ulsan, South Korea	June 2012	Shazib *et al*. (2016)
SKS204	*Spirostomum ambiguum*	KU848232	1601	48.5	KR861709	257	56.0	KU848216	716	56.1	----	----	----	----	----	----	Freshwater pond in Dongbaekdongsan Hill, Seonheul-ri, Jocheon-eup, Jeju, South Korea	February 2012	Shazib *et al*. (2016)
SKS254	*Spirostomum ambiguum*	KU848233	1671	48.2	KR861710	257	56.0	KU848217	764	55.6	**MK721469**	950	59.8	**MK721504**	696	30.7	Freshwater pond in Dongbaekdongsan Hill, Seonheul-ri, Jocheon-eup, Jeju, South Korea	September 2012	Shazib *et al*. (2016)
SKS779	*Spirostomum ambiguum*	KU848240	1671	48.0	KR861717	257	56.0	KU848224	776	55.4	**MK721471**	950	59.5	**MK721506**	696	30.0	Brakish water from Gyeongpoho pond, Jeo-dong, Gangneung, Gangwon, South Korea	June 2014	Shazib *et al*. (2016)
SKS169	*Spirostomum ambiguum*	**MK688527**	1671	48.1	**MK721433**	257	56.0	**MK713375**	694	56.3	----	----	----	----	----	----	Freshwater from natural wetland in Ulsan Grand Park, Sinjeong-dong, Ulsan, South Korea	June 2012	Present study
SKS184	*Spirostomum ambiguum*	**MK688522**	1675	48.2	**MK721434**	257	56.0	**MK713376**	694	56.3	----	----	----	**MK721499**	696	33.0	Freshwater from natural wetland in Ulsan Grand	June 2012	Present study

Population ID	Species name	18S rRNA			ITS1-5.8S-ITS2			28S rRNA			a-tubulin			CO1			Habitat and collection location	Collection date	References
		GB accession number	Length (nt)	GC (%)	GB accession number	Length (nt)	GC (%)	GB accession number	Length (nt)	GC (%)	GB accession number	Length (nt)	GC (%)	GB accession number	Length (nt)	GC (%)			
																	Park, Sinjeong-dong, Ulsan, South Korea		
SKS989	*Spirostomum ambiguum*	**MK688525**	1627	48.2	**MK721437**	257	56.0	**MK713379**	694	56.3	**MK721473**	950	59.5	**MK721507**	696	32.0	Freshwater pond in Baekdong-ri, Ungsang-eup, Yangsan, Gyeongnam, South Korea	May 2007	Present study
SKS813	*Spirostomum caudatum*	**MK688528**	1670	47.8	**MK721439**	253	53.4	**MK713381**	688	56.4	**MK721474**	950	59.8	----	----	----	Freshwater wetland (Gyeongancheon wetland), Jeongji-ri, Toechon-myeon, Gwangju, Gyeonggi, South Korea	August 2014	Present study
SKS302	*Spirostomum caudatum*	KJ651820	1597	48.1	KJ651853	254	52.8	KJ651837	685	55.8	----	----	----	**MK721508**	699	28.0	Freshwater pond (Wollangji), Nansan-ri, Seongsan-eup, Seogwipo, Jeju, South Korea	November 2012	Shazib *et al.* (2014)
SAd	*Spirostomum dharwarensis*	HG939537	1702	48.0	HG939537	251	55.0	HG939537	412	52.9	----	----	----	----	----	----	Estuary, Mtentu River, Province of Eastern Cape, South Africa	May 2013	Boscaro *et al.* (2014)
SmPS	*Spirostomum minus*	HG939546	1702	47.9	HG939546	254	53.5	HG939546	566	55.5	----	----	----	----	----	----	Freshwater waterway close to the Aurelia road, Pisa. Italy	May 2012	Boscaro *et al.* (2014)
PBG1	*Spirostomum minus*	HG939547	1702	47.9	HG939547	254	53.5	HG939547	528	54.5	----	----	----	----	----	----	Freshwater (artificial) pond, Botanical Garden, Pisa, Italy	May 2013	Boscaro *et al.* (2014)
SS5	*Spirostomum minus*	HG939548	1702	47.9	HG939548	254	54.2	HG939548	546	55.1	----	----	----	----	----	----	Freshwater pond inside the Skansen Open Air Museum, Djurgarden, Stockholm, Sweden	August 2013	Boscaro *et al.* (2014)
SKS182	*Spirostomum minus*	KU848230	1626	48.3	KR861707	254	53.5	KU848214	735	56.3	**MK721476**	950	58.8	**MK721510**	699	29.6	Freshwater from natural wetland in Ulsan Grand Park, Sinjeong-dong, Ulsan, South Korea	June 2012	Shazib *et al.* (2016)
SKS386	*Spirostomum minus*	KU848237	1675	48.2	KR861714	254	53.5	KU848221	768	56.1	**MK721478**	941	58.8	**MK721513**	699	29.6	Freshwater from Mugeocheon stream, Mugeo-dong, Ulsan, South Korea	April 2013	Shazib *et al.* (2016)
GuS1058	*Spirostomum minus*	**MK688537**	1644	48.4	**MK721448**	254	54.3	**MK713390**	513	57.1	**MK721486**	950	58.7	**MK721521**	699	30.5	Freshwater from Asan River, Asan, Guam, Micronesia	July 2016	Present study
SKS1018	*Spirostomum minus*	**MK688535**	1671	48.2	**MK721446**	254	53.5	**MK713388**	694	57.2	**MK721484**	950	58.8	**MK721519**	699	29.5	Freshwater marsh (Sinpyeongji), Sinpyeong-ri, Toseong-myeon, Goseong-gun, Gangwon, South Korea	December 2014	Present study
SKS1073	*Spirostomum minus*	**MK688538**	1648	48.2	**MK721449**	255	53.7	**MK713391**	517	57.1	**MK721487**	950	58.8	**MK721522**	699	29.5	Freshwater from Mineral spring ditch, Muryongsan Mt., Yeonam-dong, Ulsan, South Korea	March 2017	Present study
EU200	*Spirostomum minus*	AM398200	1701	47.6	AM398200	257	51.8	AM398200	583	56.3	----	----	----	----	----	----	Not provided	Not provided	Schmidt *et al.* (2007)
Mdg2-2	*Spirostomum minus*	HG939539	1702	47.6	HG939539	257	52.1	HG939539	431	52.7	----	----	----	----	----	----	Freshwater lake (Ambandrika Lake), Sainte Luce, Madagascar	July 2011	Boscaro *et al.* (2014)
Mdg4	*Spirostomum minus*	HG939540	1702	47.6	HG939540	257	52.1	HG939540	566	56.2	----	----	----	----	----	----	Freshwater swamp in a forest, Mandena, Madagascar	January 2012	Boscaro *et al.* (2014)
Gmn	*Spirostomum minus*	HG939543	1702	47.6	HG939543	257	51.8	HG939543	434	53.0	----	----	----	----	----	----	Freshwater stream, Nymphenburg Park, Munich, Germany	April 2011	Boscaro *et al.* (2014)
SAm	*Spirostomum minus*	HG939545	1702	47.6	HG939545	257	51.8	HG939545	493	54.2	----	----	----	----	----	----	Estuary, Mtentu River, Province of Eastern Cape, South Africa	May 2013	Boscaro *et al.* (2014)
SKS215	*Spirostomum minus*	KJ651821	1597	48.5	KJ651854	254	53.5	KJ651838	686	57.6	----	----	----	**MK721512**	699	29.5	Ephemeral freshwater puddle in Gukdang-1-ri,	July 2012	Shazib *et al.* (2014)

Population ID	Species name	18S rRNA			ITS1-5.8S-ITS2			28S rRNA			a-tubulin			CO1			Habitat and collection location	Collection date	References
		GB accession number	Length (nt)	GC (%)	GB accession number	Length (nt)	GC (%)	GB accession number	Length (nt)	GC (%)	GB accession number	Length (nt)	GC (%)	GB accession number	Length (nt)	GC (%)			
																	Gangdong-myeon, Gyeongju, Gyeongbuk, South Korea		
SKS161	*Spirostomum minus*	KU848229	1604	48.5	KR861706	254	53.5	KU848213	718	56.5	**MK721475**	950	58.8	**MK721509**	699	29.8	Brackish water (Deahwacheon stream), Mopo-ri, Janggi-myeon, Pohang, Gyeongbuk, South Korea	March 2012	Shazib *et al.* (2016)
SKS731	*Spirostomum minus*	KU848238	1671	48.3	KR861715	254	53.1	KU848222	583	55.6	**MK721479**	950	58.8	**MK721514**	699	29.6	Freshwater pond (Heogiji reservoir), Gumi-ri, Dudong-myeon, Ulsan, South Korea	May 2014	Shazib *et al.* (2016)
SKS743	*Spirostomum minus*	KU848239	1671	48.2	KR861716	254	53.5	KU848223	767	56.1	**MK721480**	950	58.8	**MK721515**	699	29.8	Freshwater in ricefield, Hwajeong-ri, Seosaeng-myeon, Ulsan, South Korea	May 2014	Shazib *et al.* (2016)
SKS1044	*Spirostomum minus*	**MK688536**	1669	48.2	**MK721447**	254	53.5	**MK713389**	694	57.2	**MK721485**	950	58.8	**MK721520**	699	29.6	Freshwater reservoir (Cheonjinho), Bongpo-ri, Toseong-myeon, Goseong-gun, Gangwon, South Korea	January 2016	Present study
SKS214	*Spirostomum minus*	**MK688529**	1671	48.2	**MK721440**	254	53.5	**MK713382**	694	57.2	**MK721477**	950	58.8	**MK721511**	699	29.5	Ephemeral freshwater puddle in Gukdang-1-ri, Gangdong-myeon, Gyeongju, Gyeongbuk, South Korea	July 2012	Present study
SKS794	*Spirostomum minus*	**MK688530**	1670	48.2	**MK721441**	254	53.9	**MK713383**	694	57.2	**MK721481**	944	58.9	**MK721516**	699	29.3	Freshwater pond (Nonmul pond), Ojo-ri, Seongsan-eup, Seogwipo, Jeju, South Korea	June 2014	Present study
SKS840	*Spirostomum minus*	**MK688531**	1609	48.4	**MK721442**	254	53.5	**MK713384**	694	57.2	**MK721482**	950	58.8	**MK721517**	699	29.5	Freshwater pond, Naeheung-ri, Gyeryong-myeon, Gongju, Chungnam, South Korea	October 2014	Present study
SKS939	*Spirostomum minus*	**MK688532**	1670	48.2	**MK721443**	241	53.1	**MK713385**	630	57.6	**MK721483**	950	58.8	**MK721518**	687	29.8	Freshwater pond, Myeongdo-ri, Uljin-eup, Uljin, Gyeongbuk, South Korea	February 2015	Present study
SKS940	*Spirostomum minus*	**MK688533**	1671	48.2	**MK721444**	254	53.9	**MK713386**	694	57.2	----	----	----	----	----	----	Freshwater pond, Myeongdo-ri, Uljin-eup, Uljin, Gyeongbuk, South Korea	February 2015	Present study
SKS991	*Spirostomum minus*	**MK688534**	1613	48.1	**MK721445**	257	51.8	**MK713387**	579	57.7	----	----	----	----	----	----	Freshwater wetland (Guemsa wetland), Geumsa-ri, Geumsa-myeon, Yeoju, Gyeonggi, South Korea	August 2007	Present study
DoEng	*Spirostomum semivirescens*	MH295830	1722	47.7	MH295830	254	54.3	MH295831	757	55.7	----	----	----	----	----	----	Freshwater fen pond, Dorset, South England	June 2015	Hines *et al.* (2018)
GuaCHN	*Spirostomum* sp.	FJ998027	1718	48.1	KM222081	258	55.8	KM222170	691	55.1	----	----	----	----	----	----	Freshwater pond in the campus of the South China Normal University, Guangzhou	Not provided	Gao *et al.* (2016)
StMgN	*Spirostomum sp.*	HG939526	1702	48.0	HG939526	252	55.4	HG939526	490	54.1	----	----	----	----	----	----	Freshwater stream in a forest, Mandena, Madagascar	January 2012	Boscaro *et al.* (2014)
StJFp2	*Spirostomum* sp.	HG939531	1702	48.1	HG939531	251	55.0	HG939531	563	56.1	----	----	----	----	----	----	Freshwater eutrophic stream, (Sao Pedro stream) Juiz de Fora, Brazil	June 2012	Boscaro *et al.* (2014)
SmJFp1	*Spirostomum* sp.	HG939541	1702	47.7	HG939541	257	52.3	HG939541	481	53.6	----	----	----	----	----	----	Freshwater eutrophic stream, (Sao Pedro stream), Juiz de Fora, Brazil	February 2013	Boscaro *et al.* (2014)

Population ID	Species name	18S rRNA			ITS1-5.8S-ITS2			28S rRNA			a-tubulin			CO1			Habitat and collection location	Collection date	References
		GB accession number	Length (nt)	GC (%)	GB accession number	Length (nt)	GC (%)	GB accession number	Length (nt)	GC (%)	GB accession number	Length (nt)	GC (%)	GB accession number	Length (nt)	GC (%)			
Thd2	*Spirostomum* sp.	HG939542	1702	47.6	HG939542	257	51.8	HG939542	528	55.1	----	----	----	----	----	----	Freshwater eutrophic stream, Vung Tàu, Thailand	November 2011	Boscaro *et al.* (2014)
Ind	*Spirostomum* sp.	HG939544	1702	47.4	HG939544	257	52.1	HG939544	528	54.5	----	----	----	----	----	----	Freshwater pond in cave (Borra Caves) Visakhapatnam, India	March 2013	Boscaro *et al.* (2014)
Seef2	*Spirostomum* sp.	HG939552	1702	47.8	HG939552	257	56.0	HG939552	527	53.3	----	----	----	----	----	----	Freshwater lake (Wildsee Lake) Seefeld in Tirol, Austria	August 2013	Boscaro *et al.* (2014)
SKS255	*Spirostomum* sp.	KU848234	1617	48.4	KR861711	256	53.9	KU848218	761	55.5	----	----	----	----	----	----	Freshwater pond in Dongbaekdongsan Hill, Seonheul-ri, Jocheon-eup, Jeju, South Korea	September 2012	Shazib *et al.* (2016)
Zur3	*Spirostomum subtilis*	HG939550	1702	47.1	HG939550	257	56.0	HG939550	528	53.0	----	----	----	----	----	----	Freshwater sample from the Zurichsee, Arboretum, Zurich, Switzerland	February 2013	Boscaro *et al.* (2014)
SKS1056	*Spirostomum subtilis*	**MK688540**	1623	47.5	**MK721451**	257	56.0	**MK713393**	512	55.3	**MK721489**	950	56.9	**MK721524**	696	27.7	Freshwater pond, Gangdong-dong, Gangseo-gu, Busan, South Korea	June 2016	Present study
SKS937	*Spirostomum subtilis*	**MK688539**	1670	47.4	**MK721450**	257	56.0	**MK713392**	694	55.9	**MK721488**	950	57.1	**MK721523**	696	27.2	Freshwater pond, Myeongdo-ri, Uljin-eup, Uljin, Gyeongbuk, South Korea	February 2015	Present study
SS4-1	*Spirostomum subtilis*	HG939549	1702	47.1	HG939549	257	56.0	HG939549	518	52.8	----	----	----	----	----	----	Freshwater pond inside the Skansen Open Air Museum, Djurgarden, Stockholm, Sweden	August 2013	Boscaro *et al.* (2014)
**SKS085****	*Spirostomum teres*	KJ651822	1598	48.7	**MK721452**	250	55.2	KJ651839	683	57.8	**MK721491**	950	56.8	**MK721526**	699	26.3	Freshwater stream, Gusu-ri, Eonyang-eup, Uljugun, Ulsan, South Korea	April 2012	Shazib *et al.* (2014)
SKS107	*Spirostomum teres*	KU848228	1629	48.5	KR861705	251	55.0	KU848212	730	56.4	----	----	----	**MK721527**	663	26.5	Freshwater stream (Namcheonnae stream), Eoeum-ri, Eonyang-eup, Ulsan, South Korea	April 2012	Shazib *et al.* (2016)
SKS351	*Spirostomum teres*	KU848235	1670	48.4	KR861712	251	55.0	KU848219	584	56.8	**MK721493**	950	58.5	**MK721529**	699	25.8	Freshwater wetland (Cheongsu Gotjawal wetland), Cheongsu-ri, Hangyeong-myeon, Jeju, South Korea	October 2011	Shazib *et al.* (2016)
**SKS026****	*Spirostomum teres*	**MK688541**	1609	48.6	KJ651855	251	55.0	**MK713394**	691	58.2	**MK721490**	929	58.4	**MK721525**	681	26.1	Freshwater wetland, Cheongsu-ri, Hangyeong-myeon, Jeju, South Korea	January 2012	Shazib *et al.* (2016)
SKS1045	*Spirostomum teres*	**MK688544**	1624	48.2	**MK721455**	255	53.7	**MK713397**	691	56.7	**MK721494**	950	58.7	**MK721531**	699	29.8	Freshwater reservoir (Cheonjinho), Bongpo-ri, Toseong-myeon, Goseong-gun, Gangwon, South Korea	January 2016	Present study
SKS187	*Spirostomum teres*	**MK688542**	1673	48.4	**MK721453**	251	55.0	**MK713395**	691	57.5	**MK721492**	950	56.9	**MK721528**	699	26.2	Freshwater from natural wetland in Ulsan Grand Park, Sinjeong-dong, Ulsan, South Korea	June 2012	Present study
SKS988	*Spirostomum teres*	**MK688543**	1612	48.6	**MK721454**	251	55.0	**MK713396**	691	57.5	----	----	----	**MK721530**	699	26.3	Freshwater pond, Guyeong-ri, Beomseo-eup, Ulsan, South Korea	February 2007	Present study
EU199	*Spirostomum teres*	AM398199	1701	48.1	AM398199	251	55.0	AM398199	581	55.9	----	----	----	----	----	----	Not provided	Not provided	Schmidt *et al.* (2007)
Mdg2-1	*Spirostomum teres*	HG939524	1702	48.1	HG939524	254	54.3	HG939524	429	53.1	----	----	----	----	----	----	Fresh water lake in Ambandrika, Sainte Luce, Madagascar	June 2011	Boscaro *et al.* (2014)
Seef1	*Spirostomum teres*	HG939525	1702	48.1	HG939525	254	54.3	HG939525	435	53.1	----	----	----	----	----	----	Freshwater lake (Wildsee Lake) Seefeld in Tirol, Austria	August 2013	Boscaro *et al.* (2014)
NorBG	*Spirostomum teres*	HG939527	1702	48.1	HG939527	251	55.0	HG939527	525	55.0	----	----	----	----	----	----	Freshwater (artificial) pond, University	August 2013	Boscaro *et al.* (2014)

Population ID	Species name	18S rRNA			ITS1-5.8S-ITS2			28S rRNA			a-tubulin			CO1			Habitat and collection location	Collection date	References
		GB accession number	Length (nt)	GC (%)	GB accession number	Length (nt)	GC (%)	GB accession number	Length (nt)	GC (%)	GB accession number	Length (nt)	GC (%)	GB accession number	Length (nt)	GC (%)			
																	Botanical Garden, Oslo, Norway		
NorKD	*Spirostomum teres*	HG939528	1702	48.1	HG939528	251	55.0	HG939528	478	54.2	----	----	----	----	----	----	Freshwater artificial pond, Royal Palace Park, Oslo, Norway	August 2013	Boscaro *et al.* (2014)
PFEU3Sm2	*Spirostomum teres*	HG939529	1702	48.1	HG939529	251	55.0	HG939529	471	54.1	----	----	----	----	----	----	Freshwater eutrophic marsh, (Fucecchio Marsh), Pistoia, Italy	September 2012	Boscaro *et al.* (2014)
PF1a	*Spirostomum teres*	HG939532	1702	47.9	HG939532	250	55.6	HG939532	561	56.0	----	----	----	----	----	----	Freshwater oligotrophic marsh, (Fucecchio Marsh), Pistoia, Italy	October 2013	Boscaro *et al.* (2014)
Mdg3	*Spirostomum teres*	HG939538	1702	48.2	HG939538	253	53.0	HG939538	520	55.4	----	----	----	----	----	----	Brackish (1.0%) lake, Ambandrika, Sainte Luce, Madagascar	July 2011	Boscaro *et al.* (2014)
SPT	*Spirostomum teres*	KP970241	1643	48.3	KP970295	256	53.1	KP970268	691	56.9	----	----	----	----	----	----	Sludge treatment plant in Rio de Janeiro, Brazil	Not provided	Fernandes *et al.* (2016)
JpH1020	*Spirostomum yagiui*	**MK688553**	1671	48.1	**MK721465**	253	53.0	**MK713407**	689	57.9	----	----	----	----	----	----	Freshwater, Hiroshima, Japan, sample from Prof. Suzaki T. (Kobe University, Japan)	2013	Present study
SKS315	*Spirostomum yagiui*	**MK688547**	1676	48.2	**MK721458**	253	53.0	**MK713400**	689	57.9	----	----	----	----	----	----	Brackish water (Geowugae wetland), Pyoseon-ri, Pyoseon-myeon, Seogwipo, Jeju, South Korea	November 2012	Present study
SKS583	*Spirostomum yagiui*	**MK688548**	1672	48.1	**MK721459**	253	53.0	**MK713401**	689	57.9	----	----	----	----	----	----	Brackish water (salinity 11‰, Taehwa river), Samsan-dong, Ulsan, South Korea	January 2014	Present study
SKS977	*Spirostomum yagiui*	**MK688551**	1671	48.1	**MK721463**	253	53.0	**MK713405**	689	57.9	**MK721496**	950	57.1	**MK721535**	696	28.9	Freshwater pond (Jeonggol pond), Mugeo-dong, Ulsan, South Korea	August 2015	Present study
SKS983	*Spirostomum yagiui*	**MK688552**	1671	48.1	**MK721464**	253	53.0	**MK713406**	689	57.9	----	----	----	----	----	----	Freshwater pond (Jeonggol pond), Mugeo-dong, Ulsan, South Korea	August 2015	Present study
LarnCyp	*Spirostomum yagiui*	HG939533	1702	47.9	HG939533	253	53.0	HG939533	561	56.9	----	----	----	----	----	----	Brackish (0.5%) littoral stream, Larnaca, Republic Of Cyprus	August 2012	Boscaro *et al.* (2014)
SpirWS	*Spirostomum yagiui*	HG939534	1702	47.9	HG939534	253	53.0	HG939534	561	56.9	----	----	----	----	----	----	Brackish (1.5%) littoral zone of Kerit Island, in the Kandalashka Gulf (Republic of Karelia, White Sea), Russia	August 2011	Boscaro *et al.* (2014)
GNS4	*Spirostomum yagiui*	HG939535	1702	47.9	HG939535	253	53.0	HG939535	426	53.1	----	----	----	----	----	----	Brackish (1.0%) littoral zone, North Sea, Duhnen, Germany	August 2013	Boscaro *et al.* (2014)
Pozz	*Spirostomum yagiui*	HG939536	1702	47.9	HG939536	253	53.0	HG939536	412	52.9	----	----	----	----	----	----	Freshwater pond near the littoral zone, Pozzallo, Ragusa, Italy	October 2013	Boscaro *et al.* (2014)
SKS164	*Spirostomum yagiui*	**MK688545**	1674	48.1	**MK721456**	253	53.0	**MK713398**	689	57.9	**MK721497**	950	57.2	**MK721533**	657	28.3	Brackish water (Deahwacheon stream), Mopo-ri, Janggi-myeon, Pohang, Gyeongbuk, South Korea	March 2012	Present study
SKS165	*Spirostomum yagiui*	**MK688546**	1607	48.4	**MK721457**	253	53.0	**MK713399**	689	57.9	----	----	----	----	----	----	Brackish water (Deahwacheon stream), Mopo-ri, Janggi-myeon, Pohang, Gyeongbuk, South Korea	March 2012	Present study
SKS963	*Spirostomum yagiui*	**MK688549**	1610	48.3	**MK721461**	253	53.0	**MK713403**	689	57.9	----	----	----	----	----	----	Brackish water (Taehwa river, Samho bridge), Taehwa-dong, Ulsan, South Korea	June 2015	Present study
SKS964	*Spirostomum yagiui*	**MK688550**	1671	48.1	**MK721462**	253	53.0	**MK713404**	689	57.9	----	----	----	----	----	----	Brackish water (Taehwa river, Bamboo park	June 2015	Present study

Population ID	Species name	18S rRNA			ITS1-5.8S-ITS2			28S rRNA			a-tubulin			CO1			Habitat and collection location	Collection date	References
		GB accession number	Length (nt)	GC (%)	GB accession number	Length (nt)	GC (%)	GB accession number	Length (nt)	GC (%)	GB accession number	Length (nt)	GC (%)	GB accession number	Length (nt)	GC (%)			
																	north), Taehwa-dong, Ulsan, South Korea		
SKS071	*Spirostomum yagiui*	KU848227	1682	48.0	KR861704	253	53.0	KU848211	762	57.0	**MK721495**	950	57.1	**MK721532**	696	29.0	Estuarine brakish water (salinity 0.6 ‰), Woldaecheon stream, Jeju, South Korea	February 2012	Shazib *et al*. (2016)
SKS787	*Spirostomum yagiui*	KX119519	1668	48.1	**MK721460**	253	53.0	**MK713402**	689	57.9	----	----	----	**MK721534**	624	24.5	Estuarine brackish water (salinity 1‰) in Geumjin-ri, Okgye-myeon, Gangneung , Gangwon, South Korea	June 2014	Chen *et al*. (2017)

Supplementary Table S2. Inter- and intraspecific variability of five genetic markers in *Spirostomum* species.

Alignment	No. of clones	Mean divergence	Standard error	Minimum divergence	Maximum divergence
**89 taxa**<br>**18S rRNA**					
Spirostomum ambiguum	15	0.13%	0.06%	0.00%	0.32%
Spirostomum caudatum	2	0.32%	0.14%	- - -	- - -
Spirostomum minus	24	0.37%	0.09%	0.00%	0.96%
Spirostomum subtilis	4	0.00%	0.00%	0.00%	0.00%
Spirostomum teres	16	0.53%	0.10%	0.00%	1.66%
Spirostomum yagiui	15	0.00%	0.00%	0.00%	0.00%
Spirostomum spp. (interspecific)	81	1.68%	0.01%	0.13%	3.19%
All *Spirostomum* spp.	86	1.41%	0.20%	0.00%	3.19%
**ITS1-5.8S-ITS2 regions**					
Spirostomum ambiguum	15	0.05%	0.05%	0.00%	0.39%
Spirostomum caudatum	2	1.58%	0.76%	- - -	- - -
Spirostomum minus	24	2.57%	0.57%	0.00%	7.09%
Spirostomum subtilis	4	0.00%	0.00%	0.00%	0.00%
Spirostomum teres	16	3.39%	0.64%	0.00%	8.37%
Spirostomum yagiui	15	0.00%	0.00%	0.00%	0.00%
Spirostomum spp. (interspecific)	81	9.61%	0.06%	0.40%	14.74%
All *Spirostomum* spp.	86	8.02%	1.06%	0.00%	14.74%
**D1D2 region of 28S rRNA**					
Spirostomum ambiguum	15	0.23%	0.12%	0.00%	0.58%
Spirostomum caudatum	2	2.04%	0.50%	- - -	- - -
Spirostomum minus	24	1.91%	0.34%	0.00%	6.60%
Spirostomum subtilis	4	0.52%	0.25%	0.00%	0.90%
Spirostomum teres	16	2.32%	0.40%	0.00%	5.02%
Spirostomum yagiui	15	0.00%	0.00%	0.00%	0.00%
Spirostomum spp. (interspecific)	81	5.66%	0.04%	0.00%	10.95%
All *Spirostomum* spp.	86	4.91%	0.54%	0.00%	10.95%

**Alignment**	**No. of clones**	**Mean divergence**	**Standard error**	**Minimum divergence**	**Maximum divergence**
**38 taxa**<br>**18S rRNA**					
Spirostomum ambiguum	9	0.12%	0.06	0.00%	0.25%
Spirostomum minus	14	0.01%	0.01%	0.00%	0.06%
Spirostomum subtilis	2	0.00%	0.00%	- - -	- - -
Spirostomum teres	7	0.31%	0.08%	0.00%	0.76%
Spirostomum yagiui	4	0.00%	0.00%	0.00%	0.00%
Spirostomum spp. (interspecific)	37	1.71%	0.02%	0.70%	3.30%
All *Spirostomum* spp.	37	1.33%	0.17%	0.00%	3.30%
**ITS1-5.8S-ITS2 regions**					
Spirostomum ambiguum	9	0.00%	0.00%	0.00%	0.00%
Spirostomum minus	14	0.28%	0.17%	0.00%	1.28%
Spirostomum subtilis	2	0.00%	0.00%	- - -	- - -
Spirostomum teres	7	2.12%	0.54%	0.00%	5.98%
Spirostomum yagiui	4	0.00%	0.00%	0.00%	0.00%
Spirostomum spp. (interspecific)	37	8.61%	0.16%	1.28%	13.68%
All *Spirostomum* spp.	37	6.71%	1.00%	0.00%	13.68%
**D1D2 region of 28S rRNA**					
Spirostomum ambiguum	9	0.19%	0.13%	0.00%	0.42%
Spirostomum minus	14	0.00%	0.00%	0.00%	0.00%
Spirostomum subtilis	2	0.00%	0.00%	- - -	- - -
Spirostomum teres	7	1.66%	0.36%	0.00%	4.42%
Spirostomum yagiui	4	0.00%	0.00%	0.00%	0.00%
Spirostomum spp. (interspecific)	37	6.41%	0.10%	2.74%	9.68%
All *Spirostomum* spp.	37	4.98%	0.61%	0.00%	9.68%
**mt CO1 region**					
Spirostomum ambiguum (nt)	9	11.63%	0.80%	0.00%	20.46%
Spirostomum ambiguum (aa)	9	8.67%	1.41%	0.00%	15.87%
Spirostomum minus (nt)	14	0.44%	0.13%	0.00%	2.47%
Spirostomum minus (aa)	14	0.23%	0.13%	0.00%	1.59%

Alignment	No. of clones	Mean divergence	Standard error	Minimum divergence	Maximum divergence
Spirostomum subtilis (nt)	2	1.41%	0.49%	- - -	- - -
Spirostomum subtilis (aa)	2	0.53%	0.51%	- - -	- - -
Spirostomum teres (nt)	7	10.25%	0.74%	0.00%	20.81%
Spirostomum teres (aa)	7	8.57%	1.32%	0.00%	17.99%
Spirostomum yagiui (nt)	4	8.35%	0.81%	1.23%	12.70%
Spirostomum yagiui (aa)	4	4.14%	1.02	0.00%	7.94%
Spirostomum spp. (interspecific) (nt)	37	24.98%	0.14%	16.75%	30.34%
Spirostomum spp. (interspecific) (aa)	37	23.28%	0.14%	14.29%	30.16%
All *Spirostomum* spp. (nt)	37	20.26%	0.89%	0.00%	30.34%
All *Spirostomum* spp. (aa)	37	18.67%	1.94%	0.00%	30.16%
**mt CO1 region (without insert)**					
Spirostomum ambiguum (nt)	9	8.58%	1.08%	0.00%	15.25%
Spirostomum ambiguum (aa)	9	0.53%	0.51%	0.00%	1.06%
Spirostomum minus (nt)	14	0.30%	0.12%	0.00%	2.13%
Spirostomum minus (aa)	14	0.00%	0.00%	0.00%	0.00%
Spirostomum subtilis (nt)	2	2.13%	0.85%	- - -	- - -
Spirostomum subtilis (aa)	2	0.00%	0.00%	- - -	- - -
Spirostomum teres (nt)	7	8.37%	1.06%	0.00%	17.02%
Spirostomum teres (aa)	7	1.62%	0.89%	0.00%	3.19%
Spirostomum yagiui (nt)	4	6.50%	1.06%	1.42%	9.57%
Spirostomum yagiui (aa)	4	0.53%	0.51%	0.00%	1.06%
Spirostomum spp. (interspecific) (nt)	37	17.17%	0.08%	11.70%	20.92%
Spirostomum spp. (interspecific) (aa)	37	3.30%	0.06%	1.06%	7.45%
All *Spirostomum* spp. (nt)	37	14.00%	1.26%	0.00%	20.92%
All *Spirostomum* spp. (aa)	37	2.62%	0.86%	0.00%	7.45%
**32 taxa**<br>**18S rRNA**					
Spirostomum ambiguum	7	0.14%	0.07%	0.00%	0.25%
Spirostomum minus	13	0.01%	0.01%	0.00%	0.06%
Spirostomum subtilis	2	0.00%	0.00%	- - -	- - -
Spirostomum teres	5	0.39%	0.10%	0.00%	0.76%

Alignment	No. of clones	Mean divergence	Standard error	Minimum divergence	Maximum divergence
Spirostomum yagiui	3	0.00%	0.00%	0.00%	0.00%
Spirostomum spp. (interspecific)	31	1.72%	0.03%	0.70%	3.30%
All *Spirostomum* spp.	31	1.32%	0.17%	0.00%	3.30%
**ITS1-5.8S-ITS2 regions**					
Spirostomum ambiguum	7	0.00%	0.00%	0.00%	0.00%
Spirostomum minus	13	0.30%	0.18%	0.00%	1.28%
Spirostomum subtilis	2	0.00%	0.00%	- - -	- - -
Spirostomum teres	5	2.82%	0.74%	0.00%	5.98%
Spirostomum yagiui	3	0.00%	0.00%	0.00%	0.00%
Spirostomum spp. (interspecific)	31	8.46%	0.19%	1.28%	13.68%
All *Spirostomum* spp.	31	6.51%	1.01%	0.00%	13.68%
**D1D2 region of 28S rRNA**					
Spirostomum ambiguum	7	0.22%	0.15%	0.00%	0.42%
Spirostomum minus	13	0.00%	0.00%	0.00%	0.00%
Spirostomum subtilis	2	0.00%	0.00%	- - -	- - -
Spirostomum teres	5	2.19%	0.44%	0.00%	4.42%
Spirostomum yagiui	3	0.00%	0.00%	0.00%	0.00%
Spirostomum spp. (interspecific)	31	6.32%	0.12%	2.74%	9.68%
All *Spirostomum* spp.	31	4.84%	0.60%	0.00%	9.68%
**alpha tubulin**					
Spirostomum ambiguum (nt)	7	0.15%	0.08%	0.00%	0.32%
Spirostomum ambiguum (aa)	7	0.00%	0.00%	0.00%	0.00%
Spirostomum minus (nt)	13	0.07%	0.03%	0.00%	0.32%
Spirostomum minus (aa)	13	0.00%	0.00%	0.00%	0.00%
Spirostomum subtilis (nt)	2	0.11%	0.11%	- - -	- - -
Spirostomum subtilis (aa)	2	0.00%	0.00%	0.00%	0.00%
Spirostomum teres (nt)	5	7.41%	0.64%	0.00%	10.01%
Spirostomum teres (aa)	5	0.00%	0.00%	0.00%	0.00%
Spirostomum yagiui (nt)	3	0.36%	0.16%	0.32%	0.43%
Spirostomum yagiui (aa)	3	0.00%	0.00%	0.00%	0.00%

Alignment	No. of clones	Mean divergence	Standard error	Minimum divergence	Maximum divergence
Spirostomum spp. (interspecific) (nt)	31	10.82%	0.06%	7.21%	12.81%
Spirostomum spp. (interspecific) (aa)	31	0.00%	0.00%	0.00%	0.00%
All *Spirostomum* spp. (nt)	31	8.37%	0.60%	0.00%	12.81%
All *Spirostomum* spp. (aa)	31	0.00%	0.00%	0.00%	0.00%
**mt CO1 region**					
Spirostomum ambiguum (nt)	7	12.83%	0.87%	0.00%	19.31%
Spirostomum ambiguum (aa)	7	8.94%	1.40%	0.00%	14.08%
Spirostomum minus (nt)	13	0.48%	0.14%	0.00%	2.34%
Spirostomum minus (aa)	13	0.22%	0.12%	0.00%	1.40%
Spirostomum subtilis (nt)	2	1.40%	0.49%	- - -	- - -
Spirostomum subtilis (aa)	2	0.47%	0.44%	- - -	- - -
Spirostomum teres (nt)	5	12.40%	0.89%	0.00%	19.63%
Spirostomum teres (aa)	5	9.95%	1.49%	0.00%	16.43%
Spirostomum yagiui (nt)	3	4.36%	0.62%	1.09%	6.07%
Spirostomum yagiui (aa)	3	0.63%	0.42%	0.00%	0.94%
Spirostomum spp. (interspecific) (nt)	31	24.04%	0.14%	16.51%	28.50%
Spirostomum spp. (interspecific) (aa)	31	21.43%	0.16%	12.21%	26.76%
All *Spirostomum* spp. (nt)	31	19.15%	0.88%	0.00%	28.50%
All *Spirostomum* spp. (aa)	31	16.88%	1.75%	0.00%	26.76%
**mt CO1 region (without insert)**					
Spirostomum ambiguum (nt)	7	9.56%	1.08%	0.00%	14.29%
Spirostomum ambiguum (aa)	7	0.48%	0.48%	0.00%	0.85%
Spirostomum minus (nt)	13	0.39%	0.16%	0.00%	1.96%
Spirostomum minus (aa)	13	0.00%	0.00%	0.00%	0.00%
Spirostomum subtilis (nt)	2	1.96%	0.73%	- - -	- - -
Spirostomum subtilis (aa)	2	0.00%	0.00%	- - -	- - -
Spirostomum teres (nt)	5	10.25%	1.08%	0.00%	15.97%
Spirostomum teres (aa)	5	2.03%	0.96%	0.00%	3.39%
Spirostomum yagiui (nt)	3	4.29%	0.86%	1.12%	6.16%
Spirostomum yagiui (aa)	3	0.00%	0.00%	0.00%	0.00%

Alignment	No. of clones	Mean divergence	Standard error	Minimum divergence	Maximum divergence
Spirostomum spp. (interspecific) (nt)	31	16.70%	0.07%	13.17%	20.17%
Spirostomum spp. (interspecific) (aa)	31	3.29%	0.06%	1.69%	6.78%
All *Spirostomum* spp. (nt)	31	13.39%	1.05%	0.00%	20.17%
All *Spirostomum* spp. (aa)	31	2.56%	0.80%	0.00%	6.78%

Notes: aa, amino acids; nt, nucleotides.

Supplementary Table S3. Numbers of nucleotide differences (above diagonal) and pairwise distances (below diagonal) of the 18S rRNA gene sequences among 86 *Spirostomum* populations.

Taxa	1	2	3	4	5	6	7	8	9	10	11	12	13	14	15	16	17	18	19	20	21	22	23	24	25	26	27	28	29	30	31	32	33	34	35	36	37	38	39	40	41	42	43	44	45	46	47	48	49	50	51	52	53	54	55	56	57	58	59	60	61	62	63	64	65	66	67	68	69	70	71	72	73	74	75	76	77	78	79	80	81	82	83	84	85	86
1. *S. ambiguum* SKS363		4	4	2	0	0	0	0	5	1	1	0	0	0	4	38	40	35	21	21	21	21	21	21	21	21	26	21	21	21	25	26	21	25	26	26	21	21	21	21	21	21	18	33	33	33	33	37	37	33	40	37	37	37	39	39	40	39	37	34	37	37	34	35	35	35	35	35	35	35	35	35	35	35	35	35	35	35	32	26	21	1	40	6	27	36
2. *S. ambiguum* SKS254	0.26%		0	4	4	4	4	4	3	3	3	4	4	4	0	40	42	35	21	21	21	21	21	21	21	21	26	21	21	21	25	26	21	25	26	26	21	21	21	21	21	21	18	33	33	33	33	37	37	33	40	37	37	37	39	39	40	39	37	34	37	37	34	35	35	35	35	35	35	35	35	35	35	35	35	35	35	35	32	26	21	3	40	4	27	36
3. *S. ambiguum* SKS204	0.26%	0.00%		4	4	4	4	4	3	3	3	4	4	4	0	40	42	35	21	21	21	21	21	21	21	21	26	21	21	21	25	26	21	25	26	26	21	21	21	21	21	21	18	33	33	33	33	37	37	33	40	37	37	37	39	39	40	39	37	34	37	37	34	35	35	35	35	35	35	35	35	35	35	35	35	35	35	35	32	26	21	3	40	4	27	36
4. *S. ambiguum* SKS779	0.13%	0.26%	0.26%		2	2	2	2	5	1	1	2	2	2	4	40	42	33	19	19	19	19	19	19	19	19	26	19	19	19	25	26	19	25	26	26	19	19	19	19	19	19	16	31	31	31	31	35	35	31	38	35	35	35	37	37	40	39	35	34	35	35	34	33	33	33	33	33	33	33	33	33	33	33	33	33	33	33	32	26	19	1	40	6	27	34
5. *S. ambiguum* SKS989	0.00%	0.26%	0.26%	0.13%		0	0	0	5	1	1	0	0	0	4	38	40	35	21	21	21	21	21	21	21	21	26	21	21	21	25	26	21	25	26	26	21	21	21	21	21	21	18	33	33	33	33	37	37	33	40	37	37	37	39	39	40	39	37	34	37	37	34	35	35	35	35	35	35	35	35	35	35	35	35	35	35	35	32	26	21	1	40	6	27	36
6. *S. ambiguum* SKS949	0.00%	0.26%	0.26%	0.13%	0.00%		0	0	5	1	1	0	0	0	4	38	40	35	21	21	21	21	21	21	21	21	26	21	21	21	25	26	21	25	26	26	21	21	21	21	21	21	18	33	33	33	33	37	37	33	40	37	37	37	39	39	40	39	37	34	37	37	34	35	35	35	35	35	35	35	35	35	35	35	35	35	35	35	32	26	21	1	40	6	27	36
7. *S. ambiguum* SKS947	0.00%	0.26%	0.26%	0.13%	0.00%	0.00%		0	5	1	1	0	0	0	4	38	40	35	21	21	21	21	21	21	21	21	26	21	21	21	25	26	21	25	26	26	21	21	21	21	21	21	18	33	33	33	33	37	37	33	40	37	37	37	39	39	40	39	37	34	37	37	34	35	35	35	35	35	35	35	35	35	35	35	35	35	35	35	32	26	21	1	40	6	27	36
8. *S. ambiguum* SKS185	0.00%	0.26%	0.26%	0.13%	0.00%	0.00%	0.00%		5	1	1	0	0	0	4	38	40	35	21	21	21	21	21	21	21	21	26	21	21	21	25	26	21	25	26	26	21	21	21	21	21	21	18	33	33	33	33	37	37	33	40	37	37	37	39	39	40	39	37	34	37	37	34	35	35	35	35	35	35	35	35	35	35	35	35	35	35	35	32	26	21	1	40	6	27	36
9. *S. ambiguum* SA	0.32%	0.19%	0.19%	0.32%	0.32%	0.32%	0.32%	0.32%		4	4	5	5	5	3	38	40	33	19	19	19	19	19	19	19	19	24	19	19	19	23	24	19	23	24	24	19	19	19	19	19	19	16	31	31	31	31	35	35	31	38	35	35	35	37	37	40	39	35	34	35	35	34	33	33	33	33	33	33	33	33	33	33	33	33	33	33	33	30	24	19	4	38	1	25	34
10. *S. ambiguum* SS4-2	0.06%	0.19%	0.19%	0.06%	0.06%	0.06%	0.06%	0.06%	0.26%		0	1	1	1	3	39	41	34	20	20	20	20	20	20	20	20	25	20	20	20	24	25	20	24	25	25	20	20	20	20	20	20	17	32	32	32	32	36	36	32	39	36	36	36	38	38	39	38	36	33	36	36	33	34	34	34	34	34	34	34	34	34	34	34	34	34	34	34	31	25	20	0	39	5	26	35
11. *S. ambiguum* EU201	0.06%	0.19%	0.19%	0.06%	0.06%	0.06%	0.06%	0.06%	0.26%	0.00%		1	1	1	3	39	41	34	20	20	20	20	20	20	20	20	25	20	20	20	24	25	20	24	25	25	20	20	20	20	20	20	17	32	32	32	32	36	36	32	39	36	36	36	38	38	39	38	36	33	36	36	33	34	34	34	34	34	34	34	34	34	34	34	34	34	34	34	31	25	20	0	39	5	26	35
12. *S. ambiguum* SKS1050	0.00%	0.26%	0.26%	0.13%	0.00%	0.00%	0.00%	0.00%	0.32%	0.06%	0.06%		0	0	4	38	40	35	21	21	21	21	21	21	21	21	26	21	21	21	25	26	21	25	26	26	21	21	21	21	21	21	18	33	33	33	33	37	37	33	40	37	37	37	39	39	40	39	37	34	37	37	34	35	35	35	35	35	35	35	35	35	35	35	35	35	35	35	32	26	21	1	40	6	27	36
13. *S. ambiguum* SKS184	0.00%	0.26%	0.26%	0.13%	0.00%	0.00%	0.00%	0.00%	0.32%	0.06%	0.06%	0.00%		0	4	38	40	35	21	21	21	21	21	21	21	21	26	21	21	21	25	26	21	25	26	26	21	21	21	21	21	21	18	33	33	33	33	37	37	33	40	37	37	37	39	39	40	39	37	34	37	37	34	35	35	35	35	35	35	35	35	35	35	35	35	35	35	35	32	26	21	1	40	6	27	36
14. *S. ambiguum* SKS169	0.00%	0.26%	0.26%	0.13%	0.00%	0.00%	0.00%	0.00%	0.32%	0.06%	0.06%	0.00%	0.00%		4	38	40	35	21	21	21	21	21	21	21	21	26	21	21	21	25	26	21	25	26	26	21	21	21	21	21	21	18	33	33	33	33	37	37	33	40	37	37	37	39	39	40	39	37	34	37	37	34	35	35	35	35	35	35	35	35	35	35	35	35	35	35	35	32	26	21	1	40	6	27	36
15. *S. ambiguum* SKS167	0.26%	0.00%	0.00%	0.26%	0.26%	0.26%	0.26%	0.26%	0.19%	0.19%	0.19%	0.26%	0.26%	0.26%		40	42	35	21	21	21	21	21	21	21	21	26	21	21	21	25	26	21	25	26	26	21	21	21	21	21	21	18	33	33	33	33	37	37	33	40	37	37	37	39	39	40	39	37	34	37	37	34	35	35	35	35	35	35	35	35	35	35	35	35	35	35	35	32	26	21	3	40	4	27	36
16. *S. caudatum* SKS813	2.43%	2.55%	2.55%	2.55%	2.43%	2.43%	2.43%	2.43%	2.43%	2.49%	2.49%	2.43%	2.43%	2.43%	2.55%		5	26	32	32	32	32	32	32	32	32	30	32	32	32	29	30	32	29	30	30	32	32	32	32	32	32	33	48	48	48	48	22	22	25	32	22	22	22	26	26	23	22	22	22	22	22	22	24	24	24	24	24	24	24	24	24	24	24	24	24	24	24	34	30	32	39	23	39	31	21
17. *S. caudatum* SKS302	2.55%	2.68%	2.68%	2.68%	2.55%	2.55%	2.55%	2.55%	2.55%	2.62%	2.62%	2.55%	2.55%	2.55%	2.68%	0.32%		28	34	34	34	34	34	34	34	34	31	34	34	34	30	31	34	30	31	31	34	34	34	34	34	34	35	50	50	50	50	24	24	27	32	24	24	24	26	26	23	22	24	24	24	24	24	26	26	26	26	26	26	26	26	26	26	26	26	26	26	26	33	31	34	41	25	41	32	23
18. *S. dharwarensis* SAd	2.23%	2.23%	2.23%	2.11%	2.23%	2.23%	2.23%	2.23%	2.11%	2.17%	2.17%	2.23%	2.23%	2.23%	2.23%	1.66%	1.79%		24	24	24	24	24	24	24	24	30	24	24	24	31	30	24	31	30	30	24	24	24	24	24	24	25	34	34	34	34	11	11	4	20	11	11	11	11	11	20	19	11	13	11	11	13	2	2	2	2	2	2	2	2	2	2	2	2	2	2	2	32	30	26	34	15	34	31	10
19. *S. minus* SKS215	1.34%	1.34%	1.34%	1.21%	1.34%	1.34%	1.34%	1.34%	1.21%	1.28%	1.28%	1.34%	1.34%	1.34%	1.34%	2.04%	2.17%	1.53%		0	0	0	0	0	0	0	15	0	0	0	14	15	0	14	15	15	0	0	0	0	0	0	3	24	24	24	24	21	21	24	30	21	21	21	25	25	32	31	21	24	21	21	24	22	22	22	22	22	22	22	22	22	22	22	22	22	22	22	21	15	2	20	28	20	16	20
20. *S. minus* SKS386	1.34%	1.34%	1.34%	1.21%	1.34%	1.34%	1.34%	1.34%	1.21%	1.28%	1.28%	1.34%	1.34%	1.34%	1.34%	2.04%	2.17%	1.53%	0.00%		0	0	0	0	0	0	15	0	0	0	14	15	0	14	15	15	0	0	0	0	0	0	3	24	24	24	24	21	21	24	30	21	21	21	25	25	32	31	21	24	21	21	24	22	22	22	22	22	22	22	22	22	22	22	22	22	22	22	21	15	2	20	28	20	16	20
21. *S. minus* SKS731	1.34%	1.34%	1.34%	1.21%	1.34%	1.34%	1.34%	1.34%	1.21%	1.28%	1.28%	1.34%	1.34%	1.34%	1.34%	2.04%	2.17%	1.53%	0.00%	0.00%		0	0	0	0	0	15	0	0	0	14	15	0	14	15	15	0	0	0	0	0	0	3	24	24	24	24	21	21	24	30	21	21	21	25	25	32	31	21	24	21	21	24	22	22	22	22	22	22	22	22	22	22	22	22	22	22	22	21	15	2	20	28	20	16	20
22. *S. minus* SKS161	1.34%	1.34%	1.34%	1.21%	1.34%	1.34%	1.34%	1.34%	1.21%	1.28%	1.28%	1.34%	1.34%	1.34%	1.34%	2.04%	2.17%	1.53%	0.00%	0.00%	0.00%		0	0	0	0	15	0	0	0	14	15	0	14	15	15	0	0	0	0	0	0	3	24	24	24	24	21	21	24	30	21	21	21	25	25	32	31	21	24	21	21	24	22	22	22	22	22	22	22	22	22	22	22	22	22	22	22	21	15	2	20	28	20	16	20
23. *S. minus* SKS182	1.34%	1.34%	1.34%	1.21%	1.34%	1.34%	1.34%	1.34%	1.21%	1.28%	1.28%	1.34%	1.34%	1.34%	1.34%	2.04%	2.17%	1.53%	0.00%	0.00%	0.00%	0.00%		0	0	0	15	0	0	0	14	15	0	14	15	15	0	0	0	0	0	0	3	24	24	24	24	21	21	24	30	21	21	21	25	25	32	31	21	24	21	21	24	22	22	22	22	22	22	22	22	22	22	22	22	22	22	22	21	15	2	20	28	20	16	20
24. *S. minus* SKS214	1.34%	1.34%	1.34%	1.21%	1.34%	1.34%	1.34%	1.34%	1.21%	1.28%	1.28%	1.34%	1.34%	1.34%	1.34%	2.04%	2.17%	1.53%	0.00%	0.00%	0.00%	0.00%	0.00%		0	0	15	0	0	0	14	15	0	14	15	15	0	0	0	0	0	0	3	24	24	24	24	21	21	24	30	21	21	21	25	25	32	31	21	24	21	21	24	22	22	22	22	22	22	22	22	22	22	22	22	22	22	22	21	15	2	20	28	20	16	20
25. *S. minus* SKS939	1.34%	1.34%	1.34%	1.21%	1.34%	1.34%	1.34%	1.34%	1.21%	1.28%	1.28%	1.34%	1.34%	1.34%	1.34%	2.04%	2.17%	1.53%	0.00%	0.00%	0.00%	0.00%	0.00%	0.00%		0	15	0	0	0	14	15	0	14	15	15	0	0	0	0	0	0	3	24	24	24	24	21	21	24	30	21	21	21	25	25	32	31	21	24	21	21	24	22	22	22	22	22	22	22	22	22	22	22	22	22	22	22	21	15	2	20	28	20	16	20
26. *S. minus* SKS940	1.34%	1.34%	1.34%	1.21%	1.34%	1.34%	1.34%	1.34%	1.21%	1.28%	1.28%	1.34%	1.34%	1.34%	1.34%	2.04%	2.17%	1.53%	0.00%	0.00%	0.00%	0.00%	0.00%	0.00%	0.00%		15	0	0	0	14	15	0	14	15	15	0	0	0	0	0	0	3	24	24	24	24	21	21	24	30	21	21	21	25	25	32	31	21	24	21	21	24	22	22	22	22	22	22	22	22	22	22	22	22	22	22	22	21	15	2	20	28	20	16	20
27. *S. minus* SKS991	1.66%	1.66%	1.66%	1.66%	1.66%	1.66%	1.66%	1.66%	1.53%	1.60%	1.60%	1.66%	1.66%	1.66%	1.66%	1.91%	1.98%	1.91%	0.96%	0.96%	0.96%	0.96%	0.96%	0.96%	0.96%	0.96%		15	15	15	1	0	15	1	0	0	15	15	15	15	15	15	16	33	33	33	33	30	30	30	39	30	30	30	34	34	35	34	30	27	30	30	27	30	30	30	30	30	30	30	30	30	30	30	30	30	30	30	6	0	17	25	31	25	1	29
28. *S. minus* SKS743	1.34%	1.34%	1.34%	1.21%	1.34%	1.34%	1.34%	1.34%	1.21%	1.28%	1.28%	1.34%	1.34%	1.34%	1.34%	2.04%	2.17%	1.53%	0.00%	0.00%	0.00%	0.00%	0.00%	0.00%	0.00%	0.00%	0.96%		0	0	14	15	0	14	15	15	0	0	0	0	0	0	3	24	24	24	24	21	21	24	30	21	21	21	25	25	32	31	21	24	21	21	24	22	22	22	22	22	22	22	22	22	22	22	22	22	22	22	21	15	2	20	28	20	16	20
29. *S. minus* SKS794	1.34%	1.34%	1.34%	1.21%	1.34%	1.34%	1.34%	1.34%	1.21%	1.28%	1.28%	1.34%	1.34%	1.34%	1.34%	2.04%	2.17%	1.53%	0.00%	0.00%	0.00%	0.00%	0.00%	0.00%	0.00%	0.00%	0.96%	0.00%		0	14	15	0	14	15	15	0	0	0	0	0	0	3	24	24	24	24	21	21	24	30	21	21	21	25	25	32	31	21	24	21	21	24	22	22	22	22	22	22	22	22	22	22	22	22	22	22	22	21	15	2	20	28	20	16	20
30. *S. minus* SKS840	1.34%	1.34%	1.34%	1.21%	1.34%	1.34%	1.34%	1.34%	1.21%	1.28%	1.28%	1.34%	1.34%	1.34%	1.34%	2.04%	2.17%	1.53%	0.00%	0.00%	0.00%	0.00%	0.00%	0.00%	0.00%	0.00%	0.96%	0.00%	0.00%		14	15	0	14	15	15	0	0	0	0	0	0	3	24	24	24	24	21	21	24	30	21	21	21	25	25	32	31	21	24	21	21	24	22	22	22	22	22	22	22	22	22	22	22	22	22	22	22	21	15	2	20	28	20	16	20
31. *S. minus* Gmn	1.60%	1.60%	1.60%	1.60%	1.60%	1.60%	1.60%	1.60%	1.47%	1.53%	1.53%	1.60%	1.60%	1.60%	1.60%	1.85%	1.91%	1.98%	0.89%	0.89%	0.89%	0.89%	0.89%	0.89%	0.89%	0.89%	0.06%	0.89%	0.89%	0.89%		1	14	0	1	1	14	14	14	14	14	14	15	32	32	32	32	31	31	31	40	31	31	31	35	35	36	35	31	28	31	31	28	29	29	29	29	29	29	29	29	29	29	29	29	29	29	29	7	1	16	24	32	24	2	30
32. *S. minus* SAm	1.66%	1.66%	1.66%	1.66%	1.66%	1.66%	1.66%	1.66%	1.53%	1.60%	1.60%	1.66%	1.66%	1.66%	1.66%	1.91%	1.98%	1.91%	0.96%	0.96%	0.96%	0.96%	0.96%	0.96%	0.96%	0.96%	0.00%	0.96%	0.96%	0.96%	0.06%		15	1	0	0	15	15	15	15	15	15	16	33	33	33	33	30	30	30	39	30	30	30	34	34	35	34	30	27	30	30	27	30	30	30	30	30	30	30	30	30	30	30	30	30	30	30	6	0	17	25	31	25	1	29
33. *S. minus* SmPS	1.34%	1.34%	1.34%	1.21%	1.34%	1.34%	1.34%	1.34%	1.21%	1.28%	1.28%	1.34%	1.34%	1.34%	1.34%	2.04%	2.17%	1.53%	0.00%	0.00%	0.00%	0.00%	0.00%	0.00%	0.00%	0.00%	0.96%	0.00%	0.00%	0.00%	0.89%	0.96%		14	15	15	0	0	0	0	0	0	3	24	24	24	24	21	21	24	30	21	21	21	25	25	32	31	21	24	21	21	24	22	22	22	22	22	22	22	22	22	22	22	22	22	22	22	21	15	2	20	28	20	16	20
34. *S. minus* EU200	1.60%	1.60%	1.60%	1.60%	1.60%	1.60%	1.60%	1.60%	1.47%	1.53%	1.53%	1.60%	1.60%	1.60%	1.60%	1.85%	1.91%	1.98%	0.89%	0.89%	0.89%	0.89%	0.89%	0.89%	0.89%	0.89%	0.06%	0.89%	0.89%	0.89%	0.00%	0.06%	0.89%		1	1	14	14	14	14	14	14	15	32	32	32	32	31	31	31	40	31	31	31	35	35	36	35	31	28	31	31	28	29	29	29	29	29	29	29	29	29	29	29	29	29	29	29	7	1	16	24	32	24	2	30
35. *S. minus* Mdg2-2	1.66%	1.66%	1.66%	1.66%	1.66%	1.66%	1.66%	1.66%	1.53%	1.60%	1.60%	1.66%	1.66%	1.66%	1.66%	1.91%	1.98%	1.91%	0.96%	0.96%	0.96%	0.96%	0.96%	0.96%	0.96%	0.96%	0.00%	0.96%	0.96%	0.96%	0.06%	0.00%	0.96%	0.06%		0	15	15	15	15	15	15	16	33	33	33	33	30	30	30	39	30	30	30	34	34	35	34	30	27	30	30	27	30	30	30	30	30	30	30	30	30	30	30	30	30	30	30	6	0	17	25	31	25	1	29
36. *S. minus* Mdg4	1.66%	1.66%	1.66%	1.66%	1.66%	1.66%	1.66%	1.66%	1.53%	1.60%	1.60%	1.66%	1.66%	1.66%	1.66%	1.91%	1.98%	1.91%	0.96%	0.96%	0.96%	0.96%	0.96%	0.96%	0.96%	0.96%	0.00%	0.96%	0.96%	0.96%	0.06%	0.00%	0.96%	0.06%	0.00%		15	15	15	15	15	15	16	33	33	33	33	30	30	30	39	30	30	30	34	34	35	34	30	27	30	30	27	30	30	30	30	30	30	30	30	30	30	30	30	30	30	30	6	0	17	25	31	25	1	29
37. *S. minus* SKS1044	1.34%	1.34%	1.34%	1.21%	1.34%	1.34%	1.34%	1.34%	1.21%	1.28%	1.28%	1.34%	1.34%	1.34%	1.34%	2.04%	2.17%	1.53%	0.00%	0.00%	0.00%	0.00%	0.00%	0.00%	0.00%	0.00%	0.96%	0.00%	0.00%	0.00%	0.89%	0.96%	0.00%	0.89%	0.96%	0.96%		0	0	0	0	0	3	24	24	24	24	21	21	24	30	21	21	21	25	25	32	31	21	24	21	21	24	22	22	22	22	22	22	22	22	22	22	22	22	22	22	22	21	15	2	20	28	20	16	20
38. *S. minus* GuS1058	1.34%	1.34%	1.34%	1.21%	1.34%	1.34%	1.34%	1.34%	1.21%	1.28%	1.28%	1.34%	1.34%	1.34%	1.34%	2.04%	2.17%	1.53%	0.00%	0.00%	0.00%	0.00%	0.00%	0.00%	0.00%	0.00%	0.96%	0.00%	0.00%	0.00%	0.89%	0.96%	0.00%	0.89%	0.96%	0.96%	0.00%		0	0	0	0	3	24	24	24	24	21	21	24	30	21	21	21	25	25	32	31	21	24	21	21	24	22	22	22	22	22	22	22	22	22	22	22	22	22	22	22	21	15	2	20	28	20	16	20
39. *S. minus* SKS1073	1.34%	1.34%	1.34%	1.21%	1.34%	1.34%	1.34%	1.34%	1.21%	1.28%	1.28%	1.34%	1.34%	1.34%	1.34%	2.04%	2.17%	1.53%	0.00%	0.00%	0.00%	0.00%	0.00%	0.00%	0.00%	0.00%	0.96%	0.00%	0.00%	0.00%	0.89%	0.96%	0.00%	0.89%	0.96%	0.96%	0.00%	0.00%		0	0	0	3	24	24	24	24	21	21	24	30	21	21	21	25	25	32	31	21	24	21	21	24	22	22	22	22	22	22	22	22	22	22	22	22	22	22	22	21	15	2	20	28	20	16	20
40. *S. minus* PBG1	1.34%	1.34%	1.34%	1.21%	1.34%	1.34%	1.34%	1.34%	1.21%	1.28%	1.28%	1.34%	1.34%	1.34%	1.34%	2.04%	2.17%	1.53%	0.00%	0.00%	0.00%	0.00%	0.00%	0.00%	0.00%	0.00%	0.96%	0.00%	0.00%	0.00%	0.89%	0.96%	0.00%	0.89%	0.96%	0.96%	0.00%	0.00%	0.00%		0	0	3	24	24	24	24	21	21	24	30	21	21	21	25	25	32	31	21	24	21	21	24	22	22	22	22	22	22	22	22	22	22	22	22	22	22	22	21	15	2	20	28	20	16	20
41. *S. minus* SS5	1.34%	1.34%	1.34%	1.21%	1.34%	1.34%	1.34%	1.34%	1.21%	1.28%	1.28%	1.34%	1.34%	1.34%	1.34%	2.04%	2.17%	1.53%	0.00%	0.00%	0.00%	0.00%	0.00%	0.00%	0.00%	0.00%	0.96%	0.00%	0.00%	0.00%	0.89%	0.96%	0.00%	0.89%	0.96%	0.96%	0.00%	0.00%	0.00%	0.00%		0	3	24	24	24	24	21	21	24	30	21	21	21	25	25	32	31	21	24	21	21	24	22	22	22	22	22	22	22	22	22	22	22	22	22	22	22	21	15	2	20	28	20	16	20
42. *S. minus* SKS1018	1.34%	1.34%	1.34%	1.21%	1.34%	1.34%	1.34%	1.34%	1.21%	1.28%	1.28%	1.34%	1.34%	1.34%	1.34%	2.04%	2.17%	1.53%	0.00%	0.00%	0.00%	0.00%	0.00%	0.00%	0.00%	0.00%	0.96%	0.00%	0.00%	0.00%	0.89%	0.96%	0.00%	0.89%	0.96%	0.96%	0.00%	0.00%	0.00%	0.00%	0.00%		3	24	24	24	24	21	21	24	30	21	21	21	25	25	32	31	21	24	21	21	24	22	22	22	22	22	22	22	22	22	22	22	22	22	22	22	21	15	2	20	28	20	16	20
43. *S. semivirescens* DoEng	1.15%	1.15%	1.15%	1.02%	1.15%	1.15%	1.15%	1.15%	1.02%	1.08%	1.08%	1.15%	1.15%	1.15%	1.15%	2.11%	2.23%	1.60%	0.19%	0.19%	0.19%	0.19%	0.19%	0.19%	0.19%	0.19%	1.02%	0.19%	0.19%	0.19%	0.96%	1.02%	0.19%	0.96%	1.02%	1.02%	0.19%	0.19%	0.19%	0.19%	0.19%	0.19%		23	23	23	23	24	24	23	29	24	24	24	28	28	35	34	24	27	24	24	27	23	23	23	23	23	23	23	23	23	23	23	23	23	23	23	22	16	3	17	31	17	17	23
44. *S. subtilis* SKS1056	2.11%	2.11%	2.11%	1.98%	2.11%	2.11%	2.11%	2.11%	1.98%	2.04%	2.04%	2.11%	2.11%	2.11%	2.11%	3.06%	3.19%	2.17%	1.53%	1.53%	1.53%	1.53%	1.53%	1.53%	1.53%	1.53%	2.11%	1.53%	1.53%	1.53%	2.04%	2.11%	1.53%	2.04%	2.11%	2.11%	1.53%	1.53%	1.53%	1.53%	1.53%	1.53%	1.47%		0	0	0	37	37	34	44	37	37	37	37	37	45	44	37	39	37	37	39	34	34	34	34	34	34	34	34	34	34	34	34	34	34	34	37	33	26	32	41	32	34	36
45. *S. subtilis* SKS937	2.11%	2.11%	2.11%	1.98%	2.11%	2.11%	2.11%	2.11%	1.98%	2.04%	2.04%	2.11%	2.11%	2.11%	2.11%	3.06%	3.19%	2.17%	1.53%	1.53%	1.53%	1.53%	1.53%	1.53%	1.53%	1.53%	2.11%	1.53%	1.53%	1.53%	2.04%	2.11%	1.53%	2.04%	2.11%	2.11%	1.53%	1.53%	1.53%	1.53%	1.53%	1.53%	1.47%	0.00%		0	0	37	37	34	44	37	37	37	37	37	45	44	37	39	37	37	39	34	34	34	34	34	34	34	34	34	34	34	34	34	34	34	37	33	26	32	41	32	34	36
46. *S. subtilis* SS4-1	2.11%	2.11%	2.11%	1.98%	2.11%	2.11%	2.11%	2.11%	1.98%	2.04%	2.04%	2.11%	2.11%	2.11%	2.11%	3.06%	3.19%	2.17%	1.53%	1.53%	1.53%	1.53%	1.53%	1.53%	1.53%	1.53%	2.11%	1.53%	1.53%	1.53%	2.04%	2.11%	1.53%	2.04%	2.11%	2.11%	1.53%	1.53%	1.53%	1.53%	1.53%	1.53%	1.47%	0.00%	0.00%		0	37	37	34	44	37	37	37	37	37	45	44	37	39	37	37	39	34	34	34	34	34	34	34	34	34	34	34	34	34	34	34	37	33	26	32	41	32	34	36
47. *S. subtilis* Zur3	2.11%	2.11%	2.11%	1.98%	2.11%	2.11%	2.11%	2.11%	1.98%	2.04%	2.04%	2.11%	2.11%	2.11%	2.11%	3.06%	3.19%	2.17%	1.53%	1.53%	1.53%	1.53%	1.53%	1.53%	1.53%	1.53%	2.11%	1.53%	1.53%	1.53%	2.04%	2.11%	1.53%	2.04%	2.11%	2.11%	1.53%	1.53%	1.53%	1.53%	1.53%	1.53%	1.47%	0.00%	0.00%	0.00%		37	37	34	44	37	37	37	37	37	45	44	37	39	37	37	39	34	34	34	34	34	34	34	34	34	34	34	34	34	34	34	37	33	26	32	41	32	34	36
48. *S. teres* PFEU3Sm2	2.36%	2.36%	2.36%	2.23%	2.36%	2.36%	2.36%	2.36%	2.23%	2.30%	2.30%	2.36%	2.36%	2.36%	2.36%	1.40%	1.53%	0.70%	1.34%	1.34%	1.34%	1.34%	1.34%	1.34%	1.34%	1.34%	1.91%	1.34%	1.34%	1.34%	1.98%	1.91%	1.34%	1.98%	1.91%	1.91%	1.34%	1.34%	1.34%	1.34%	1.34%	1.34%	1.53%	2.36%	2.36%	2.36%	2.36%		0	13	17	0	0	0	4	4	13	12	0	8	0	0	8	11	11	11	11	11	11	11	11	11	11	11	11	11	11	11	32	30	21	36	9	36	31	1
49. *S. teres* NorKD	2.36%	2.36%	2.36%	2.23%	2.36%	2.36%	2.36%	2.36%	2.23%	2.30%	2.30%	2.36%	2.36%	2.36%	2.36%	1.40%	1.53%	0.70%	1.34%	1.34%	1.34%	1.34%	1.34%	1.34%	1.34%	1.34%	1.91%	1.34%	1.34%	1.34%	1.98%	1.91%	1.34%	1.98%	1.91%	1.91%	1.34%	1.34%	1.34%	1.34%	1.34%	1.34%	1.53%	2.36%	2.36%	2.36%	2.36%	0.00%		13	17	0	0	0	4	4	13	12	0	8	0	0	8	11	11	11	11	11	11	11	11	11	11	11	11	11	11	11	32	30	21	36	9	36	31	1
50. *S. teres* Mdg3	2.11%	2.11%	2.11%	1.98%	2.11%	2.11%	2.11%	2.11%	1.98%	2.04%	2.04%	2.11%	2.11%	2.11%	2.11%	1.60%	1.72%	0.26%	1.53%	1.53%	1.53%	1.53%	1.53%	1.53%	1.53%	1.53%	1.91%	1.53%	1.53%	1.53%	1.98%	1.91%	1.53%	1.98%	1.91%	1.91%	1.53%	1.53%	1.53%	1.53%	1.53%	1.53%	1.47%	2.17%	2.17%	2.17%	2.17%	0.83%	0.83%		16	13	13	13	13	13	24	23	13	17	13	13	17	6	6	6	6	6	6	6	6	6	6	6	6	6	6	6	34	30	26	32	17	32	31	12
51. *S. teres* PF1a	2.55%	2.55%	2.55%	2.43%	2.55%	2.55%	2.55%	2.55%	2.43%	2.49%	2.49%	2.55%	2.55%	2.55%	2.55%	2.04%	2.04%	1.28%	1.91%	1.91%	1.91%	1.91%	1.91%	1.91%	1.91%	1.91%	2.49%	1.91%	1.91%	1.91%	2.55%	2.49%	1.91%	2.55%	2.49%	2.49%	1.91%	1.91%	1.91%	1.91%	1.91%	1.91%	1.85%	2.81%	2.81%	2.81%	2.81%	1.08%	1.08%	1.02%		17	17	17	15	15	26	25	17	21	17	17	21	22	22	22	22	22	22	22	22	22	22	22	22	22	22	22	41	39	30	39	23	39	40	18
52. *S. teres* SKS187	2.36%	2.36%	2.36%	2.23%	2.36%	2.36%	2.36%	2.36%	2.23%	2.30%	2.30%	2.36%	2.36%	2.36%	2.36%	1.40%	1.53%	0.70%	1.34%	1.34%	1.34%	1.34%	1.34%	1.34%	1.34%	1.34%	1.91%	1.34%	1.34%	1.34%	1.98%	1.91%	1.34%	1.98%	1.91%	1.91%	1.34%	1.34%	1.34%	1.34%	1.34%	1.34%	1.53%	2.36%	2.36%	2.36%	2.36%	0.00%	0.00%	0.83%	1.08%		0	0	4	4	13	12	0	8	0	0	8	11	11	11	11	11	11	11	11	11	11	11	11	11	11	11	32	30	21	36	9	36	31	1
53. *S. teres* SKS107	2.36%	2.36%	2.36%	2.23%	2.36%	2.36%	2.36%	2.36%	2.23%	2.30%	2.30%	2.36%	2.36%	2.36%	2.36%	1.40%	1.53%	0.70%	1.34%	1.34%	1.34%	1.34%	1.34%	1.34%	1.34%	1.34%	1.91%	1.34%	1.34%	1.34%	1.98%	1.91%	1.34%	1.98%	1.91%	1.91%	1.34%	1.34%	1.34%	1.34%	1.34%	1.34%	1.53%	2.36%	2.36%	2.36%	2.36%	0.00%	0.00%	0.83%	1.08%	0.00%		0	4	4	13	12	0	8	0	0	8	11	11	11	11	11	11	11	11	11	11	11	11	11	11	11	32	30	21	36	9	36	31	1
54. *S. teres* SKS988	2.36%	2.36%	2.36%	2.23%	2.36%	2.36%	2.36%	2.36%	2.23%	2.30%	2.30%	2.36%	2.36%	2.36%	2.36%	1.40%	1.53%	0.70%	1.34%	1.34%	1.34%	1.34%	1.34%	1.34%	1.34%	1.34%	1.91%	1.34%	1.34%	1.34%	1.98%	1.91%	1.34%	1.98%	1.91%	1.91%	1.34%	1.34%	1.34%	1.34%	1.34%	1.34%	1.53%	2.36%	2.36%	2.36%	2.36%	0.00%	0.00%	0.83%	1.08%	0.00%	0.00%		4	4	13	12	0	8	0	0	8	11	11	11	11	11	11	11	11	11	11	11	11	11	11	11	32	30	21	36	9	36	31	1
55. *S. teres* SKS351	2.49%	2.49%	2.49%	2.36%	2.49%	2.49%	2.49%	2.49%	2.36%	2.43%	2.43%	2.49%	2.49%	2.49%	2.49%	1.66%	1.66%	0.70%	1.60%	1.60%	1.60%	1.60%	1.60%	1.60%	1.60%	1.60%	2.17%	1.60%	1.60%	1.60%	2.23%	2.17%	1.60%	2.23%	2.17%	2.17%	1.60%	1.60%	1.60%	1.60%	1.60%	1.60%	1.79%	2.36%	2.36%	2.36%	2.36%	0.26%	0.26%	0.83%	0.96%	0.26%	0.26%	0.26%		0	12	11	4	10	4	4	10	13	13	13	13	13	13	13	13	13	13	13	13	13	13	13	34	34	25	38	12	38	35	5
56. *S. teres* SKS026	2.49%	2.49%	2.49%	2.36%	2.49%	2.49%	2.49%	2.49%	2.36%	2.43%	2.43%	2.49%	2.49%	2.49%	2.49%	1.66%	1.66%	0.70%	1.60%	1.60%	1.60%	1.60%	1.60%	1.60%	1.60%	1.60%	2.17%	1.60%	1.60%	1.60%	2.23%	2.17%	1.60%	2.23%	2.17%	2.17%	1.60%	1.60%	1.60%	1.60%	1.60%	1.60%	1.79%	2.36%	2.36%	2.36%	2.36%	0.26%	0.26%	0.83%	0.96%	0.26%	0.26%	0.26%	0.00%		12	11	4	10	4	4	10	13	13	13	13	13	13	13	13	13	13	13	13	13	13	13	34	34	25	38	12	38	35	5
57. *S. teres* SPT	2.55%	2.55%	2.55%	2.55%	2.55%	2.55%	2.55%	2.55%	2.55%	2.49%	2.49%	2.55%	2.55%	2.55%	2.55%	1.47%	1.47%	1.28%	2.04%	2.04%	2.04%	2.04%	2.04%	2.04%	2.04%	2.04%	2.23%	2.04%	2.04%	2.04%	2.30%	2.23%	2.04%	2.30%	2.23%	2.23%	2.04%	2.04%	2.04%	2.04%	2.04%	2.04%	2.23%	2.87%	2.87%	2.87%	2.87%	0.83%	0.83%	1.53%	1.66%	0.83%	0.83%	0.83%	0.77%	0.77%		1	13	11	13	13	11	22	22	22	22	22	22	22	22	22	22	22	22	22	22	22	35	35	32	39	17	41	36	14
58. *S. teres* SKS1045	2.49%	2.49%	2.49%	2.49%	2.49%	2.49%	2.49%	2.49%	2.49%	2.43%	2.43%	2.49%	2.49%	2.49%	2.49%	1.40%	1.40%	1.21%	1.98%	1.98%	1.98%	1.98%	1.98%	1.98%	1.98%	1.98%	2.17%	1.98%	1.98%	1.98%	2.23%	2.17%	1.98%	2.23%	2.17%	2.17%	1.98%	1.98%	1.98%	1.98%	1.98%	1.98%	2.17%	2.81%	2.81%	2.81%	2.81%	0.77%	0.77%	1.47%	1.60%	0.77%	0.77%	0.77%	0.70%	0.70%	0.06%		12	10	12	12	10	21	21	21	21	21	21	21	21	21	21	21	21	21	21	21	34	34	31	38	16	40	35	13
59. *S. teres* SKS085	2.36%	2.36%	2.36%	2.23%	2.36%	2.36%	2.36%	2.36%	2.23%	2.30%	2.30%	2.36%	2.36%	2.36%	2.36%	1.40%	1.53%	0.70%	1.34%	1.34%	1.34%	1.34%	1.34%	1.34%	1.34%	1.34%	1.91%	1.34%	1.34%	1.34%	1.98%	1.91%	1.34%	1.98%	1.91%	1.91%	1.34%	1.34%	1.34%	1.34%	1.34%	1.34%	1.53%	2.36%	2.36%	2.36%	2.36%	0.00%	0.00%	0.83%	1.08%	0.00%	0.00%	0.00%	0.26%	0.26%	0.83%	0.77%		8	0	0	8	11	11	11	11	11	11	11	11	11	11	11	11	11	11	11	32	30	21	36	9	36	31	1
60. *S. teres* Mdg2-1	2.17%	2.17%	2.17%	2.17%	2.17%	2.17%	2.17%	2.17%	2.17%	2.11%	2.11%	2.17%	2.17%	2.17%	2.17%	1.40%	1.53%	0.83%	1.53%	1.53%	1.53%	1.53%	1.53%	1.53%	1.53%	1.53%	1.72%	1.53%	1.53%	1.53%	1.79%	1.72%	1.53%	1.79%	1.72%	1.72%	1.53%	1.53%	1.53%	1.53%	1.53%	1.53%	1.72%	2.49%	2.49%	2.49%	2.49%	0.51%	0.51%	1.08%	1.34%	0.51%	0.51%	0.51%	0.64%	0.64%	0.70%	0.64%	0.51%		8	8	0	15	15	15	15	15	15	15	15	15	15	15	15	15	15	15	29	27	26	33	10	35	28	9
61. *S. teres* EU199	2.36%	2.36%	2.36%	2.23%	2.36%	2.36%	2.36%	2.36%	2.23%	2.30%	2.30%	2.36%	2.36%	2.36%	2.36%	1.40%	1.53%	0.70%	1.34%	1.34%	1.34%	1.34%	1.34%	1.34%	1.34%	1.34%	1.91%	1.34%	1.34%	1.34%	1.98%	1.91%	1.34%	1.98%	1.91%	1.91%	1.34%	1.34%	1.34%	1.34%	1.34%	1.34%	1.53%	2.36%	2.36%	2.36%	2.36%	0.00%	0.00%	0.83%	1.08%	0.00%	0.00%	0.00%	0.26%	0.26%	0.83%	0.77%	0.00%	0.51%		0	8	11	11	11	11	11	11	11	11	11	11	11	11	11	11	11	32	30	21	36	9	36	31	1
62. *S. teres* NorBG	2.36%	2.36%	2.36%	2.23%	2.36%	2.36%	2.36%	2.36%	2.23%	2.30%	2.30%	2.36%	2.36%	2.36%	2.36%	1.40%	1.53%	0.70%	1.34%	1.34%	1.34%	1.34%	1.34%	1.34%	1.34%	1.34%	1.91%	1.34%	1.34%	1.34%	1.98%	1.91%	1.34%	1.98%	1.91%	1.91%	1.34%	1.34%	1.34%	1.34%	1.34%	1.34%	1.53%	2.36%	2.36%	2.36%	2.36%	0.00%	0.00%	0.83%	1.08%	0.00%	0.00%	0.00%	0.26%	0.26%	0.83%	0.77%	0.00%	0.51%	0.00%		8	11	11	11	11	11	11	11	11	11	11	11	11	11	11	11	32	30	21	36	9	36	31	1
63. *S. teres* Seef1	2.17%	2.17%	2.17%	2.17%	2.17%	2.17%	2.17%	2.17%	2.17%	2.11%	2.11%	2.17%	2.17%	2.17%	2.17%	1.40%	1.53%	0.83%	1.53%	1.53%	1.53%	1.53%	1.53%	1.53%	1.53%	1.53%	1.72%	1.53%	1.53%	1.53%	1.79%	1.72%	1.53%	1.79%	1.72%	1.72%	1.53%	1.53%	1.53%	1.53%	1.53%	1.53%	1.72%	2.49%	2.49%	2.49%	2.49%	0.51%	0.51%	1.08%	1.34%	0.51%	0.51%	0.51%	0.64%	0.64%	0.70%	0.64%	0.51%	0.00%	0.51%	0.51%		15	15	15	15	15	15	15	15	15	15	15	15	15	15	15	29	27	26	33	10	35	28	9
64. *S. yagiui* SKS165	2.23%	2.23%	2.23%	2.11%	2.23%	2.23%	2.23%	2.23%	2.11%	2.17%	2.17%	2.23%	2.23%	2.23%	2.23%	1.53%	1.66%	0.13%	1.40%	1.40%	1.40%	1.40%	1.40%	1.40%	1.40%	1.40%	1.91%	1.40%	1.40%	1.40%	1.85%	1.91%	1.40%	1.85%	1.91%	1.91%	1.40%	1.40%	1.40%	1.40%	1.40%	1.40%	1.47%	2.17%	2.17%	2.17%	2.17%	0.70%	0.70%	0.38%	1.40%	0.70%	0.70%	0.70%	0.83%	0.83%	1.40%	1.34%	0.70%	0.96%	0.70%	0.70%	0.96%		0	0	0	0	0	0	0	0	0	0	0	0	0	0	32	30	24	34	16	34	31	10
65. *S. yagiui* SKS315	2.23%	2.23%	2.23%	2.11%	2.23%	2.23%	2.23%	2.23%	2.11%	2.17%	2.17%	2.23%	2.23%	2.23%	2.23%	1.53%	1.66%	0.13%	1.40%	1.40%	1.40%	1.40%	1.40%	1.40%	1.40%	1.40%	1.91%	1.40%	1.40%	1.40%	1.85%	1.91%	1.40%	1.85%	1.91%	1.91%	1.40%	1.40%	1.40%	1.40%	1.40%	1.40%	1.47%	2.17%	2.17%	2.17%	2.17%	0.70%	0.70%	0.38%	1.40%	0.70%	0.70%	0.70%	0.83%	0.83%	1.40%	1.34%	0.70%	0.96%	0.70%	0.70%	0.96%	0.00%		0	0	0	0	0	0	0	0	0	0	0	0	0	32	30	24	34	16	34	31	10
66. *S. yagiui* SKS164	2.23%	2.23%	2.23%	2.11%	2.23%	2.23%	2.23%	2.23%	2.11%	2.17%	2.17%	2.23%	2.23%	2.23%	2.23%	1.53%	1.66%	0.13%	1.40%	1.40%	1.40%	1.40%	1.40%	1.40%	1.40%	1.40%	1.91%	1.40%	1.40%	1.40%	1.85%	1.91%	1.40%	1.85%	1.91%	1.91%	1.40%	1.40%	1.40%	1.40%	1.40%	1.40%	1.47%	2.17%	2.17%	2.17%	2.17%	0.70%	0.70%	0.38%	1.40%	0.70%	0.70%	0.70%	0.83%	0.83%	1.40%	1.34%	0.70%	0.96%	0.70%	0.70%	0.96%	0.00%	0.00%		0	0	0	0	0	0	0	0	0	0	0	0	32	30	24	34	16	34	31	10
67. *S. yagiui* SKS071	2.23%	2.23%	2.23%	2.11%	2.23%	2.23%	2.23%	2.23%	2.11%	2.17%	2.17%	2.23%	2.23%	2.23%	2.23%	1.53%	1.66%	0.13%	1.40%	1.40%	1.40%	1.40%	1.40%	1.40%	1.40%	1.40%	1.91%	1.40%	1.40%	1.40%	1.85%	1.91%	1.40%	1.85%	1.91%	1.91%	1.40%	1.40%	1.40%	1.40%	1.40%	1.40%	1.47%	2.17%	2.17%	2.17%	2.17%	0.70%	0.70%	0.38%	1.40%	0.70%	0.70%	0.70%	0.83%	0.83%	1.40%	1.34%	0.70%	0.96%	0.70%	0.70%	0.96%	0.00%	0.00%	0.00%		0	0	0	0	0	0	0	0	0	0	0	32	30	24	34	16	34	31	10
68. *S. yagiui* JpH1020	2.23%	2.23%	2.23%	2.11%	2.23%	2.23%	2.23%	2.23%	2.11%	2.17%	2.17%	2.23%	2.23%	2.23%	2.23%	1.53%	1.66%	0.13%	1.40%	1.40%	1.40%	1.40%	1.40%	1.40%	1.40%	1.40%	1.91%	1.40%	1.40%	1.40%	1.85%	1.91%	1.40%	1.85%	1.91%	1.91%	1.40%	1.40%	1.40%	1.40%	1.40%	1.40%	1.47%	2.17%	2.17%	2.17%	2.17%	0.70%	0.70%	0.38%	1.40%	0.70%	0.70%	0.70%	0.83%	0.83%	1.40%	1.34%	0.70%	0.96%	0.70%	0.70%	0.96%	0.00%	0.00%	0.00%	0.00%		0	0	0	0	0	0	0	0	0	0	32	30	24	34	16	34	31	10
69. *S. yagiui* SKS583	2.23%	2.23%	2.23%	2.11%	2.23%	2.23%	2.23%	2.23%	2.11%	2.17%	2.17%	2.23%	2.23%	2.23%	2.23%	1.53%	1.66%	0.13%	1.40%	1.40%	1.40%	1.40%	1.40%	1.40%	1.40%	1.40%	1.91%	1.40%	1.40%	1.40%	1.85%	1.91%	1.40%	1.85%	1.91%	1.91%	1.40%	1.40%	1.40%	1.40%	1.40%	1.40%	1.47%	2.17%	2.17%	2.17%	2.17%	0.70%	0.70%	0.38%	1.40%	0.70%	0.70%	0.70%	0.83%	0.83%	1.40%	1.34%	0.70%	0.96%	0.70%	0.70%	0.96%	0.00%	0.00%	0.00%	0.00%	0.00%		0	0	0	0	0	0	0	0	0	32	30	24	34	16	34	31	10
70. *S. yagiui* SKS977	2.23%	2.23%	2.23%	2.11%	2.23%	2.23%	2.23%	2.23%	2.11%	2.17%	2.17%	2.23%	2.23%	2.23%	2.23%	1.53%	1.66%	0.13%	1.40%	1.40%	1.40%	1.40%	1.40%	1.40%	1.40%	1.40%	1.91%	1.40%	1.40%	1.40%	1.85%	1.91%	1.40%	1.85%	1.91%	1.91%	1.40%	1.40%	1.40%	1.40%	1.40%	1.40%	1.47%	2.17%	2.17%	2.17%	2.17%	0.70%	0.70%	0.38%	1.40%	0.70%	0.70%	0.70%	0.83%	0.83%	1.40%	1.34%	0.70%	0.96%	0.70%	0.70%	0.96%	0.00%	0.00%	0.00%	0.00%	0.00%	0.00%		0	0	0	0	0	0	0	0	32	30	24	34	16	34	31	10
71. *S. yagiui* SKS983	2.23%	2.23%	2.23%	2.11%	2.23%	2.23%	2.23%	2.23%	2.11%	2.17%	2.17%	2.23%	2.23%	2.23%	2.23%	1.53%	1.66%	0.13%	1.40%	1.40%	1.40%	1.40%	1.40%	1.40%	1.40%	1.40%	1.91%	1.40%	1.40%	1.40%	1.85%	1.91%	1.40%	1.85%	1.91%	1.91%	1.40%	1.40%	1.40%	1.40%	1.40%	1.40%	1.47%	2.17%	2.17%	2.17%	2.17%	0.70%	0.70%	0.38%	1.40%	0.70%	0.70%	0.70%	0.83%	0.83%	1.40%	1.34%	0.70%	0.96%	0.70%	0.70%	0.96%	0.00%	0.00%	0.00%	0.00%	0.00%	0.00%	0.00%		0	0	0	0	0	0	0	32	30	24	34	16	34	31	10
72. *S. yagiui* SKS964	2.23%	2.23%	2.23%	2.11%	2.23%	2.23%	2.23%	2.23%	2.11%	2.17%	2.17%	2.23%	2.23%	2.23%	2.23%	1.53%	1.66%	0.13%	1.40%	1.40%	1.40%	1.40%	1.40%	1.40%	1.40%	1.40%	1.91%	1.40%	1.40%	1.40%	1.85%	1.91%	1.40%	1.85%	1.91%	1.91%	1.40%	1.40%	1.40%	1.40%	1.40%	1.40%	1.47%	2.17%	2.17%	2.17%	2.17%	0.70%	0.70%	0.38%	1.40%	0.70%	0.70%	0.70%	0.83%	0.83%	1.40%	1.34%	0.70%	0.96%	0.70%	0.70%	0.96%	0.00%	0.00%	0.00%	0.00%	0.00%	0.00%	0.00%	0.00%		0	0	0	0	0	0	32	30	24	34	16	34	31	10
73. *S. yagiui* SKS787	2.23%	2.23%	2.23%	2.11%	2.23%	2.23%	2.23%	2.23%	2.11%	2.17%	2.17%	2.23%	2.23%	2.23%	2.23%	1.53%	1.66%	0.13%	1.40%	1.40%	1.40%	1.40%	1.40%	1.40%	1.40%	1.40%	1.91%	1.40%	1.40%	1.40%	1.85%	1.91%	1.40%	1.85%	1.91%	1.91%	1.40%	1.40%	1.40%	1.40%	1.40%	1.40%	1.47%	2.17%	2.17%	2.17%	2.17%	0.70%	0.70%	0.38%	1.40%	0.70%	0.70%	0.70%	0.83%	0.83%	1.40%	1.34%	0.70%	0.96%	0.70%	0.70%	0.96%	0.00%	0.00%	0.00%	0.00%	0.00%	0.00%	0.00%	0.00%	0.00%		0	0	0	0	0	32	30	24	34	16	34	31	10
74. *S. yagiui* SKS963	2.23%	2.23%	2.23%	2.11%	2.23%	2.23%	2.23%	2.23%	2.11%	2.17%	2.17%	2.23%	2.23%	2.23%	2.23%	1.53%	1.66%	0.13%	1.40%	1.40%	1.40%	1.40%	1.40%	1.40%	1.40%	1.40%	1.91%	1.40%	1.40%	1.40%	1.85%	1.91%	1.40%	1.85%	1.91%	1.91%	1.40%	1.40%	1.40%	1.40%	1.40%	1.40%	1.47%	2.17%	2.17%	2.17%	2.17%	0.70%	0.70%	0.38%	1.40%	0.70%	0.70%	0.70%	0.83%	0.83%	1.40%	1.34%	0.70%	0.96%	0.70%	0.70%	0.96%	0.00%	0.00%	0.00%	0.00%	0.00%	0.00%	0.00%	0.00%	0.00%	0.00%		0	0	0	0	32	30	24	34	16	34	31	10
75. *S. yagiui* Pozz	2.23%	2.23%	2.23%	2.11%	2.23%	2.23%	2.23%	2.23%	2.11%	2.17%	2.17%	2.23%	2.23%	2.23%	2.23%	1.53%	1.66%	0.13%	1.40%	1.40%	1.40%	1.40%	1.40%	1.40%	1.40%	1.40%	1.91%	1.40%	1.40%	1.40%	1.85%	1.91%	1.40%	1.85%	1.91%	1.91%	1.40%	1.40%	1.40%	1.40%	1.40%	1.40%	1.47%	2.17%	2.17%	2.17%	2.17%	0.70%	0.70%	0.38%	1.40%	0.70%	0.70%	0.70%	0.83%	0.83%	1.40%	1.34%	0.70%	0.96%	0.70%	0.70%	0.96%	0.00%	0.00%	0.00%	0.00%	0.00%	0.00%	0.00%	0.00%	0.00%	0.00%	0.00%		0	0	0	32	30	24	34	16	34	31	10
76. *S. yagiui* LarnCyp	2.23%	2.23%	2.23%	2.11%	2.23%	2.23%	2.23%	2.23%	2.11%	2.17%	2.17%	2.23%	2.23%	2.23%	2.23%	1.53%	1.66%	0.13%	1.40%	1.40%	1.40%	1.40%	1.40%	1.40%	1.40%	1.40%	1.91%	1.40%	1.40%	1.40%	1.85%	1.91%	1.40%	1.85%	1.91%	1.91%	1.40%	1.40%	1.40%	1.40%	1.40%	1.40%	1.47%	2.17%	2.17%	2.17%	2.17%	0.70%	0.70%	0.38%	1.40%	0.70%	0.70%	0.70%	0.83%	0.83%	1.40%	1.34%	0.70%	0.96%	0.70%	0.70%	0.96%	0.00%	0.00%	0.00%	0.00%	0.00%	0.00%	0.00%	0.00%	0.00%	0.00%	0.00%	0.00%		0	0	32	30	24	34	16	34	31	10
77. *S. yagiui* SpirWS	2.23%	2.23%	2.23%	2.11%	2.23%	2.23%	2.23%	2.23%	2.11%	2.17%	2.17%	2.23%	2.23%	2.23%	2.23%	1.53%	1.66%	0.13%	1.40%	1.40%	1.40%	1.40%	1.40%	1.40%	1.40%	1.40%	1.91%	1.40%	1.40%	1.40%	1.85%	1.91%	1.40%	1.85%	1.91%	1.91%	1.40%	1.40%	1.40%	1.40%	1.40%	1.40%	1.47%	2.17%	2.17%	2.17%	2.17%	0.70%	0.70%	0.38%	1.40%	0.70%	0.70%	0.70%	0.83%	0.83%	1.40%	1.34%	0.70%	0.96%	0.70%	0.70%	0.96%	0.00%	0.00%	0.00%	0.00%	0.00%	0.00%	0.00%	0.00%	0.00%	0.00%	0.00%	0.00%	0.00%		0	32	30	24	34	16	34	31	10
78. *S. yagiui* GNS4	2.23%	2.23%	2.23%	2.11%	2.23%	2.23%	2.23%	2.23%	2.11%	2.17%	2.17%	2.23%	2.23%	2.23%	2.23%	1.53%	1.66%	0.13%	1.40%	1.40%	1.40%	1.40%	1.40%	1.40%	1.40%	1.40%	1.91%	1.40%	1.40%	1.40%	1.85%	1.91%	1.40%	1.85%	1.91%	1.91%	1.40%	1.40%	1.40%	1.40%	1.40%	1.40%	1.47%	2.17%	2.17%	2.17%	2.17%	0.70%	0.70%	0.38%	1.40%	0.70%	0.70%	0.70%	0.83%	0.83%	1.40%	1.34%	0.70%	0.96%	0.70%	0.70%	0.96%	0.00%	0.00%	0.00%	0.00%	0.00%	0.00%	0.00%	0.00%	0.00%	0.00%	0.00%	0.00%	0.00%	0.00%		32	30	24	34	16	34	31	10
79. *S.* sp. Ind	2.04%	2.04%	2.04%	2.04%	2.04%	2.04%	2.04%	2.04%	1.91%	1.98%	1.98%	2.04%	2.04%	2.04%	2.04%	2.17%	2.11%	2.04%	1.34%	1.34%	1.34%	1.34%	1.34%	1.34%	1.34%	1.34%	0.38%	1.34%	1.34%	1.34%	0.45%	0.38%	1.34%	0.45%	0.38%	0.38%	1.34%	1.34%	1.34%	1.34%	1.34%	1.34%	1.40%	2.36%	2.36%	2.36%	2.36%	2.04%	2.04%	2.17%	2.62%	2.04%	2.04%	2.04%	2.17%	2.17%	2.23%	2.17%	2.04%	1.85%	2.04%	2.04%	1.85%	2.04%	2.04%	2.04%	2.04%	2.04%	2.04%	2.04%	2.04%	2.04%	2.04%	2.04%	2.04%	2.04%	2.04%	2.04%		6	23	31	33	31	5	31
80. *S.* sp. Thd2	1.66%	1.66%	1.66%	1.66%	1.66%	1.66%	1.66%	1.66%	1.53%	1.60%	1.60%	1.66%	1.66%	1.66%	1.66%	1.91%	1.98%	1.91%	0.96%	0.96%	0.96%	0.96%	0.96%	0.96%	0.96%	0.96%	0.00%	0.96%	0.96%	0.96%	0.06%	0.00%	0.96%	0.06%	0.00%	0.00%	0.96%	0.96%	0.96%	0.96%	0.96%	0.96%	1.02%	2.11%	2.11%	2.11%	2.11%	1.91%	1.91%	1.91%	2.49%	1.91%	1.91%	1.91%	2.17%	2.17%	2.23%	2.17%	1.91%	1.72%	1.91%	1.91%	1.72%	1.91%	1.91%	1.91%	1.91%	1.91%	1.91%	1.91%	1.91%	1.91%	1.91%	1.91%	1.91%	1.91%	1.91%	1.91%	0.38%		17	25	31	25	1	29
81. *S.* sp. SKS255	1.34%	1.34%	1.34%	1.21%	1.34%	1.34%	1.34%	1.34%	1.21%	1.28%	1.28%	1.34%	1.34%	1.34%	1.34%	2.04%	2.17%	1.66%	0.13%	0.13%	0.13%	0.13%	0.13%	0.13%	0.13%	0.13%	1.08%	0.13%	0.13%	0.13%	1.02%	1.08%	0.13%	1.02%	1.08%	1.08%	0.13%	0.13%	0.13%	0.13%	0.13%	0.13%	0.19%	1.66%	1.66%	1.66%	1.66%	1.34%	1.34%	1.66%	1.91%	1.34%	1.34%	1.34%	1.60%	1.60%	2.04%	1.98%	1.34%	1.66%	1.34%	1.34%	1.66%	1.53%	1.53%	1.53%	1.53%	1.53%	1.53%	1.53%	1.53%	1.53%	1.53%	1.53%	1.53%	1.53%	1.53%	1.53%	1.47%	1.08%		20	30	20	18	20
82. *S.* sp. Seef2	0.06%	0.19%	0.19%	0.06%	0.06%	0.06%	0.06%	0.06%	0.26%	0.00%	0.00%	0.06%	0.06%	0.06%	0.19%	2.49%	2.62%	2.17%	1.28%	1.28%	1.28%	1.28%	1.28%	1.28%	1.28%	1.28%	1.60%	1.28%	1.28%	1.28%	1.53%	1.60%	1.28%	1.53%	1.60%	1.60%	1.28%	1.28%	1.28%	1.28%	1.28%	1.28%	1.08%	2.04%	2.04%	2.04%	2.04%	2.30%	2.30%	2.04%	2.49%	2.30%	2.30%	2.30%	2.43%	2.43%	2.49%	2.43%	2.30%	2.11%	2.30%	2.30%	2.11%	2.17%	2.17%	2.17%	2.17%	2.17%	2.17%	2.17%	2.17%	2.17%	2.17%	2.17%	2.17%	2.17%	2.17%	2.17%	1.98%	1.60%	1.28%		39	5	26	35
83. *S.* sp. StMgN	2.55%	2.55%	2.55%	2.55%	2.55%	2.55%	2.55%	2.55%	2.43%	2.49%	2.49%	2.55%	2.55%	2.55%	2.55%	1.47%	1.60%	0.96%	1.79%	1.79%	1.79%	1.79%	1.79%	1.79%	1.79%	1.79%	1.98%	1.79%	1.79%	1.79%	2.04%	1.98%	1.79%	2.04%	1.98%	1.98%	1.79%	1.79%	1.79%	1.79%	1.79%	1.79%	1.98%	2.62%	2.62%	2.62%	2.62%	0.57%	0.57%	1.08%	1.47%	0.57%	0.57%	0.57%	0.77%	0.77%	1.08%	1.02%	0.57%	0.64%	0.57%	0.57%	0.64%	1.02%	1.02%	1.02%	1.02%	1.02%	1.02%	1.02%	1.02%	1.02%	1.02%	1.02%	1.02%	1.02%	1.02%	1.02%	2.11%	1.98%	1.91%	2.49%		39	32	10
84. *S.* sp. GuaCHN	0.38%	0.26%	0.26%	0.38%	0.38%	0.38%	0.38%	0.38%	0.06%	0.32%	0.32%	0.38%	0.38%	0.38%	0.26%	2.49%	2.62%	2.17%	1.28%	1.28%	1.28%	1.28%	1.28%	1.28%	1.28%	1.28%	1.60%	1.28%	1.28%	1.28%	1.53%	1.60%	1.28%	1.53%	1.60%	1.60%	1.28%	1.28%	1.28%	1.28%	1.28%	1.28%	1.08%	2.04%	2.04%	2.04%	2.04%	2.30%	2.30%	2.04%	2.49%	2.30%	2.30%	2.30%	2.43%	2.43%	2.62%	2.55%	2.30%	2.23%	2.30%	2.30%	2.23%	2.17%	2.17%	2.17%	2.17%	2.17%	2.17%	2.17%	2.17%	2.17%	2.17%	2.17%	2.17%	2.17%	2.17%	2.17%	1.98%	1.60%	1.28%	0.32%	2.49%		26	35
85. *S.* sp. SmJFp1	1.72%	1.72%	1.72%	1.72%	1.72%	1.72%	1.72%	1.72%	1.60%	1.66%	1.66%	1.72%	1.72%	1.72%	1.72%	1.98%	2.04%	1.98%	1.02%	1.02%	1.02%	1.02%	1.02%	1.02%	1.02%	1.02%	0.06%	1.02%	1.02%	1.02%	0.13%	0.06%	1.02%	0.13%	0.06%	0.06%	1.02%	1.02%	1.02%	1.02%	1.02%	1.02%	1.08%	2.17%	2.17%	2.17%	2.17%	1.98%	1.98%	1.98%	2.55%	1.98%	1.98%	1.98%	2.23%	2.23%	2.30%	2.23%	1.98%	1.79%	1.98%	1.98%	1.79%	1.98%	1.98%	1.98%	1.98%	1.98%	1.98%	1.98%	1.98%	1.98%	1.98%	1.98%	1.98%	1.98%	1.98%	1.98%	0.32%	0.06%	1.15%	1.66%	2.04%	1.66%		30
86. *S.* sp. StJFp2	2.30%	2.30%	2.30%	2.17%	2.30%	2.30%	2.30%	2.30%	2.17%	2.23%	2.23%	2.30%	2.30%	2.30%	2.30%	1.34%	1.47%	0.64%	1.28%	1.28%	1.28%	1.28%	1.28%	1.28%	1.28%	1.28%	1.85%	1.28%	1.28%	1.28%	1.91%	1.85%	1.28%	1.91%	1.85%	1.85%	1.28%	1.28%	1.28%	1.28%	1.28%	1.28%	1.47%	2.30%	2.30%	2.30%	2.30%	0.06%	0.06%	0.77%	1.15%	0.06%	0.06%	0.06%	0.32%	0.32%	0.89%	0.83%	0.06%	0.57%	0.06%	0.06%	0.57%	0.64%	0.64%	0.64%	0.64%	0.64%	0.64%	0.64%	0.64%	0.64%	0.64%	0.64%	0.64%	0.64%	0.64%	0.64%	1.98%	1.85%	1.28%	2.23%	0.64%	2.23%	1.91%	

Supplementary Table S4. Numbers of nucleotide differences (above diagonal) and pairwise distances (below diagonal) of the ITS regions among 86 *Spirostomum* populations.

Taxa	1	2	3	4	5	6	7	8	9	10	11	12	13	14	15	16	17	18	19	20	21	22	23	24	25	26	27	28	29	30	31	32	33	34	35	36	37	38	39	40	41	42	43	44	45	46	47	48	49	50	51	52	53	54	55	56	57	58	59	60	61	62	63	64	65	66	67	68	69	70	71	72	73	74	75	76	77	78	79	80	81	82	83	84	85	86
1. *S. ambiguum* SKS363		0	0	0	0	0	0	0	1	0	0	0	0	0	0	27	28	25	16	16	17	16	16	16	15	15	23	16	15	16	23	23	16	23	22	22	16	14	16	16	13	16	15	5	5	5	5	23	23	29	23	23	23	23	21	21	28	28	22	23	23	23	23	30	30	30	30	30	30	30	30	30	30	30	30	30	30	30	2	0	23	15	23	26	23	22
2. *S. ambiguum* SKS254	0.00%		0	0	0	0	0	0	1	0	0	0	0	0	0	27	28	25	16	16	17	16	16	16	15	15	23	16	15	16	23	23	16	23	22	22	16	14	16	16	13	16	15	5	5	5	5	23	23	29	23	23	23	23	21	21	28	28	22	23	23	23	23	30	30	30	30	30	30	30	30	30	30	30	30	30	30	30	2	0	23	15	23	26	23	22
3. *S. ambiguum* SKS204	0.00%	0.00%		0	0	0	0	0	1	0	0	0	0	0	0	27	28	25	16	16	17	16	16	16	15	15	23	16	15	16	23	23	16	23	22	22	16	14	16	16	13	16	15	5	5	5	5	23	23	29	23	23	23	23	21	21	28	28	22	23	23	23	23	30	30	30	30	30	30	30	30	30	30	30	30	30	30	30	2	0	23	15	23	26	23	22
4. *S. ambiguum* SKS779	0.00%	0.00%	0.00%		0	0	0	0	1	0	0	0	0	0	0	27	28	25	16	16	17	16	16	16	15	15	23	16	15	16	23	23	16	23	22	22	16	14	16	16	13	16	15	5	5	5	5	23	23	29	23	23	23	23	21	21	28	28	22	23	23	23	23	30	30	30	30	30	30	30	30	30	30	30	30	30	30	30	2	0	23	15	23	26	23	22
5. *S. ambiguum* SKS989	0.00%	0.00%	0.00%	0.00%		0	0	0	1	0	0	0	0	0	0	27	28	25	16	16	17	16	16	16	15	15	23	16	15	16	23	23	16	23	22	22	16	14	16	16	13	16	15	5	5	5	5	23	23	29	23	23	23	23	21	21	28	28	22	23	23	23	23	30	30	30	30	30	30	30	30	30	30	30	30	30	30	30	2	0	23	15	23	26	23	22
6. *S. ambiguum* SKS949	0.00%	0.00%	0.00%	0.00%	0.00%		0	0	1	0	0	0	0	0	0	27	28	25	16	16	17	16	16	16	15	15	23	16	15	16	23	23	16	23	22	22	16	14	16	16	13	16	15	5	5	5	5	23	23	29	23	23	23	23	21	21	28	28	22	23	23	23	23	30	30	30	30	30	30	30	30	30	30	30	30	30	30	30	2	0	23	15	23	26	23	22
7. *S. ambiguum* SKS947	0.00%	0.00%	0.00%	0.00%	0.00%	0.00%		0	1	0	0	0	0	0	0	27	28	25	16	16	17	16	16	16	15	15	23	16	15	16	23	23	16	23	22	22	16	14	16	16	13	16	15	5	5	5	5	23	23	29	23	23	23	23	21	21	28	28	22	23	23	23	23	30	30	30	30	30	30	30	30	30	30	30	30	30	30	30	2	0	23	15	23	26	23	22
8. *S. ambiguum* SKS185	0.00%	0.00%	0.00%	0.00%	0.00%	0.00%	0.00%		1	0	0	0	0	0	0	27	28	25	16	16	17	16	16	16	15	15	23	16	15	16	23	23	16	23	22	22	16	14	16	16	13	16	15	5	5	5	5	23	23	29	23	23	23	23	21	21	28	28	22	23	23	23	23	30	30	30	30	30	30	30	30	30	30	30	30	30	30	30	2	0	23	15	23	26	23	22
9. *S. ambiguum* SA	0.39%	0.39%	0.39%	0.39%	0.39%	0.39%	0.39%	0.39%		1	1	1	1	1	1	28	29	25	16	16	17	16	16	16	15	15	23	16	15	16	23	23	16	23	22	22	16	14	16	16	13	16	15	4	4	4	4	23	23	29	23	23	23	23	21	21	28	28	22	23	23	23	23	30	30	30	30	30	30	30	30	30	30	30	30	30	30	30	3	1	23	15	23	26	23	22
10. *S. ambiguum* SS4-2	0.00%	0.00%	0.00%	0.00%	0.00%	0.00%	0.00%	0.00%	0.39%		0	0	0	0	0	28	29	25	16	16	17	16	16	16	15	15	23	16	15	16	23	23	16	23	22	22	16	14	16	16	13	16	15	5	5	5	5	23	23	29	23	23	23	23	21	21	28	28	22	23	23	23	23	30	30	30	30	30	30	30	30	30	30	30	30	30	30	30	2	0	23	15	23	26	23	22
11. *S. ambiguum* EU201	0.00%	0.00%	0.00%	0.00%	0.00%	0.00%	0.00%	0.00%	0.39%	0.00%		0	0	0	0	28	29	25	16	16	17	16	16	16	15	15	23	16	15	16	23	23	16	23	22	22	16	14	16	16	13	16	15	5	5	5	5	23	23	29	23	23	23	23	21	21	28	28	22	23	23	23	23	30	30	30	30	30	30	30	30	30	30	30	30	30	30	30	2	0	23	15	23	26	23	22
12. *S. ambiguum* SKS1050	0.00%	0.00%	0.00%	0.00%	0.00%	0.00%	0.00%	0.00%	0.39%	0.00%	0.00%		0	0	0	27	28	25	16	16	17	16	16	16	15	15	23	16	15	16	23	23	16	23	22	22	16	14	16	16	13	16	15	5	5	5	5	23	23	29	23	23	23	23	21	21	28	28	22	23	23	23	23	30	30	30	30	30	30	30	30	30	30	30	30	30	30	30	2	0	23	15	23	26	23	22
13. *S. ambiguum* SKS184	0.00%	0.00%	0.00%	0.00%	0.00%	0.00%	0.00%	0.00%	0.39%	0.00%	0.00%	0.00%		0	0	27	28	25	16	16	17	16	16	16	15	15	23	16	15	16	23	23	16	23	22	22	16	14	16	16	13	16	15	5	5	5	5	23	23	29	23	23	23	23	21	21	28	28	22	23	23	23	23	30	30	30	30	30	30	30	30	30	30	30	30	30	30	30	2	0	23	15	23	26	23	22
14. *S. ambiguum* SKS169	0.00%	0.00%	0.00%	0.00%	0.00%	0.00%	0.00%	0.00%	0.39%	0.00%	0.00%	0.00%	0.00%		0	27	28	25	16	16	17	16	16	16	15	15	23	16	15	16	23	23	16	23	22	22	16	14	16	16	13	16	15	5	5	5	5	23	23	29	23	23	23	23	21	21	28	28	22	23	23	23	23	30	30	30	30	30	30	30	30	30	30	30	30	30	30	30	2	0	23	15	23	26	23	22
15. *S. ambiguum* SKS167	0.00%	0.00%	0.00%	0.00%	0.00%	0.00%	0.00%	0.00%	0.39%	0.00%	0.00%	0.00%	0.00%	0.00%		27	28	25	16	16	17	16	16	16	15	15	23	16	15	16	23	23	16	23	22	22	16	14	16	16	13	16	15	5	5	5	5	23	23	29	23	23	23	23	21	21	28	28	22	23	23	23	23	30	30	30	30	30	30	30	30	30	30	30	30	30	30	30	2	0	23	15	23	26	23	22
16. *S. caudatum* SKS813	10.76%	10.76%	10.76%	10.76%	10.76%	10.76%	10.76%	10.76%	11.11%	11.11%	11.11%	10.76%	10.76%	10.76%	10.76%		4	23	36	36	35	36	36	36	32	35	32	36	35	36	33	33	37	33	34	34	36	34	37	37	34	36	35	28	28	29	29	20	20	25	24	19	19	19	18	18	25	23	19	21	20	20	21	22	22	22	22	22	22	22	22	22	22	22	23	23	23	23	27	27	32	35	18	21	31	31
17. *S. caudatum* SKS302	11.11%	11.11%	11.11%	11.11%	11.11%	11.11%	11.11%	11.11%	11.46%	11.46%	11.46%	11.11%	11.11%	11.11%	11.11%	1.58%		24	35	35	34	35	35	35	31	34	33	35	34	35	34	34	36	34	35	35	35	33	36	36	33	35	34	29	29	30	30	21	21	26	25	20	20	20	19	19	24	23	20	23	21	21	23	24	24	24	24	24	24	24	24	24	24	24	25	25	25	25	28	28	33	35	19	22	31	32
18. *S. dharwarensis* SAd	9.96%	9.96%	9.96%	9.96%	9.96%	9.96%	9.96%	9.96%	9.92%	9.92%	9.92%	9.96%	9.96%	9.96%	9.96%	9.27%	9.68%		30	30	30	30	30	30	27	29	30	30	29	30	30	30	30	30	30	30	30	28	30	30	27	30	31	26	26	26	26	13	13	14	9	13	13	13	12	12	25	24	13	17	13	13	17	8	8	8	8	8	8	8	8	8	8	8	8	8	8	8	25	25	30	30	14	19	30	29
19. *S. minus* SKS215	6.30%	6.30%	6.30%	6.30%	6.30%	6.30%	6.30%	6.30%	6.30%	6.30%	6.30%	6.30%	6.30%	6.30%	6.30%	14.40%	14.00%	11.95%		0	1	0	0	0	1	1	16	0	1	0	16	16	0	16	15	15	0	2	0	0	0	0	2	15	15	15	15	30	30	34	29	30	30	30	28	28	33	32	29	30	30	30	30	34	34	34	34	34	34	34	34	34	34	34	34	34	34	34	14	16	16	2	30	32	18	17
20. *S. minus* SKS386	6.30%	6.30%	6.30%	6.30%	6.30%	6.30%	6.30%	6.30%	6.30%	6.30%	6.30%	6.30%	6.30%	6.30%	6.30%	14.40%	14.00%	11.95%	0.00%		1	0	0	0	1	1	16	0	1	0	16	16	0	16	15	15	0	2	0	0	0	0	2	15	15	15	15	30	30	34	29	30	30	30	28	28	33	32	29	30	30	30	30	34	34	34	34	34	34	34	34	34	34	34	34	34	34	34	14	16	16	2	30	32	18	17
21. *S. minus* SKS731	6.69%	6.69%	6.69%	6.69%	6.69%	6.69%	6.69%	6.69%	6.69%	6.69%	6.69%	6.69%	6.69%	6.69%	6.69%	14.00%	13.60%	11.95%	0.39%	0.39%		1	1	1	2	2	15	1	2	1	15	15	1	15	16	16	1	3	1	1	1	1	3	16	16	16	16	30	30	33	30	30	30	30	28	28	32	31	29	29	30	30	29	33	33	33	33	33	33	33	33	33	33	33	33	33	33	33	15	17	15	3	29	31	17	16
22. *S. minus* SKS161	6.30%	6.30%	6.30%	6.30%	6.30%	6.30%	6.30%	6.30%	6.30%	6.30%	6.30%	6.30%	6.30%	6.30%	6.30%	14.40%	14.00%	11.95%	0.00%	0.00%	0.39%		0	0	1	1	16	0	1	0	16	16	0	16	15	15	0	2	0	0	0	0	2	15	15	15	15	30	30	34	29	30	30	30	28	28	33	32	29	30	30	30	30	34	34	34	34	34	34	34	34	34	34	34	34	34	34	34	14	16	16	2	30	32	18	17
23. *S. minus* SKS182	6.30%	6.30%	6.30%	6.30%	6.30%	6.30%	6.30%	6.30%	6.30%	6.30%	6.30%	6.30%	6.30%	6.30%	6.30%	14.40%	14.00%	11.95%	0.00%	0.00%	0.39%	0.00%		0	1	1	16	0	1	0	16	16	0	16	15	15	0	2	0	0	0	0	2	15	15	15	15	30	30	34	29	30	30	30	28	28	33	32	29	30	30	30	30	34	34	34	34	34	34	34	34	34	34	34	34	34	34	34	14	16	16	2	30	32	18	17
24. *S. minus* SKS214	6.30%	6.30%	6.30%	6.30%	6.30%	6.30%	6.30%	6.30%	6.30%	6.30%	6.30%	6.30%	6.30%	6.30%	6.30%	14.40%	14.00%	11.95%	0.00%	0.00%	0.39%	0.00%	0.00%		1	1	16	0	1	0	16	16	0	16	15	15	0	2	0	0	0	0	2	15	15	15	15	30	30	34	29	30	30	30	28	28	33	32	29	30	30	30	30	34	34	34	34	34	34	34	34	34	34	34	34	34	34	34	14	16	16	2	30	32	18	17
25. *S. minus* SKS939	6.22%	6.22%	6.22%	6.22%	6.22%	6.22%	6.22%	6.22%	6.22%	6.22%	6.22%	6.22%	6.22%	6.22%	6.22%	13.50%	13.08%	11.34%	0.41%	0.41%	0.83%	0.41%	0.41%	0.41%		0	15	1	0	1	15	15	1	15	14	14	1	1	1	1	0	1	3	14	14	14	14	28	28	32	27	28	28	28	26	26	29	29	28	27	28	28	27	31	31	31	31	31	31	31	31	31	31	31	31	31	31	31	13	15	15	3	28	28	17	16
26. *S. minus* SKS940	5.91%	5.91%	5.91%	5.91%	5.91%	5.91%	5.91%	5.91%	5.91%	5.91%	5.91%	5.91%	5.91%	5.91%	5.91%	14.00%	13.60%	11.55%	0.39%	0.39%	0.79%	0.39%	0.39%	0.39%	0.00%		17	1	0	1	17	17	1	17	16	16	1	1	1	1	0	1	3	14	14	14	14	29	29	33	28	29	29	29	27	27	32	31	28	29	29	29	29	33	33	33	33	33	33	33	33	33	33	33	33	33	33	33	13	15	17	3	29	31	19	18
27. *S. minus* SKS991	8.98%	8.98%	8.98%	8.98%	8.98%	8.98%	8.98%	8.98%	8.98%	8.98%	8.98%	8.98%	8.98%	8.98%	8.98%	12.70%	13.04%	11.95%	6.30%	6.30%	5.91%	6.30%	6.30%	6.30%	6.22%	6.69%		16	17	16	0	0	16	0	1	1	16	18	16	16	15	16	15	23	23	23	23	25	25	27	28	25	25	25	24	24	26	26	24	22	25	25	22	32	32	32	32	32	32	32	32	32	32	32	32	32	32	32	21	23	0	18	24	24	4	2
28. *S. minus* SKS743	6.30%	6.30%	6.30%	6.30%	6.30%	6.30%	6.30%	6.30%	6.30%	6.30%	6.30%	6.30%	6.30%	6.30%	6.30%	14.40%	14.00%	11.95%	0.00%	0.00%	0.39%	0.00%	0.00%	0.00%	0.41%	0.39%	6.30%		1	0	16	16	0	16	15	15	0	2	0	0	0	0	2	15	15	15	15	30	30	34	29	30	30	30	28	28	33	32	29	30	30	30	30	34	34	34	34	34	34	34	34	34	34	34	34	34	34	34	14	16	16	2	30	32	18	17
29. *S. minus* SKS794	5.91%	5.91%	5.91%	5.91%	5.91%	5.91%	5.91%	5.91%	5.91%	5.91%	5.91%	5.91%	5.91%	5.91%	5.91%	14.00%	13.60%	11.55%	0.39%	0.39%	0.79%	0.39%	0.39%	0.39%	0.00%	0.00%	6.69%	0.39%		1	17	17	1	17	16	16	1	1	1	1	0	1	3	14	14	14	14	29	29	33	28	29	29	29	27	27	32	31	28	29	29	29	29	33	33	33	33	33	33	33	33	33	33	33	33	33	33	33	13	15	17	3	29	31	19	18
30. *S. minus* SKS840	6.30%	6.30%	6.30%	6.30%	6.30%	6.30%	6.30%	6.30%	6.30%	6.30%	6.30%	6.30%	6.30%	6.30%	6.30%	14.40%	14.00%	11.95%	0.00%	0.00%	0.39%	0.00%	0.00%	0.00%	0.41%	0.39%	6.30%	0.00%	0.39%		16	16	0	16	15	15	0	2	0	0	0	0	2	15	15	15	15	30	30	34	29	30	30	30	28	28	33	32	29	30	30	30	30	34	34	34	34	34	34	34	34	34	34	34	34	34	34	34	14	16	16	2	30	32	18	17
31. *S. minus* Gmn	8.98%	8.98%	8.98%	8.98%	8.98%	8.98%	8.98%	8.98%	8.95%	8.95%	8.95%	8.98%	8.98%	8.98%	8.98%	13.04%	13.39%	11.90%	6.30%	6.30%	5.91%	6.30%	6.30%	6.30%	6.22%	6.69%	0.00%	6.30%	6.69%	6.30%		0	16	0	1	1	16	18	16	16	15	16	15	23	23	23	23	25	25	27	28	25	25	25	24	24	26	26	24	22	25	25	22	32	32	32	32	32	32	32	32	32	32	32	32	32	32	32	21	23	0	18	24	24	4	2
32. *S. minus* SAm	8.98%	8.98%	8.98%	8.98%	8.98%	8.98%	8.98%	8.98%	8.95%	8.95%	8.95%	8.98%	8.98%	8.98%	8.98%	13.04%	13.39%	11.90%	6.30%	6.30%	5.91%	6.30%	6.30%	6.30%	6.22%	6.69%	0.00%	6.30%	6.69%	6.30%	0.00%		16	0	1	1	16	18	16	16	15	16	15	23	23	23	23	25	25	27	28	25	25	25	24	24	26	26	24	22	25	25	22	32	32	32	32	32	32	32	32	32	32	32	32	32	32	32	21	23	0	18	24	24	4	2
33. *S. minus* SmPS	6.30%	6.30%	6.30%	6.30%	6.30%	6.30%	6.30%	6.30%	6.27%	6.27%	6.27%	6.30%	6.30%	6.30%	6.30%	14.74%	14.34%	11.90%	0.00%	0.00%	0.39%	0.00%	0.00%	0.00%	0.41%	0.39%	6.30%	0.00%	0.39%	0.00%	6.27%	6.27%		16	15	15	0	2	0	0	0	0	2	15	15	15	15	30	30	34	29	30	30	30	28	28	33	32	29	30	30	30	30	34	34	34	34	34	34	34	34	34	34	34	34	34	34	34	14	16	16	2	30	32	18	17
34. *S. minus* EU200	8.98%	8.98%	8.98%	8.98%	8.98%	8.98%	8.98%	8.98%	8.95%	8.95%	8.95%	8.98%	8.98%	8.98%	8.98%	13.04%	13.39%	11.90%	6.30%	6.30%	5.91%	6.30%	6.30%	6.30%	6.22%	6.69%	0.00%	6.30%	6.69%	6.30%	0.00%	0.00%	6.27%		1	1	16	18	16	16	15	16	15	23	23	23	23	25	25	27	28	25	25	25	24	24	26	26	24	22	25	25	22	32	32	32	32	32	32	32	32	32	32	32	32	32	32	32	21	23	0	18	24	24	4	2
35. *S. minus* Mdg2-2	8.59%	8.59%	8.59%	8.59%	8.59%	8.59%	8.59%	8.59%	8.56%	8.56%	8.56%	8.59%	8.59%	8.59%	8.59%	13.44%	13.78%	11.90%	5.91%	5.91%	6.30%	5.91%	5.91%	5.91%	5.81%	6.30%	0.39%	5.91%	6.30%	5.91%	0.39%	0.39%	5.88%	0.39%		0	15	17	15	15	14	15	14	22	22	22	22	25	25	28	27	25	25	25	24	24	27	27	24	23	25	25	23	33	33	33	33	33	33	33	33	33	33	33	33	33	33	33	20	22	1	17	25	25	5	3
36. *S. minus* Mdg4	8.59%	8.59%	8.59%	8.59%	8.59%	8.59%	8.59%	8.59%	8.56%	8.56%	8.56%	8.59%	8.59%	8.59%	8.59%	13.44%	13.78%	11.90%	5.91%	5.91%	6.30%	5.91%	5.91%	5.91%	5.81%	6.30%	0.39%	5.91%	6.30%	5.91%	0.39%	0.39%	5.88%	0.39%	0.00%		15	17	15	15	14	15	14	22	22	22	22	25	25	28	27	25	25	25	24	24	27	27	24	23	25	25	23	33	33	33	33	33	33	33	33	33	33	33	33	33	33	33	20	22	1	17	25	25	5	3
37. *S. minus* SKS1044	6.30%	6.30%	6.30%	6.30%	6.30%	6.30%	6.30%	6.30%	6.30%	6.30%	6.30%	6.30%	6.30%	6.30%	6.30%	14.40%	14.00%	11.95%	0.00%	0.00%	0.39%	0.00%	0.00%	0.00%	0.41%	0.39%	6.30%	0.00%	0.39%	0.00%	6.30%	6.30%	0.00%	6.30%	5.91%	5.91%		2	0	0	0	0	2	15	15	15	15	30	30	34	29	30	30	30	28	28	33	32	29	30	30	30	30	34	34	34	34	34	34	34	34	34	34	34	34	34	34	34	14	16	16	2	30	32	18	17
38. *S. minus* GuS1058	5.51%	5.51%	5.51%	5.51%	5.51%	5.51%	5.51%	5.51%	5.51%	5.51%	5.51%	5.51%	5.51%	5.51%	5.51%	13.60%	13.20%	11.16%	0.79%	0.79%	1.18%	0.79%	0.79%	0.79%	0.41%	0.39%	7.09%	0.79%	0.39%	0.79%	7.09%	7.09%	0.79%	7.09%	6.69%	6.69%	0.79%		2	2	0	2	4	13	13	13	13	28	28	32	27	28	28	28	26	26	32	31	27	29	28	28	29	32	32	32	32	32	32	32	32	32	32	32	32	32	32	32	12	14	18	4	28	31	20	19
39. *S. minus* SKS1073	6.30%	6.30%	6.30%	6.30%	6.30%	6.30%	6.30%	6.30%	6.27%	6.27%	6.27%	6.30%	6.30%	6.30%	6.30%	14.74%	14.34%	11.90%	0.00%	0.00%	0.39%	0.00%	0.00%	0.00%	0.41%	0.39%	6.30%	0.00%	0.39%	0.00%	6.27%	6.27%	0.00%	6.27%	5.88%	5.88%	0.00%	0.79%		0	0	0	2	15	15	15	15	30	30	34	29	30	30	30	28	28	33	32	29	30	30	30	30	34	34	34	34	34	34	34	34	34	34	34	34	34	34	34	14	16	16	2	30	32	18	17
40. *S. minus* PBG1	6.30%	6.30%	6.30%	6.30%	6.30%	6.30%	6.30%	6.30%	6.27%	6.27%	6.27%	6.30%	6.30%	6.30%	6.30%	14.74%	14.34%	11.90%	0.00%	0.00%	0.39%	0.00%	0.00%	0.00%	0.41%	0.39%	6.30%	0.00%	0.39%	0.00%	6.27%	6.27%	0.00%	6.27%	5.88%	5.88%	0.00%	0.79%	0.00%		0	0	2	15	15	15	15	30	30	34	29	30	30	30	28	28	33	32	29	30	30	30	30	34	34	34	34	34	34	34	34	34	34	34	34	34	34	34	14	16	16	2	30	32	18	17
41. *S. minus* SS5	5.18%	5.18%	5.18%	5.18%	5.18%	5.18%	5.18%	5.18%	5.16%	5.16%	5.16%	5.18%	5.18%	5.18%	5.18%	13.71%	13.31%	10.84%	0.00%	0.00%	0.40%	0.00%	0.00%	0.00%	0.00%	0.00%	5.98%	0.00%	0.00%	0.00%	5.95%	5.95%	0.00%	5.95%	5.56%	5.56%	0.00%	0.00%	0.00%	0.00%		0	1	12	12	12	12	27	27	31	26	27	27	27	25	25	30	29	26	27	27	27	27	31	31	31	31	31	31	31	31	31	31	31	31	31	31	31	11	13	15	2	27	29	17	16
42. *S. minus* SKS1018	6.30%	6.30%	6.30%	6.30%	6.30%	6.30%	6.30%	6.30%	6.30%	6.30%	6.30%	6.30%	6.30%	6.30%	6.30%	14.40%	14.00%	11.95%	0.00%	0.00%	0.39%	0.00%	0.00%	0.00%	0.41%	0.39%	6.30%	0.00%	0.39%	0.00%	6.30%	6.30%	0.00%	6.30%	5.91%	5.91%	0.00%	0.79%	0.00%	0.00%	0.00%		2	15	15	15	15	30	30	34	29	30	30	30	28	28	33	32	29	30	30	30	30	34	34	34	34	34	34	34	34	34	34	34	34	34	34	34	14	16	16	2	30	32	18	17
43. *S. semivirescens* DoEng	5.91%	5.91%	5.91%	5.91%	5.91%	5.91%	5.91%	5.91%	5.91%	5.91%	5.91%	5.91%	5.91%	5.91%	5.91%	14.00%	13.60%	12.35%	0.79%	0.79%	1.18%	0.79%	0.79%	0.79%	1.24%	1.18%	5.91%	0.79%	1.18%	0.79%	5.91%	5.91%	0.79%	5.91%	5.51%	5.51%	0.79%	1.57%	0.79%	0.79%	0.40%	0.79%		13	13	13	13	29	29	33	30	29	29	29	27	27	32	31	28	29	29	29	29	35	35	35	35	35	35	35	35	35	35	35	35	35	35	35	13	15	15	2	29	31	17	16
44. *S. subtilis* SKS1056	1.95%	1.95%	1.95%	1.95%	1.95%	1.95%	1.95%	1.95%	1.56%	1.95%	1.95%	1.95%	1.95%	1.95%	1.95%	11.16%	11.51%	10.36%	5.91%	5.91%	6.30%	5.91%	5.91%	5.91%	5.81%	5.51%	8.98%	5.91%	5.51%	5.91%	8.98%	8.98%	5.91%	8.98%	8.59%	8.59%	5.91%	5.12%	5.91%	5.91%	4.78%	5.91%	5.12%		0	0	0	24	24	30	25	24	24	24	22	22	29	29	23	25	24	24	25	31	31	31	31	31	31	31	31	31	31	31	31	31	31	31	3	5	23	14	24	26	23	22
45. *S. subtilis* SKS937	1.95%	1.95%	1.95%	1.95%	1.95%	1.95%	1.95%	1.95%	1.56%	1.95%	1.95%	1.95%	1.95%	1.95%	1.95%	11.16%	11.51%	10.36%	5.91%	5.91%	6.30%	5.91%	5.91%	5.91%	5.81%	5.51%	8.98%	5.91%	5.51%	5.91%	8.98%	8.98%	5.91%	8.98%	8.59%	8.59%	5.91%	5.12%	5.91%	5.91%	4.78%	5.91%	5.12%	0.00%		0	0	24	24	30	25	24	24	24	22	22	29	29	23	25	24	24	25	31	31	31	31	31	31	31	31	31	31	31	31	31	31	31	3	5	23	14	24	26	23	22
46. *S. subtilis* SS4-1	1.95%	1.95%	1.95%	1.95%	1.95%	1.95%	1.95%	1.95%	1.55%	1.94%	1.94%	1.95%	1.95%	1.95%	1.95%	11.51%	11.86%	10.32%	5.91%	5.91%	6.30%	5.91%	5.91%	5.91%	5.81%	5.51%	8.98%	5.91%	5.51%	5.91%	8.95%	8.95%	5.88%	8.95%	8.56%	8.56%	5.91%	5.12%	5.88%	5.88%	4.76%	5.91%	5.12%	0.00%	0.00%		0	24	24	30	25	24	24	24	22	22	29	29	23	25	24	24	25	31	31	31	31	31	31	31	31	31	31	31	31	31	31	31	3	5	23	14	24	26	23	22
47. *S. subtilis* Zur3	1.95%	1.95%	1.95%	1.95%	1.95%	1.95%	1.95%	1.95%	1.55%	1.94%	1.94%	1.95%	1.95%	1.95%	1.95%	11.51%	11.86%	10.32%	5.91%	5.91%	6.30%	5.91%	5.91%	5.91%	5.81%	5.51%	8.98%	5.91%	5.51%	5.91%	8.95%	8.95%	5.88%	8.95%	8.56%	8.56%	5.91%	5.12%	5.88%	5.88%	4.76%	5.91%	5.12%	0.00%	0.00%	0.00%		24	24	30	25	24	24	24	22	22	29	29	23	25	24	24	25	31	31	31	31	31	31	31	31	31	31	31	31	31	31	31	3	5	23	14	24	26	23	22
48. *S. teres* PFEU3Sm2	9.16%	9.16%	9.16%	9.16%	9.16%	9.16%	9.16%	9.16%	9.13%	9.13%	9.13%	9.16%	9.16%	9.16%	9.16%	8.06%	8.47%	5.18%	11.95%	11.95%	11.95%	11.95%	11.95%	11.95%	11.76%	11.55%	9.96%	11.95%	11.55%	11.95%	9.92%	9.92%	11.90%	9.92%	9.92%	9.92%	11.95%	11.16%	11.90%	11.90%	10.84%	11.95%	11.55%	9.56%	9.56%	9.52%	9.52%		0	12	12	0	0	0	3	3	15	14	0	12	0	0	12	16	16	16	16	16	16	16	16	16	16	16	16	16	16	16	23	23	25	28	1	16	24	24
49. *S. teres* NorKD	9.16%	9.16%	9.16%	9.16%	9.16%	9.16%	9.16%	9.16%	9.13%	9.13%	9.13%	9.16%	9.16%	9.16%	9.16%	8.06%	8.47%	5.18%	11.95%	11.95%	11.95%	11.95%	11.95%	11.95%	11.76%	11.55%	9.96%	11.95%	11.55%	11.95%	9.92%	9.92%	11.90%	9.92%	9.92%	9.92%	11.95%	11.16%	11.90%	11.90%	10.84%	11.95%	11.55%	9.56%	9.56%	9.52%	9.52%	0.00%		12	12	0	0	0	3	3	15	14	0	12	0	0	12	16	16	16	16	16	16	16	16	16	16	16	16	16	16	16	23	23	25	28	1	16	24	24
50. *S. teres* Mdg3	11.46%	11.46%	11.46%	11.46%	11.46%	11.46%	11.46%	11.46%	11.42%	11.42%	11.42%	11.46%	11.46%	11.46%	11.46%	10.00%	10.40%	5.56%	13.44%	13.44%	13.04%	13.44%	13.44%	13.44%	13.33%	13.04%	10.67%	13.44%	13.04%	13.44%	10.63%	10.63%	13.39%	10.63%	11.02%	11.02%	13.44%	12.65%	13.39%	13.39%	12.35%	13.44%	13.04%	11.86%	11.86%	11.81%	11.81%	4.76%	4.76%		10	12	12	12	13	13	15	14	12	14	12	12	14	7	7	7	7	7	7	7	7	7	7	7	7	7	7	7	29	29	27	32	11	20	26	26
51. *S. teres* PF1a	9.20%	9.20%	9.20%	9.20%	9.20%	9.20%	9.20%	9.20%	9.16%	9.16%	9.16%	9.20%	9.20%	9.20%	9.20%	9.68%	10.08%	3.60%	11.60%	11.60%	12.00%	11.60%	11.60%	11.60%	11.39%	11.20%	11.20%	11.60%	11.20%	11.60%	11.16%	11.16%	11.55%	11.16%	10.76%	10.76%	11.60%	10.80%	11.55%	11.55%	10.48%	11.60%	12.00%	10.00%	10.00%	9.96%	9.96%	4.78%	4.78%	3.98%		12	12	12	13	13	21	20	12	15	12	12	15	11	11	11	11	11	11	11	11	11	11	11	11	11	11	11	24	23	28	29	12	16	27	27
52. *S. teres* SKS187	9.16%	9.16%	9.16%	9.16%	9.16%	9.16%	9.16%	9.16%	9.16%	9.16%	9.16%	9.16%	9.16%	9.16%	9.16%	7.69%	8.10%	5.20%	11.95%	11.95%	11.95%	11.95%	11.95%	11.95%	11.76%	11.55%	9.96%	11.95%	11.55%	11.95%	9.96%	9.96%	11.95%	9.96%	9.96%	9.96%	11.95%	11.16%	11.95%	11.95%	10.89%	11.95%	11.55%	9.56%	9.56%	9.56%	9.56%	0.00%	0.00%	4.78%	4.80%		0	0	3	3	15	14	0	12	0	0	12	16	16	16	16	16	16	16	16	16	16	16	16	16	16	16	23	23	25	28	1	16	24	24
53. *S. teres* SKS107	9.16%	9.16%	9.16%	9.16%	9.16%	9.16%	9.16%	9.16%	9.16%	9.16%	9.16%	9.16%	9.16%	9.16%	9.16%	7.69%	8.10%	5.20%	11.95%	11.95%	11.95%	11.95%	11.95%	11.95%	11.76%	11.55%	9.96%	11.95%	11.55%	11.95%	9.96%	9.96%	11.95%	9.96%	9.96%	9.96%	11.95%	11.16%	11.95%	11.95%	10.89%	11.95%	11.55%	9.56%	9.56%	9.56%	9.56%	0.00%	0.00%	4.78%	4.80%	0.00%		0	3	3	15	14	0	12	0	0	12	16	16	16	16	16	16	16	16	16	16	16	16	16	16	16	23	23	25	28	1	16	24	24
54. *S. teres* SKS988	9.16%	9.16%	9.16%	9.16%	9.16%	9.16%	9.16%	9.16%	9.16%	9.16%	9.16%	9.16%	9.16%	9.16%	9.16%	7.69%	8.10%	5.20%	11.95%	11.95%	11.95%	11.95%	11.95%	11.95%	11.76%	11.55%	9.96%	11.95%	11.55%	11.95%	9.96%	9.96%	11.95%	9.96%	9.96%	9.96%	11.95%	11.16%	11.95%	11.95%	10.89%	11.95%	11.55%	9.56%	9.56%	9.56%	9.56%	0.00%	0.00%	4.78%	4.80%	0.00%	0.00%		3	3	15	14	0	12	0	0	12	16	16	16	16	16	16	16	16	16	16	16	16	16	16	16	23	23	25	28	1	16	24	24
55. *S. teres* SKS351	8.37%	8.37%	8.37%	8.37%	8.37%	8.37%	8.37%	8.37%	8.37%	8.37%	8.37%	8.37%	8.37%	8.37%	8.37%	7.29%	7.69%	4.80%	11.16%	11.16%	11.16%	11.16%	11.16%	11.16%	10.92%	10.76%	9.56%	11.16%	10.76%	11.16%	9.56%	9.56%	11.16%	9.56%	9.56%	9.56%	11.16%	10.36%	11.16%	11.16%	10.08%	11.16%	10.76%	8.76%	8.76%	8.76%	8.76%	1.20%	1.20%	5.18%	5.20%	1.20%	1.20%	1.20%		0	16	15	3	13	3	3	13	15	15	15	15	15	15	15	15	15	15	15	15	15	15	15	21	21	24	26	4	17	24	24
56. *S. teres* SKS026	8.37%	8.37%	8.37%	8.37%	8.37%	8.37%	8.37%	8.37%	8.37%	8.37%	8.37%	8.37%	8.37%	8.37%	8.37%	7.29%	7.69%	4.80%	11.16%	11.16%	11.16%	11.16%	11.16%	11.16%	10.92%	10.76%	9.56%	11.16%	10.76%	11.16%	9.56%	9.56%	11.16%	9.56%	9.56%	9.56%	11.16%	10.36%	11.16%	11.16%	10.08%	11.16%	10.76%	8.76%	8.76%	8.76%	8.76%	1.20%	1.20%	5.18%	5.20%	1.20%	1.20%	1.20%	0.00%		16	15	3	13	3	3	13	15	15	15	15	15	15	15	15	15	15	15	15	15	15	15	21	21	24	26	4	17	24	24
57. *S. teres* SPT	11.02%	11.02%	11.02%	11.02%	11.02%	11.02%	11.02%	11.02%	10.98%	10.98%	10.98%	11.02%	11.02%	11.02%	11.02%	9.96%	9.52%	9.92%	13.10%	13.10%	12.70%	13.10%	13.10%	13.10%	12.13%	12.70%	10.20%	13.10%	12.70%	13.10%	10.16%	10.16%	13.04%	10.16%	10.55%	10.55%	13.10%	12.70%	13.04%	13.04%	12.00%	13.10%	12.70%	11.42%	11.42%	11.37%	11.37%	5.95%	5.95%	5.93%	8.37%	5.98%	5.98%	5.98%	6.37%	6.37%		2	15	14	15	15	14	19	19	19	19	19	19	19	19	19	19	19	19	19	19	19	28	28	26	32	14	10	25	24
58. *S. teres* SKS1045	11.02%	11.02%	11.02%	11.02%	11.02%	11.02%	11.02%	11.02%	11.02%	11.02%	11.02%	11.02%	11.02%	11.02%	11.02%	9.20%	9.16%	9.56%	12.70%	12.70%	12.30%	12.70%	12.70%	12.70%	12.13%	12.30%	10.20%	12.70%	12.30%	12.70%	10.20%	10.20%	12.70%	10.20%	10.59%	10.59%	12.70%	12.30%	12.70%	12.70%	11.65%	12.70%	12.30%	11.42%	11.42%	11.42%	11.42%	5.58%	5.58%	5.56%	8.00%	5.58%	5.58%	5.58%	5.98%	5.98%	0.78%		14	13	14	14	13	18	18	18	18	18	18	18	18	18	18	18	18	18	18	18	28	28	26	31	13	9	25	24
59. *S. teres* SKS085	8.80%	8.80%	8.80%	8.80%	8.80%	8.80%	8.80%	8.80%	8.80%	8.80%	8.80%	8.80%	8.80%	8.80%	8.80%	7.72%	8.13%	5.22%	11.60%	11.60%	11.60%	11.60%	11.60%	11.60%	11.76%	11.20%	9.60%	11.60%	11.20%	11.60%	9.60%	9.60%	11.60%	9.60%	9.60%	9.60%	11.60%	10.80%	11.60%	11.60%	10.53%	11.60%	11.20%	9.20%	9.20%	9.20%	9.20%	0.00%	0.00%	4.80%	4.82%	0.00%	0.00%	0.00%	1.20%	1.20%	6.00%	5.60%		11	0	0	11	16	16	16	16	16	16	16	16	16	16	16	16	16	16	16	22	22	24	27	1	16	23	23
60. *S. teres* Mdg2-1	9.16%	9.16%	9.16%	9.16%	9.16%	9.16%	9.16%	9.16%	9.13%	9.13%	9.13%	9.16%	9.16%	9.16%	9.16%	8.43%	9.24%	6.77%	11.95%	11.95%	11.55%	11.95%	11.95%	11.95%	11.34%	11.55%	8.73%	11.95%	11.55%	11.95%	8.70%	8.70%	11.90%	8.70%	9.09%	9.09%	11.95%	11.55%	11.90%	11.90%	10.84%	11.95%	11.55%	9.96%	9.96%	9.92%	9.92%	4.76%	4.76%	5.56%	5.98%	4.78%	4.78%	4.78%	5.18%	5.18%	5.53%	5.16%	4.40%		12	12	0	17	17	17	17	17	17	17	17	17	17	17	17	17	17	17	24	23	22	28	11	14	21	21
61. *S. teres* EU199	9.16%	9.16%	9.16%	9.16%	9.16%	9.16%	9.16%	9.16%	9.13%	9.13%	9.13%	9.16%	9.16%	9.16%	9.16%	8.06%	8.47%	5.18%	11.95%	11.95%	11.95%	11.95%	11.95%	11.95%	11.76%	11.55%	9.96%	11.95%	11.55%	11.95%	9.92%	9.92%	11.90%	9.92%	9.92%	9.92%	11.95%	11.16%	11.90%	11.90%	10.84%	11.95%	11.55%	9.56%	9.56%	9.52%	9.52%	0.00%	0.00%	4.76%	4.78%	0.00%	0.00%	0.00%	1.20%	1.20%	5.95%	5.58%	0.00%	4.76%		0	12	16	16	16	16	16	16	16	16	16	16	16	16	16	16	16	23	23	25	28	1	16	24	24
62. *S. teres* NorBG	9.16%	9.16%	9.16%	9.16%	9.16%	9.16%	9.16%	9.16%	9.13%	9.13%	9.13%	9.16%	9.16%	9.16%	9.16%	8.06%	8.47%	5.18%	11.95%	11.95%	11.95%	11.95%	11.95%	11.95%	11.76%	11.55%	9.96%	11.95%	11.55%	11.95%	9.92%	9.92%	11.90%	9.92%	9.92%	9.92%	11.95%	11.16%	11.90%	11.90%	10.84%	11.95%	11.55%	9.56%	9.56%	9.52%	9.52%	0.00%	0.00%	4.76%	4.78%	0.00%	0.00%	0.00%	1.20%	1.20%	5.95%	5.58%	0.00%	4.76%	0.00%		12	16	16	16	16	16	16	16	16	16	16	16	16	16	16	16	23	23	25	28	1	16	24	24
63. *S. teres* Seef1	9.16%	9.16%	9.16%	9.16%	9.16%	9.16%	9.16%	9.16%	9.13%	9.13%	9.13%	9.16%	9.16%	9.16%	9.16%	8.43%	9.24%	6.77%	11.95%	11.95%	11.55%	11.95%	11.95%	11.95%	11.34%	11.55%	8.73%	11.95%	11.55%	11.95%	8.70%	8.70%	11.90%	8.70%	9.09%	9.09%	11.95%	11.55%	11.90%	11.90%	10.84%	11.95%	11.55%	9.96%	9.96%	9.92%	9.92%	4.76%	4.76%	5.56%	5.98%	4.78%	4.78%	4.78%	5.18%	5.18%	5.53%	5.16%	4.40%	0.00%	4.76%	4.76%		17	17	17	17	17	17	17	17	17	17	17	17	17	17	17	24	23	22	28	11	14	21	21
64. *S. yagiui* SKS165	11.90%	11.90%	11.90%	11.90%	11.90%	11.90%	11.90%	11.90%	11.90%	11.90%	11.90%	11.90%	11.90%	11.90%	11.90%	8.84%	9.64%	3.20%	13.55%	13.55%	13.15%	13.55%	13.55%	13.55%	13.03%	13.15%	12.65%	13.55%	13.15%	13.55%	12.65%	12.65%	13.55%	12.65%	13.04%	13.04%	13.55%	12.75%	13.55%	13.55%	12.50%	13.55%	13.94%	12.30%	12.30%	12.30%	12.30%	6.37%	6.37%	2.79%	4.40%	6.37%	6.37%	6.37%	5.98%	5.98%	7.51%	7.11%	6.40%	6.75%	6.37%	6.37%	6.75%		0	0	0	0	0	0	0	0	0	0	0	0	0	0	30	30	32	34	15	19	30	30
65. *S. yagiui* SKS315	11.90%	11.90%	11.90%	11.90%	11.90%	11.90%	11.90%	11.90%	11.90%	11.90%	11.90%	11.90%	11.90%	11.90%	11.90%	8.84%	9.64%	3.20%	13.55%	13.55%	13.15%	13.55%	13.55%	13.55%	13.03%	13.15%	12.65%	13.55%	13.15%	13.55%	12.65%	12.65%	13.55%	12.65%	13.04%	13.04%	13.55%	12.75%	13.55%	13.55%	12.50%	13.55%	13.94%	12.30%	12.30%	12.30%	12.30%	6.37%	6.37%	2.79%	4.40%	6.37%	6.37%	6.37%	5.98%	5.98%	7.51%	7.11%	6.40%	6.75%	6.37%	6.37%	6.75%	0.00%		0	0	0	0	0	0	0	0	0	0	0	0	0	30	30	32	34	15	19	30	30
66. *S. yagiui* SKS164	11.90%	11.90%	11.90%	11.90%	11.90%	11.90%	11.90%	11.90%	11.90%	11.90%	11.90%	11.90%	11.90%	11.90%	11.90%	8.84%	9.64%	3.20%	13.55%	13.55%	13.15%	13.55%	13.55%	13.55%	13.03%	13.15%	12.65%	13.55%	13.15%	13.55%	12.65%	12.65%	13.55%	12.65%	13.04%	13.04%	13.55%	12.75%	13.55%	13.55%	12.50%	13.55%	13.94%	12.30%	12.30%	12.30%	12.30%	6.37%	6.37%	2.79%	4.40%	6.37%	6.37%	6.37%	5.98%	5.98%	7.51%	7.11%	6.40%	6.75%	6.37%	6.37%	6.75%	0.00%	0.00%		0	0	0	0	0	0	0	0	0	0	0	0	30	30	32	34	15	19	30	30
67. *S. yagiui* SKS071	11.90%	11.90%	11.90%	11.90%	11.90%	11.90%	11.90%	11.90%	11.90%	11.90%	11.90%	11.90%	11.90%	11.90%	11.90%	8.84%	9.64%	3.20%	13.55%	13.55%	13.15%	13.55%	13.55%	13.55%	13.03%	13.15%	12.65%	13.55%	13.15%	13.55%	12.65%	12.65%	13.55%	12.65%	13.04%	13.04%	13.55%	12.75%	13.55%	13.55%	12.50%	13.55%	13.94%	12.30%	12.30%	12.30%	12.30%	6.37%	6.37%	2.79%	4.40%	6.37%	6.37%	6.37%	5.98%	5.98%	7.51%	7.11%	6.40%	6.75%	6.37%	6.37%	6.75%	0.00%	0.00%	0.00%		0	0	0	0	0	0	0	0	0	0	0	30	30	32	34	15	19	30	30
68. *S. yagiui* JpH1020	11.90%	11.90%	11.90%	11.90%	11.90%	11.90%	11.90%	11.90%	11.90%	11.90%	11.90%	11.90%	11.90%	11.90%	11.90%	8.84%	9.64%	3.20%	13.55%	13.55%	13.15%	13.55%	13.55%	13.55%	13.03%	13.15%	12.65%	13.55%	13.15%	13.55%	12.65%	12.65%	13.55%	12.65%	13.04%	13.04%	13.55%	12.75%	13.55%	13.55%	12.50%	13.55%	13.94%	12.30%	12.30%	12.30%	12.30%	6.37%	6.37%	2.79%	4.40%	6.37%	6.37%	6.37%	5.98%	5.98%	7.51%	7.11%	6.40%	6.75%	6.37%	6.37%	6.75%	0.00%	0.00%	0.00%	0.00%		0	0	0	0	0	0	0	0	0	0	30	30	32	34	15	19	30	30
69. *S. yagiui* SKS583	11.90%	11.90%	11.90%	11.90%	11.90%	11.90%	11.90%	11.90%	11.90%	11.90%	11.90%	11.90%	11.90%	11.90%	11.90%	8.84%	9.64%	3.20%	13.55%	13.55%	13.15%	13.55%	13.55%	13.55%	13.03%	13.15%	12.65%	13.55%	13.15%	13.55%	12.65%	12.65%	13.55%	12.65%	13.04%	13.04%	13.55%	12.75%	13.55%	13.55%	12.50%	13.55%	13.94%	12.30%	12.30%	12.30%	12.30%	6.37%	6.37%	2.79%	4.40%	6.37%	6.37%	6.37%	5.98%	5.98%	7.51%	7.11%	6.40%	6.75%	6.37%	6.37%	6.75%	0.00%	0.00%	0.00%	0.00%	0.00%		0	0	0	0	0	0	0	0	0	30	30	32	34	15	19	30	30
70. *S. yagiui* SKS977	11.90%	11.90%	11.90%	11.90%	11.90%	11.90%	11.90%	11.90%	11.90%	11.90%	11.90%	11.90%	11.90%	11.90%	11.90%	8.84%	9.64%	3.20%	13.55%	13.55%	13.15%	13.55%	13.55%	13.55%	13.03%	13.15%	12.65%	13.55%	13.15%	13.55%	12.65%	12.65%	13.55%	12.65%	13.04%	13.04%	13.55%	12.75%	13.55%	13.55%	12.50%	13.55%	13.94%	12.30%	12.30%	12.30%	12.30%	6.37%	6.37%	2.79%	4.40%	6.37%	6.37%	6.37%	5.98%	5.98%	7.51%	7.11%	6.40%	6.75%	6.37%	6.37%	6.75%	0.00%	0.00%	0.00%	0.00%	0.00%	0.00%		0	0	0	0	0	0	0	0	30	30	32	34	15	19	30	30
71. *S. yagiui* SKS983	11.90%	11.90%	11.90%	11.90%	11.90%	11.90%	11.90%	11.90%	11.90%	11.90%	11.90%	11.90%	11.90%	11.90%	11.90%	8.84%	9.64%	3.20%	13.55%	13.55%	13.15%	13.55%	13.55%	13.55%	13.03%	13.15%	12.65%	13.55%	13.15%	13.55%	12.65%	12.65%	13.55%	12.65%	13.04%	13.04%	13.55%	12.75%	13.55%	13.55%	12.50%	13.55%	13.94%	12.30%	12.30%	12.30%	12.30%	6.37%	6.37%	2.79%	4.40%	6.37%	6.37%	6.37%	5.98%	5.98%	7.51%	7.11%	6.40%	6.75%	6.37%	6.37%	6.75%	0.00%	0.00%	0.00%	0.00%	0.00%	0.00%	0.00%		0	0	0	0	0	0	0	30	30	32	34	15	19	30	30
72. *S. yagiui* SKS964	11.90%	11.90%	11.90%	11.90%	11.90%	11.90%	11.90%	11.90%	11.90%	11.90%	11.90%	11.90%	11.90%	11.90%	11.90%	8.84%	9.64%	3.20%	13.55%	13.55%	13.15%	13.55%	13.55%	13.55%	13.03%	13.15%	12.65%	13.55%	13.15%	13.55%	12.65%	12.65%	13.55%	12.65%	13.04%	13.04%	13.55%	12.75%	13.55%	13.55%	12.50%	13.55%	13.94%	12.30%	12.30%	12.30%	12.30%	6.37%	6.37%	2.79%	4.40%	6.37%	6.37%	6.37%	5.98%	5.98%	7.51%	7.11%	6.40%	6.75%	6.37%	6.37%	6.75%	0.00%	0.00%	0.00%	0.00%	0.00%	0.00%	0.00%	0.00%		0	0	0	0	0	0	30	30	32	34	15	19	30	30
73. *S. yagiui* SKS787	11.90%	11.90%	11.90%	11.90%	11.90%	11.90%	11.90%	11.90%	11.90%	11.90%	11.90%	11.90%	11.90%	11.90%	11.90%	8.84%	9.64%	3.20%	13.55%	13.55%	13.15%	13.55%	13.55%	13.55%	13.03%	13.15%	12.65%	13.55%	13.15%	13.55%	12.65%	12.65%	13.55%	12.65%	13.04%	13.04%	13.55%	12.75%	13.55%	13.55%	12.50%	13.55%	13.94%	12.30%	12.30%	12.30%	12.30%	6.37%	6.37%	2.79%	4.40%	6.37%	6.37%	6.37%	5.98%	5.98%	7.51%	7.11%	6.40%	6.75%	6.37%	6.37%	6.75%	0.00%	0.00%	0.00%	0.00%	0.00%	0.00%	0.00%	0.00%	0.00%		0	0	0	0	0	30	30	32	34	15	19	30	30
74. *S. yagiui* SKS963	11.90%	11.90%	11.90%	11.90%	11.90%	11.90%	11.90%	11.90%	11.90%	11.90%	11.90%	11.90%	11.90%	11.90%	11.90%	8.84%	9.64%	3.20%	13.55%	13.55%	13.15%	13.55%	13.55%	13.55%	13.03%	13.15%	12.65%	13.55%	13.15%	13.55%	12.65%	12.65%	13.55%	12.65%	13.04%	13.04%	13.55%	12.75%	13.55%	13.55%	12.50%	13.55%	13.94%	12.30%	12.30%	12.30%	12.30%	6.37%	6.37%	2.79%	4.40%	6.37%	6.37%	6.37%	5.98%	5.98%	7.51%	7.11%	6.40%	6.75%	6.37%	6.37%	6.75%	0.00%	0.00%	0.00%	0.00%	0.00%	0.00%	0.00%	0.00%	0.00%	0.00%		0	0	0	0	30	30	32	34	15	19	30	30
75. *S. yagiui* Pozz	11.90%	11.90%	11.90%	11.90%	11.90%	11.90%	11.90%	11.90%	11.86%	11.86%	11.86%	11.90%	11.90%	11.90%	11.90%	9.20%	10.00%	3.19%	13.55%	13.55%	13.15%	13.55%	13.55%	13.55%	13.03%	13.15%	12.65%	13.55%	13.15%	13.55%	12.60%	12.60%	13.49%	12.60%	12.99%	12.99%	13.55%	12.75%	13.49%	13.49%	12.45%	13.55%	13.94%	12.30%	12.30%	12.25%	12.25%	6.35%	6.35%	2.78%	4.38%	6.37%	6.37%	6.37%	5.98%	5.98%	7.48%	7.11%	6.40%	6.72%	6.35%	6.35%	6.72%	0.00%	0.00%	0.00%	0.00%	0.00%	0.00%	0.00%	0.00%	0.00%	0.00%	0.00%		0	0	0	30	30	32	34	15	19	30	30
76. *S. yagiui* LarnCyp	11.90%	11.90%	11.90%	11.90%	11.90%	11.90%	11.90%	11.90%	11.86%	11.86%	11.86%	11.90%	11.90%	11.90%	11.90%	9.20%	10.00%	3.19%	13.55%	13.55%	13.15%	13.55%	13.55%	13.55%	13.03%	13.15%	12.65%	13.55%	13.15%	13.55%	12.60%	12.60%	13.49%	12.60%	12.99%	12.99%	13.55%	12.75%	13.49%	13.49%	12.45%	13.55%	13.94%	12.30%	12.30%	12.25%	12.25%	6.35%	6.35%	2.78%	4.38%	6.37%	6.37%	6.37%	5.98%	5.98%	7.48%	7.11%	6.40%	6.72%	6.35%	6.35%	6.72%	0.00%	0.00%	0.00%	0.00%	0.00%	0.00%	0.00%	0.00%	0.00%	0.00%	0.00%	0.00%		0	0	30	30	32	34	15	19	30	30
77. *S. yagiui* SpirWS	11.90%	11.90%	11.90%	11.90%	11.90%	11.90%	11.90%	11.90%	11.86%	11.86%	11.86%	11.90%	11.90%	11.90%	11.90%	9.20%	10.00%	3.19%	13.55%	13.55%	13.15%	13.55%	13.55%	13.55%	13.03%	13.15%	12.65%	13.55%	13.15%	13.55%	12.60%	12.60%	13.49%	12.60%	12.99%	12.99%	13.55%	12.75%	13.49%	13.49%	12.45%	13.55%	13.94%	12.30%	12.30%	12.25%	12.25%	6.35%	6.35%	2.78%	4.38%	6.37%	6.37%	6.37%	5.98%	5.98%	7.48%	7.11%	6.40%	6.72%	6.35%	6.35%	6.72%	0.00%	0.00%	0.00%	0.00%	0.00%	0.00%	0.00%	0.00%	0.00%	0.00%	0.00%	0.00%	0.00%		0	30	30	32	34	15	19	30	30
78. *S. yagiui* GNS4	11.90%	11.90%	11.90%	11.90%	11.90%	11.90%	11.90%	11.90%	11.86%	11.86%	11.86%	11.90%	11.90%	11.90%	11.90%	9.20%	10.00%	3.19%	13.55%	13.55%	13.15%	13.55%	13.55%	13.55%	13.03%	13.15%	12.65%	13.55%	13.15%	13.55%	12.60%	12.60%	13.49%	12.60%	12.99%	12.99%	13.55%	12.75%	13.49%	13.49%	12.45%	13.55%	13.94%	12.30%	12.30%	12.25%	12.25%	6.35%	6.35%	2.78%	4.38%	6.37%	6.37%	6.37%	5.98%	5.98%	7.48%	7.11%	6.40%	6.72%	6.35%	6.35%	6.72%	0.00%	0.00%	0.00%	0.00%	0.00%	0.00%	0.00%	0.00%	0.00%	0.00%	0.00%	0.00%	0.00%	0.00%		30	30	32	34	15	19	30	30
79. *S.* sp. GuaCHN	0.78%	0.78%	0.78%	0.78%	0.78%	0.78%	0.78%	0.78%	1.16%	0.78%	0.78%	0.78%	0.78%	0.78%	0.78%	10.71%	11.07%	9.92%	5.51%	5.51%	5.91%	5.51%	5.51%	5.51%	5.39%	5.12%	8.20%	5.51%	5.12%	5.51%	8.17%	8.17%	5.49%	8.17%	7.78%	7.78%	5.51%	4.72%	5.49%	5.49%	4.37%	5.51%	5.12%	1.17%	1.17%	1.16%	1.16%	9.13%	9.13%	11.42%	9.56%	9.16%	9.16%	9.16%	8.37%	8.37%	10.98%	11.02%	8.80%	9.52%	9.13%	9.13%	9.52%	11.90%	11.90%	11.90%	11.90%	11.90%	11.90%	11.90%	11.90%	11.90%	11.90%	11.90%	11.86%	11.86%	11.86%	11.86%		2	21	13	23	25	21	20
80. *S.* sp. Seef2	0.00%	0.00%	0.00%	0.00%	0.00%	0.00%	0.00%	0.00%	0.39%	0.00%	0.00%	0.00%	0.00%	0.00%	0.00%	10.76%	11.11%	9.96%	6.30%	6.30%	6.69%	6.30%	6.30%	6.30%	6.22%	5.91%	8.98%	6.30%	5.91%	6.30%	8.98%	8.98%	6.30%	8.98%	8.59%	8.59%	6.30%	5.51%	6.30%	6.30%	5.18%	6.30%	5.91%	1.95%	1.95%	1.95%	1.95%	9.16%	9.16%	11.46%	9.20%	9.16%	9.16%	9.16%	8.37%	8.37%	11.02%	11.02%	8.80%	9.16%	9.16%	9.16%	9.16%	11.90%	11.90%	11.90%	11.90%	11.90%	11.90%	11.90%	11.90%	11.90%	11.90%	11.90%	11.90%	11.90%	11.90%	11.90%	0.78%		23	15	23	26	23	22
81. *S.* sp. Thd2	8.98%	8.98%	8.98%	8.98%	8.98%	8.98%	8.98%	8.98%	8.98%	8.98%	8.98%	8.98%	8.98%	8.98%	8.98%	12.70%	13.04%	11.95%	6.30%	6.30%	5.91%	6.30%	6.30%	6.30%	6.22%	6.69%	0.00%	6.30%	6.69%	6.30%	0.00%	0.00%	6.30%	0.00%	0.39%	0.39%	6.30%	7.09%	6.30%	6.30%	5.98%	6.30%	5.91%	8.98%	8.98%	8.98%	8.98%	9.96%	9.96%	10.67%	11.20%	9.96%	9.96%	9.96%	9.56%	9.56%	10.20%	10.20%	9.60%	8.73%	9.96%	9.96%	8.73%	12.65%	12.65%	12.65%	12.65%	12.65%	12.65%	12.65%	12.65%	12.65%	12.65%	12.65%	12.65%	12.65%	12.65%	12.65%	8.20%	8.98%		18	24	24	4	2
82. *S.* sp. SKS255	5.88%	5.88%	5.88%	5.88%	5.88%	5.88%	5.88%	5.88%	5.88%	5.88%	5.88%	5.88%	5.88%	5.88%	5.88%	13.89%	13.89%	11.95%	0.79%	0.79%	1.18%	0.79%	0.79%	0.79%	1.24%	1.18%	7.03%	0.79%	1.18%	0.79%	7.03%	7.03%	0.79%	7.03%	6.64%	6.64%	0.79%	1.57%	0.79%	0.79%	0.80%	0.79%	0.79%	5.49%	5.49%	5.49%	5.49%	11.16%	11.16%	12.65%	11.60%	11.16%	11.16%	11.16%	10.36%	10.36%	12.60%	12.20%	10.80%	11.11%	11.16%	11.16%	11.11%	13.44%	13.44%	13.44%	13.44%	13.44%	13.44%	13.44%	13.44%	13.44%	13.44%	13.44%	13.44%	13.44%	13.44%	13.44%	5.10%	5.88%	7.03%		28	30	19	18
83. *S.* sp. StJFp2	9.16%	9.16%	9.16%	9.16%	9.16%	9.16%	9.16%	9.16%	9.16%	9.16%	9.16%	9.16%	9.16%	9.16%	9.16%	7.29%	7.69%	5.60%	11.95%	11.95%	11.55%	11.95%	11.95%	11.95%	11.76%	11.55%	9.56%	11.95%	11.55%	11.95%	9.56%	9.56%	11.95%	9.56%	9.96%	9.96%	11.95%	11.16%	11.95%	11.95%	10.89%	11.95%	11.55%	9.56%	9.56%	9.56%	9.56%	0.40%	0.40%	4.38%	4.80%	0.40%	0.40%	0.40%	1.59%	1.59%	5.58%	5.18%	0.40%	4.38%	0.40%	0.40%	4.38%	5.98%	5.98%	5.98%	5.98%	5.98%	5.98%	5.98%	5.98%	5.98%	5.98%	5.98%	5.98%	5.98%	5.98%	5.98%	9.16%	9.16%	9.56%	11.16%		15	23	23
84. *S.* sp. StMgN	10.36%	10.36%	10.36%	10.36%	10.36%	10.36%	10.36%	10.36%	10.36%	10.36%	10.36%	10.36%	10.36%	10.36%	10.36%	8.50%	8.91%	7.60%	12.75%	12.75%	12.35%	12.75%	12.75%	12.75%	11.76%	12.35%	9.56%	12.75%	12.35%	12.75%	9.56%	9.56%	12.75%	9.56%	9.96%	9.96%	12.75%	12.35%	12.75%	12.75%	11.69%	12.75%	12.35%	10.36%	10.36%	10.36%	10.36%	6.40%	6.40%	7.97%	6.43%	6.40%	6.40%	6.40%	6.80%	6.80%	3.98%	3.59%	6.43%	5.60%	6.40%	6.40%	5.60%	7.60%	7.60%	7.60%	7.60%	7.60%	7.60%	7.60%	7.60%	7.60%	7.60%	7.60%	7.60%	7.60%	7.60%	7.60%	9.96%	10.36%	9.56%	11.95%	6.00%		24	23
85. *S.* sp. Ind	8.98%	8.98%	8.98%	8.98%	8.98%	8.98%	8.98%	8.98%	8.98%	8.98%	8.98%	8.98%	8.98%	8.98%	8.98%	12.30%	12.25%	11.95%	7.09%	7.09%	6.69%	7.09%	7.09%	7.09%	7.05%	7.48%	1.56%	7.09%	7.48%	7.09%	1.56%	1.56%	7.09%	1.56%	1.95%	1.95%	7.09%	7.87%	7.09%	7.09%	6.77%	7.09%	6.69%	8.98%	8.98%	8.98%	8.98%	9.56%	9.56%	10.28%	10.80%	9.56%	9.56%	9.56%	9.56%	9.56%	9.80%	9.80%	9.20%	8.33%	9.56%	9.56%	8.33%	11.86%	11.86%	11.86%	11.86%	11.86%	11.86%	11.86%	11.86%	11.86%	11.86%	11.86%	11.86%	11.86%	11.86%	11.86%	8.20%	8.98%	1.56%	7.42%	9.16%	9.56%		1
86. *S.* sp. SmJFp1	8.63%	8.63%	8.63%	8.63%	8.63%	8.63%	8.63%	8.63%	8.63%	8.63%	8.63%	8.63%	8.63%	8.63%	8.63%	12.35%	12.70%	11.60%	6.72%	6.72%	6.32%	6.72%	6.72%	6.72%	6.67%	7.11%	0.78%	6.72%	7.11%	6.72%	0.78%	0.78%	6.72%	0.78%	1.17%	1.17%	6.72%	7.51%	6.72%	6.72%	6.40%	6.72%	6.32%	8.63%	8.63%	8.63%	8.63%	9.60%	9.60%	10.32%	10.84%	9.60%	9.60%	9.60%	9.60%	9.60%	9.45%	9.45%	9.24%	8.37%	9.60%	9.60%	8.37%	11.90%	11.90%	11.90%	11.90%	11.90%	11.90%	11.90%	11.90%	11.90%	11.90%	11.90%	11.90%	11.90%	11.90%	11.90%	7.84%	8.63%	0.78%	7.06%	9.20%	9.20%	0.39%	

Supplementary Table S5. Numbers of nucleotide differences (above diagonal) and pairwise distances (below diagonal) of the D1D2 domain of the 28S rRNA gene sequences among 86 *Spirostomum* populations.

Taxa	1	2	3	4	5	6	7	8	9	10	11	12	13	14	15	16	17	18	19	20	21	22	23	24	25	26	27	28	29	30	31	32	33	34	35	36	37	38	39	40	41	42	43	44	45	46	47	48	49	50	51	52	53	54	55	56	57	58	59	60	61	62	63	64	65	66	67	68	69	70	71	72	73	74	75	76	77	78	79	80	81	82	83	84	85	86
1. *S. ambiguum* SKS363		3	3	2	0	0	0	0	4	1	1	0	0	0	3	46	45	19	23	23	22	23	23	23	23	23	27	23	23	23	15	21	22	27	15	26	23	22	22	22	29	23	24	22	23	21	23	25	29	38	45	48	48	48	46	51	51	50	48	23	44	44	23	43	43	43	43	43	43	43	43	43	43	43	19	39	39	21	26	26	1	8	31	28	16	44
2. *S. ambiguum* SKS254	0.43%		0	1	3	3	3	3	1	1	1	3	3	3	0	45	44	19	22	22	20	22	22	22	22	22	26	22	22	22	13	19	20	25	13	24	22	20	20	20	27	22	25	20	22	19	21	25	29	38	45	47	47	47	46	50	50	49	47	23	44	44	23	42	42	42	42	42	42	42	42	42	42	42	19	39	39	21	24	24	1	5	31	27	14	44
3. *S. ambiguum* SKS204	0.43%	0.00%		1	3	3	3	3	1	1	1	3	3	3	0	45	44	19	22	22	20	22	22	22	22	22	26	22	22	22	13	19	20	25	13	24	22	20	20	20	27	22	25	20	22	19	21	25	29	38	45	47	47	47	46	50	50	49	47	23	44	44	23	42	42	42	42	42	42	42	42	42	42	42	19	39	39	21	24	24	1	5	31	27	14	44
4. *S. ambiguum* SKS779	0.29%	0.14%	0.14%		2	2	2	2	2	2	2	2	2	2	1	46	45	20	23	23	21	23	23	23	23	23	27	23	23	23	14	20	21	26	14	25	23	21	21	21	28	23	26	21	23	20	22	26	30	39	46	48	48	48	47	51	51	50	48	24	45	45	24	43	43	43	43	43	43	43	43	43	43	43	20	40	40	22	25	25	2	6	32	28	15	45
5. *S. ambiguum* SKS989	0.00%	0.43%	0.43%	0.29%		0	0	0	4	1	1	0	0	0	3	46	45	19	23	23	22	23	23	23	23	23	27	23	23	23	15	21	22	27	15	26	23	22	22	22	29	23	24	22	23	21	23	25	29	38	45	48	48	48	46	51	51	50	48	23	44	44	23	43	43	43	43	43	43	43	43	43	43	43	19	39	39	21	26	26	1	8	31	28	16	44
6. *S. ambiguum* SKS949	0.00%	0.43%	0.43%	0.29%	0.00%		0	0	4	1	1	0	0	0	3	46	45	19	23	23	22	23	23	23	23	23	27	23	23	23	15	21	22	27	15	26	23	22	22	22	29	23	24	22	23	21	23	25	29	38	45	48	48	48	46	51	51	50	48	23	44	44	23	43	43	43	43	43	43	43	43	43	43	43	19	39	39	21	26	26	1	8	31	28	16	44
7. *S. ambiguum* SKS947	0.00%	0.43%	0.43%	0.29%	0.00%	0.00%		0	4	1	1	0	0	0	3	46	45	19	23	23	22	23	23	23	23	23	27	23	23	23	15	21	22	27	15	26	23	22	22	22	29	23	24	22	23	21	23	25	29	38	45	48	48	48	46	51	51	50	48	23	44	44	23	43	43	43	43	43	43	43	43	43	43	43	19	39	39	21	26	26	1	8	31	28	16	44
8. *S. ambiguum* SKS185	0.00%	0.43%	0.43%	0.29%	0.00%	0.00%	0.00%		4	1	1	0	0	0	3	46	45	19	23	23	22	23	23	23	23	23	27	23	23	23	15	21	22	27	15	26	23	22	22	22	29	23	24	22	23	21	23	25	29	38	45	48	48	48	46	51	51	50	48	23	44	44	23	43	43	43	43	43	43	43	43	43	43	43	19	39	39	21	26	26	1	8	31	28	16	44
9. *S. ambiguum* SA	0.58%	0.15%	0.14%	0.29%	0.58%	0.58%	0.58%	0.58%		2	2	4	4	4	1	44	43	18	21	21	19	21	21	21	21	21	25	21	21	21	12	18	19	24	12	23	21	19	19	19	26	21	24	21	23	20	22	24	28	37	44	46	46	46	45	49	49	48	46	22	43	43	22	41	41	41	41	41	41	41	41	41	41	41	18	38	38	20	23	23	2	6	30	26	13	43
10. *S. ambiguum* SS4-2	0.23%	0.23%	0.23%	0.46%	0.23%	0.23%	0.23%	0.23%	0.46%		0	1	1	1	1	35	36	18	19	19	19	19	19	19	19	19	21	19	19	19	14	20	19	21	14	20	19	19	19	19	23	19	19	16	16	18	17	24	28	30	35	34	34	34	35	35	38	37	34	22	34	34	22	30	30	30	30	30	30	30	30	30	30	30	18	30	30	20	21	21	0	6	30	24	15	34
11. *S. ambiguum* EU201	0.20%	0.20%	0.20%	0.39%	0.20%	0.20%	0.20%	0.20%	0.39%	0.00%		1	1	1	1	40	40	18	21	21	21	21	21	21	21	21	26	21	21	21	14	20	21	26	14	25	21	21	21	21	28	21	22	21	21	20	22	24	28	37	44	43	43	43	45	45	45	44	43	22	43	43	22	38	38	38	38	38	38	38	38	38	38	38	18	38	38	20	25	25	0	6	30	26	15	43
12. *S. ambiguum* SKS1050	0.00%	0.43%	0.43%	0.29%	0.00%	0.00%	0.00%	0.00%	0.58%	0.23%	0.20%		0	0	3	46	45	19	23	23	22	23	23	23	23	23	27	23	23	23	15	21	22	27	15	26	23	22	22	22	29	23	24	22	23	21	23	25	29	38	45	48	48	48	46	51	51	50	48	23	44	44	23	43	43	43	43	43	43	43	43	43	43	43	19	39	39	21	26	26	1	8	31	28	16	44
13. *S. ambiguum* SKS184	0.00%	0.43%	0.43%	0.29%	0.00%	0.00%	0.00%	0.00%	0.58%	0.23%	0.20%	0.00%		0	3	46	45	19	23	23	22	23	23	23	23	23	27	23	23	23	15	21	22	27	15	26	23	22	22	22	29	23	24	22	23	21	23	25	29	38	45	48	48	48	46	51	51	50	48	23	44	44	23	43	43	43	43	43	43	43	43	43	43	43	19	39	39	21	26	26	1	8	31	28	16	44
14. *S. ambiguum* SKS169	0.00%	0.43%	0.43%	0.29%	0.00%	0.00%	0.00%	0.00%	0.58%	0.23%	0.20%	0.00%	0.00%		3	46	45	19	23	23	22	23	23	23	23	23	27	23	23	23	15	21	22	27	15	26	23	22	22	22	29	23	24	22	23	21	23	25	29	38	45	48	48	48	46	51	51	50	48	23	44	44	23	43	43	43	43	43	43	43	43	43	43	43	19	39	39	21	26	26	1	8	31	28	16	44
15. *S. ambiguum* SKS167	0.43%	0.00%	0.00%	0.14%	0.43%	0.43%	0.43%	0.43%	0.14%	0.23%	0.20%	0.43%	0.43%	0.43%		45	44	19	22	22	20	22	22	22	22	22	26	22	22	22	13	19	20	25	13	24	22	20	20	20	27	22	25	20	22	19	21	25	29	38	45	47	47	47	46	50	50	49	47	23	44	44	23	42	42	42	42	42	42	42	42	42	42	42	19	39	39	21	24	24	1	5	31	27	14	44
16. *S. caudatum* SKS813	6.69%	6.54%	6.54%	6.69%	6.69%	6.69%	6.69%	6.69%	6.41%	8.10%	7.84%	6.69%	6.69%	6.69%	6.54%		14	13	46	46	42	46	46	46	46	46	49	46	46	46	27	36	42	47	26	44	46	42	42	42	48	46	47	44	48	40	43	19	20	25	30	30	30	30	30	31	28	27	30	17	28	28	17	27	27	27	27	27	27	27	27	27	27	27	13	26	26	15	45	46	40	50	23	43	33	28
17. *S. caudatum* SKS302	6.57%	6.42%	6.42%	6.57%	6.57%	6.57%	6.57%	6.57%	6.30%	8.33%	7.84%	6.57%	6.57%	6.57%	6.42%	2.04%		14	44	44	41	44	44	44	44	44	48	44	44	44	26	37	41	47	26	44	44	41	41	41	47	44	43	44	47	38	42	23	24	27	38	32	32	32	34	36	37	36	32	19	31	31	20	28	28	28	28	28	28	28	28	28	28	28	14	28	28	17	43	46	40	49	28	41	33	31
18. *S. dharwarensis* SAd	5.59%	5.59%	5.59%	5.88%	5.59%	5.59%	5.59%	5.59%	5.33%	5.29%	5.29%	5.59%	5.59%	5.59%	5.59%	3.83%	4.13%		16	16	16	16	16	16	16	16	21	16	16	16	21	21	16	21	21	21	16	16	16	16	16	16	15	21	21	20	20	9	9	0	10	9	9	9	8	8	11	10	9	7	9	9	7	0	0	0	0	0	0	0	0	0	0	0	0	0	0	0	24	21	18	20	7	18	21	9
19. *S. minus* SKS215	3.31%	3.18%	3.17%	3.31%	3.31%	3.31%	3.31%	3.31%	3.03%	4.39%	4.11%	3.31%	3.31%	3.31%	3.17%	6.69%	6.42%	4.71%		0	0	0	0	0	0	0	25	0	0	0	15	20	0	25	15	24	0	0	0	0	7	0	3	26	26	26	28	15	19	35	39	39	39	39	38	42	43	42	39	17	36	36	17	39	39	39	39	39	39	39	39	39	39	39	16	36	36	16	22	24	21	27	26	11	16	36
20. *S. minus* SKS386	3.31%	3.18%	3.17%	3.31%	3.31%	3.31%	3.31%	3.31%	3.03%	4.39%	4.11%	3.31%	3.31%	3.31%	3.17%	6.69%	6.42%	4.71%	0.00%		0	0	0	0	0	0	25	0	0	0	15	20	0	25	15	24	0	0	0	0	7	0	3	26	26	26	28	15	19	35	39	39	39	39	38	42	43	42	39	17	36	36	17	39	39	39	39	39	39	39	39	39	39	39	16	36	36	16	22	24	21	27	26	11	16	36
21. *S. minus* SKS731	4.31%	3.91%	3.91%	4.11%	4.31%	4.31%	4.31%	4.31%	3.73%	4.39%	4.11%	4.31%	4.31%	4.31%	3.91%	8.24%	8.04%	4.71%	0.00%	0.00%		0	0	0	0	0	25	0	0	0	15	20	0	25	15	24	0	0	0	0	7	0	3	26	26	26	28	15	19	35	39	36	36	36	38	38	39	38	36	17	36	36	17	36	36	36	36	36	36	36	36	36	36	36	16	36	36	16	22	24	21	25	26	9	16	36
22. *S. minus* SKS161	3.31%	3.18%	3.17%	3.31%	3.31%	3.31%	3.31%	3.31%	3.03%	4.39%	4.11%	3.31%	3.31%	3.31%	3.17%	6.69%	6.42%	4.71%	0.00%	0.00%	0.00%		0	0	0	0	25	0	0	0	15	20	0	25	15	24	0	0	0	0	7	0	3	26	26	26	28	15	19	35	39	39	39	39	38	42	43	42	39	17	36	36	17	39	39	39	39	39	39	39	39	39	39	39	16	36	36	16	22	24	21	27	26	11	16	36
23. *S. minus* SKS182	3.31%	3.18%	3.17%	3.31%	3.31%	3.31%	3.31%	3.31%	3.03%	4.39%	4.11%	3.31%	3.31%	3.31%	3.17%	6.69%	6.42%	4.71%	0.00%	0.00%	0.00%	0.00%		0	0	0	25	0	0	0	15	20	0	25	15	24	0	0	0	0	7	0	3	26	26	26	28	15	19	35	39	39	39	39	38	42	43	42	39	17	36	36	17	39	39	39	39	39	39	39	39	39	39	39	16	36	36	16	22	24	21	27	26	11	16	36
24. *S. minus* SKS214	3.31%	3.18%	3.17%	3.31%	3.31%	3.31%	3.31%	3.31%	3.03%	4.39%	4.11%	3.31%	3.31%	3.31%	3.17%	6.69%	6.42%	4.71%	0.00%	0.00%	0.00%	0.00%	0.00%		0	0	25	0	0	0	15	20	0	25	15	24	0	0	0	0	7	0	3	26	26	26	28	15	19	35	39	39	39	39	38	42	43	42	39	17	36	36	17	39	39	39	39	39	39	39	39	39	39	39	16	36	36	16	22	24	21	27	26	11	16	36
25. *S. minus* SKS939	3.65%	3.49%	3.49%	3.65%	3.65%	3.65%	3.65%	3.65%	3.33%	4.73%	4.38%	3.65%	3.65%	3.65%	3.49%	7.31%	7.00%	5.18%	0.00%	0.00%	0.00%	0.00%	0.00%	0.00%		0	25	0	0	0	15	20	0	25	15	24	0	0	0	0	7	0	3	26	26	26	28	15	19	35	39	39	39	39	38	42	43	42	39	17	36	36	17	39	39	39	39	39	39	39	39	39	39	39	16	36	36	16	22	24	21	27	26	11	16	36
26. *S. minus* SKS940	3.31%	3.18%	3.17%	3.31%	3.31%	3.31%	3.31%	3.31%	3.03%	4.39%	4.11%	3.31%	3.31%	3.31%	3.17%	6.69%	6.42%	4.71%	0.00%	0.00%	0.00%	0.00%	0.00%	0.00%	0.00%		25	0	0	0	15	20	0	25	15	24	0	0	0	0	7	0	3	26	26	26	28	15	19	35	39	39	39	39	38	42	43	42	39	17	36	36	17	39	39	39	39	39	39	39	39	39	39	39	16	36	36	16	22	24	21	27	26	11	16	36
27. *S. minus* SKS991	4.66%	4.49%	4.49%	4.66%	4.66%	4.66%	4.66%	4.66%	4.33%	4.85%	5.09%	4.66%	4.66%	4.66%	4.49%	8.48%	8.30%	6.18%	4.32%	4.32%	4.89%	4.32%	4.32%	4.32%	4.56%	4.32%		25	25	25	0	0	25	0	0	5	25	25	25	24	31	25	28	33	33	30	31	21	26	39	47	47	47	47	47	49	47	46	47	23	46	45	23	42	42	42	42	42	42	42	42	42	42	42	21	41	41	21	9	0	25	31	31	34	1	46
28. *S. minus* SKS743	3.31%	3.18%	3.17%	3.31%	3.31%	3.31%	3.31%	3.31%	3.03%	4.39%	4.11%	3.31%	3.31%	3.31%	3.17%	6.69%	6.42%	4.71%	0.00%	0.00%	0.00%	0.00%	0.00%	0.00%	0.00%	0.00%	4.32%		0	0	15	20	0	25	15	24	0	0	0	0	7	0	3	26	26	26	28	15	19	35	39	39	39	39	38	42	43	42	39	17	36	36	17	39	39	39	39	39	39	39	39	39	39	39	16	36	36	16	22	24	21	27	26	11	16	36
29. *S. minus* SKS794	3.31%	3.18%	3.17%	3.31%	3.31%	3.31%	3.31%	3.31%	3.03%	4.39%	4.11%	3.31%	3.31%	3.31%	3.17%	6.69%	6.42%	4.71%	0.00%	0.00%	0.00%	0.00%	0.00%	0.00%	0.00%	0.00%	4.32%	0.00%		0	15	20	0	25	15	24	0	0	0	0	7	0	3	26	26	26	28	15	19	35	39	39	39	39	38	42	43	42	39	17	36	36	17	39	39	39	39	39	39	39	39	39	39	39	16	36	36	16	22	24	21	27	26	11	16	36
30. *S. minus* SKS840	3.31%	3.18%	3.17%	3.31%	3.31%	3.31%	3.31%	3.31%	3.03%	4.39%	4.11%	3.31%	3.31%	3.31%	3.17%	6.69%	6.42%	4.71%	0.00%	0.00%	0.00%	0.00%	0.00%	0.00%	0.00%	0.00%	4.32%	0.00%	0.00%		15	20	0	25	15	24	0	0	0	0	7	0	3	26	26	26	28	15	19	35	39	39	39	39	38	42	43	42	39	17	36	36	17	39	39	39	39	39	39	39	39	39	39	39	16	36	36	16	22	24	21	27	26	11	16	36
31. *S. minus* Gmn	4.14%	3.59%	3.59%	3.87%	4.14%	4.14%	4.14%	4.14%	3.33%	3.87%	3.87%	4.14%	4.14%	4.14%	3.59%	7.48%	7.20%	6.18%	4.14%	4.14%	4.14%	4.14%	4.14%	4.14%	4.53%	4.14%	0.00%	4.14%	4.14%	4.14%		0	15	0	0	0	15	15	15	15	16	15	16	16	16	15	15	21	21	21	25	21	21	21	22	22	24	23	21	23	21	21	23	21	21	21	21	21	21	21	21	21	21	21	21	21	21	21	4	0	14	14	23	17	0	21
32. *S. minus* SAm	4.99%	4.51%	4.51%	4.75%	4.99%	4.99%	4.99%	4.99%	4.30%	4.75%	4.75%	4.99%	4.99%	4.99%	4.51%	8.57%	8.81%	6.18%	4.75%	4.75%	4.75%	4.75%	4.75%	4.75%	5.13%	4.75%	0.00%	4.75%	4.75%	4.75%	0.00%		20	0	0	3	20	20	20	20	22	20	22	24	24	24	23	21	26	28	32	29	29	29	30	30	31	30	29	23	29	29	23	28	28	28	28	28	28	28	28	28	28	28	21	28	28	21	8	0	20	23	31	23	1	29
33. *S. minus* SmPS	4.45%	4.05%	4.05%	4.25%	4.45%	4.45%	4.45%	4.45%	3.86%	4.39%	4.25%	4.45%	4.45%	4.45%	4.05%	8.52%	8.32%	4.71%	0.00%	0.00%	0.00%	0.00%	0.00%	0.00%	0.00%	0.00%	5.06%	0.00%	0.00%	0.00%	4.14%	4.75%		25	15	24	0	0	0	0	7	0	2	26	26	26	28	15	19	35	39	36	36	36	38	38	39	38	36	17	36	36	17	36	36	36	36	36	36	36	36	36	36	36	16	36	36	16	22	24	21	25	26	9	16	36
34. *S. minus* EU200	5.28%	4.89%	4.89%	5.09%	5.28%	5.28%	5.28%	5.28%	4.72%	4.85%	5.09%	5.28%	5.28%	5.28%	4.89%	9.22%	9.22%	6.18%	4.89%	4.89%	4.89%	4.89%	4.89%	4.89%	5.21%	4.89%	0.00%	4.89%	4.89%	4.89%	0.00%	0.00%	5.06%		0	5	25	25	25	24	31	25	28	33	33	30	31	21	26	39	47	46	46	46	47	47	45	44	46	23	46	45	23	41	41	41	41	41	41	41	41	41	41	41	21	41	41	21	9	0	25	30	31	34	1	46
35. *S. minus* Mdg2-2	4.18%	3.62%	3.62%	3.90%	4.18%	4.18%	4.18%	4.18%	3.36%	3.90%	3.90%	4.18%	4.18%	4.18%	3.62%	7.26%	7.26%	6.18%	4.18%	4.18%	4.18%	4.18%	4.18%	4.18%	4.57%	4.18%	0.00%	4.18%	4.18%	4.18%	0.00%	0.00%	4.18%	0.00%		0	15	15	15	15	16	15	16	16	16	15	15	21	21	21	24	21	21	21	22	22	23	22	21	22	21	21	22	21	21	21	21	21	21	21	21	21	21	21	21	21	21	21	4	0	14	14	22	17	0	21
36. *S. minus* Mdg4	5.26%	4.86%	4.86%	5.06%	5.26%	5.26%	5.26%	5.26%	4.67%	4.62%	5.06%	5.26%	5.26%	5.26%	4.86%	8.92%	8.92%	6.18%	4.86%	4.86%	4.86%	4.86%	4.86%	4.86%	5.18%	4.86%	1.01%	4.86%	4.86%	4.86%	0.00%	0.71%	4.86%	1.01%	0.00%		24	24	24	23	30	24	26	31	31	28	29	21	25	37	47	43	43	43	45	45	43	42	43	23	43	42	23	39	39	39	39	39	39	39	39	39	39	39	21	39	39	21	7	5	24	29	31	29	1	43
37. *S. minus* SKS1044	3.31%	3.18%	3.17%	3.31%	3.31%	3.31%	3.31%	3.31%	3.03%	4.39%	4.11%	3.31%	3.31%	3.31%	3.17%	6.69%	6.42%	4.71%	0.00%	0.00%	0.00%	0.00%	0.00%	0.00%	0.00%	0.00%	4.32%	0.00%	0.00%	0.00%	4.14%	4.75%	0.00%	4.89%	4.18%	4.86%		0	0	0	7	0	3	26	26	26	28	15	19	35	39	39	39	39	38	42	43	42	39	17	36	36	17	39	39	39	39	39	39	39	39	39	39	39	16	36	36	16	22	24	21	27	26	11	16	36
38. *S. minus* GuS1058	4.29%	3.90%	3.90%	4.09%	4.29%	4.29%	4.29%	4.29%	3.72%	4.39%	4.11%	4.29%	4.29%	4.29%	3.90%	8.20%	8.01%	4.71%	0.00%	0.00%	0.00%	0.00%	0.00%	0.00%	0.00%	0.00%	4.87%	0.00%	0.00%	0.00%	4.14%	4.75%	0.00%	4.89%	4.18%	4.86%	0.00%		0	0	7	0	3	26	26	26	28	15	19	35	39	36	36	36	38	38	39	38	36	17	36	36	17	36	36	36	36	36	36	36	36	36	36	36	16	36	36	16	22	24	21	25	26	9	16	36
39. *S. minus* SKS1073	4.26%	3.87%	3.87%	4.06%	4.26%	4.26%	4.26%	4.26%	3.69%	4.39%	4.11%	4.26%	4.26%	4.26%	3.87%	8.14%	7.95%	4.71%	0.00%	0.00%	0.00%	0.00%	0.00%	0.00%	0.00%	0.00%	4.84%	0.00%	0.00%	0.00%	4.14%	4.75%	0.00%	4.89%	4.18%	4.86%	0.00%	0.00%		0	7	0	3	26	26	26	28	15	19	35	39	36	36	36	38	38	39	38	36	17	36	36	17	36	36	36	36	36	36	36	36	36	36	36	16	36	36	16	22	24	21	25	26	9	16	36
40. *S. minus* PBG1	4.82%	4.39%	4.39%	4.61%	4.82%	4.82%	4.82%	4.82%	4.19%	4.39%	4.61%	4.82%	4.82%	4.82%	4.39%	9.23%	9.01%	4.71%	0.00%	0.00%	0.00%	0.00%	0.00%	0.00%	0.00%	0.00%	5.26%	0.00%	0.00%	0.00%	4.14%	4.75%	0.00%	5.26%	4.18%	5.04%	0.00%	0.00%	0.00%		5	0	2	26	26	26	28	15	19	35	38	36	36	36	37	37	39	38	36	17	36	36	17	35	35	35	35	35	35	35	35	35	35	35	16	35	35	16	22	24	21	25	26	9	16	36
41. *S. minus* SS5	6.17%	5.74%	5.74%	5.96%	6.17%	6.17%	6.17%	6.17%	5.56%	5.36%	5.96%	6.17%	6.17%	6.17%	5.74%	10.23%	10.02%	4.75%	1.49%	1.49%	1.49%	1.49%	1.49%	1.49%	1.59%	1.49%	6.60%	1.49%	1.49%	1.49%	4.46%	5.28%	1.49%	6.60%	4.49%	6.38%	1.49%	1.49%	1.49%	1.11%		7	9	32	32	30	32	16	21	40	44	43	43	43	44	44	46	45	43	18	43	41	18	42	42	42	42	42	42	42	42	42	42	42	16	42	42	17	27	29	26	31	28	16	18	43
42. *S. minus* SKS1018	3.31%	3.18%	3.17%	3.31%	3.31%	3.31%	3.31%	3.31%	3.03%	4.39%	4.11%	3.31%	3.31%	3.31%	3.17%	6.69%	6.42%	4.71%	0.00%	0.00%	0.00%	0.00%	0.00%	0.00%	0.00%	0.00%	4.32%	0.00%	0.00%	0.00%	4.14%	4.75%	0.00%	4.89%	4.18%	4.86%	0.00%	0.00%	0.00%	0.00%	1.49%		3	26	26	26	28	15	19	35	39	39	39	39	38	42	43	42	39	17	36	36	17	39	39	39	39	39	39	39	39	39	39	39	16	36	36	16	22	24	21	27	26	11	16	36
43. *S. semivirescens* DoEng	3.46%	3.62%	3.60%	3.75%	3.46%	3.46%	3.46%	3.46%	3.47%	4.39%	4.31%	3.46%	3.46%	3.46%	3.60%	6.83%	6.28%	4.41%	0.43%	0.43%	0.59%	0.43%	0.43%	0.43%	0.48%	0.43%	4.84%	0.43%	0.43%	0.43%	4.42%	5.23%	0.40%	5.48%	4.46%	5.26%	0.43%	0.58%	0.58%	0.44%	1.91%	0.43%		29	29	26	29	15	19	35	39	40	40	40	39	43	44	43	40	16	37	36	17	40	40	40	40	40	40	40	40	40	40	40	15	36	36	15	25	27	22	31	27	15	19	37
44. *S. subtilis* SKS1056	4.30%	3.91%	3.91%	4.10%	4.30%	4.30%	4.30%	4.30%	4.12%	3.70%	4.11%	4.30%	4.30%	4.30%	3.91%	8.61%	8.61%	6.18%	5.08%	5.08%	5.09%	5.08%	5.08%	5.08%	5.41%	5.08%	6.45%	5.08%	5.08%	5.08%	4.42%	5.70%	5.26%	6.46%	4.46%	6.28%	5.08%	5.08%	5.08%	5.70%	6.81%	5.08%	5.66%		0	4	3	23	27	41	45	45	45	45	47	47	46	45	45	22	45	45	22	42	42	42	42	42	42	42	42	42	42	42	21	42	42	21	30	32	21	23	31	30	18	45
45. *S. subtilis* SKS937	3.31%	3.18%	3.17%	3.31%	3.31%	3.31%	3.31%	3.31%	3.32%	3.70%	4.11%	3.31%	3.31%	3.31%	3.17%	6.98%	6.86%	6.18%	3.75%	3.75%	5.09%	3.75%	3.75%	3.75%	4.13%	3.75%	5.70%	3.75%	3.75%	3.75%	4.42%	5.70%	5.26%	6.46%	4.46%	6.28%	3.75%	5.07%	5.03%	5.70%	6.81%	3.75%	4.18%	0.00%		4	3	23	27	41	45	48	48	48	47	51	50	49	48	22	45	45	22	45	45	45	45	45	45	45	45	45	45	45	21	42	42	21	30	32	21	25	31	32	18	45
46. *S. subtilis* SS4-1	4.72%	4.27%	4.27%	4.49%	4.72%	4.72%	4.72%	4.72%	4.51%	4.17%	4.49%	4.72%	4.72%	4.72%	4.27%	9.01%	8.56%	5.90%	5.84%	5.84%	5.84%	5.84%	5.84%	5.84%	6.28%	5.84%	6.74%	5.84%	5.84%	5.84%	4.16%	5.71%	5.84%	6.74%	4.19%	6.29%	5.84%	5.84%	5.84%	5.84%	6.80%	5.84%	5.84%	0.90%	0.90%		0	23	27	35	42	41	41	41	42	42	42	41	41	21	41	41	22	35	35	35	35	35	35	35	35	35	35	35	20	35	35	20	29	30	20	22	31	30	18	41
47. *S. subtilis* Zur3	5.07%	4.63%	4.63%	4.85%	5.07%	5.07%	5.07%	5.07%	4.87%	3.94%	4.85%	5.07%	5.07%	5.07%	4.63%	9.49%	9.27%	5.90%	6.17%	6.17%	6.17%	6.17%	6.17%	6.17%	6.62%	6.17%	6.83%	6.17%	6.17%	6.17%	4.16%	5.49%	6.17%	6.83%	4.19%	6.39%	6.17%	6.17%	6.17%	6.17%	7.11%	6.17%	6.39%	0.66%	0.66%	0.00%		22	26	40	46	46	46	46	47	47	45	44	46	21	46	46	21	40	40	40	40	40	40	40	40	40	40	40	20	40	40	20	30	31	22	24	30	32	17	46
48. *S. teres* PFEUSm2	6.27%	6.27%	6.27%	6.52%	6.27%	6.27%	6.27%	6.27%	6.05%	6.02%	6.02%	6.27%	6.27%	6.27%	6.27%	4.77%	5.78%	2.65%	3.76%	3.76%	3.76%	3.76%	3.76%	3.76%	4.08%	3.76%	5.26%	3.76%	3.76%	3.76%	5.85%	5.26%	3.76%	5.26%	5.90%	5.26%	3.76%	3.76%	3.76%	3.76%	4.05%	3.76%	3.76%	5.76%	5.76%	5.78%	5.54%		0	9	15	0	0	0	1	1	13	12	0	13	0	0	13	9	9	9	9	9	9	9	9	9	9	9	9	9	9	9	24	21	24	27	17	17	21	0
49. *S. teres* NorKD	7.14%	7.14%	7.14%	7.39%	7.14%	7.14%	7.14%	7.14%	6.93%	6.90%	6.90%	7.14%	7.14%	7.14%	7.14%	4.94%	5.93%	2.65%	4.68%	4.68%	4.68%	4.68%	4.68%	4.68%	5.07%	4.68%	6.40%	4.68%	4.68%	4.68%	5.85%	6.40%	4.68%	6.40%	5.90%	6.16%	4.68%	4.68%	4.68%	4.68%	5.22%	4.68%	4.68%	6.65%	6.65%	6.67%	6.44%	0.00%		10	17	0	0	0	2	2	16	15	0	13	0	0	13	10	10	10	10	10	10	10	10	10	10	10	9	10	10	9	27	26	28	31	20	21	25	0
50. *S. teres* Mdg3	8.48%	8.48%	8.48%	8.71%	8.48%	8.48%	8.48%	8.48%	8.30%	7.03%	8.26%	8.48%	8.48%	8.48%	8.48%	5.59%	6.04%	0.00%	7.81%	7.81%	7.81%	7.81%	7.81%	7.81%	8.39%	7.81%	8.71%	7.81%	7.81%	7.81%	5.88%	6.73%	7.81%	8.71%	5.93%	8.26%	7.81%	7.81%	7.81%	7.81%	9.01%	7.81%	7.81%	9.15%	9.15%	7.97%	8.97%	2.27%	2.48%		19	15	15	15	13	13	21	20	15	9	15	15	9	0	0	0	0	0	0	0	0	0	0	0	0	0	0	0	40	39	37	43	14	32	24	15
51. *S. teres* PF1a	9.20%	9.20%	9.20%	9.41%	9.20%	9.20%	9.20%	9.20%	9.03%	8.18%	9.00%	9.20%	9.20%	9.20%	9.20%	6.15%	7.79%	2.94%	7.98%	7.98%	7.98%	7.98%	7.98%	7.98%	8.52%	7.98%	9.61%	7.98%	7.98%	7.98%	7.00%	7.69%	7.98%	9.61%	6.78%	9.61%	7.98%	7.98%	7.98%	8.43%	9.46%	7.98%	7.98%	9.20%	9.20%	9.55%	10.24%	3.78%	4.21%	4.24%		23	23	23	20	20	23	22	23	14	23	22	14	19	19	19	19	19	19	19	19	19	19	19	10	19	19	10	45	45	43	48	17	38	28	23
52. *S. teres* SKS187	6.95%	6.83%	6.80%	6.95%	6.95%	6.95%	6.95%	6.95%	6.68%	7.91%	8.46%	6.95%	6.95%	6.95%	6.80%	4.38%	4.69%	2.65%	5.64%	5.64%	7.09%	5.64%	5.64%	5.64%	6.22%	5.64%	8.16%	5.64%	5.64%	5.64%	5.85%	6.94%	7.33%	9.06%	5.90%	8.76%	5.64%	7.06%	7.00%	7.95%	9.21%	5.64%	5.79%	8.84%	6.95%	9.28%	10.20%	0.00%	0.00%	3.35%	4.70%		0	0	5	6	24	23	0	13	0	0	13	17	17	17	17	17	17	17	17	17	17	17	9	16	16	9	44	45	43	50	22	36	25	0
53. *S. teres* SKS107	6.95%	6.83%	6.80%	6.95%	6.95%	6.95%	6.95%	6.95%	6.68%	7.91%	8.46%	6.95%	6.95%	6.95%	6.80%	4.38%	4.69%	2.65%	5.64%	5.64%	7.09%	5.64%	5.64%	5.64%	6.22%	5.64%	8.16%	5.64%	5.64%	5.64%	5.85%	6.94%	7.33%	9.06%	5.90%	8.76%	5.64%	7.06%	7.00%	7.95%	9.21%	5.64%	5.79%	8.84%	6.95%	9.28%	10.20%	0.00%	0.00%	3.35%	4.70%	0.00%		0	5	6	24	23	0	13	0	0	13	17	17	17	17	17	17	17	17	17	17	17	9	16	16	9	44	45	43	50	22	36	25	0
54. *S. teres* SKS988	6.95%	6.83%	6.80%	6.95%	6.95%	6.95%	6.95%	6.95%	6.68%	7.91%	8.46%	6.95%	6.95%	6.95%	6.80%	4.38%	4.69%	2.65%	5.64%	5.64%	7.09%	5.64%	5.64%	5.64%	6.22%	5.64%	8.16%	5.64%	5.64%	5.64%	5.85%	6.94%	7.33%	9.06%	5.90%	8.76%	5.64%	7.06%	7.00%	7.95%	9.21%	5.64%	5.79%	8.84%	6.95%	9.28%	10.20%	0.00%	0.00%	3.35%	4.70%	0.00%	0.00%		5	6	24	23	0	13	0	0	13	17	17	17	17	17	17	17	17	17	17	17	9	16	16	9	44	45	43	50	22	36	25	0
55. *S. teres* SKS351	8.98%	8.98%	8.98%	9.18%	8.98%	8.98%	8.98%	8.98%	8.82%	8.14%	8.86%	8.98%	8.98%	8.98%	8.98%	5.87%	6.65%	2.35%	7.42%	7.42%	7.48%	7.42%	7.42%	7.42%	7.90%	7.42%	9.18%	7.42%	7.42%	7.42%	6.13%	7.18%	7.74%	9.25%	6.18%	9.16%	7.42%	7.45%	7.42%	8.17%	9.42%	7.42%	7.62%	9.23%	9.18%	9.50%	10.42%	0.25%	0.49%	2.90%	4.09%	0.98%	0.98%	0.98%		0	22	21	5	12	5	4	12	13	13	13	13	13	13	13	13	13	13	13	8	13	13	8	46	45	44	49	21	35	25	5
56. *S. teres* SKS026	7.38%	7.27%	7.24%	7.38%	7.38%	7.38%	7.38%	7.38%	7.11%	8.14%	8.86%	7.38%	7.38%	7.38%	7.24%	4.53%	5.28%	2.35%	6.08%	6.08%	7.48%	6.08%	6.08%	6.08%	6.70%	6.08%	8.51%	6.08%	6.08%	6.08%	6.13%	7.18%	7.74%	9.25%	6.18%	9.16%	6.08%	7.45%	7.39%	8.17%	9.42%	6.08%	6.22%	9.23%	7.38%	9.50%	10.42%	0.25%	0.49%	2.90%	4.09%	0.87%	0.87%	0.87%	0.00%		23	22	6	12	5	4	12	15	15	15	15	15	15	15	15	15	15	15	8	13	13	8	46	45	44	53	21	39	25	5
57. *S. teres* SPT	7.38%	7.27%	7.24%	7.38%	7.38%	7.38%	7.38%	7.38%	7.11%	8.84%	8.86%	7.38%	7.38%	7.38%	7.24%	4.09%	5.43%	3.24%	6.22%	6.22%	7.68%	6.22%	6.22%	6.22%	6.86%	6.22%	8.16%	6.22%	6.22%	6.22%	6.69%	7.42%	7.94%	8.86%	6.46%	8.76%	6.22%	7.65%	7.59%	8.61%	9.85%	6.22%	6.37%	9.04%	7.24%	9.50%	9.98%	3.26%	3.94%	4.69%	4.70%	3.47%	3.47%	3.47%	4.30%	3.33%		1	24	9	22	22	9	23	23	23	23	23	23	23	23	23	23	23	11	22	22	11	44	44	45	55	14	40	27	22
58. *S. teres* SKS1045	7.24%	7.12%	7.09%	7.24%	7.24%	7.24%	7.24%	7.24%	6.97%	8.60%	8.66%	7.24%	7.24%	7.24%	7.09%	3.94%	5.28%	2.94%	6.08%	6.08%	7.48%	6.08%	6.08%	6.08%	6.70%	6.08%	7.99%	6.08%	6.08%	6.08%	6.41%	7.18%	7.74%	8.66%	6.18%	8.55%	6.08%	7.45%	7.39%	8.39%	9.64%	6.08%	6.22%	8.84%	7.09%	9.28%	9.76%	3.01%	3.69%	4.46%	4.50%	3.33%	3.33%	3.33%	4.10%	3.18%	0.14%		23	8	21	21	8	22	22	22	22	22	22	22	22	22	22	22	10	21	21	10	43	43	44	54	13	39	26	21
59. *S. teres* SKS085	7.31%	7.15%	7.15%	7.31%	7.31%	7.31%	7.31%	7.31%	7.00%	8.52%	9.01%	7.31%	7.31%	7.31%	7.15%	4.59%	4.92%	2.91%	5.94%	5.94%	7.55%	5.94%	5.94%	5.94%	6.22%	5.94%	8.62%	5.94%	5.94%	5.94%	6.40%	7.49%	7.83%	9.64%	6.46%	9.35%	5.94%	7.52%	7.45%	8.53%	9.86%	5.94%	6.09%	9.41%	7.31%	9.98%	10.95%	0.00%	0.00%	3.60%	5.02%	0.00%	0.00%	0.00%	1.04%	0.91%	3.65%	3.50%		13	0	0	13	17	17	17	17	17	17	17	17	17	17	17	9	16	16	9	44	45	43	50	22	36	25	0
60. *S. teres* Mdg2-1	6.44%	6.44%	6.44%	6.72%	6.44%	6.44%	6.44%	6.44%	6.20%	6.16%	6.16%	6.44%	6.44%	6.44%	6.44%	4.78%	5.34%	2.06%	4.76%	4.76%	4.76%	4.76%	4.76%	4.76%	5.21%	4.76%	6.44%	4.76%	4.76%	4.76%	6.44%	6.44%	4.76%	6.44%	6.21%	6.44%	4.76%	4.76%	4.76%	4.76%	5.08%	4.76%	4.48%	6.16%	6.16%	5.90%	5.90%	3.64%	3.64%	2.52%	3.92%	3.64%	3.64%	3.64%	3.36%	3.36%	2.52%	2.24%	3.99%		13	13	0	9	9	9	9	9	9	9	9	9	9	9	7	9	9	8	26	23	22	24	5	19	23	13
61. *S. teres* EU199	8.66%	8.66%	8.66%	8.86%	8.66%	8.66%	8.66%	8.66%	8.50%	7.91%	8.46%	8.66%	8.66%	8.66%	8.66%	5.52%	6.11%	2.65%	7.09%	7.09%	7.09%	7.09%	7.09%	7.09%	7.55%	7.09%	9.06%	7.09%	7.09%	7.09%	5.85%	6.94%	7.33%	9.06%	5.90%	8.76%	7.09%	7.09%	7.09%	7.95%	9.21%	7.09%	7.28%	8.86%	8.86%	9.28%	10.20%	0.00%	0.00%	3.35%	4.70%	0.00%	0.00%	0.00%	0.98%	0.98%	4.33%	4.13%	0.00%	3.64%		0	13	16	16	16	16	16	16	16	16	16	16	16	9	16	16	9	44	45	43	47	22	33	25	0
62. *S. teres* NorBG	9.71%	9.71%	9.71%	9.93%	9.71%	9.71%	9.71%	9.71%	9.53%	7.91%	9.49%	9.71%	9.71%	9.71%	9.71%	6.19%	6.86%	2.65%	7.95%	7.95%	7.95%	7.95%	7.95%	7.95%	8.53%	7.95%	9.93%	7.95%	7.95%	7.95%	5.85%	6.94%	7.95%	9.93%	5.90%	9.27%	7.95%	7.95%	7.95%	7.95%	9.13%	7.95%	7.95%	9.93%	9.93%	9.28%	10.20%	0.00%	0.00%	3.35%	4.88%	0.00%	0.00%	0.00%	0.88%	0.88%	4.86%	4.64%	0.00%	3.64%	0.00%		13	15	15	15	15	15	15	15	15	15	15	15	9	15	15	9	44	45	43	47	22	33	25	0
63. *S. teres* Seef1	6.34%	6.34%	6.34%	6.61%	6.34%	6.34%	6.34%	6.34%	6.09%	6.06%	6.06%	6.34%	6.34%	6.34%	6.34%	4.70%	5.52%	2.06%	4.68%	4.68%	4.68%	4.68%	4.68%	4.68%	5.12%	4.68%	6.34%	4.68%	4.68%	4.68%	6.44%	6.34%	4.68%	6.34%	6.21%	6.34%	4.68%	4.68%	4.68%	4.68%	5.00%	4.68%	4.68%	6.06%	6.06%	6.08%	5.82%	3.58%	3.58%	2.48%	3.86%	3.58%	3.58%	3.58%	3.31%	3.31%	2.48%	2.20%	3.92%	0.00%	3.58%	3.58%		9	9	9	9	9	9	9	9	9	9	9	7	9	9	8	26	23	22	25	5	19	23	13
64. *S. yagiui* SKS165	6.24%	6.12%	6.10%	6.24%	6.24%	6.24%	6.24%	6.24%	5.97%	7.01%	7.51%	6.24%	6.24%	6.24%	6.10%	3.95%	4.12%	0.00%	5.66%	5.66%	7.11%	5.66%	5.66%	5.66%	6.24%	5.66%	7.32%	5.66%	5.66%	5.66%	5.88%	6.73%	7.36%	8.10%	5.93%	7.98%	5.66%	7.09%	7.03%	7.76%	9.03%	5.66%	5.81%	8.28%	6.53%	7.95%	8.91%	2.27%	2.48%	0.00%	3.89%	2.47%	2.47%	2.47%	2.55%	2.18%	3.34%	3.19%	2.60%	2.52%	3.16%	3.33%	2.48%		0	0	0	0	0	0	0	0	0	0	0	0	0	0	40	39	37	47	14	36	24	16
65. *S. yagiui* SKS315	6.24%	6.12%	6.10%	6.24%	6.24%	6.24%	6.24%	6.24%	5.97%	7.01%	7.51%	6.24%	6.24%	6.24%	6.10%	3.95%	4.12%	0.00%	5.66%	5.66%	7.11%	5.66%	5.66%	5.66%	6.24%	5.66%	7.32%	5.66%	5.66%	5.66%	5.88%	6.73%	7.36%	8.10%	5.93%	7.98%	5.66%	7.09%	7.03%	7.76%	9.03%	5.66%	5.81%	8.28%	6.53%	7.95%	8.91%	2.27%	2.48%	0.00%	3.89%	2.47%	2.47%	2.47%	2.55%	2.18%	3.34%	3.19%	2.60%	2.52%	3.16%	3.33%	2.48%	0.00%		0	0	0	0	0	0	0	0	0	0	0	0	0	40	39	37	47	14	36	24	16
66. *S. yagiui* SKS164	6.24%	6.12%	6.10%	6.24%	6.24%	6.24%	6.24%	6.24%	5.97%	7.01%	7.51%	6.24%	6.24%	6.24%	6.10%	3.95%	4.12%	0.00%	5.66%	5.66%	7.11%	5.66%	5.66%	5.66%	6.24%	5.66%	7.32%	5.66%	5.66%	5.66%	5.88%	6.73%	7.36%	8.10%	5.93%	7.98%	5.66%	7.09%	7.03%	7.76%	9.03%	5.66%	5.81%	8.28%	6.53%	7.95%	8.91%	2.27%	2.48%	0.00%	3.89%	2.47%	2.47%	2.47%	2.55%	2.18%	3.34%	3.19%	2.60%	2.52%	3.16%	3.33%	2.48%	0.00%	0.00%		0	0	0	0	0	0	0	0	0	0	0	0	40	39	37	47	14	36	24	16
67. *S. yagiui* SKS071	6.24%	6.12%	6.10%	6.24%	6.24%	6.24%	6.24%	6.24%	5.97%	7.01%	7.51%	6.24%	6.24%	6.24%	6.10%	3.95%	4.12%	0.00%	5.66%	5.66%	7.11%	5.66%	5.66%	5.66%	6.24%	5.66%	7.32%	5.66%	5.66%	5.66%	5.88%	6.73%	7.36%	8.10%	5.93%	7.98%	5.66%	7.09%	7.03%	7.76%	9.03%	5.66%	5.81%	8.28%	6.53%	7.95%	8.91%	2.27%	2.48%	0.00%	3.89%	2.47%	2.47%	2.47%	2.55%	2.18%	3.34%	3.19%	2.60%	2.52%	3.16%	3.33%	2.48%	0.00%	0.00%	0.00%		0	0	0	0	0	0	0	0	0	0	0	40	39	37	47	14	36	24	16
68. *S. yagiui* JpH1020	6.24%	6.12%	6.10%	6.24%	6.24%	6.24%	6.24%	6.24%	5.97%	7.01%	7.51%	6.24%	6.24%	6.24%	6.10%	3.95%	4.12%	0.00%	5.66%	5.66%	7.11%	5.66%	5.66%	5.66%	6.24%	5.66%	7.32%	5.66%	5.66%	5.66%	5.88%	6.73%	7.36%	8.10%	5.93%	7.98%	5.66%	7.09%	7.03%	7.76%	9.03%	5.66%	5.81%	8.28%	6.53%	7.95%	8.91%	2.27%	2.48%	0.00%	3.89%	2.47%	2.47%	2.47%	2.55%	2.18%	3.34%	3.19%	2.60%	2.52%	3.16%	3.33%	2.48%	0.00%	0.00%	0.00%	0.00%		0	0	0	0	0	0	0	0	0	0	40	39	37	47	14	36	24	16
69. *S. yagiui* SKS583	6.24%	6.12%	6.10%	6.24%	6.24%	6.24%	6.24%	6.24%	5.97%	7.01%	7.51%	6.24%	6.24%	6.24%	6.10%	3.95%	4.12%	0.00%	5.66%	5.66%	7.11%	5.66%	5.66%	5.66%	6.24%	5.66%	7.32%	5.66%	5.66%	5.66%	5.88%	6.73%	7.36%	8.10%	5.93%	7.98%	5.66%	7.09%	7.03%	7.76%	9.03%	5.66%	5.81%	8.28%	6.53%	7.95%	8.91%	2.27%	2.48%	0.00%	3.89%	2.47%	2.47%	2.47%	2.55%	2.18%	3.34%	3.19%	2.60%	2.52%	3.16%	3.33%	2.48%	0.00%	0.00%	0.00%	0.00%	0.00%		0	0	0	0	0	0	0	0	0	40	39	37	47	14	36	24	16
70. *S. yagiui* SKS977	6.24%	6.12%	6.10%	6.24%	6.24%	6.24%	6.24%	6.24%	5.97%	7.01%	7.51%	6.24%	6.24%	6.24%	6.10%	3.95%	4.12%	0.00%	5.66%	5.66%	7.11%	5.66%	5.66%	5.66%	6.24%	5.66%	7.32%	5.66%	5.66%	5.66%	5.88%	6.73%	7.36%	8.10%	5.93%	7.98%	5.66%	7.09%	7.03%	7.76%	9.03%	5.66%	5.81%	8.28%	6.53%	7.95%	8.91%	2.27%	2.48%	0.00%	3.89%	2.47%	2.47%	2.47%	2.55%	2.18%	3.34%	3.19%	2.60%	2.52%	3.16%	3.33%	2.48%	0.00%	0.00%	0.00%	0.00%	0.00%	0.00%		0	0	0	0	0	0	0	0	40	39	37	47	14	36	24	16
71. *S. yagiui* SKS983	6.24%	6.12%	6.10%	6.24%	6.24%	6.24%	6.24%	6.24%	5.97%	7.01%	7.51%	6.24%	6.24%	6.24%	6.10%	3.95%	4.12%	0.00%	5.66%	5.66%	7.11%	5.66%	5.66%	5.66%	6.24%	5.66%	7.32%	5.66%	5.66%	5.66%	5.88%	6.73%	7.36%	8.10%	5.93%	7.98%	5.66%	7.09%	7.03%	7.76%	9.03%	5.66%	5.81%	8.28%	6.53%	7.95%	8.91%	2.27%	2.48%	0.00%	3.89%	2.47%	2.47%	2.47%	2.55%	2.18%	3.34%	3.19%	2.60%	2.52%	3.16%	3.33%	2.48%	0.00%	0.00%	0.00%	0.00%	0.00%	0.00%	0.00%		0	0	0	0	0	0	0	40	39	37	47	14	36	24	16
72. *S. yagiui* SKS964	6.24%	6.12%	6.10%	6.24%	6.24%	6.24%	6.24%	6.24%	5.97%	7.01%	7.51%	6.24%	6.24%	6.24%	6.10%	3.95%	4.12%	0.00%	5.66%	5.66%	7.11%	5.66%	5.66%	5.66%	6.24%	5.66%	7.32%	5.66%	5.66%	5.66%	5.88%	6.73%	7.36%	8.10%	5.93%	7.98%	5.66%	7.09%	7.03%	7.76%	9.03%	5.66%	5.81%	8.28%	6.53%	7.95%	8.91%	2.27%	2.48%	0.00%	3.89%	2.47%	2.47%	2.47%	2.55%	2.18%	3.34%	3.19%	2.60%	2.52%	3.16%	3.33%	2.48%	0.00%	0.00%	0.00%	0.00%	0.00%	0.00%	0.00%	0.00%		0	0	0	0	0	0	40	39	37	47	14	36	24	16
73. *S. yagiui* SKS787	6.24%	6.12%	6.10%	6.24%	6.24%	6.24%	6.24%	6.24%	5.97%	7.01%	7.51%	6.24%	6.24%	6.24%	6.10%	3.95%	4.12%	0.00%	5.66%	5.66%	7.11%	5.66%	5.66%	5.66%	6.24%	5.66%	7.32%	5.66%	5.66%	5.66%	5.88%	6.73%	7.36%	8.10%	5.93%	7.98%	5.66%	7.09%	7.03%	7.76%	9.03%	5.66%	5.81%	8.28%	6.53%	7.95%	8.91%	2.27%	2.48%	0.00%	3.89%	2.47%	2.47%	2.47%	2.55%	2.18%	3.34%	3.19%	2.60%	2.52%	3.16%	3.33%	2.48%	0.00%	0.00%	0.00%	0.00%	0.00%	0.00%	0.00%	0.00%	0.00%		0	0	0	0	0	40	39	37	47	14	36	24	16
74. *S. yagiui* SKS963	6.24%	6.12%	6.10%	6.24%	6.24%	6.24%	6.24%	6.24%	5.97%	7.01%	7.51%	6.24%	6.24%	6.24%	6.10%	3.95%	4.12%	0.00%	5.66%	5.66%	7.11%	5.66%	5.66%	5.66%	6.24%	5.66%	7.32%	5.66%	5.66%	5.66%	5.88%	6.73%	7.36%	8.10%	5.93%	7.98%	5.66%	7.09%	7.03%	7.76%	9.03%	5.66%	5.81%	8.28%	6.53%	7.95%	8.91%	2.27%	2.48%	0.00%	3.89%	2.47%	2.47%	2.47%	2.55%	2.18%	3.34%	3.19%	2.60%	2.52%	3.16%	3.33%	2.48%	0.00%	0.00%	0.00%	0.00%	0.00%	0.00%	0.00%	0.00%	0.00%	0.00%		0	0	0	0	40	39	37	47	14	36	24	16
75. *S. yagiui* Pozz	5.59%	5.59%	5.59%	5.88%	5.59%	5.59%	5.59%	5.59%	5.33%	5.29%	5.29%	5.59%	5.59%	5.59%	5.59%	3.83%	4.13%	0.00%	4.71%	4.71%	4.71%	4.71%	4.71%	4.71%	5.18%	4.71%	6.18%	4.71%	4.71%	4.71%	6.18%	6.18%	4.71%	6.18%	6.18%	6.18%	4.71%	4.71%	4.71%	4.71%	4.75%	4.71%	4.41%	6.18%	6.18%	5.90%	5.90%	2.65%	2.65%	0.00%	2.94%	2.65%	2.65%	2.65%	2.35%	2.35%	3.24%	2.94%	2.91%	2.06%	2.65%	2.65%	2.06%	0.00%	0.00%	0.00%	0.00%	0.00%	0.00%	0.00%	0.00%	0.00%	0.00%	0.00%		0	0	0	24	21	18	20	7	18	21	9
76. *S. yagiui* LamCyp	7.98%	7.98%	7.98%	8.18%	7.98%	7.98%	7.98%	7.98%	7.80%	7.01%	7.77%	7.98%	7.98%	7.98%	7.98%	5.33%	5.74%	0.00%	7.36%	7.36%	7.36%	7.36%	7.36%	7.36%	7.86%	7.36%	8.38%	7.36%	7.36%	7.36%	5.88%	6.73%	7.36%	8.38%	5.93%	7.98%	7.36%	7.36%	7.36%	7.76%	9.03%	7.36%	7.36%	8.59%	8.59%	7.95%	8.91%	2.27%	2.48%	0.00%	3.89%	3.27%	3.27%	3.27%	2.66%	2.66%	4.50%	4.29%	3.49%	2.52%	3.27%	3.33%	2.48%	0.00%	0.00%	0.00%	0.00%	0.00%	0.00%	0.00%	0.00%	0.00%	0.00%	0.00%	0.00%		0	0	40	39	37	44	14	33	24	16
77. *S. yagiui* SpirWS	7.98%	7.98%	7.98%	8.18%	7.98%	7.98%	7.98%	7.98%	7.80%	7.01%	7.77%	7.98%	7.98%	7.98%	7.98%	5.33%	5.74%	0.00%	7.36%	7.36%	7.36%	7.36%	7.36%	7.36%	7.86%	7.36%	8.38%	7.36%	7.36%	7.36%	5.88%	6.73%	7.36%	8.38%	5.93%	7.98%	7.36%	7.36%	7.36%	7.76%	9.03%	7.36%	7.36%	8.59%	8.59%	7.95%	8.91%	2.27%	2.48%	0.00%	3.89%	3.27%	3.27%	3.27%	2.66%	2.66%	4.50%	4.29%	3.49%	2.52%	3.27%	3.33%	2.48%	0.00%	0.00%	0.00%	0.00%	0.00%	0.00%	0.00%	0.00%	0.00%	0.00%	0.00%	0.00%	0.00%		0	40	39	37	44	14	33	24	16
78. *S. yagiui* GNS4	5.93%	5.93%	5.93%	6.21%	5.93%	5.93%	5.93%	5.93%	5.68%	5.65%	5.65%	5.93%	5.93%	5.93%	5.93%	4.25%	4.82%	0.00%	4.52%	4.52%	4.52%	4.52%	4.52%	4.52%	4.95%	4.52%	5.93%	4.52%	4.52%	4.52%	5.93%	5.93%	4.52%	5.93%	5.93%	5.93%	4.52%	4.52%	4.52%	4.52%	4.84%	4.52%	4.24%	5.93%	5.93%	5.67%	5.67%	2.54%	2.54%	0.00%	2.82%	2.54%	2.54%	2.54%	2.26%	2.26%	3.11%	2.82%	2.79%	2.26%	2.54%	2.54%	2.26%	0.00%	0.00%	0.00%	0.00%	0.00%	0.00%	0.00%	0.00%	0.00%	0.00%	0.00%	0.00%	0.00%	0.00%		24	21	20	22	8	18	21	9
79. *S.* sp. Ind	5.70%	5.26%	5.26%	5.48%	5.70%	5.70%	5.70%	5.70%	5.07%	4.85%	5.48%	5.70%	5.70%	5.70%	5.26%	9.89%	9.45%	7.06%	4.82%	4.82%	4.82%	4.82%	4.82%	4.82%	5.18%	4.82%	1.97%	4.82%	4.82%	4.82%	1.10%	1.90%	4.82%	1.97%	1.11%	1.54%	4.82%	4.82%	4.82%	4.82%	5.97%	4.82%	4.92%	6.58%	6.58%	6.52%	6.61%	6.02%	6.65%	8.93%	9.98%	9.71%	9.71%	9.71%	10.15%	10.15%	9.71%	9.49%	10.43%	7.28%	9.71%	9.71%	7.16%	8.87%	8.87%	8.87%	8.87%	8.87%	8.87%	8.87%	8.87%	8.87%	8.87%	8.87%	7.06%	8.87%	8.87%	6.78%		9	25	29	35	29	5	46
80. *S.* sp. Thd2	5.70%	5.26%	5.26%	5.48%	5.70%	5.70%	5.70%	5.70%	5.07%	4.85%	5.48%	5.70%	5.70%	5.70%	5.26%	10.11%	10.11%	6.18%	5.26%	5.26%	5.26%	5.26%	5.26%	5.26%	5.65%	5.26%	0.00%	5.26%	5.26%	5.26%	0.00%	0.00%	5.26%	0.00%	0.00%	1.10%	5.26%	5.26%	5.26%	5.26%	6.42%	5.26%	5.31%	7.02%	7.02%	6.74%	6.83%	5.26%	6.40%	8.71%	9.98%	9.93%	9.93%	9.93%	9.93%	9.93%	9.71%	9.49%	10.66%	6.44%	9.93%	9.93%	6.34%	8.65%	8.65%	8.65%	8.65%	8.65%	8.65%	8.65%	8.65%	8.65%	8.65%	8.65%	6.18%	8.65%	8.65%	5.93%	1.70%		25	29	33	33	1	47
81. *S.* sp. Seef2	0.22%	0.22%	0.22%	0.44%	0.22%	0.22%	0.22%	0.22%	0.44%	0.00%	0.00%	0.22%	0.22%	0.22%	0.22%	8.81%	8.81%	5.29%	4.62%	4.62%	4.62%	4.62%	4.62%	4.62%	4.95%	4.62%	5.49%	4.62%	4.62%	4.62%	3.87%	4.75%	4.62%	5.49%	3.90%	5.27%	4.62%	4.62%	4.62%	4.62%	5.76%	4.62%	4.34%	4.62%	4.62%	4.49%	4.86%	6.02%	6.90%	8.26%	9.56%	9.51%	9.51%	9.51%	9.73%	9.73%	9.96%	9.73%	10.21%	6.16%	9.51%	9.51%	6.06%	8.22%	8.22%	8.22%	8.22%	8.22%	8.22%	8.22%	8.22%	8.22%	8.22%	8.22%	5.29%	8.22%	8.22%	5.65%	4.74%	4.74%		6	32	26	15	45
82. *S.* sp. GuaCHN	1.25%	0.78%	0.78%	0.94%	1.25%	1.25%	1.25%	1.25%	0.94%	1.39%	1.17%	1.25%	1.25%	1.25%	0.78%	7.81%	7.66%	5.88%	4.21%	4.21%	4.89%	4.21%	4.21%	4.21%	4.43%	4.21%	5.35%	4.21%	4.21%	4.21%	3.87%	5.46%	5.06%	5.87%	3.90%	5.87%	4.21%	4.87%	4.84%	5.48%	6.60%	4.21%	4.49%	4.49%	3.90%	4.94%	5.29%	6.77%	7.64%	9.60%	9.82%	7.84%	7.84%	7.84%	9.57%	8.31%	8.62%	8.46%	8.24%	6.72%	9.25%	10.38%	6.89%	7.39%	7.39%	7.39%	7.39%	7.39%	7.39%	7.39%	7.39%	7.39%	7.39%	7.39%	5.88%	9.00%	9.00%	6.21%	5.73%	5.73%	1.19%		36	32	18	48
83. *S.* sp. StMgN	7.42%	7.42%	7.42%	7.66%	7.42%	7.42%	7.42%	7.42%	7.21%	7.18%	7.18%	7.42%	7.42%	7.42%	7.42%	5.52%	6.71%	2.06%	6.22%	6.22%	6.22%	6.22%	6.22%	6.22%	6.72%	6.22%	7.42%	6.22%	6.22%	6.22%	6.44%	7.45%	6.22%	7.42%	6.21%	7.42%	6.22%	6.22%	6.22%	6.22%	6.76%	6.22%	5.74%	7.42%	7.42%	7.43%	7.21%	4.28%	4.95%	3.36%	4.08%	5.26%	5.26%	5.26%	5.02%	5.02%	3.35%	3.11%	5.68%	1.40%	5.26%	5.26%	1.38%	3.36%	3.36%	3.36%	3.36%	3.36%	3.36%	3.36%	3.36%	3.36%	3.36%	3.36%	2.06%	3.36%	3.36%	2.27%	7.14%	6.73%	6.53%	7.69%		29	29	22
84. *S.* sp. SKS255	4.06%	3.92%	3.92%	4.06%	4.06%	4.06%	4.06%	4.06%	3.78%	5.54%	5.09%	4.06%	4.06%	4.06%	3.92%	6.25%	5.99%	5.29%	1.60%	1.60%	1.76%	1.60%	1.60%	1.60%	1.75%	1.60%	5.87%	1.60%	1.60%	1.60%	4.70%	5.46%	1.82%	6.65%	4.74%	5.87%	1.60%	1.75%	1.74%	1.97%	3.40%	1.60%	2.02%	5.86%	4.64%	6.74%	7.05%	4.26%	5.17%	7.14%	7.77%	5.25%	5.25%	5.25%	6.84%	5.69%	5.83%	5.69%	5.50%	5.32%	6.50%	7.28%	5.23%	5.26%	5.26%	5.26%	5.26%	5.26%	5.26%	5.26%	5.26%	5.26%	5.26%	5.26%	5.29%	6.75%	6.75%	5.08%	5.49%	6.25%	4.93%	4.63%	5.92%		18	35
85. *S.* sp. SmJFp1	3.91%	3.42%	3.42%	3.67%	3.91%	3.91%	3.91%	3.91%	3.19%	3.67%	3.67%	3.91%	3.91%	3.91%	3.42%	8.09%	8.09%	6.18%	3.91%	3.91%	3.91%	3.91%	3.91%	3.91%	4.23%	3.91%	0.24%	3.91%	3.91%	3.91%	0.00%	0.24%	3.91%	0.24%	0.00%	0.24%	3.91%	3.91%	3.91%	3.91%	4.44%	3.91%	4.12%	4.40%	4.40%	4.41%	4.18%	5.26%	6.16%	5.94%	6.93%	6.16%	6.16%	6.16%	6.16%	6.16%	6.65%	6.40%	6.67%	6.44%	6.16%	6.16%	6.34%	5.94%	5.94%	5.94%	5.94%	5.94%	5.94%	5.94%	5.94%	5.94%	5.94%	5.94%	6.18%	5.94%	5.94%	5.93%	1.04%	0.21%	3.12%	3.92%	6.09%	3.74%		27
86. *S.* sp. StJFp2	8.96%	8.96%	8.96%	9.16%	8.96%	8.96%	8.96%	8.96%	8.79%	7.91%	8.76%	8.96%	8.96%	8.96%	8.96%	5.71%	6.33%	2.65%	7.33%	7.33%	7.33%	7.33%	7.33%	7.33%	7.83%	7.33%	9.37%	7.33%	7.33%	7.33%	5.85%	6.94%	7.33%	9.37%	5.90%	8.76%	7.33%	7.33%	7.33%	7.95%	9.21%	7.33%	6.81%	9.16%	9.16%	9.28%	10.20%	0.00%	0.00%	3.35%	4.70%	0.00%	0.00%	0.00%	1.02%	1.02%	4.48%	4.28%	0.00%	3.64%	0.00%	0.00%	3.58%	3.27%	3.27%	3.27%	3.27%	3.27%	3.27%	3.27%	3.27%	3.27%	3.27%	3.27%	2.65%	3.27%	3.27%	2.54%	8.76%	8.95%	8.59%	8.87%	4.49%	6.22%	5.65%	

Supplementary Table S6. Numbers of nucleotide differences (above diagonal) and pairwise distances (%, below diagonal) of the 18S rRNA gene sequences among 37 *Spirostomum* populations.

Taxon	1	2	3	4	5	6	7	8	9	10	11	12	13	14	15	16	17	18	19	20	21	22	23	24	25	26	27	28	29	30	31	32	33	34	35	36	37
1. *S. ambiguum* SKS167		0	4	4	4	4	4	4	4	44	21	22	22	22	22	22	22	22	22	22	22	22	22	22	33	33	40	40	38	38	40	38	38	36	36	36	36
2. *S. ambiguum* SKS254	0.00		4	4	4	4	4	4	4	44	21	22	22	22	22	22	22	22	22	22	22	22	22	22	33	33	40	40	38	38	40	38	38	36	36	36	36
3. *S. ambiguum* SKS779	0.25	0.25		2	2	2	2	2	2	44	19	20	20	20	20	20	20	20	20	20	20	20	20	20	31	31	40	38	36	36	38	36	36	34	34	34	34
4. *S. ambiguum* SKS184	0.25	0.25	0.13		0	0	0	0	0	42	21	22	22	22	22	22	22	22	22	22	22	22	22	22	33	33	40	40	38	38	40	38	38	36	36	36	36
5. *S. ambiguum* SKS185	0.25	0.25	0.13	0.00		0	0	0	0	42	21	22	22	22	22	22	22	22	22	22	22	22	22	22	33	33	40	40	38	38	40	38	38	36	36	36	36
6. *S. ambiguum* SKS989	0.25	0.25	0.13	0.00	0.00		0	0	0	42	21	22	22	22	22	22	22	22	22	22	22	22	22	22	33	33	40	40	38	38	40	38	38	36	36	36	36
7. *S. ambiguum* SKS363	0.25	0.25	0.13	0.00	0.00	0.00		0	0	42	21	22	22	22	22	22	22	22	22	22	22	22	22	22	33	33	40	40	38	38	40	38	38	36	36	36	36
8. *S. ambiguum* SKS947	0.25	0.25	0.13	0.00	0.00	0.00	0.00		0	42	21	22	22	22	22	22	22	22	22	22	22	22	22	22	33	33	40	40	38	38	40	38	38	36	36	36	36
9. *S. ambiguum* SKS949	0.25	0.25	0.13	0.00	0.00	0.00	0.00	0.00		42	21	22	22	22	22	22	22	22	22	22	22	22	22	22	33	33	40	40	38	38	40	38	38	36	36	36	36
10. *S. caudatum* SKS302	2.79	2.79	2.79	2.66	2.66	2.66	2.66	2.66	2.66		36	36	36	36	36	36	36	36	36	36	36	36	36	36	52	52	24	28	26	26	28	26	26	28	28	28	28
11. *S. minus* SKS731	1.33	1.33	1.20	1.33	1.33	1.33	1.33	1.33	1.33	2.28		1	1	1	1	1	1	1	1	1	1	1	1	1	24	24	32	26	22	22	26	22	22	23	23	23	23
12. *S. minus* SKS794	1.40	1.40	1.27	1.40	1.40	1.40	1.40	1.40	1.40	2.28	0.06		0	0	0	0	0	0	0	0	0	0	0	0	25	25	31	25	21	21	25	21	21	22	22	22	22
13. *S. minus* SKS939	1.40	1.40	1.27	1.40	1.40	1.40	1.40	1.40	1.40	2.28	0.06	0.00		0	0	0	0	0	0	0	0	0	0	0	25	25	31	25	21	21	25	21	21	22	22	22	22
14. *S. minus* SKS1044	1.40	1.40	1.27	1.40	1.40	1.40	1.40	1.40	1.40	2.28	0.06	0.00	0.00		0	0	0	0	0	0	0	0	0	0	25	25	31	25	21	21	25	21	21	22	22	22	22
15. *S. minus* GuS1058	1.40	1.40	1.27	1.40	1.40	1.40	1.40	1.40	1.40	2.28	0.06	0.00	0.00	0.00		0	0	0	0	0	0	0	0	0	25	25	31	25	21	21	25	21	21	22	22	22	22
16. *S. minus* SKS215	1.40	1.40	1.27	1.40	1.40	1.40	1.40	1.40	1.40	2.28	0.06	0.00	0.00	0.00	0.00		0	0	0	0	0	0	0	0	25	25	31	25	21	21	25	21	21	22	22	22	22
17. *S. minus* SKS214	1.40	1.40	1.27	1.40	1.40	1.40	1.40	1.40	1.40	2.28	0.06	0.00	0.00	0.00	0.00	0.00		0	0	0	0	0	0	0	25	25	31	25	21	21	25	21	21	22	22	22	22
18. *S. minus* SKS182	1.40	1.40	1.27	1.40	1.40	1.40	1.40	1.40	1.40	2.28	0.06	0.00	0.00	0.00	0.00	0.00	0.00		0	0	0	0	0	0	25	25	31	25	21	21	25	21	21	22	22	22	22
19. *S. minus* SKS840	1.40	1.40	1.27	1.40	1.40	1.40	1.40	1.40	1.40	2.28	0.06	0.00	0.00	0.00	0.00	0.00	0.00	0.00		0	0	0	0	0	25	25	31	25	21	21	25	21	21	22	22	22	22
20. *S. minus* SKS386	1.40	1.40	1.27	1.40	1.40	1.40	1.40	1.40	1.40	2.28	0.06	0.00	0.00	0.00	0.00	0.00	0.00	0.00	0.00		0	0	0	0	25	25	31	25	21	21	25	21	21	22	22	22	22
21. *S. minus* SKS1073	1.40	1.40	1.27	1.40	1.40	1.40	1.40	1.40	1.40	2.28	0.06	0.00	0.00	0.00	0.00	0.00	0.00	0.00	0.00	0.00		0	0	0	25	25	31	25	21	21	25	21	21	22	22	22	22
22. *S. minus* SKS1018	1.40	1.40	1.27	1.40	1.40	1.40	1.40	1.40	1.40	2.28	0.06	0.00	0.00	0.00	0.00	0.00	0.00	0.00	0.00	0.00	0.00		0	0	25	25	31	25	21	21	25	21	21	22	22	22	22
23. *S. minus* SKS743	1.40	1.40	1.27	1.40	1.40	1.40	1.40	1.40	1.40	2.28	0.06	0.00	0.00	0.00	0.00	0.00	0.00	0.00	0.00	0.00	0.00	0.00		0	25	25	31	25	21	21	25	21	21	22	22	22	22
24. *S. minus* SKS161	1.40	1.40	1.27	1.40	1.40	1.40	1.40	1.40	1.40	2.28	0.06	0.00	0.00	0.00	0.00	0.00	0.00	0.00	0.00	0.00	0.00	0.00	0.00		25	25	31	25	21	21	25	21	21	22	22	22	22
25. S. subtilis SKS937	2.09	2.09	1.97	2.09	2.09	2.09	2.09	2.09	2.09	3.30	1.52	1.59	1.59	1.59	1.59	1.59	1.59	1.59	1.59	1.59	1.59	1.59	1.59	1.59		0	45	38	38	38	38	38	38	35	35	35	35
26. *S. subtilis* SKS1056	2.09	2.09	1.97	2.09	2.09	2.09	2.09	2.09	2.09	3.30	1.52	1.59	1.59	1.59	1.59	1.59	1.59	1.59	1.59	1.59	1.59	1.59	1.59	1.59	0.00		45	38	38	38	38	38	38	35	35	35	35
27. *S. teres* SKS1045	2.54	2.54	2.54	2.54	2.54	2.54	2.54	2.54	2.54	1.52	2.03	1.97	1.97	1.97	1.97	1.97	1.97	1.97	1.97	1.97	1.97	1.97	1.97	1.97	2.85	2.85		11	12	12	11	12	12	21	21	21	21
28. *S. teres* SKS026	2.54	2.54	2.41	2.54	2.54	2.54	2.54	2.54	2.54	1.78	1.65	1.59	1.59	1.59	1.59	1.59	1.59	1.59	1.59	1.59	1.59	1.59	1.59	1.59	2.41	2.41	0.70		4	4	0	4	4	13	13	13	13
29. *S. teres* SKS187	2.41	2.41	2.28	2.41	2.41	2.41	2.41	2.41	2.41	1.65	1.40	1.33	1.33	1.33	1.33	1.33	1.33	1.33	1.33	1.33	1.33	1.33	1.33	1.33	2.41	2.41	0.76	0.25		0	4	0	0	11	11	11	11
30. *S. teres* SKS085	2.41	2.41	2.28	2.41	2.41	2.41	2.41	2.41	2.41	1.65	1.40	1.33	1.33	1.33	1.33	1.33	1.33	1.33	1.33	1.33	1.33	1.33	1.33	1.33	2.41	2.41	0.76	0.25	0.00		4	0	0	11	11	11	11
31. *S. teres* SKS351	2.54	2.54	2.41	2.54	2.54	2.54	2.54	2.54	2.54	1.78	1.65	1.59	1.59	1.59	1.59	1.59	1.59	1.59	1.59	1.59	1.59	1.59	1.59	1.59	2.41	2.41	0.70	0.00	0.25	0.25		4	4	13	13	13	13
32. *S. teres* SKS107	2.41	2.41	2.28	2.41	2.41	2.41	2.41	2.41	2.41	1.65	1.40	1.33	1.33	1.33	1.33	1.33	1.33	1.33	1.33	1.33	1.33	1.33	1.33	1.33	2.41	2.41	0.76	0.25	0.00	0.00	0.25		0	11	11	11	11
33. *S. teres* SKS988	2.41	2.41	2.28	2.41	2.41	2.41	2.41	2.41	2.41	1.65	1.40	1.33	1.33	1.33	1.33	1.33	1.33	1.33	1.33	1.33	1.33	1.33	1.33	1.33	2.41	2.41	0.76	0.25	0.00	0.00	0.25	0.00		11	11	11	11
34. *S. yagiui* SKS977	2.28	2.28	2.16	2.28	2.28	2.28	2.28	2.28	2.28	1.78	1.46	1.40	1.40	1.40	1.40	1.40	1.40	1.40	1.40	1.40	1.40	1.40	1.40	1.40	2.22	2.22	1.33	0.82	0.70	0.70	0.82	0.70	0.70		0	0	0
35. *S. yagiui* SKS787	2.28	2.28	2.16	2.28	2.28	2.28	2.28	2.28	2.28	1.78	1.46	1.40	1.40	1.40	1.40	1.40	1.40	1.40	1.40	1.40	1.40	1.40	1.40	1.40	2.22	2.22	1.33	0.82	0.70	0.70	0.82	0.70	0.70	0.00		0	0
36. *S. yagiui* SKS164	2.28	2.28	2.16	2.28	2.28	2.28	2.28	2.28	2.28	1.78	1.46	1.40	1.40	1.40	1.40	1.40	1.40	1.40	1.40	1.40	1.40	1.40	1.40	1.40	2.22	2.22	1.33	0.82	0.70	0.70	0.82	0.70	0.70	0.00	0.00		0
37. *S. yagiui* SKS071	2.28	2.28	2.16	2.28	2.28	2.28	2.28	2.28	2.28	1.78	1.46	1.40	1.40	1.40	1.40	1.40	1.40	1.40	1.40	1.40	1.40	1.40	1.40	1.40	2.22	2.22	1.33	0.82	0.70	0.70	0.82	0.70	0.70	0.00	0.00	0.00	

Supplementary Table S7. Numbers of nucleotide differences (above diagonal) and pairwise distances (%, below diagonal) of the ITS regions among 37 *Spirostomum* populations.

Taxon	1	2	3	4	5	6	7	8	9	10	11	12	13	14	15	16	17	18	19	20	21	22	23	24	25	26	27	28	29	30	31	32	33	34	35	36	37
1. *S. ambiguum* SKS167		0	0	0	0	0	0	0	0	25	11	11	11	11	11	11	11	11	11	11	12	9	10	10	3	3	19	21	19	23	21	21	21	29	29	29	29
2. *S. ambiguum* SKS184	0.00		0	0	0	0	0	0	0	25	11	11	11	11	11	11	11	11	11	11	12	9	10	10	3	3	19	21	19	23	21	21	21	29	29	29	29
3. *S. ambiguum* SKS185	0.00	0.00		0	0	0	0	0	0	25	11	11	11	11	11	11	11	11	11	11	12	9	10	10	3	3	19	21	19	23	21	21	21	29	29	29	29
4. *S. ambiguum* SKS949	0.00	0.00	0.00		0	0	0	0	0	25	11	11	11	11	11	11	11	11	11	11	12	9	10	10	3	3	19	21	19	23	21	21	21	29	29	29	29
5. *S. ambiguum* SKS989	0.00	0.00	0.00	0.00		0	0	0	0	25	11	11	11	11	11	11	11	11	11	11	12	9	10	10	3	3	19	21	19	23	21	21	21	29	29	29	29
6. *S. ambiguum* SKS363	0.00	0.00	0.00	0.00	0.00		0	0	0	25	11	11	11	11	11	11	11	11	11	11	12	9	10	10	3	3	19	21	19	23	21	21	21	29	29	29	29
7. *S. ambiguum* SKS947	0.00	0.00	0.00	0.00	0.00	0.00		0	0	25	11	11	11	11	11	11	11	11	11	11	12	9	10	10	3	3	19	21	19	23	21	21	21	29	29	29	29
8. *S. ambiguum* SKS779	0.00	0.00	0.00	0.00	0.00	0.00	0.00		0	25	11	11	11	11	11	11	11	11	11	11	12	9	10	10	3	3	19	21	19	23	21	21	21	29	29	29	29
9. *S. ambiguum* SKS254	0.00	0.00	0.00	0.00	0.00	0.00	0.00	0.00		25	11	11	11	11	11	11	11	11	11	11	12	9	10	10	3	3	19	21	19	23	21	21	21	29	29	29	29
10. *S. caudatum* SKS302	10.68	10.68	10.68	10.68	10.68	10.68	10.68	10.68	10.68		31	31	31	31	31	31	31	31	31	31	30	29	30	30	25	25	19	20	19	19	20	20	20	24	24	24	24
11. *S. minus* SKS161	4.70	4.70	4.70	4.70	4.70	4.70	4.70	4.70	4.70	13.25		0	0	0	0	0	0	0	0	0	1	2	1	1	12	12	25	27	25	27	27	27	27	32	32	32	32
12. *S. minus* SKS182	4.70	4.70	4.70	4.70	4.70	4.70	4.70	4.70	4.70	13.25	0.00		0	0	0	0	0	0	0	0	1	2	1	1	12	12	25	27	25	27	27	27	27	32	32	32	32
13. *S. minus* SKS214	4.70	4.70	4.70	4.70	4.70	4.70	4.70	4.70	4.70	13.25	0.00	0.00		0	0	0	0	0	0	0	1	2	1	1	12	12	25	27	25	27	27	27	27	32	32	32	32
14. *S. minus* SKS215	4.70	4.70	4.70	4.70	4.70	4.70	4.70	4.70	4.70	13.25	0.00	0.00	0.00		0	0	0	0	0	0	1	2	1	1	12	12	25	27	25	27	27	27	27	32	32	32	32
15. *S. minus* SKS386	4.70	4.70	4.70	4.70	4.70	4.70	4.70	4.70	4.70	13.25	0.00	0.00	0.00	0.00		0	0	0	0	0	1	2	1	1	12	12	25	27	25	27	27	27	27	32	32	32	32
16. *S. minus* SKS743	4.70	4.70	4.70	4.70	4.70	4.70	4.70	4.70	4.70	13.25	0.00	0.00	0.00	0.00	0.00		0	0	0	0	1	2	1	1	12	12	25	27	25	27	27	27	27	32	32	32	32
17. *S. minus* SKS840	4.70	4.70	4.70	4.70	4.70	4.70	4.70	4.70	4.70	13.25	0.00	0.00	0.00	0.00	0.00	0.00		0	0	0	1	2	1	1	12	12	25	27	25	27	27	27	27	32	32	32	32
18. *S. minus* SKS1018	4.70	4.70	4.70	4.70	4.70	4.70	4.70	4.70	4.70	13.25	0.00	0.00	0.00	0.00	0.00	0.00	0.00		0	0	1	2	1	1	12	12	25	27	25	27	27	27	27	32	32	32	32
19. *S. minus* SKS1044	4.70	4.70	4.70	4.70	4.70	4.70	4.70	4.70	4.70	13.25	0.00	0.00	0.00	0.00	0.00	0.00	0.00	0.00		0	1	2	1	1	12	12	25	27	25	27	27	27	27	32	32	32	32
20. *S. minus* SKS1073	4.70	4.70	4.70	4.70	4.70	4.70	4.70	4.70	4.70	13.25	0.00	0.00	0.00	0.00	0.00	0.00	0.00	0.00	0.00		1	2	1	1	12	12	25	27	25	27	27	27	27	32	32	32	32
21. *S. minus* SKS731	5.13	5.13	5.13	5.13	5.13	5.13	5.13	5.13	5.13	12.82	0.43	0.43	0.43	0.43	0.43	0.43	0.43	0.43	0.43	0.43		3	2	2	13	13	25	27	25	26	27	27	27	31	31	31	31
22. *S. minus* GuS1058	3.85	3.85	3.85	3.85	3.85	3.85	3.85	3.85	3.85	12.39	0.85	0.85	0.85	0.85	0.85	0.85	0.85	0.85	0.85	0.85	1.28		1	1	10	10	23	25	23	26	25	25	25	30	30	30	30
23. *S. minus* SKS794	4.27	4.27	4.27	4.27	4.27	4.27	4.27	4.27	4.27	12.82	0.43	0.43	0.43	0.43	0.43	0.43	0.43	0.43	0.43	0.43	0.85	0.43		0	11	11	24	26	24	26	26	26	26	31	31	31	31
24. *S. minus* SKS939	4.27	4.27	4.27	4.27	4.27	4.27	4.27	4.27	4.27	12.82	0.43	0.43	0.43	0.43	0.43	0.43	0.43	0.43	0.43	0.43	0.85	0.43	0.00		11	11	24	26	24	26	26	26	26	31	31	31	31
25. *S. subtilis* SKS937	1.28	1.28	1.28	1.28	1.28	1.28	1.28	1.28	1.28	10.68	5.13	5.13	5.13	5.13	5.13	5.13	5.13	5.13	5.13	5.13	5.56	4.27	4.70	4.70		0	19	21	19	23	21	21	21	29	29	29	29
26. *S. subtilis* SKS1056	1.28	1.28	1.28	1.28	1.28	1.28	1.28	1.28	1.28	10.68	5.13	5.13	5.13	5.13	5.13	5.13	5.13	5.13	5.13	5.13	5.56	4.27	4.70	4.70	0.00		19	21	19	23	21	21	21	29	29	29	29
27. *S. teres* SKS351	8.12	8.12	8.12	8.12	8.12	8.12	8.12	8.12	8.12	8.12	10.68	10.68	10.68	10.68	10.68	10.68	10.68	10.68	10.68	10.68	10.68	9.83	10.26	10.26	8.12	8.12		3	0	14	3	3	3	14	14	14	14
28. *S. teres* SKS107	8.97	8.97	8.97	8.97	8.97	8.97	8.97	8.97	8.97	8.55	11.54	11.54	11.54	11.54	11.54	11.54	11.54	11.54	11.54	11.54	11.54	10.68	11.11	11.11	8.97	8.97	1.28		3	13	0	0	0	15	15	15	15
29. *S. teres* SKS026	8.12	8.12	8.12	8.12	8.12	8.12	8.12	8.12	8.12	8.12	10.68	10.68	10.68	10.68	10.68	10.68	10.68	10.68	10.68	10.68	10.68	9.83	10.26	10.26	8.12	8.12	0.00	1.28		14	3	3	3	14	14	14	14
30. *S. teres* SKS1045	9.83	9.83	9.83	9.83	9.83	9.83	9.83	9.83	9.83	8.12	11.54	11.54	11.54	11.54	11.54	11.54	11.54	11.54	11.54	11.54	11.11	11.11	11.11	11.11	9.83	9.83	5.98	5.56	5.98		13	13	13	15	15	15	15
31. *S. teres* SKS187	8.97	8.97	8.97	8.97	8.97	8.97	8.97	8.97	8.97	8.55	11.54	11.54	11.54	11.54	11.54	11.54	11.54	11.54	11.54	11.54	11.54	10.68	11.11	11.11	8.97	8.97	1.28	0.00	1.28	5.56		0	0	15	15	15	15
32. *S. teres* SKS085	8.97	8.97	8.97	8.97	8.97	8.97	8.97	8.97	8.97	8.55	11.54	11.54	11.54	11.54	11.54	11.54	11.54	11.54	11.54	11.54	11.54	10.68	11.11	11.11	8.97	8.97	1.28	0.00	1.28	5.56	0.00		0	15	15	15	15
33. *S. teres* SKS988	8.97	8.97	8.97	8.97	8.97	8.97	8.97	8.97	8.97	8.55	11.54	11.54	11.54	11.54	11.54	11.54	11.54	11.54	11.54	11.54	11.54	10.68	11.11	11.11	8.97	8.97	1.28	0.00	1.28	5.56	0.00	0.00		15	15	15	15
34. *S. yagiui* SKS071	12.39	12.39	12.39	12.39	12.39	12.39	12.39	12.39	12.39	10.26	13.68	13.68	13.68	13.68	13.68	13.68	13.68	13.68	13.68	13.68	13.25	12.82	13.25	13.25	12.39	12.39	5.98	6.41	5.98	6.41	6.41	6.41	6.41		0	0	0
35. *S. yagiui* SKS164	12.39	12.39	12.39	12.39	12.39	12.39	12.39	12.39	12.39	10.26	13.68	13.68	13.68	13.68	13.68	13.68	13.68	13.68	13.68	13.68	13.25	12.82	13.25	13.25	12.39	12.39	5.98	6.41	5.98	6.41	6.41	6.41	6.41	0.00		0	0
36. *S. yagiui* SKS787	12.39	12.39	12.39	12.39	12.39	12.39	12.39	12.39	12.39	10.26	13.68	13.68	13.68	13.68	13.68	13.68	13.68	13.68	13.68	13.68	13.25	12.82	13.25	13.25	12.39	12.39	5.98	6.41	5.98	6.41	6.41	6.41	6.41	0.00	0.00		0
37. *S. yagiui* SKS977	12.39	12.39	12.39	12.39	12.39	12.39	12.39	12.39	12.39	10.26	13.68	13.68	13.68	13.68	13.68	13.68	13.68	13.68	13.68	13.68	13.25	12.82	13.25	13.25	12.39	12.39	5.98	6.41	5.98	6.41	6.41	6.41	6.41	0.00	0.00	0.00	

Supplementary Table S8. Numbers of nucleotide differences (above diagonal) and pairwise distances (%, below diagonal) of the D1D2 domain of the 28S rRNA gene sequences among 37 *Spirostomum* populations.

Taxon	1	2	3	4	5	6	7	8	9	10	11	12	13	14	15	16	17	18	19	20	21	22	23	24	25	26	27	28	29	30	31	32	33	34	35	36	37
1. *S. ambiguum* SKS185		0	0	0	2	2	1	0	0	39	18	18	18	18	18	18	18	18	18	18	18	18	18	18	22	22	45	45	43	43	43	43	44	39	39	39	39
2. *S. ambiguum* SKS184	0.00		0	0	2	2	1	0	0	39	18	18	18	18	18	18	18	18	18	18	18	18	18	18	22	22	45	45	43	43	43	43	44	39	39	39	39
3. *S. ambiguum* SKS947	0.00	0.00		0	2	2	1	0	0	39	18	18	18	18	18	18	18	18	18	18	18	18	18	18	22	22	45	45	43	43	43	43	44	39	39	39	39
4. *S. ambiguum* SKS989	0.00	0.00	0.00		2	2	1	0	0	39	18	18	18	18	18	18	18	18	18	18	18	18	18	18	22	22	45	45	43	43	43	43	44	39	39	39	39
5. *S. ambiguum* SKS167	0.42	0.42	0.42	0.42		0	1	2	2	39	16	16	16	16	16	16	16	16	16	16	16	16	16	16	20	20	45	45	43	43	43	43	44	39	39	39	39
6. *S. ambiguum* SKS254	0.42	0.42	0.42	0.42	0.00		1	2	2	39	16	16	16	16	16	16	16	16	16	16	16	16	16	16	20	20	45	45	43	43	43	43	44	39	39	39	39
7. *S. ambiguum* SKS779	0.21	0.21	0.21	0.21	0.21	0.21		1	1	40	17	17	17	17	17	17	17	17	17	17	17	17	17	17	21	21	46	46	44	44	44	44	45	40	40	40	40
8. *S. ambiguum* SKS949	0.00	0.00	0.00	0.00	0.42	0.42	0.21		0	39	18	18	18	18	18	18	18	18	18	18	18	18	18	18	22	22	45	45	43	43	43	43	44	39	39	39	39
9. *S. ambiguum* SKS363	0.00	0.00	0.00	0.00	0.42	0.42	0.21	0.00		39	18	18	18	18	18	18	18	18	18	18	18	18	18	18	22	22	45	45	43	43	43	43	44	39	39	39	39
10. *S. caudatum* SKS302	8.21	8.21	8.21	8.21	8.21	8.21	8.42	8.21	8.21		39	39	39	39	39	39	39	39	39	39	39	39	39	39	42	42	36	36	33	33	33	33	37	30	30	30	30
11. *S. minus* SKS939	3.79	3.79	3.79	3.79	3.37	3.37	3.58	3.79	3.79	8.21		0	0	0	0	0	0	0	0	0	0	0	0	0	22	22	36	36	34	34	34	34	36	36	36	36	36
12. *S. minus* SKS161	3.79	3.79	3.79	3.79	3.37	3.37	3.58	3.79	3.79	8.21	0.00		0	0	0	0	0	0	0	0	0	0	0	0	22	22	36	36	34	34	34	34	36	36	36	36	36
13. *S. minus* SKS182	3.79	3.79	3.79	3.79	3.37	3.37	3.58	3.79	3.79	8.21	0.00	0.00		0	0	0	0	0	0	0	0	0	0	0	22	22	36	36	34	34	34	34	36	36	36	36	36
14. *S. minus* SKS214	3.79	3.79	3.79	3.79	3.37	3.37	3.58	3.79	3.79	8.21	0.00	0.00	0.00		0	0	0	0	0	0	0	0	0	0	22	22	36	36	34	34	34	34	36	36	36	36	36
15. *S. minus* SKS215	3.79	3.79	3.79	3.79	3.37	3.37	3.58	3.79	3.79	8.21	0.00	0.00	0.00	0.00		0	0	0	0	0	0	0	0	0	22	22	36	36	34	34	34	34	36	36	36	36	36
16. *S. minus* SKS386	3.79	3.79	3.79	3.79	3.37	3.37	3.58	3.79	3.79	8.21	0.00	0.00	0.00	0.00	0.00		0	0	0	0	0	0	0	0	22	22	36	36	34	34	34	34	36	36	36	36	36
17. *S. minus* SKS743	3.79	3.79	3.79	3.79	3.37	3.37	3.58	3.79	3.79	8.21	0.00	0.00	0.00	0.00	0.00	0.00		0	0	0	0	0	0	0	22	22	36	36	34	34	34	34	36	36	36	36	36
18. *S. minus* SKS794	3.79	3.79	3.79	3.79	3.37	3.37	3.58	3.79	3.79	8.21	0.00	0.00	0.00	0.00	0.00	0.00	0.00		0	0	0	0	0	0	22	22	36	36	34	34	34	34	36	36	36	36	36
19. *S. minus* SKS840	3.79	3.79	3.79	3.79	3.37	3.37	3.58	3.79	3.79	8.21	0.00	0.00	0.00	0.00	0.00	0.00	0.00	0.00		0	0	0	0	0	22	22	36	36	34	34	34	34	36	36	36	36	36
20. *S. minus* SKS1018	3.79	3.79	3.79	3.79	3.37	3.37	3.58	3.79	3.79	8.21	0.00	0.00	0.00	0.00	0.00	0.00	0.00	0.00	0.00		0	0	0	0	22	22	36	36	34	34	34	34	36	36	36	36	36
21. *S. minus* SKS1044	3.79	3.79	3.79	3.79	3.37	3.37	3.58	3.79	3.79	8.21	0.00	0.00	0.00	0.00	0.00	0.00	0.00	0.00	0.00	0.00		0	0	0	22	22	36	36	34	34	34	34	36	36	36	36	36
22. *S. minus* SKS731	3.79	3.79	3.79	3.79	3.37	3.37	3.58	3.79	3.79	8.21	0.00	0.00	0.00	0.00	0.00	0.00	0.00	0.00	0.00	0.00	0.00		0	0	22	22	36	36	34	34	34	34	36	36	36	36	36
23. *S. minus* GuS1058	3.79	3.79	3.79	3.79	3.37	3.37	3.58	3.79	3.79	8.21	0.00	0.00	0.00	0.00	0.00	0.00	0.00	0.00	0.00	0.00	0.00	0.00		0	22	22	36	36	34	34	34	34	36	36	36	36	36
24. *S. minus* SKS1073	3.79	3.79	3.79	3.79	3.37	3.37	3.58	3.79	3.79	8.21	0.00	0.00	0.00	0.00	0.00	0.00	0.00	0.00	0.00	0.00	0.00	0.00	0.00		22	22	36	36	34	34	34	34	36	36	36	36	36
25. *S. subtilis* SKS937	4.63	4.63	4.63	4.63	4.21	4.21	4.42	4.63	4.63	8.84	4.63	4.63	4.63	4.63	4.63	4.63	4.63	4.63	4.63	4.63	4.63	4.63	4.63	4.63		0	46	46	44	44	44	44	44	42	42	42	42
26. *S. subtilis* SKS1056	4.63	4.63	4.63	4.63	4.21	4.21	4.42	4.63	4.63	8.84	4.63	4.63	4.63	4.63	4.63	4.63	4.63	4.63	4.63	4.63	4.63	4.63	4.63	4.63	0.00		46	46	44	44	44	44	44	42	42	42	42
27. *S. teres* SKS026	9.47	9.47	9.47	9.47	9.47	9.47	9.68	9.47	9.47	7.58	7.58	7.58	7.58	7.58	7.58	7.58	7.58	7.58	7.58	7.58	7.58	7.58	7.58	7.58	9.68	9.68		0	5	5	5	5	21	13	13	13	13
28. *S. teres* SKS351	9.47	9.47	9.47	9.47	9.47	9.47	9.68	9.47	9.47	7.58	7.58	7.58	7.58	7.58	7.58	7.58	7.58	7.58	7.58	7.58	7.58	7.58	7.58	7.58	9.68	9.68	0.00		5	5	5	5	21	13	13	13	13
29. *S. teres* SKS085	9.05	9.05	9.05	9.05	9.05	9.05	9.26	9.05	9.05	6.95	7.16	7.16	7.16	7.16	7.16	7.16	7.16	7.16	7.16	7.16	7.16	7.16	7.16	7.16	9.26	9.26	1.05	1.05		0	0	0	21	16	16	16	16
30. *S. teres* SKS107	9.05	9.05	9.05	9.05	9.05	9.05	9.26	9.05	9.05	6.95	7.16	7.16	7.16	7.16	7.16	7.16	7.16	7.16	7.16	7.16	7.16	7.16	7.16	7.16	9.26	9.26	1.05	1.05	0.00		0	0	21	16	16	16	16
31. *S. teres* SKS187	9.05	9.05	9.05	9.05	9.05	9.05	9.26	9.05	9.05	6.95	7.16	7.16	7.16	7.16	7.16	7.16	7.16	7.16	7.16	7.16	7.16	7.16	7.16	7.16	9.26	9.26	1.05	1.05	0.00	0.00		0	21	16	16	16	16
32. *S. teres* SKS988	9.05	9.05	9.05	9.05	9.05	9.05	9.26	9.05	9.05	6.95	7.16	7.16	7.16	7.16	7.16	7.16	7.16	7.16	7.16	7.16	7.16	7.16	7.16	7.16	9.26	9.26	1.05	1.05	0.00	0.00	0.00		21	16	16	16	16
33. *S. teres* SKS1045	9.26	9.26	9.26	9.26	9.26	9.26	9.47	9.26	9.26	7.79	7.58	7.58	7.58	7.58	7.58	7.58	7.58	7.58	7.58	7.58	7.58	7.58	7.58	7.58	9.26	9.26	4.42	4.42	4.42	4.42	4.42	4.42		21	21	21	21
34. *S. yagiui* SKS071	8.21	8.21	8.21	8.21	8.21	8.21	8.42	8.21	8.21	6.32	7.58	7.58	7.58	7.58	7.58	7.58	7.58	7.58	7.58	7.58	7.58	7.58	7.58	7.58	8.84	8.84	2.74	2.74	3.37	3.37	3.37	3.37	4.42		0	0	0
35. *S. yagiui* SKS164	8.21	8.21	8.21	8.21	8.21	8.21	8.42	8.21	8.21	6.32	7.58	7.58	7.58	7.58	7.58	7.58	7.58	7.58	7.58	7.58	7.58	7.58	7.58	7.58	8.84	8.84	2.74	2.74	3.37	3.37	3.37	3.37	4.42	0.00		0	0
36. *S. yagiui* SKS787	8.21	8.21	8.21	8.21	8.21	8.21	8.42	8.21	8.21	6.32	7.58	7.58	7.58	7.58	7.58	7.58	7.58	7.58	7.58	7.58	7.58	7.58	7.58	7.58	8.84	8.84	2.74	2.74	3.37	3.37	3.37	3.37	4.42	0.00	0.00		0
37. *S. yagiui* SKS977	8.21	8.21	8.21	8.21	8.21	8.21	8.42	8.21	8.21	6.32	7.58	7.58	7.58	7.58	7.58	7.58	7.58	7.58	7.58	7.58	7.58	7.58	7.58	7.58	8.84	8.84	2.74	2.74	3.37	3.37	3.37	3.37	4.42	0.00	0.00	0.00	

Supplementary Table S9. Numbers of nucleotide differences (above diagonal) and pairwise distances (%, below diagonal) of the mitochondrial CO1 gene sequences among 37 *Spirostomum* populations.

Taxon	1	2	3	4	5	6	7	8	9	10	11	12	13	14	15	16	17	18	19	20	21	22	23	24	25	26	27	28	29	30	31	32	33	34	35	36	37
1. *S. ambiguum* SKS184		0	114	7	102	114	7	7	38	139	166	165	165	164	164	164	165	165	163	164	164	165	164	164	124	123	137	137	147	125	125	125	125	120	129	130	132
2. *S. ambiguum* SKS185	0.00		114	7	102	114	7	7	38	139	166	165	165	164	164	164	165	165	163	164	164	165	164	164	124	123	137	137	147	125	125	125	125	120	129	130	132
3. *S. ambiguum* SKS254	20.11	20.11		116	75	1	116	116	109	149	170	172	172	171	171	171	172	172	171	171	171	172	171	171	144	145	146	146	152	132	132	132	132	131	136	135	135
4. *S. ambiguum* SKS949	1.23	1.23	20.46		104	116	0	0	40	140	165	164	164	163	163	163	164	164	162	163	163	164	163	163	124	123	135	135	146	124	124	124	124	121	130	129	133
5. *S. ambiguum* SKS779	17.99	17.99	13.23	18.34		75	104	104	98	140	163	160	160	159	159	159	160	160	159	159	159	160	159	159	133	133	141	141	141	130	130	130	130	124	134	131	137
6. *S. ambiguum* SKS167	20.11	20.11	0.18	20.46	13.23		116	116	109	149	170	172	172	171	171	171	172	172	171	171	171	172	171	171	144	145	145	145	152	132	132	132	132	131	136	135	135
7. *S. ambiguum* SKS947	1.23	1.23	20.46	0.00	18.34	20.46		0	40	140	165	164	164	163	163	163	164	164	162	163	163	164	163	163	124	123	135	135	146	124	124	124	124	121	130	129	133
8. *S. ambiguum* SKS363	1.23	1.23	20.46	0.00	18.34	20.46	0.00		40	140	165	164	164	163	163	163	164	164	162	163	163	164	163	163	124	123	135	135	146	124	124	124	124	121	130	129	133
9. *S. ambiguum* SKS989	6.70	6.70	19.22	7.05	17.28	19.22	7.05	7.05		138	167	166	166	165	165	165	166	166	164	165	165	166	165	165	123	123	143	143	146	126	126	126	126	123	135	135	136
10. *S. caudatum* SKS302	24.51	24.51	26.28	24.69	24.69	26.28	24.69	24.69	24.34		142	144	144	143	143	143	144	144	142	143	143	144	143	143	131	134	111	111	126	95	95	95	95	111	116	115	116
11. *S. minus* GuS1058	29.28	29.28	29.98	29.10	28.75	29.98	29.10	29.10	29.45	25.04		14	14	13	13	13	14	14	12	13	13	14	13	13	156	159	146	146	151	134	134	134	134	151	152	152	154
12. *S. minus* SKS161	29.10	29.10	30.34	28.92	28.22	30.34	28.92	28.92	29.28	25.40	2.47		0	1	1	1	0	0	2	1	1	0	1	1	153	156	139	139	147	128	128	128	128	146	148	146	149
13. *S. minus* SKS182	29.10	29.10	30.34	28.92	28.22	30.34	28.92	28.92	29.28	25.40	2.47	0.00		1	1	1	0	0	2	1	1	0	1	1	153	156	139	139	147	128	128	128	128	146	148	146	149
14. *S. minus* SKS214	28.92	28.92	30.16	28.75	28.04	30.16	28.75	28.75	29.10	25.22	2.29	0.18	0.18		0	0	1	1	1	0	0	1	0	0	152	155	138	138	146	127	127	127	127	145	147	145	148
15. *S. minus* SKS215	28.92	28.92	30.16	28.75	28.04	30.16	28.75	28.75	29.10	25.22	2.29	0.18	0.18	0.00		0	1	1	1	0	0	1	0	0	152	155	138	138	146	127	127	127	127	145	147	145	148
16. *S. minus* SKS386	28.92	28.92	30.16	28.75	28.04	30.16	28.75	28.75	29.10	25.22	2.29	0.18	0.18	0.00	0.00		1	1	1	0	0	1	0	0	152	155	138	138	146	127	127	127	127	145	147	145	148
17. *S. minus* SKS731	29.10	29.10	30.34	28.92	28.22	30.34	28.92	28.92	29.28	25.40	2.47	0.00	0.00	0.18	0.18	0.18		0	2	1	1	0	1	1	153	156	139	139	147	128	128	128	128	146	148	146	149
18. *S. minus* SKS743	29.10	29.10	30.34	28.92	28.22	30.34	28.92	28.92	29.28	25.40	2.47	0.00	0.00	0.18	0.18	0.18	0.00		2	1	1	0	1	1	153	156	139	139	147	128	128	128	128	146	148	146	149
19. *S. minus* SKS794	28.75	28.75	30.16	28.57	28.04	30.16	28.57	28.57	28.92	25.04	2.12	0.35	0.35	0.18	0.18	0.18	0.35	0.35		1	1	2	1	1	151	154	138	138	147	126	126	126	126	145	146	144	147
20. *S. minus* SKS840	28.92	28.92	30.16	28.75	28.04	30.16	28.75	28.75	29.10	25.22	2.29	0.18	0.18	0.00	0.00	0.00	0.18	0.18	0.18		0	1	0	0	152	155	138	138	146	127	127	127	127	145	147	145	148
21. *S. minus* SKS1018	28.92	28.92	30.16	28.75	28.04	30.16	28.75	28.75	29.10	25.22	2.29	0.18	0.18	0.00	0.00	0.00	0.18	0.18	0.18	0.00		1	0	0	152	155	138	138	146	127	127	127	127	145	147	145	148
22. *S. minus* SKS1044	29.10	29.10	30.34	28.92	28.22	30.34	28.92	28.92	29.28	25.40	2.47	0.00	0.00	0.18	0.18	0.18	0.00	0.00	0.35	0.18	0.18		1	1	153	156	139	139	147	128	128	128	128	146	148	146	149
23. *S. minus* SKS1073	28.92	28.92	30.16	28.75	28.04	30.16	28.75	28.75	29.10	25.22	2.29	0.18	0.18	0.00	0.00	0.00	0.18	0.18	0.18	0.00	0.00	0.18		0	152	155	138	138	146	127	127	127	127	145	147	145	148
24. *S. minus* SKS939	28.92	28.92	30.16	28.75	28.04	30.16	28.75	28.75	29.10	25.22	2.29	0.18	0.18	0.00	0.00	0.00	0.18	0.18	0.18	0.00	0.00	0.18	0.00		152	155	138	138	146	127	127	127	127	145	147	145	148
25. *S. subtilis* SKS937	21.87	21.87	25.40	21.87	23.46	25.40	21.87	21.87	21.69	23.10	27.51	26.98	26.98	26.81	26.81	26.81	26.98	26.98	26.63	26.81	26.81	26.98	26.81	26.81		8	127	127	143	117	117	117	117	124	136	134	132
26. *S. subtilis* SKS1056	21.69	21.69	25.57	21.69	23.46	25.57	21.69	21.69	21.69	23.63	28.04	27.51	27.51	27.34	27.34	27.34	27.51	27.51	27.16	27.34	27.34	27.51	27.34	27.34	1.41		129	129	143	118	118	118	118	126	137	135	134
27. *S. teres* SKS026	24.16	24.16	25.75	23.81	24.87	25.57	23.81	23.81	25.22	19.58	25.75	24.51	24.51	24.34	24.34	24.34	24.51	24.51	24.34	24.34	24.34	24.51	24.34	24.34	22.40	22.75		0	118	77	77	77	77	106	115	115	111
28. *S. teres* SKS351	24.16	24.16	25.75	23.81	24.87	25.57	23.81	23.81	25.22	19.58	25.75	24.51	24.51	24.34	24.34	24.34	24.51	24.51	24.34	24.34	24.34	24.51	24.34	24.34	22.40	22.75	0.00		118	77	77	77	77	106	115	115	111
29. *S. teres* SKS1045	25.93	25.93	26.81	25.75	24.87	26.81	25.75	25.75	25.75	22.22	26.63	25.93	25.93	25.75	25.75	25.75	25.93	25.93	25.93	25.75	25.75	25.93	25.75	25.75	25.22	25.22	20.81	20.81		92	92	92	92	120	122	122	123
30. *S. teres* SKS85	22.05	22.05	23.28	21.87	22.93	23.28	21.87	21.87	22.22	16.75	23.63	22.57	22.57	22.40	22.40	22.40	22.57	22.57	22.22	22.40	22.40	22.57	22.40	22.40	20.63	20.81	13.58	13.58	16.23		0	0	0	96	100	98	108
31. *S. teres* SKS988	22.05	22.05	23.28	21.87	22.93	23.28	21.87	21.87	22.22	16.75	23.63	22.57	22.57	22.40	22.40	22.40	22.57	22.57	22.22	22.40	22.40	22.57	22.40	22.40	20.63	20.81	13.58	13.58	16.23	0.00		0	0	96	100	98	108
32. *S. teres* SKS107	22.05	22.05	23.28	21.87	22.93	23.28	21.87	21.87	22.22	16.75	23.63	22.57	22.57	22.40	22.40	22.40	22.57	22.57	22.22	22.40	22.40	22.57	22.40	22.40	20.63	20.81	13.58	13.58	16.23	0.00	0.00		0	96	100	98	108
33. *S. teres* SKS187	22.05	22.05	23.28	21.87	22.93	23.28	21.87	21.87	22.22	16.75	23.63	22.57	22.57	22.40	22.40	22.40	22.57	22.57	22.22	22.40	22.40	22.57	22.40	22.40	20.63	20.81	13.58	13.58	16.23	0.00	0.00	0.00		96	100	98	108
34. *S. yagiui* SKS787	21.16	21.16	23.10	21.34	21.87	23.10	21.34	21.34	21.69	19.58	26.63	25.75	25.75	25.57	25.57	25.57	25.75	25.75	25.57	25.57	25.57	25.75	25.57	25.57	21.87	22.22	18.69	18.69	21.16	16.93	16.93	16.93	16.93		72	70	70
35. *S. yagiui* SKS071	22.75	22.75	23.99	22.93	23.63	23.99	22.93	22.93	23.81	20.46	26.81	26.10	26.10	25.93	25.93	25.93	26.10	26.10	25.75	25.93	25.93	26.10	25.93	25.93	23.99	24.16	20.28	20.28	21.52	17.64	17.64	17.64	17.64	12.70		7	33
36. *S. yagiui* SKS977	22.93	22.93	23.81	22.75	23.10	23.81	22.75	22.75	23.81	20.28	26.81	25.75	25.75	25.57	25.57	25.57	25.75	25.75	25.40	25.57	25.57	25.75	25.57	25.57	23.63	23.81	20.28	20.28	21.52	17.28	17.28	17.28	17.28	12.35	1.23		32
37. *S. yagiui* SKS164	23.28	23.28	23.81	23.46	24.16	23.81	23.46	23.46	23.99	20.46	27.16	26.28	26.28	26.10	26.10	26.10	26.28	26.28	25.93	26.10	26.10	26.28	26.10	26.10	23.28	23.63	19.58	19.58	21.69	19.05	19.05	19.05	19.05	12.35	5.82	5.64	

Supplementary Table S10. Numbers of amino acid differences (above diagonal) and pairwise distances (%, below diagonal) of the mitochondrial CO1 gene sequences among 37 *Spirostomum* populations.

Taxon	1	2	3	4	5	6	7	8	9	10	11	12	13	14	15	16	17	18	19	20	21	22	23	24	25	26	27	28	29	30	31	32	33	34	35	36	37
1. *S. ambiguum* SKS184		0	29	1	29	29	1	1	4	42	49	46	46	46	46	46	46	46	46	46	46	46	46	46	37	36	44	44	43	39	39	39	39	38	40	40	40
2. *S. ambiguum* SKS185	0.00		29	1	29	29	1	1	4	42	49	46	46	46	46	46	46	46	46	46	46	46	46	46	37	36	44	44	43	39	39	39	39	38	40	40	40
3. *S. ambiguum* SKS254	15.34	15.34		30	17	0	30	30	28	54	55	54	54	54	54	54	54	54	54	54	54	54	54	54	44	44	48	48	47	44	44	44	44	44	45	45	45
4. *S. ambiguum* SKS949	0.53	0.53	15.87		30	30	0	0	5	43	48	45	45	45	45	45	45	45	45	45	45	45	45	45	38	37	44	44	43	39	39	39	39	39	41	41	41
5. *S. ambiguum* SKS779	15.34	15.34	8.99	15.87		17	30	30	27	51	51	50	50	50	50	50	50	50	50	50	50	50	50	50	43	42	46	46	46	43	43	43	43	41	43	43	44
6. *S. ambiguum* SKS167	15.34	15.34	0.00	15.87	8.99		30	30	28	54	55	54	54	54	54	54	54	54	54	54	54	54	54	54	44	44	48	48	47	44	44	44	44	44	45	45	45
7. *S. ambiguum* SKS947	0.53	0.53	15.87	0.00	15.87	15.87		0	5	43	48	45	45	45	45	45	45	45	45	45	45	45	45	45	38	37	44	44	43	39	39	39	39	39	41	41	41
8. *S. ambiguum* SKS363	0.53	0.53	15.87	0.00	15.87	15.87	0.00		5	43	48	45	45	45	45	45	45	45	45	45	45	45	45	45	38	37	44	44	43	39	39	39	39	39	41	41	41
9. *S. ambiguum* SKS989	2.12	2.12	14.81	2.65	14.29	14.81	2.65	2.65		43	50	47	47	47	47	47	47	47	47	47	47	47	47	47	36	35	43	43	41	38	38	38	38	39	41	41	41
10. *S. caudatum* SKS302	22.22	22.22	28.57	22.75	26.98	28.57	22.75	22.75	22.75		46	46	46	46	46	46	46	46	46	46	46	46	46	46	43	43	33	33	37	28	28	28	28	34	31	31	32
11. *S. minus* GuS1058	25.93	25.93	29.10	25.40	26.98	29.10	25.40	25.40	26.46	24.34		3	3	3	3	3	3	3	3	3	3	3	3	3	57	56	51	51	39	46	46	46	46	49	45	45	46
12. *S. minus* SKS161	24.34	24.34	28.57	23.81	26.46	28.57	23.81	23.81	24.87	24.34	1.59		0	0	0	0	0	0	0	0	0	0	0	0	56	55	48	48	38	43	43	43	43	46	42	42	43
13. *S. minus* SKS182	24.34	24.34	28.57	23.81	26.46	28.57	23.81	23.81	24.87	24.34	1.59	0.00		0	0	0	0	0	0	0	0	0	0	0	56	55	48	48	38	43	43	43	43	46	42	42	43
14. *S. minus* SKS214	24.34	24.34	28.57	23.81	26.46	28.57	23.81	23.81	24.87	24.34	1.59	0.00	0.00		0	0	0	0	0	0	0	0	0	0	56	55	48	48	38	43	43	43	43	46	42	42	43
15. *S. minus* SKS215	24.34	24.34	28.57	23.81	26.46	28.57	23.81	23.81	24.87	24.34	1.59	0.00	0.00	0.00		0	0	0	0	0	0	0	0	0	56	55	48	48	38	43	43	43	43	46	42	42	43
16. *S. minus* SKS386	24.34	24.34	28.57	23.81	26.46	28.57	23.81	23.81	24.87	24.34	1.59	0.00	0.00	0.00	0.00		0	0	0	0	0	0	0	0	56	55	48	48	38	43	43	43	43	46	42	42	43
17. *S. minus* SKS731	24.34	24.34	28.57	23.81	26.46	28.57	23.81	23.81	24.87	24.34	1.59	0.00	0.00	0.00	0.00	0.00		0	0	0	0	0	0	0	56	55	48	48	38	43	43	43	43	46	42	42	43
18. *S. minus* SKS743	24.34	24.34	28.57	23.81	26.46	28.57	23.81	23.81	24.87	24.34	1.59	0.00	0.00	0.00	0.00	0.00	0.00		0	0	0	0	0	0	56	55	48	48	38	43	43	43	43	46	42	42	43
19. *S. minus* SKS794	24.34	24.34	28.57	23.81	26.46	28.57	23.81	23.81	24.87	24.34	1.59	0.00	0.00	0.00	0.00	0.00	0.00	0.00		0	0	0	0	0	56	55	48	48	38	43	43	43	43	46	42	42	43
20. *S. minus* SKS840	24.34	24.34	28.57	23.81	26.46	28.57	23.81	23.81	24.87	24.34	1.59	0.00	0.00	0.00	0.00	0.00	0.00	0.00	0.00		0	0	0	0	56	55	48	48	38	43	43	43	43	46	42	42	43
21. *S. minus* SKS1018	24.34	24.34	28.57	23.81	26.46	28.57	23.81	23.81	24.87	24.34	1.59	0.00	0.00	0.00	0.00	0.00	0.00	0.00	0.00	0.00		0	0	0	56	55	48	48	38	43	43	43	43	46	42	42	43
22. *S. minus* SKS1044	24.34	24.34	28.57	23.81	26.46	28.57	23.81	23.81	24.87	24.34	1.59	0.00	0.00	0.00	0.00	0.00	0.00	0.00	0.00	0.00	0.00		0	0	56	55	48	48	38	43	43	43	43	46	42	42	43
23. *S. minus* SKS1073	24.34	24.34	28.57	23.81	26.46	28.57	23.81	23.81	24.87	24.34	1.59	0.00	0.00	0.00	0.00	0.00	0.00	0.00	0.00	0.00	0.00	0.00		0	56	55	48	48	38	43	43	43	43	46	42	42	43
24. *S. minus* SKS939	24.34	24.34	28.57	23.81	26.46	28.57	23.81	23.81	24.87	24.34	1.59	0.00	0.00	0.00	0.00	0.00	0.00	0.00	0.00	0.00	0.00	0.00	0.00		56	55	48	48	38	43	43	43	43	46	42	42	43
25. *S. subtilis* SKS937	19.58	19.58	23.28	20.11	22.75	23.28	20.11	20.11	19.05	22.75	30.16	29.63	29.63	29.63	29.63	29.63	29.63	29.63	29.63	29.63	29.63	29.63	29.63	29.63		1	45	45	41	38	38	38	38	46	46	46	46
26. *S. subtilis* SKS1056	19.05	19.05	23.28	19.58	22.22	23.28	19.58	19.58	18.52	22.75	29.63	29.10	29.10	29.10	29.10	29.10	29.10	29.10	29.10	29.10	29.10	29.10	29.10	29.10	0.53		44	44	40	37	37	37	37	46	46	46	46
27. *S. teres* SKS026	23.28	23.28	25.40	23.28	24.34	25.40	23.28	23.28	22.75	17.46	26.98	25.40	25.40	25.40	25.40	25.40	25.40	25.40	25.40	25.40	25.40	25.40	25.40	25.40	23.81	23.28		0	34	22	22	22	22	35	30	30	31
28. *S. teres* SKS351	23.28	23.28	25.40	23.28	24.34	25.40	23.28	23.28	22.75	17.46	26.98	25.40	25.40	25.40	25.40	25.40	25.40	25.40	25.40	25.40	25.40	25.40	25.40	25.40	23.81	23.28	0.00		34	22	22	22	22	35	30	30	31
29. *S. teres* SKS1045	22.75	22.75	24.87	22.75	24.34	24.87	22.75	22.75	21.69	19.58	20.63	20.11	20.11	20.11	20.11	20.11	20.11	20.11	20.11	20.11	20.11	20.11	20.11	20.11	21.69	21.16	17.99	17.99		24	24	24	24	36	32	32	33
30. *S. teres* SKS85	20.63	20.63	23.28	20.63	22.75	23.28	20.63	20.63	20.11	14.81	24.34	22.75	22.75	22.75	22.75	22.75	22.75	22.75	22.75	22.75	22.75	22.75	22.75	22.75	20.11	19.58	11.64	11.64	12.70		0	0	0	33	27	27	28
31. *S. teres* SKS988	20.63	20.63	23.28	20.63	22.75	23.28	20.63	20.63	20.11	14.81	24.34	22.75	22.75	22.75	22.75	22.75	22.75	22.75	22.75	22.75	22.75	22.75	22.75	22.75	20.11	19.58	11.64	11.64	12.70	0.00		0	0	33	27	27	28
32. *S. teres* SKS107	20.63	20.63	23.28	20.63	22.75	23.28	20.63	20.63	20.11	14.81	24.34	22.75	22.75	22.75	22.75	22.75	22.75	22.75	22.75	22.75	22.75	22.75	22.75	22.75	20.11	19.58	11.64	11.64	12.70	0.00	0.00		0	33	27	27	28
33. *S. teres* SKS187	20.63	20.63	23.28	20.63	22.75	23.28	20.63	20.63	20.11	14.81	24.34	22.75	22.75	22.75	22.75	22.75	22.75	22.75	22.75	22.75	22.75	22.75	22.75	22.75	20.11	19.58	11.64	11.64	12.70	0.00	0.00	0.00		33	27	27	28
34. *S. yagiui* SKS787	20.11	20.11	23.28	20.63	21.69	23.28	20.63	20.63	20.63	17.99	25.93	24.34	24.34	24.34	24.34	24.34	24.34	24.34	24.34	24.34	24.34	24.34	24.34	24.34	24.34	24.34	18.52	18.52	19.05	17.46	17.46	17.46	17.46		14	14	15
35. *S. yagiui* SKS071	21.16	21.16	23.81	21.69	22.75	23.81	21.69	21.69	21.69	16.40	23.81	22.22	22.22	22.22	22.22	22.22	22.22	22.22	22.22	22.22	22.22	22.22	22.22	22.22	24.34	24.34	15.87	15.87	16.93	14.29	14.29	14.29	14.29	7.41		0	2
36. *S. yagiui* SKS977	21.16	21.16	23.81	21.69	22.75	23.81	21.69	21.69	21.69	16.40	23.81	22.22	22.22	22.22	22.22	22.22	22.22	22.22	22.22	22.22	22.22	22.22	22.22	22.22	24.34	24.34	15.87	15.87	16.93	14.29	14.29	14.29	14.29	7.41	0.00		2
37. *S. yagiui* SKS164	21.16	21.16	23.81	21.69	23.28	23.81	21.69	21.69	21.69	16.93	24.34	22.75	22.75	22.75	22.75	22.75	22.75	22.75	22.75	22.75	22.75	22.75	22.75	22.75	24.34	24.34	16.40	16.40	17.46	14.81	14.81	14.81	14.81	7.94	1.06	1.06	

Supplementary Table S11. Numbers of nucleotide differences (above diagonal) and pairwise distances (%, below diagonal) of the mitochondrial CO1 (nt) gene sequences (without insert) among 37 *Spirostomum* populations.

Taxon	1	2	3	4	5	6	7	8	9	10	11	12	13	14	15	16	17	18	19	20	21	22	23	24	25	26	27	28	29	30	31	32	33	34	35	36	37
1. *S. ambiguum* SKS167		0	43	29	40	43	43	43	43	48	57	57	56	57	57	57	57	57	57	57	57	57	57	57	54	53	49	52	42	42	49	42	42	44	41	40	42
2. *S. ambiguum* SKS254	0.00		43	29	40	43	43	43	43	48	57	57	56	57	57	57	57	57	57	57	57	57	57	57	54	53	49	52	42	42	49	42	42	44	41	40	42
3. *S. ambiguum* SKS363	15.25	15.25		35	15	3	3	0	0	53	53	53	53	53	53	53	53	53	53	53	53	53	53	53	43	43	47	55	46	46	47	46	46	49	47	41	46
4. *S. ambiguum* SKS779	10.28	10.28	12.41		37	34	34	35	35	50	51	51	53	51	51	51	51	51	51	51	51	51	51	51	53	53	49	48	46	46	49	46	46	42	42	42	41
5. *S. ambiguum* SKS989	14.18	14.18	5.32	13.12		15	15	15	15	51	53	53	53	53	53	53	53	53	53	53	53	53	53	53	44	43	51	54	46	46	51	46	46	48	50	43	48
6. *S. ambiguum* SKS184	15.25	15.25	1.06	12.06	5.32		0	3	3	54	54	54	54	54	54	54	54	54	54	54	54	54	54	54	45	45	49	54	47	47	49	47	47	49	47	43	48
7. *S. ambiguum* SKS185	15.25	15.25	1.06	12.06	5.32	0.00		3	3	54	54	54	54	54	54	54	54	54	54	54	54	54	54	54	45	45	49	54	47	47	49	47	47	49	47	43	48
8. *S. ambiguum* SKS947	15.25	15.25	0.00	12.41	5.32	1.06	1.06		0	53	53	53	53	53	53	53	53	53	53	53	53	53	53	53	43	43	47	55	46	46	47	46	46	49	47	41	46
9. *S. ambiguum* SKS949	15.25	15.25	0.00	12.41	5.32	1.06	1.06	0.00		53	53	53	53	53	53	53	53	53	53	53	53	53	53	53	43	43	47	55	46	46	47	46	46	49	47	41	46
10. *S. caudatum* SKS302	17.02	17.02	18.79	17.73	18.09	19.15	19.15	18.79	18.79		46	46	45	46	46	46	46	46	46	46	46	46	46	46	55	53	51	52	44	44	51	44	44	48	45	40	47
11. *S. minus* SKS1018	20.21	20.21	18.79	18.09	18.79	19.15	19.15	18.79	18.79	16.31		0	6	0	0	0	0	0	0	0	0	0	0	0	50	47	48	55	42	42	48	42	42	50	52	49	47
12. *S. minus* SKS1044	20.21	20.21	18.79	18.09	18.79	19.15	19.15	18.79	18.79	16.31	0.00		6	0	0	0	0	0	0	0	0	0	0	0	50	47	48	55	42	42	48	42	42	50	52	49	47
13. *S. minus* GuS1058	19.86	19.86	18.79	18.79	18.79	19.15	19.15	18.79	18.79	15.96	2.13	2.13		6	6	6	6	6	6	6	6	6	6	6	53	50	52	57	46	46	52	46	46	52	55	52	51
14. *S. minus* SKS1073	20.21	20.21	18.79	18.09	18.79	19.15	19.15	18.79	18.79	16.31	0.00	0.00	2.13		0	0	0	0	0	0	0	0	0	0	50	47	48	55	42	42	48	42	42	50	52	49	47
15. *S. minus* SKS161	20.21	20.21	18.79	18.09	18.79	19.15	19.15	18.79	18.79	16.31	0.00	0.00	2.13	0.00		0	0	0	0	0	0	0	0	0	50	47	48	55	42	42	48	42	42	50	52	49	47
16. *S. minus* SKS182	20.21	20.21	18.79	18.09	18.79	19.15	19.15	18.79	18.79	16.31	0.00	0.00	2.13	0.00	0.00		0	0	0	0	0	0	0	0	50	47	48	55	42	42	48	42	42	50	52	49	47
17. *S. minus* SKS214	20.21	20.21	18.79	18.09	18.79	19.15	19.15	18.79	18.79	16.31	0.00	0.00	2.13	0.00	0.00	0.00		0	0	0	0	0	0	0	50	47	48	55	42	42	48	42	42	50	52	49	47
18. *S. minus* SKS215	20.21	20.21	18.79	18.09	18.79	19.15	19.15	18.79	18.79	16.31	0.00	0.00	2.13	0.00	0.00	0.00	0.00		0	0	0	0	0	0	50	47	48	55	42	42	48	42	42	50	52	49	47
19. *S. minus* SKS386	20.21	20.21	18.79	18.09	18.79	19.15	19.15	18.79	18.79	16.31	0.00	0.00	2.13	0.00	0.00	0.00	0.00	0.00		0	0	0	0	0	50	47	48	55	42	42	48	42	42	50	52	49	47
20. *S. minus* SKS731	20.21	20.21	18.79	18.09	18.79	19.15	19.15	18.79	18.79	16.31	0.00	0.00	2.13	0.00	0.00	0.00	0.00	0.00	0.00		0	0	0	0	50	47	48	55	42	42	48	42	42	50	52	49	47
21. *S. minus* SKS743	20.21	20.21	18.79	18.09	18.79	19.15	19.15	18.79	18.79	16.31	0.00	0.00	2.13	0.00	0.00	0.00	0.00	0.00	0.00	0.00		0	0	0	50	47	48	55	42	42	48	42	42	50	52	49	47
22. *S. minus* SKS794	20.21	20.21	18.79	18.09	18.79	19.15	19.15	18.79	18.79	16.31	0.00	0.00	2.13	0.00	0.00	0.00	0.00	0.00	0.00	0.00	0.00		0	0	50	47	48	55	42	42	48	42	42	50	52	49	47
23. *S. minus* SKS840	20.21	20.21	18.79	18.09	18.79	19.15	19.15	18.79	18.79	16.31	0.00	0.00	2.13	0.00	0.00	0.00	0.00	0.00	0.00	0.00	0.00	0.00		0	50	47	48	55	42	42	48	42	42	50	52	49	47
24. *S. minus* SKS939	20.21	20.21	18.79	18.09	18.79	19.15	19.15	18.79	18.79	16.31	0.00	0.00	2.13	0.00	0.00	0.00	0.00	0.00	0.00	0.00	0.00	0.00	0.00		50	47	48	55	42	42	48	42	42	50	52	49	47
25. *S. subtilis* SKS1056	19.15	19.15	15.25	18.79	15.60	15.96	15.96	15.25	15.25	19.50	17.73	17.73	18.79	17.73	17.73	17.73	17.73	17.73	17.73	17.73	17.73	17.73	17.73	17.73		6	52	59	48	48	52	48	48	51	49	46	48
26. *S. subtilis* SKS937	18.79	18.79	15.25	18.79	15.25	15.96	15.96	15.25	15.25	18.79	16.67	16.67	17.73	16.67	16.67	16.67	16.67	16.67	16.67	16.67	16.67	16.67	16.67	16.67	2.13		50	59	47	47	50	47	47	51	47	45	48
27. *S. teres* SKS026	17.38	17.38	16.67	17.38	18.09	17.38	17.38	16.67	16.67	18.09	17.02	17.02	18.44	17.02	17.02	17.02	17.02	17.02	17.02	17.02	17.02	17.02	17.02	17.02	18.44	17.73		48	32	32	0	32	32	43	41	37	41
28. *S. teres* SKS1045	18.44	18.44	19.50	17.02	19.15	19.15	19.15	19.50	19.50	18.44	19.50	19.50	20.21	19.50	19.50	19.50	19.50	19.50	19.50	19.50	19.50	19.50	19.50	19.50	20.92	20.92	17.02		36	36	48	36	36	45	40	42	44
29. *S. teres* SKS107	14.89	14.89	16.31	16.31	16.31	16.67	16.67	16.31	16.31	15.60	14.89	14.89	16.31	14.89	14.89	14.89	14.89	14.89	14.89	14.89	14.89	14.89	14.89	14.89	17.02	16.67	11.35	12.77		0	32	0	0	36	37	33	33
30. *S. teres* SKS187	14.89	14.89	16.31	16.31	16.31	16.67	16.67	16.31	16.31	15.60	14.89	14.89	16.31	14.89	14.89	14.89	14.89	14.89	14.89	14.89	14.89	14.89	14.89	14.89	17.02	16.67	11.35	12.77	0.00		32	0	0	36	37	33	33
31. *S. teres* SKS351	17.38	17.38	16.67	17.38	18.09	17.38	17.38	16.67	16.67	18.09	17.02	17.02	18.44	17.02	17.02	17.02	17.02	17.02	17.02	17.02	17.02	17.02	17.02	17.02	18.44	17.73	0.00	17.02	11.35	11.35		32	32	43	41	37	41
32. *S. teres* SKS85	14.89	14.89	16.31	16.31	16.31	16.67	16.67	16.31	16.31	15.60	14.89	14.89	16.31	14.89	14.89	14.89	14.89	14.89	14.89	14.89	14.89	14.89	14.89	14.89	17.02	16.67	11.35	12.77	0.00	0.00	11.35		0	36	37	33	33
33. *S. teres* SKS988	14.89	14.89	16.31	16.31	16.31	16.67	16.67	16.31	16.31	15.60	14.89	14.89	16.31	14.89	14.89	14.89	14.89	14.89	14.89	14.89	14.89	14.89	14.89	14.89	17.02	16.67	11.35	12.77	0.00	0.00	11.35	0.00		36	37	33	33
34. *S. yagiui* SKS071	15.60	15.60	17.38	14.89	17.02	17.38	17.38	17.38	17.38	17.02	17.73	17.73	18.44	17.73	17.73	17.73	17.73	17.73	17.73	17.73	17.73	17.73	17.73	17.73	18.09	18.09	15.25	15.96	12.77	12.77	15.25	12.77	12.77		16	27	4
35. *S. yagiui* SKS164	14.54	14.54	16.67	14.89	17.73	16.67	16.67	16.67	16.67	15.96	18.44	18.44	19.50	18.44	18.44	18.44	18.44	18.44	18.44	18.44	18.44	18.44	18.44	18.44	17.38	16.67	14.54	14.18	13.12	13.12	14.54	13.12	13.12	5.67		24	14
36. *S. yagiui* SKS787	14.18	14.18	14.54	14.89	15.25	15.25	15.25	14.54	14.54	14.18	17.38	17.38	18.44	17.38	17.38	17.38	17.38	17.38	17.38	17.38	17.38	17.38	17.38	17.38	16.31	15.96	13.12	14.89	11.70	11.70	13.12	11.70	11.70	9.57	8.51		25
37. *S. yagiui* SKS977	14.89	14.89	16.31	14.54	17.02	17.02	17.02	16.31	16.31	16.67	16.67	16.67	18.09	16.67	16.67	16.67	16.67	16.67	16.67	16.67	16.67	16.67	16.67	16.67	17.02	17.02	14.54	15.60	11.70	11.70	14.54	11.70	11.70	1.42	4.96	8.87	

Supplementary Table S12. Numbers of amino acid differences (above diagonal) and pairwise distances (%, below diagonal) of the mitochondrial CO1 gene sequences (without insert) among 37 *Spirostomum* populations.

Taxon	1	2	3	4	5	6	7	8	9	10	11	12	13	14	15	16	17	18	19	20	21	22	23	24	25	26	27	28	29	30	31	32	33	34	35	36	37
1. *S. ambiguum* SKS167		0	1	0	1	1	1	1	1	5	1	1	1	1	1	1	1	1	1	1	1	1	1	1	5	5	4	2	3	3	4	3	3	4	4	5	4
2. *S. ambiguum* SKS254	0.00		1	0	1	1	1	1	1	5	1	1	1	1	1	1	1	1	1	1	1	1	1	1	5	5	4	2	3	3	4	3	3	4	4	5	4
3. *S. ambiguum* SKS363	1.06	1.06		1	0	0	0	0	0	4	2	2	2	2	2	2	2	2	2	2	2	2	2	2	4	4	5	3	4	4	5	4	4	5	5	4	5
4. *S. ambiguum* SKS779	0.00	0.00	1.06		1	1	1	1	1	5	1	1	1	1	1	1	1	1	1	1	1	1	1	1	5	5	4	2	3	3	4	3	3	4	4	5	4
5. *S. ambiguum* SKS989	1.06	1.06	0.00	1.06		0	0	0	0	4	2	2	2	2	2	2	2	2	2	2	2	2	2	2	4	4	5	3	4	4	5	4	4	5	5	4	5
6. *S. ambiguum* SKS184	1.06	1.06	0.00	1.06	0.00		0	0	0	4	2	2	2	2	2	2	2	2	2	2	2	2	2	2	4	4	5	3	4	4	5	4	4	5	5	4	5
7. *S. ambiguum* SKS185	1.06	1.06	0.00	1.06	0.00	0.00		0	0	4	2	2	2	2	2	2	2	2	2	2	2	2	2	2	4	4	5	3	4	4	5	4	4	5	5	4	5
8. *S. ambiguum* SKS947	1.06	1.06	0.00	1.06	0.00	0.00	0.00		0	4	2	2	2	2	2	2	2	2	2	2	2	2	2	2	4	4	5	3	4	4	5	4	4	5	5	4	5
9. *S. ambiguum* SKS949	1.06	1.06	0.00	1.06	0.00	0.00	0.00	0.00		4	2	2	2	2	2	2	2	2	2	2	2	2	2	2	4	4	5	3	4	4	5	4	4	5	5	4	5
10. *S. caudatum* SKS302	5.32	5.32	4.26	5.32	4.26	4.26	4.26	4.26	4.26		4	4	4	4	4	4	4	4	4	4	4	4	4	4	6	6	5	3	4	4	5	4	4	5	5	4	5
11. *S. minus* SKS1018	1.06	1.06	2.13	1.06	2.13	2.13	2.13	2.13	2.13	4.26		0	0	0	0	0	0	0	0	0	0	0	0	0	4	4	3	1	2	2	3	2	2	3	3	4	3
12. *S. minus* SKS1044	1.06	1.06	2.13	1.06	2.13	2.13	2.13	2.13	2.13	4.26	0.00		0	0	0	0	0	0	0	0	0	0	0	0	4	4	3	1	2	2	3	2	2	3	3	4	3
13. *S. minus* GuS1058	1.06	1.06	2.13	1.06	2.13	2.13	2.13	2.13	2.13	4.26	0.00	0.00		0	0	0	0	0	0	0	0	0	0	0	4	4	3	1	2	2	3	2	2	3	3	4	3
14. *S. minus* SKS1073	1.06	1.06	2.13	1.06	2.13	2.13	2.13	2.13	2.13	4.26	0.00	0.00	0.00		0	0	0	0	0	0	0	0	0	0	4	4	3	1	2	2	3	2	2	3	3	4	3
15. *S. minus* SKS161	1.06	1.06	2.13	1.06	2.13	2.13	2.13	2.13	2.13	4.26	0.00	0.00	0.00	0.00		0	0	0	0	0	0	0	0	0	4	4	3	1	2	2	3	2	2	3	3	4	3
16. *S. minus* SKS182	1.06	1.06	2.13	1.06	2.13	2.13	2.13	2.13	2.13	4.26	0.00	0.00	0.00	0.00	0.00		0	0	0	0	0	0	0	0	4	4	3	1	2	2	3	2	2	3	3	4	3
17. *S. minus* SKS214	1.06	1.06	2.13	1.06	2.13	2.13	2.13	2.13	2.13	4.26	0.00	0.00	0.00	0.00	0.00	0.00		0	0	0	0	0	0	0	4	4	3	1	2	2	3	2	2	3	3	4	3
18. *S. minus* SKS215	1.06	1.06	2.13	1.06	2.13	2.13	2.13	2.13	2.13	4.26	0.00	0.00	0.00	0.00	0.00	0.00	0.00		0	0	0	0	0	0	4	4	3	1	2	2	3	2	2	3	3	4	3
19. *S. minus* SKS386	1.06	1.06	2.13	1.06	2.13	2.13	2.13	2.13	2.13	4.26	0.00	0.00	0.00	0.00	0.00	0.00	0.00	0.00		0	0	0	0	0	4	4	3	1	2	2	3	2	2	3	3	4	3
20. *S. minus* SKS731	1.06	1.06	2.13	1.06	2.13	2.13	2.13	2.13	2.13	4.26	0.00	0.00	0.00	0.00	0.00	0.00	0.00	0.00	0.00		0	0	0	0	4	4	3	1	2	2	3	2	2	3	3	4	3
21. *S. minus* SKS743	1.06	1.06	2.13	1.06	2.13	2.13	2.13	2.13	2.13	4.26	0.00	0.00	0.00	0.00	0.00	0.00	0.00	0.00	0.00	0.00		0	0	0	4	4	3	1	2	2	3	2	2	3	3	4	3
22. *S. minus* SKS794	1.06	1.06	2.13	1.06	2.13	2.13	2.13	2.13	2.13	4.26	0.00	0.00	0.00	0.00	0.00	0.00	0.00	0.00	0.00	0.00	0.00		0	0	4	4	3	1	2	2	3	2	2	3	3	4	3
23. *S. minus* SKS840	1.06	1.06	2.13	1.06	2.13	2.13	2.13	2.13	2.13	4.26	0.00	0.00	0.00	0.00	0.00	0.00	0.00	0.00	0.00	0.00	0.00	0.00		0	4	4	3	1	2	2	3	2	2	3	3	4	3
24. *S. minus* SKS939	1.06	1.06	2.13	1.06	2.13	2.13	2.13	2.13	2.13	4.26	0.00	0.00	0.00	0.00	0.00	0.00	0.00	0.00	0.00	0.00	0.00	0.00	0.00		4	4	3	1	2	2	3	2	2	3	3	4	3
25. *S. subtilis* SKS1056	5.32	5.32	4.26	5.32	4.26	4.26	4.26	4.26	4.26	6.38	4.26	4.26	4.26	4.26	4.26	4.26	4.26	4.26	4.26	4.26	4.26	4.26	4.26	4.26		0	7	5	6	6	7	6	6	7	7	6	7
26. *S. subtilis* SKS937	5.32	5.32	4.26	5.32	4.26	4.26	4.26	4.26	4.26	6.38	4.26	4.26	4.26	4.26	4.26	4.26	4.26	4.26	4.26	4.26	4.26	4.26	4.26	4.26	0.00		7	5	6	6	7	6	6	7	7	6	7
27. *S. teres* SKS026	4.26	4.26	5.32	4.26	5.32	5.32	5.32	5.32	5.32	5.32	3.19	3.19	3.19	3.19	3.19	3.19	3.19	3.19	3.19	3.19	3.19	3.19	3.19	3.19	7.45	7.45		2	3	3	0	3	3	4	4	5	4
28. *S. teres* SKS1045	2.13	2.13	3.19	2.13	3.19	3.19	3.19	3.19	3.19	3.19	1.06	1.06	1.06	1.06	1.06	1.06	1.06	1.06	1.06	1.06	1.06	1.06	1.06	1.06	5.32	5.32	2.13		1	1	2	1	1	2	2	3	2
29. *S. teres* SKS107	3.19	3.19	4.26	3.19	4.26	4.26	4.26	4.26	4.26	4.26	2.13	2.13	2.13	2.13	2.13	2.13	2.13	2.13	2.13	2.13	2.13	2.13	2.13	2.13	6.38	6.38	3.19	1.06		0	3	0	0	3	3	4	3
30. *S. teres* SKS187	3.19	3.19	4.26	3.19	4.26	4.26	4.26	4.26	4.26	4.26	2.13	2.13	2.13	2.13	2.13	2.13	2.13	2.13	2.13	2.13	2.13	2.13	2.13	2.13	6.38	6.38	3.19	1.06	0.00		3	0	0	3	3	4	3
31. *S. teres* SKS351	4.26	4.26	5.32	4.26	5.32	5.32	5.32	5.32	5.32	5.32	3.19	3.19	3.19	3.19	3.19	3.19	3.19	3.19	3.19	3.19	3.19	3.19	3.19	3.19	7.45	7.45	0.00	2.13	3.19	3.19		3	3	4	4	5	4
32. *S. teres* SKS85	3.19	3.19	4.26	3.19	4.26	4.26	4.26	4.26	4.26	4.26	2.13	2.13	2.13	2.13	2.13	2.13	2.13	2.13	2.13	2.13	2.13	2.13	2.13	2.13	6.38	6.38	3.19	1.06	0.00	0.00	3.19		0	3	3	4	3
33. *S. teres* SKS988	3.19	3.19	4.26	3.19	4.26	4.26	4.26	4.26	4.26	4.26	2.13	2.13	2.13	2.13	2.13	2.13	2.13	2.13	2.13	2.13	2.13	2.13	2.13	2.13	6.38	6.38	3.19	1.06	0.00	0.00	3.19	0.00		3	3	4	3
34. *S. yagiui* SKS071	4.26	4.26	5.32	4.26	5.32	5.32	5.32	5.32	5.32	5.32	3.19	3.19	3.19	3.19	3.19	3.19	3.19	3.19	3.19	3.19	3.19	3.19	3.19	3.19	7.45	7.45	4.26	2.13	3.19	3.19	4.26	3.19	3.19		0	1	0
35. *S. yagiui* SKS164	4.26	4.26	5.32	4.26	5.32	5.32	5.32	5.32	5.32	5.32	3.19	3.19	3.19	3.19	3.19	3.19	3.19	3.19	3.19	3.19	3.19	3.19	3.19	3.19	7.45	7.45	4.26	2.13	3.19	3.19	4.26	3.19	3.19	0.00		1	0
36. *S. yagiui* SKS787	5.32	5.32	4.26	5.32	4.26	4.26	4.26	4.26	4.26	4.26	4.26	4.26	4.26	4.26	4.26	4.26	4.26	4.26	4.26	4.26	4.26	4.26	4.26	4.26	6.38	6.38	5.32	3.19	4.26	4.26	5.32	4.26	4.26	1.06	1.06		1
37. *S. yagiui* SKS977	4.26	4.26	5.32	4.26	5.32	5.32	5.32	5.32	5.32	5.32	3.19	3.19	3.19	3.19	3.19	3.19	3.19	3.19	3.19	3.19	3.19	3.19	3.19	3.19	7.45	7.45	4.26	2.13	3.19	3.19	4.26	3.19	3.19	0.00	0.00	1.06	

Supplementary Table S13. Numbers of nucleotide differences (above diagonal) and pairwise distances (%, below diagonal) of the 18S rRNA gene sequences among 31 *Spirostomum* populations.

Taxon	1	2	3	4	5	6	7	8	9	10	11	12	13	14	15	16	17	18	19	20	21	22	23	24	25	26	27	28	29	30	31
1. *S. ambiguum* SKS167		0	4	4	4	4	4	44	21	22	22	22	22	22	22	22	22	22	22	22	22	33	33	40	40	40	38	38	36	36	36
2. *S. ambiguum* SKS254	0.00		4	4	4	4	4	44	21	22	22	22	22	22	22	22	22	22	22	22	22	33	33	40	40	40	38	38	36	36	36
3. *S. ambiguum* SKS779	0.25	0.25		2	2	2	2	44	19	20	20	20	20	20	20	20	20	20	20	20	20	31	31	40	38	38	36	36	34	34	34
4. *S. ambiguum* SKS185	0.25	0.25	0.13		0	0	0	42	21	22	22	22	22	22	22	22	22	22	22	22	22	33	33	40	40	40	38	38	36	36	36
5. *S. ambiguum* SKS989	0.25	0.25	0.13	0.00		0	0	42	21	22	22	22	22	22	22	22	22	22	22	22	22	33	33	40	40	40	38	38	36	36	36
6. *S. ambiguum* SKS363	0.25	0.25	0.13	0.00	0.00		0	42	21	22	22	22	22	22	22	22	22	22	22	22	22	33	33	40	40	40	38	38	36	36	36
7. *S. ambiguum* SKS949	0.25	0.25	0.13	0.00	0.00	0.00		42	21	22	22	22	22	22	22	22	22	22	22	22	22	33	33	40	40	40	38	38	36	36	36
8. *S. caudatum* SKS302	2.79	2.79	2.79	2.66	2.66	2.66	2.66		36	36	36	36	36	36	36	36	36	36	36	36	36	52	52	24	28	28	26	26	28	28	28
9. *S. minus* SKS731	1.33	1.33	1.20	1.33	1.33	1.33	1.33	2.28		1	1	1	1	1	1	1	1	1	1	1	1	24	24	32	26	26	22	22	23	23	23
10. *S. minus* SKS794	1.40	1.40	1.27	1.40	1.40	1.40	1.40	2.28	0.06		0	0	0	0	0	0	0	0	0	0	0	25	25	31	25	25	21	21	22	22	22
11. *S. minus* SKS939	1.40	1.40	1.27	1.40	1.40	1.40	1.40	2.28	0.06	0.00		0	0	0	0	0	0	0	0	0	0	25	25	31	25	25	21	21	22	22	22
12. *S. minus* SKS1044	1.40	1.40	1.27	1.40	1.40	1.40	1.40	2.28	0.06	0.00	0.00		0	0	0	0	0	0	0	0	0	25	25	31	25	25	21	21	22	22	22
13. *S. minus* GuS1058	1.40	1.40	1.27	1.40	1.40	1.40	1.40	2.28	0.06	0.00	0.00	0.00		0	0	0	0	0	0	0	0	25	25	31	25	25	21	21	22	22	22
14. *S. minus* SKS840	1.40	1.40	1.27	1.40	1.40	1.40	1.40	2.28	0.06	0.00	0.00	0.00	0.00		0	0	0	0	0	0	0	25	25	31	25	25	21	21	22	22	22
15. *S. minus* SKS161	1.40	1.40	1.27	1.40	1.40	1.40	1.40	2.28	0.06	0.00	0.00	0.00	0.00	0.00		0	0	0	0	0	0	25	25	31	25	25	21	21	22	22	22
16. *S. minus* SKS182	1.40	1.40	1.27	1.40	1.40	1.40	1.40	2.28	0.06	0.00	0.00	0.00	0.00	0.00	0.00		0	0	0	0	0	25	25	31	25	25	21	21	22	22	22
17. *S. minus* SKS743	1.40	1.40	1.27	1.40	1.40	1.40	1.40	2.28	0.06	0.00	0.00	0.00	0.00	0.00	0.00	0.00		0	0	0	0	25	25	31	25	25	21	21	22	22	22
18. *S. minus* SKS214	1.40	1.40	1.27	1.40	1.40	1.40	1.40	2.28	0.06	0.00	0.00	0.00	0.00	0.00	0.00	0.00	0.00		0	0	0	25	25	31	25	25	21	21	22	22	22
19. *S. minus* SKS386	1.40	1.40	1.27	1.40	1.40	1.40	1.40	2.28	0.06	0.00	0.00	0.00	0.00	0.00	0.00	0.00	0.00	0.00		0	0	25	25	31	25	25	21	21	22	22	22
20. *S. minus* SKS1018	1.40	1.40	1.27	1.40	1.40	1.40	1.40	2.28	0.06	0.00	0.00	0.00	0.00	0.00	0.00	0.00	0.00	0.00	0.00		0	25	25	31	25	25	21	21	22	22	22
21. *S. minus* SKS1073	1.40	1.40	1.27	1.40	1.40	1.40	1.40	2.28	0.06	0.00	0.00	0.00	0.00	0.00	0.00	0.00	0.00	0.00	0.00	0.00		25	25	31	25	25	21	21	22	22	22
22. *S. subtilis* SKS937	2.09	2.09	1.97	2.09	2.09	2.09	2.09	3.30	1.52	1.59	1.59	1.59	1.59	1.59	1.59	1.59	1.59	1.59	1.59	1.59	1.59		0	45	38	38	38	38	35	35	35
23. *S. subtilis* SKS1056	2.09	2.09	1.97	2.09	2.09	2.09	2.09	3.30	1.52	1.59	1.59	1.59	1.59	1.59	1.59	1.59	1.59	1.59	1.59	1.59	1.59	0.00		45	38	38	38	38	35	35	35
24. *S. teres* SKS1045	2.54	2.54	2.54	2.54	2.54	2.54	2.54	1.52	2.03	1.97	1.97	1.97	1.97	1.97	1.97	1.97	1.97	1.97	1.97	1.97	1.97	2.85	2.85		11	11	12	12	21	21	21
25. *S. teres* SKS026	2.54	2.54	2.41	2.54	2.54	2.54	2.54	1.78	1.65	1.59	1.59	1.59	1.59	1.59	1.59	1.59	1.59	1.59	1.59	1.59	1.59	2.41	2.41	0.70		0	4	4	13	13	13
26. *S. teres* SKS351	2.54	2.54	2.41	2.54	2.54	2.54	2.54	1.78	1.65	1.59	1.59	1.59	1.59	1.59	1.59	1.59	1.59	1.59	1.59	1.59	1.59	2.41	2.41	0.70	0.00		4	4	13	13	13
27. *S. teres* SKS187	2.41	2.41	2.28	2.41	2.41	2.41	2.41	1.65	1.40	1.33	1.33	1.33	1.33	1.33	1.33	1.33	1.33	1.33	1.33	1.33	1.33	2.41	2.41	0.76	0.25	0.25		0	11	11	11
28. *S. teres* SKS085	2.41	2.41	2.28	2.41	2.41	2.41	2.41	1.65	1.40	1.33	1.33	1.33	1.33	1.33	1.33	1.33	1.33	1.33	1.33	1.33	1.33	2.41	2.41	0.76	0.25	0.25	0.00		11	11	11
29. *S. yagiui* SKS977	2.28	2.28	2.16	2.28	2.28	2.28	2.28	1.78	1.46	1.40	1.40	1.40	1.40	1.40	1.40	1.40	1.40	1.40	1.40	1.40	1.40	2.22	2.22	1.33	0.82	0.82	0.70	0.70		0	0
30. *S. yagiui* SKS164	2.28	2.28	2.16	2.28	2.28	2.28	2.28	1.78	1.46	1.40	1.40	1.40	1.40	1.40	1.40	1.40	1.40	1.40	1.40	1.40	1.40	2.22	2.22	1.33	0.82	0.82	0.70	0.70	0.00		0
31. *S. yagiui* SKS071	2.28	2.28	2.16	2.28	2.28	2.28	2.28	1.78	1.46	1.40	1.40	1.40	1.40	1.40	1.40	1.40	1.40	1.40	1.40	1.40	1.40	2.22	2.22	1.33	0.82	0.82	0.70	0.70	0.00	0.00	

Supplementary Table S14. Numbers of nucleotide differences (above diagonal) and pairwise distances (%, below diagonal) of the ITS regions among 31 *Spirostomum* populations.

Taxon	1	2	3	4	5	6	7	8	9	10	11	12	13	14	15	16	17	18	19	20	21	22	23	24	25	26	27	28	29	30	31
1. *S. ambiguum* SKS167		0	0	0	0	0	0	25	11	11	11	11	11	11	12	9	11	10	10	11	11	3	3	19	21	21	23	19	29	29	29
2. *S. ambiguum* SKS185	0.00		0	0	0	0	0	25	11	11	11	11	11	11	12	9	11	10	10	11	11	3	3	19	21	21	23	19	29	29	29
3. *S. ambiguum* SKS254	0.00	0.00		0	0	0	0	25	11	11	11	11	11	11	12	9	11	10	10	11	11	3	3	19	21	21	23	19	29	29	29
4. *S. ambiguum* SKS363	0.00	0.00	0.00		0	0	0	25	11	11	11	11	11	11	12	9	11	10	10	11	11	3	3	19	21	21	23	19	29	29	29
5. *S. ambiguum* SKS779	0.00	0.00	0.00	0.00		0	0	25	11	11	11	11	11	11	12	9	11	10	10	11	11	3	3	19	21	21	23	19	29	29	29
6. *S. ambiguum* SKS949	0.00	0.00	0.00	0.00	0.00		0	25	11	11	11	11	11	11	12	9	11	10	10	11	11	3	3	19	21	21	23	19	29	29	29
7. *S. ambiguum* SKS989	0.00	0.00	0.00	0.00	0.00	0.00		25	11	11	11	11	11	11	12	9	11	10	10	11	11	3	3	19	21	21	23	19	29	29	29
8. *S. caudatum* SKS302	10.68	10.68	10.68	10.68	10.68	10.68	10.68		31	31	31	31	31	31	30	29	31	30	30	31	31	25	25	19	20	20	19	19	24	24	24
9. *S. minus* SKS161	4.70	4.70	4.70	4.70	4.70	4.70	4.70	13.25		0	0	0	0	0	1	2	0	1	1	0	0	12	12	25	27	27	27	25	32	32	32
10. *S. minus* SKS214	4.70	4.70	4.70	4.70	4.70	4.70	4.70	13.25	0.00		0	0	0	0	1	2	0	1	1	0	0	12	12	25	27	27	27	25	32	32	32
11. *S. minus* SKS386	4.70	4.70	4.70	4.70	4.70	4.70	4.70	13.25	0.00	0.00		0	0	0	1	2	0	1	1	0	0	12	12	25	27	27	27	25	32	32	32
12. *S. minus* SKS182	4.70	4.70	4.70	4.70	4.70	4.70	4.70	13.25	0.00	0.00	0.00		0	0	1	2	0	1	1	0	0	12	12	25	27	27	27	25	32	32	32
13. *S. minus* SKS840	4.70	4.70	4.70	4.70	4.70	4.70	4.70	13.25	0.00	0.00	0.00	0.00		0	1	2	0	1	1	0	0	12	12	25	27	27	27	25	32	32	32
14. *S. minus* SKS1018	4.70	4.70	4.70	4.70	4.70	4.70	4.70	13.25	0.00	0.00	0.00	0.00	0.00		1	2	0	1	1	0	0	12	12	25	27	27	27	25	32	32	32
15. *S. minus* SKS731	5.13	5.13	5.13	5.13	5.13	5.13	5.13	12.82	0.43	0.43	0.43	0.43	0.43	0.43		3	1	2	2	1	1	13	13	25	27	27	26	25	31	31	31
16. *S. minus* GuS1058	3.85	3.85	3.85	3.85	3.85	3.85	3.85	12.39	0.85	0.85	0.85	0.85	0.85	0.85	1.28		2	1	1	2	2	10	10	23	25	25	26	23	30	30	30
17. *S. minus* SKS1044	4.70	4.70	4.70	4.70	4.70	4.70	4.70	13.25	0.00	0.00	0.00	0.00	0.00	0.00	0.43	0.85		1	1	0	0	12	12	25	27	27	27	25	32	32	32
18. *S. minus* SKS794	4.27	4.27	4.27	4.27	4.27	4.27	4.27	12.82	0.43	0.43	0.43	0.43	0.43	0.43	0.85	0.43	0.43		0	1	1	11	11	24	26	26	26	24	31	31	31
19. *S. minus* SKS939	4.27	4.27	4.27	4.27	4.27	4.27	4.27	12.82	0.43	0.43	0.43	0.43	0.43	0.43	0.85	0.43	0.43	0.00		1	1	11	11	24	26	26	26	24	31	31	31
20. *S. minus* SKS1073	4.70	4.70	4.70	4.70	4.70	4.70	4.70	13.25	0.00	0.00	0.00	0.00	0.00	0.00	0.43	0.85	0.00	0.43	0.43		0	12	12	25	27	27	27	25	32	32	32
21. *S. minus* SKS743	4.70	4.70	4.70	4.70	4.70	4.70	4.70	13.25	0.00	0.00	0.00	0.00	0.00	0.00	0.43	0.85	0.00	0.43	0.43	0.00		12	12	25	27	27	27	25	32	32	32
22. *S. subtilis* SKS937	1.28	1.28	1.28	1.28	1.28	1.28	1.28	10.68	5.13	5.13	5.13	5.13	5.13	5.13	5.56	4.27	5.13	4.70	4.70	5.13	5.13		0	19	21	21	23	19	29	29	29
23. *S. subtilis* SKS1056	1.28	1.28	1.28	1.28	1.28	1.28	1.28	10.68	5.13	5.13	5.13	5.13	5.13	5.13	5.56	4.27	5.13	4.70	4.70	5.13	5.13	0.00		19	21	21	23	19	29	29	29
24. *S. teres* SKS026	8.12	8.12	8.12	8.12	8.12	8.12	8.12	8.12	10.68	10.68	10.68	10.68	10.68	10.68	10.68	9.83	10.68	10.26	10.26	10.68	10.68	8.12	8.12		3	3	14	0	14	14	14
25. *S. teres* SKS187	8.97	8.97	8.97	8.97	8.97	8.97	8.97	8.55	11.54	11.54	11.54	11.54	11.54	11.54	11.54	10.68	11.54	11.11	11.11	11.54	11.54	8.97	8.97	1.28		0	13	3	15	15	15
26. *S. teres* SKS085	8.97	8.97	8.97	8.97	8.97	8.97	8.97	8.55	11.54	11.54	11.54	11.54	11.54	11.54	11.54	10.68	11.54	11.11	11.11	11.54	11.54	8.97	8.97	1.28	0.00		13	3	15	15	15
27. *S. teres* SKS1045	9.83	9.83	9.83	9.83	9.83	9.83	9.83	8.12	11.54	11.54	11.54	11.54	11.54	11.54	11.11	11.11	11.54	11.11	11.11	11.54	11.54	9.83	9.83	5.98	5.56	5.56		14	15	15	15
28. *S. teres* SKS351	8.12	8.12	8.12	8.12	8.12	8.12	8.12	8.12	10.68	10.68	10.68	10.68	10.68	10.68	10.68	9.83	10.68	10.26	10.26	10.68	10.68	8.12	8.12	0.00	1.28	1.28	5.98		14	14	14
29. *S. yagiui* SKS071	12.39	12.39	12.39	12.39	12.39	12.39	12.39	10.26	13.68	13.68	13.68	13.68	13.68	13.68	13.25	12.82	13.68	13.25	13.25	13.68	13.68	12.39	12.39	5.98	6.41	6.41	6.41	5.98		0	0
30. *S. yagiui* SKS164	12.39	12.39	12.39	12.39	12.39	12.39	12.39	10.26	13.68	13.68	13.68	13.68	13.68	13.68	13.25	12.82	13.68	13.25	13.25	13.68	13.68	12.39	12.39	5.98	6.41	6.41	6.41	5.98	0.00		0
31. *S. yagiui* SKS977	12.39	12.39	12.39	12.39	12.39	12.39	12.39	10.26	13.68	13.68	13.68	13.68	13.68	13.68	13.25	12.82	13.68	13.25	13.25	13.68	13.68	12.39	12.39	5.98	6.41	6.41	6.41	5.98	0.00	0.00	

Supplementary Table S15. Numbers of nucleotide differences (above diagonal) and pairwise distances (%, below diagonal) of the D1D2 domain of the 28S rRNA gene sequences among 31 *Spirostomum* populations.

Taxon	**1**	**2**	**3**	**4**	**5**	**6**	**7**	**8**	**9**	**10**	**11**	**12**	**13**	**14**	**15**	**16**	**17**	**18**	**19**	**20**	**21**	**22**	**23**	**24**	**25**	**26**	**27**	**28**	**29**	**30**	**31**
1. *S. ambiguum* SKS185		0	0	0	1	2	2	39	18	18	18	18	18	18	18	18	18	18	18	18	18	22	22	44	43	45	45	43	39	39	39
2. *S. ambiguum* SKS363	0.00		0	0	1	2	2	39	18	18	18	18	18	18	18	18	18	18	18	18	18	22	22	44	43	45	45	43	39	39	39
3. *S. ambiguum* SKS949	0.00	0.00		0	1	2	2	39	18	18	18	18	18	18	18	18	18	18	18	18	18	22	22	44	43	45	45	43	39	39	39
4. *S. ambiguum* SKS989	0.00	0.00	0.00		1	2	2	39	18	18	18	18	18	18	18	18	18	18	18	18	18	22	22	44	43	45	45	43	39	39	39
5. *S. ambiguum* SKS779	0.21	0.21	0.21	0.21		1	1	40	17	17	17	17	17	17	17	17	17	17	17	17	17	21	21	45	44	46	46	44	40	40	40
6. *S. ambiguum* SKS167	0.42	0.42	0.42	0.42	0.21		0	39	16	16	16	16	16	16	16	16	16	16	16	16	16	20	20	44	43	45	45	43	39	39	39
7. *S. ambiguum* SKS254	0.42	0.42	0.42	0.42	0.21	0.00		39	16	16	16	16	16	16	16	16	16	16	16	16	16	20	20	44	43	45	45	43	39	39	39
8. *S. caudatum* SKS302	8.21	8.21	8.21	8.21	8.42	8.21	8.21		39	39	39	39	39	39	39	39	39	39	39	39	39	42	42	37	33	36	36	33	30	30	30
9. *S. minus* SKS939	3.79	3.79	3.79	3.79	3.58	3.37	3.37	8.21		0	0	0	0	0	0	0	0	0	0	0	0	22	22	36	34	36	36	34	36	36	36
10. *S. minus* SKS161	3.79	3.79	3.79	3.79	3.58	3.37	3.37	8.21	0.00		0	0	0	0	0	0	0	0	0	0	0	22	22	36	34	36	36	34	36	36	36
11. *S. minus* SKS214	3.79	3.79	3.79	3.79	3.58	3.37	3.37	8.21	0.00	0.00		0	0	0	0	0	0	0	0	0	0	22	22	36	34	36	36	34	36	36	36
12. *S. minus* SKS794	3.79	3.79	3.79	3.79	3.58	3.37	3.37	8.21	0.00	0.00	0.00		0	0	0	0	0	0	0	0	0	22	22	36	34	36	36	34	36	36	36
13. *S. minus* SKS840	3.79	3.79	3.79	3.79	3.58	3.37	3.37	8.21	0.00	0.00	0.00	0.00		0	0	0	0	0	0	0	0	22	22	36	34	36	36	34	36	36	36
14. *S. minus* SKS1018	3.79	3.79	3.79	3.79	3.58	3.37	3.37	8.21	0.00	0.00	0.00	0.00	0.00		0	0	0	0	0	0	0	22	22	36	34	36	36	34	36	36	36
15. *S. minus* SKS1044	3.79	3.79	3.79	3.79	3.58	3.37	3.37	8.21	0.00	0.00	0.00	0.00	0.00	0.00		0	0	0	0	0	0	22	22	36	34	36	36	34	36	36	36
16. *S. minus* SKS731	3.79	3.79	3.79	3.79	3.58	3.37	3.37	8.21	0.00	0.00	0.00	0.00	0.00	0.00	0.00		0	0	0	0	0	22	22	36	34	36	36	34	36	36	36
17. *S. minus* SKS743	3.79	3.79	3.79	3.79	3.58	3.37	3.37	8.21	0.00	0.00	0.00	0.00	0.00	0.00	0.00	0.00		0	0	0	0	22	22	36	34	36	36	34	36	36	36
18. *S. minus* GuS1058	3.79	3.79	3.79	3.79	3.58	3.37	3.37	8.21	0.00	0.00	0.00	0.00	0.00	0.00	0.00	0.00	0.00		0	0	0	22	22	36	34	36	36	34	36	36	36
19. *S. minus* SKS1073	3.79	3.79	3.79	3.79	3.58	3.37	3.37	8.21	0.00	0.00	0.00	0.00	0.00	0.00	0.00	0.00	0.00	0.00		0	0	22	22	36	34	36	36	34	36	36	36
20. *S. minus* SKS386	3.79	3.79	3.79	3.79	3.58	3.37	3.37	8.21	0.00	0.00	0.00	0.00	0.00	0.00	0.00	0.00	0.00	0.00	0.00		0	22	22	36	34	36	36	34	36	36	36
21. *S. minus* SKS182	3.79	3.79	3.79	3.79	3.58	3.37	3.37	8.21	0.00	0.00	0.00	0.00	0.00	0.00	0.00	0.00	0.00	0.00	0.00	0.00		22	22	36	34	36	36	34	36	36	36
22. *S. subtilis* SKS1056	4.63	4.63	4.63	4.63	4.42	4.21	4.21	8.84	4.63	4.63	4.63	4.63	4.63	4.63	4.63	4.63	4.63	4.63	4.63	4.63	4.63		0	44	44	46	46	44	42	42	42
23. *S. subtilis* SKS937	4.63	4.63	4.63	4.63	4.42	4.21	4.21	8.84	4.63	4.63	4.63	4.63	4.63	4.63	4.63	4.63	4.63	4.63	4.63	4.63	4.63	0.00		44	44	46	46	44	42	42	42
24. *S. teres* SKS1045	9.26	9.26	9.26	9.26	9.47	9.26	9.26	7.79	7.58	7.58	7.58	7.58	7.58	7.58	7.58	7.58	7.58	7.58	7.58	7.58	7.58	9.26	9.26		21	21	21	21	21	21	21
25. *S. teres* SKS187	9.05	9.05	9.05	9.05	9.26	9.05	9.05	6.95	7.16	7.16	7.16	7.16	7.16	7.16	7.16	7.16	7.16	7.16	7.16	7.16	7.16	9.26	9.26	4.42		5	5	0	16	16	16
26. *S. teres* SKS026	9.47	9.47	9.47	9.47	9.68	9.47	9.47	7.58	7.58	7.58	7.58	7.58	7.58	7.58	7.58	7.58	7.58	7.58	7.58	7.58	7.58	9.68	9.68	4.42	1.05		0	5	13	13	13
27. *S. teres* SKS351	9.47	9.47	9.47	9.47	9.68	9.47	9.47	7.58	7.58	7.58	7.58	7.58	7.58	7.58	7.58	7.58	7.58	7.58	7.58	7.58	7.58	9.68	9.68	4.42	1.05	0.00		5	13	13	13
28. *S. teres* SKS085	9.05	9.05	9.05	9.05	9.26	9.05	9.05	6.95	7.16	7.16	7.16	7.16	7.16	7.16	7.16	7.16	7.16	7.16	7.16	7.16	7.16	9.26	9.26	4.42	0.00	1.05	1.05		16	16	16
29. *S. yagiui* SKS071	8.21	8.21	8.21	8.21	8.42	8.21	8.21	6.32	7.58	7.58	7.58	7.58	7.58	7.58	7.58	7.58	7.58	7.58	7.58	7.58	7.58	8.84	8.84	4.42	3.37	2.74	2.74	3.37		0	0
30. *S. yagiui* SKS164	8.21	8.21	8.21	8.21	8.42	8.21	8.21	6.32	7.58	7.58	7.58	7.58	7.58	7.58	7.58	7.58	7.58	7.58	7.58	7.58	7.58	8.84	8.84	4.42	3.37	2.74	2.74	3.37	0.00		0
31. *S. yagiui* SKS977	8.21	8.21	8.21	8.21	8.42	8.21	8.21	6.32	7.58	7.58	7.58	7.58	7.58	7.58	7.58	7.58	7.58	7.58	7.58	7.58	7.58	8.84	8.84	4.42	3.37	2.74	2.74	3.37	0.00	0.00	

Supplementary Table S16. Numbers of nucleotide differences (above diagonal) and pairwise distances (%, below diagonal) of the alpha-tubulin gene sequences among 31 *Spirostomum* populations.

Taxon	1	2	3	4	5	6	7	8	9	10	11	12	13	14	15	16	17	18	19	20	21	22	23	24	25	26	27	28	29	30	31
1. *S. ambiguum* SKS989		0	0	3	0	3	0	100	99	99	99	99	99	100	100	99	100	99	99	99	99	89	88	110	119	115	119	110	101	100	101
2. *S. ambiguum* SKS363	0.00		0	3	0	3	0	100	99	99	99	99	99	100	100	99	100	99	99	99	99	89	88	110	119	115	119	110	101	100	101
3. *S. ambiguum* SKS779	0.00	0.00		3	0	3	0	100	99	99	99	99	99	100	100	99	100	99	99	99	99	89	88	110	119	115	119	110	101	100	101
4. *S. ambiguum* SKS254	0.32	0.32	0.32		3	0	3	102	99	99	99	99	99	100	100	99	100	99	99	99	99	89	88	110	119	113	119	110	99	98	99
5. *S. ambiguum* SKS. 949	0.00	0.00	0.00	0.32		3	0	100	99	99	99	99	99	100	100	99	100	99	99	99	99	89	88	110	119	115	119	110	101	100	101
6. *S. ambiguum* SKS167	0.32	0.32	0.32	0.00	0.32		3	102	99	99	99	99	99	100	100	99	100	99	99	99	99	89	88	110	119	113	119	110	99	98	99
7. *S. ambiguum* SKS185	0.00	0.00	0.00	0.32	0.00	0.32		100	99	99	99	99	99	100	100	99	100	99	99	99	99	89	88	110	119	115	119	110	101	100	101
8. *S. caudatum* SKS813	10.76	10.76	10.76	10.98	10.76	10.98	10.76		108	108	108	108	108	109	109	108	108	108	108	108	108	117	118	100	98	97	100	100	107	106	107
9. *S. minus* SKS182	10.66	10.66	10.66	10.66	10.66	10.66	10.66	11.63		0	0	0	0	1	2	0	1	0	0	0	0	67	68	101	103	109	104	101	107	106	108
10. *S. minus* SKS731	10.66	10.66	10.66	10.66	10.66	10.66	10.66	11.63	0.00		0	0	0	1	2	0	1	0	0	0	0	67	68	101	103	109	104	101	107	106	108
11. *S. minus* SKS1044	10.66	10.66	10.66	10.66	10.66	10.66	10.66	11.63	0.00	0.00		0	0	1	2	0	1	0	0	0	0	67	68	101	103	109	104	101	107	106	108
12. *S. minus* SKS161	10.66	10.66	10.66	10.66	10.66	10.66	10.66	11.63	0.00	0.00	0.00		0	1	2	0	1	0	0	0	0	67	68	101	103	109	104	101	107	106	108
13. *S. minus* SKS1018	10.66	10.66	10.66	10.66	10.66	10.66	10.66	11.63	0.00	0.00	0.00	0.00		1	2	0	1	0	0	0	0	67	68	101	103	109	104	101	107	106	108
14. *S. minus* GuS1058	10.76	10.76	10.76	10.76	10.76	10.76	10.76	11.73	0.11	0.11	0.11	0.11	0.11		3	1	2	1	1	1	1	68	69	102	104	108	105	102	108	107	109
15. *S. minus* SKS1073	10.76	10.76	10.76	10.76	10.76	10.76	10.76	11.73	0.22	0.22	0.22	0.22	0.22	0.32		2	3	2	2	2	2	67	68	101	103	111	104	101	108	107	109
16. *S. minus* SKS939	10.66	10.66	10.66	10.66	10.66	10.66	10.66	11.63	0.00	0.00	0.00	0.00	0.00	0.11	0.22		1	0	0	0	0	67	68	101	103	109	104	101	107	106	108
17. *S. minus* SKS386	10.76	10.76	10.76	10.76	10.76	10.76	10.76	11.63	0.11	0.11	0.11	0.11	0.11	0.22	0.32	0.11		1	1	1	1	68	69	101	103	109	104	101	108	107	109
18. *S. minus* SKS794	10.66	10.66	10.66	10.66	10.66	10.66	10.66	11.63	0.00	0.00	0.00	0.00	0.00	0.11	0.22	0.00	0.11		0	0	0	67	68	101	103	109	104	101	107	106	108
19. *S. minus* SKS840	10.66	10.66	10.66	10.66	10.66	10.66	10.66	11.63	0.00	0.00	0.00	0.00	0.00	0.11	0.22	0.00	0.11	0.00		0	0	67	68	101	103	109	104	101	107	106	108
20. *S. minus* SKS743	10.66	10.66	10.66	10.66	10.66	10.66	10.66	11.63	0.00	0.00	0.00	0.00	0.00	0.11	0.22	0.00	0.11	0.00	0.00		0	67	68	101	103	109	104	101	107	106	108
21. *S. minus* SKS214	10.66	10.66	10.66	10.66	10.66	10.66	10.66	11.63	0.00	0.00	0.00	0.00	0.00	0.11	0.22	0.00	0.11	0.00	0.00	0.00		67	68	101	103	109	104	101	107	106	108
22. *S. subtilis* SKS937	9.58	9.58	9.58	9.58	9.58	9.58	9.58	12.59	7.21	7.21	7.21	7.21	7.21	7.32	7.21	7.21	7.32	7.21	7.21	7.21	7.21		1	106	117	114	116	106	100	99	100
23. *S. subtilis* SKS1056	9.47	9.47	9.47	9.47	9.47	9.47	9.47	12.70	7.32	7.32	7.32	7.32	7.32	7.43	7.32	7.32	7.43	7.32	7.32	7.32	7.32	0.11		107	118	115	117	107	99	98	99
24. *S. teres* SKS351	11.84	11.84	11.84	11.84	11.84	11.84	11.84	10.76	10.87	10.87	10.87	10.87	10.87	10.98	10.87	10.87	10.87	10.87	10.87	10.87	10.87	11.41	11.52		79	92	79	0	101	100	103
25. *S. teres* SKS187	12.81	12.81	12.81	12.81	12.81	12.81	12.81	10.55	11.09	11.09	11.09	11.09	11.09	11.19	11.09	11.09	11.09	11.09	11.09	11.09	11.09	12.59	12.70	8.50		93	3	79	95	96	95
26. *S. teres* SKS1045	12.38	12.38	12.38	12.16	12.38	12.16	12.38	10.44	11.73	11.73	11.73	11.73	11.73	11.63	11.95	11.73	11.73	11.73	11.73	11.73	11.73	12.27	12.38	9.90	10.01		92	92	91	93	93
27. *S. teres* SKS085	12.81	12.81	12.81	12.81	12.81	12.81	12.81	10.76	11.19	11.19	11.19	11.19	11.19	11.30	11.19	11.19	11.19	11.19	11.19	11.19	11.19	12.49	12.59	8.50	0.32	9.90		79	94	95	93
28. *S. teres* SKS026	11.84	11.84	11.84	11.84	11.84	11.84	11.84	10.76	10.87	10.87	10.87	10.87	10.87	10.98	10.87	10.87	10.87	10.87	10.87	10.87	10.87	11.41	11.52	0.00	8.50	9.90	8.50		101	100	103
29. *S. yagiui* SKS071	10.87	10.87	10.87	10.66	10.87	10.66	10.87	11.52	11.52	11.52	11.52	11.52	11.52	11.63	11.63	11.52	11.63	11.52	11.52	11.52	11.52	10.76	10.66	10.87	10.23	9.80	10.12	10.87		3	3
30. *S. yagiui* SKS164	10.76	10.76	10.76	10.55	10.76	10.55	10.76	11.41	11.41	11.41	11.41	11.41	11.41	11.52	11.52	11.41	11.52	11.41	11.41	11.41	11.41	10.66	10.55	10.76	10.33	10.01	10.23	10.76	0.32		4
31. *S. yagiui* SKS977	10.87	10.87	10.87	10.66	10.87	10.66	10.87	11.52	11.63	11.63	11.63	11.63	11.63	11.73	11.73	11.63	11.73	11.63	11.63	11.63	11.63	10.76	10.66	11.09	10.23	10.01	10.01	11.09	0.32	0.43	

Supplementary Table S17. Numbers of nucleotide differences (above diagonal) and pairwise distances (%, below diagonal) of the mitochondrial CO1 (nt) gene sequences among 31 *Spirostomum* populations.

Taxon	1	2	3	4	5	6	7	8	9	10	11	12	13	14	15	16	17	18	19	20	21	22	23	24	25	26	27	28	29	30	31
1. *S. ambiguum* SKS363		0	124	124	112	8	46	155	176	175	175	174	174	175	175	173	174	174	175	174	174	139	139	144	134	135	158	144	145	144	144
2. *S. ambiguum* SKS949	0.00		124	124	112	8	46	155	176	175	175	174	174	175	175	173	174	174	175	174	174	139	139	144	134	135	158	144	145	144	144
3. *S. ambiguum* SKS167	19.31	19.31		1	82	121	117	157	181	183	183	182	182	183	183	182	182	182	183	182	182	158	156	152	139	139	161	152	151	150	146
4. *S. ambiguum* SKS254	19.31	19.31	0.16		82	121	117	157	181	183	183	182	182	183	183	182	182	182	183	182	182	158	156	153	139	139	161	153	151	150	146
5. *S. ambiguum* SKS779	17.45	17.45	12.77	12.77		111	107	147	171	168	168	167	167	168	168	167	167	167	168	167	167	142	141	146	136	137	148	146	147	144	146
6. *S. ambiguum* SKS185	1.25	1.25	18.85	18.85	17.29		43	155	178	177	177	176	176	177	177	175	176	176	177	176	176	139	139	147	136	136	160	147	143	144	142
7. *S. ambiguum* SKS989	7.17	7.17	18.22	18.22	16.67	6.70		152	176	175	176	175	174	176	175	174	175	175	176	175	175	140	139	153	135	135	160	153	148	148	145
8. *S. caudatum* SKS302	24.14	24.14	24.45	24.45	22.90	24.14	23.68		148	150	151	150	149	151	150	149	150	150	151	150	150	145	141	120	106	107	138	120	126	125	122
9. *S. minus* GuS1058	27.41	27.41	28.19	28.19	26.64	27.73	27.41	23.05		14	15	14	13	15	14	13	14	14	15	14	14	168	164	152	145	146	160	152	161	161	161
10. *S. minus* SKS161	27.26	27.26	28.50	28.50	26.17	27.57	27.26	23.36	2.18		1	2	1	1	0	3	2	2	1	2	2	165	161	145	139	140	156	145	157	155	155
11. *S. minus* SKS182	27.26	27.26	28.50	28.50	26.17	27.57	27.41	23.52	2.34	0.16		1	2	0	1	2	1	1	0	1	1	165	161	144	139	140	156	144	157	155	156
12. *S. minus* SKS214	27.10	27.10	28.35	28.35	26.01	27.41	27.26	23.36	2.18	0.31	0.16		1	1	2	1	0	0	1	0	0	164	160	143	138	139	155	143	156	154	155
13. *S. minus* SKS386	27.10	27.10	28.35	28.35	26.01	27.41	27.10	23.21	2.02	0.16	0.31	0.16		2	1	2	1	1	2	1	1	164	160	144	138	139	155	144	156	154	155
14. *S. minus* SKS731	27.26	27.26	28.50	28.50	26.17	27.57	27.41	23.52	2.34	0.16	0.00	0.16	0.31		1	2	1	1	0	1	1	165	161	144	139	140	156	144	157	155	156
15. *S. minus* SKS743	27.26	27.26	28.50	28.50	26.17	27.57	27.26	23.36	2.18	0.00	0.16	0.31	0.16	0.16		3	2	2	1	2	2	165	161	145	139	140	156	145	157	155	156
16. *S. minus* SKS794	26.95	26.95	28.35	28.35	26.01	27.26	27.10	23.21	2.02	0.47	0.31	0.16	0.31	0.31	0.47		1	1	2	1	1	163	159	143	137	138	156	143	155	153	154
17. *S. minus* SKS840	27.10	27.10	28.35	28.35	26.01	27.41	27.26	23.36	2.18	0.31	0.16	0.00	0.16	0.16	0.31	0.16		0	1	0	0	164	160	143	138	139	155	143	156	154	155
18. *S. minus* SKS1018	27.10	27.10	28.35	28.35	26.01	27.41	27.26	23.36	2.18	0.31	0.16	0.00	0.16	0.16	0.31	0.16	0.00		1	0	0	164	160	143	138	139	155	143	156	154	155
19. *S. minus* SKS1044	27.26	27.26	28.50	28.50	26.17	27.57	27.41	23.52	2.34	0.16	0.00	0.16	0.31	0.00	0.16	0.31	0.16	0.16		1	1	165	161	144	139	140	156	144	157	155	156
20. *S. minus* SKS1073	27.10	27.10	28.35	28.35	26.01	27.41	27.26	23.36	2.18	0.31	0.16	0.00	0.16	0.16	0.31	0.16	0.00	0.00	0.16		0	164	160	143	138	139	155	143	156	154	155
21. *S. minus* SKS939	27.10	27.10	28.35	28.35	26.01	27.41	27.26	23.36	2.18	0.31	0.16	0.00	0.16	0.16	0.31	0.16	0.00	0.00	0.16	0.00		164	160	143	138	139	155	143	156	154	155
22. *S. subtilis* SKS1056	21.65	21.65	24.61	24.61	22.12	21.65	21.81	22.59	26.17	25.70	25.70	25.55	25.55	25.70	25.70	25.39	25.55	25.55	25.70	25.55	25.55		9	143	134	133	153	143	151	149	147
23. *S. subtilis* SKS937	21.65	21.65	24.30	24.30	21.96	21.65	21.65	21.96	25.55	25.08	25.08	24.92	24.92	25.08	25.08	24.77	24.92	24.92	25.08	24.92	24.92	1.40		140	134	133	152	140	149	147	144
24. *S. teres* SKS026	22.43	22.43	23.68	23.83	22.74	22.90	23.83	18.69	23.68	22.59	22.43	22.27	22.43	22.43	22.59	22.27	22.27	22.27	22.43	22.27	22.27	22.27	21.81		85	86	126	0	127	127	119
25. *S. teres* SKS187	20.87	20.87	21.65	21.65	21.18	21.18	21.03	16.51	22.59	21.65	21.65	21.50	21.50	21.65	21.65	21.34	21.50	21.50	21.65	21.50	21.50	20.87	20.87	13.24		1	100	85	113	111	119
26. *S. teres* SKS85	21.03	21.03	21.65	21.65	21.34	21.18	21.03	16.67	22.74	21.81	21.81	21.65	21.65	21.81	21.81	21.50	21.65	21.65	21.81	21.65	21.65	20.72	20.72	13.40	0.16		101	86	113	111	119
27. *S. teres* SKS1045	24.61	24.61	25.08	25.08	23.05	24.92	24.92	21.50	24.92	24.30	24.30	24.14	24.14	24.30	24.30	24.30	24.14	24.14	24.30	24.14	24.14	23.83	23.68	19.63	15.58	15.73		126	136	136	131
28. *S. teres* SKS351	22.43	22.43	23.68	23.83	22.74	22.90	23.83	18.69	23.68	22.59	22.43	22.27	22.43	22.43	22.59	22.27	22.27	22.27	22.43	22.27	22.27	22.27	21.81	0.00	13.24	13.40	19.63		127	127	119
29. *S. yagiui* SKS071	22.59	22.59	23.52	23.52	22.90	22.27	23.05	19.63	25.08	24.45	24.45	24.30	24.30	24.45	24.45	24.14	24.30	24.30	24.45	24.30	24.30	23.52	23.21	19.78	17.60	17.60	21.18	19.78		7	39
30. *S. yagiui* SKS977	22.43	22.43	23.36	23.36	22.43	22.43	23.05	19.47	25.08	24.14	24.14	23.99	23.99	24.14	24.14	23.83	23.99	23.99	24.14	23.99	23.99	23.21	22.90	19.78	17.29	17.29	21.18	19.78	1.09		38
31. *S. yagiui* SKS164	22.43	22.43	22.74	22.74	22.74	22.12	22.59	19.00	25.08	24.30	24.30	24.14	24.14	24.30	24.30	23.99	24.14	24.14	24.30	24.14	24.14	22.90	22.43	18.54	18.54	18.54	20.40	18.54	6.07	5.92	

Supplementary Table S18. Numbers of amino acid differences (above diagonal) and pairwise distances (%, below diagonal) of the mitochondrial CO1 gene sequences among 31 *Spirostomum* populations.

Taxon	1	2	3	4	5	6	7	8	9	10	11	12	13	14	15	16	17	18	19	20	21	22	23	24	25	26	27	28	29	30	31
1. *S. ambiguum* SKS363		0	30	30	30	1	5	45	50	47	47	47	47	47	47	47	47	47	47	47	47	37	38	45	40	40	44	45	42	42	42
2. *S. ambiguum* SKS949	0.00		30	30	30	1	5	45	50	47	47	47	47	47	47	47	47	47	47	47	47	37	38	45	40	40	44	45	42	42	42
3. *S. ambiguum* SKS167	14.08	14.08		0	17	29	28	55	56	55	55	55	55	55	55	55	55	55	55	55	55	44	44	48	44	44	48	48	46	46	46
4. *S. ambiguum* SKS254	14.08	14.08	0.00		17	29	28	55	56	55	55	55	55	55	55	55	55	55	55	55	55	44	44	48	44	44	48	48	46	46	46
5. *S. ambiguum* SKS779	14.08	14.08	7.98	7.98		29	27	53	52	51	51	51	51	51	51	51	51	51	51	51	51	42	43	47	42	42	46	47	44	44	45
6. *S. ambiguum* SKS185	0.47	0.47	13.62	13.62	13.62		4	44	51	48	48	48	48	48	48	48	48	48	48	48	48	36	37	45	40	40	44	45	41	41	41
7. *S. ambiguum* SKS989	2.35	2.35	13.15	13.15	12.68	1.88		45	51	48	48	48	48	48	48	48	48	48	48	48	48	35	36	44	37	37	42	44	42	42	42
8. *S. caudatum* SKS302	21.13	21.13	25.82	25.82	24.88	20.66	21.13		46	46	46	46	46	46	46	46	46	46	46	46	46	44	44	34	30	30	38	34	31	31	32
9. *S. minus* GuS1058	23.47	23.47	26.29	26.29	24.41	23.94	23.94	21.60		3	3	3	3	3	3	3	3	3	3	3	3	56	57	51	47	47	40	51	46	46	47
10. *S. minus* SKS161	22.07	22.07	25.82	25.82	23.94	22.54	22.54	21.60	1.41		0	0	0	0	0	0	0	0	0	0	0	55	56	48	44	44	39	48	43	43	44
11. *S. minus* SKS182	22.07	22.07	25.82	25.82	23.94	22.54	22.54	21.60	1.41	0.00		0	0	0	0	0	0	0	0	0	0	55	56	48	44	44	39	48	43	43	44
12. *S. minus* SKS214	22.07	22.07	25.82	25.82	23.94	22.54	22.54	21.60	1.41	0.00	0.00		0	0	0	0	0	0	0	0	0	55	56	48	44	44	39	48	43	43	44
13. *S. minus* SKS386	22.07	22.07	25.82	25.82	23.94	22.54	22.54	21.60	1.41	0.00	0.00	0.00		0	0	0	0	0	0	0	0	55	56	48	44	44	39	48	43	43	44
14. *S. minus* SKS731	22.07	22.07	25.82	25.82	23.94	22.54	22.54	21.60	1.41	0.00	0.00	0.00	0.00		0	0	0	0	0	0	0	55	56	48	44	44	39	48	43	43	44
15. *S. minus* SKS743	22.07	22.07	25.82	25.82	23.94	22.54	22.54	21.60	1.41	0.00	0.00	0.00	0.00	0.00		0	0	0	0	0	0	55	56	48	44	44	39	48	43	43	44
16. *S. minus* SKS794	22.07	22.07	25.82	25.82	23.94	22.54	22.54	21.60	1.41	0.00	0.00	0.00	0.00	0.00	0.00		0	0	0	0	0	55	56	48	44	44	39	48	43	43	44
17. *S. minus* SKS840	22.07	22.07	25.82	25.82	23.94	22.54	22.54	21.60	1.41	0.00	0.00	0.00	0.00	0.00	0.00	0.00		0	0	0	0	55	56	48	44	44	39	48	43	43	44
18. *S. minus* SKS1018	22.07	22.07	25.82	25.82	23.94	22.54	22.54	21.60	1.41	0.00	0.00	0.00	0.00	0.00	0.00	0.00	0.00		0	0	0	55	56	48	44	44	39	48	43	43	44
19. *S. minus* SKS1044	22.07	22.07	25.82	25.82	23.94	22.54	22.54	21.60	1.41	0.00	0.00	0.00	0.00	0.00	0.00	0.00	0.00	0.00		0	0	55	56	48	44	44	39	48	43	43	44
20. *S. minus* SKS1073	22.07	22.07	25.82	25.82	23.94	22.54	22.54	21.60	1.41	0.00	0.00	0.00	0.00	0.00	0.00	0.00	0.00	0.00	0.00		0	55	56	48	44	44	39	48	43	43	44
21. *S. minus* SKS939	22.07	22.07	25.82	25.82	23.94	22.54	22.54	21.60	1.41	0.00	0.00	0.00	0.00	0.00	0.00	0.00	0.00	0.00	0.00	0.00		55	56	48	44	44	39	48	43	43	44
22. *S. subtilis* SKS1056	17.37	17.37	20.66	20.66	19.72	16.90	16.43	20.66	26.29	25.82	25.82	25.82	25.82	25.82	25.82	25.82	25.82	25.82	25.82	25.82	25.82		1	46	40	40	39	46	47	47	47
23. *S. subtilis* SKS937	17.84	17.84	20.66	20.66	20.19	17.37	16.90	20.66	26.76	26.29	26.29	26.29	26.29	26.29	26.29	26.29	26.29	26.29	26.29	26.29	26.29	0.47		47	41	41	40	47	47	47	47
24. *S. teres* SKS026	21.13	21.13	22.54	22.54	22.07	21.13	20.66	15.96	23.94	22.54	22.54	22.54	22.54	22.54	22.54	22.54	22.54	22.54	22.54	22.54	22.54	21.60	22.07		24	24	35	0	30	30	31
25. *S. teres* SKS187	18.78	18.78	20.66	20.66	19.72	18.78	17.37	14.08	22.07	20.66	20.66	20.66	20.66	20.66	20.66	20.66	20.66	20.66	20.66	20.66	20.66	18.78	19.25	11.27		0	23	24	26	26	27
26. *S. teres* SKS85	18.78	18.78	20.66	20.66	19.72	18.78	17.37	14.08	22.07	20.66	20.66	20.66	20.66	20.66	20.66	20.66	20.66	20.66	20.66	20.66	20.66	18.78	19.25	11.27	0.00		23	24	26	26	27
27. *S. teres* SKS1045	20.66	20.66	22.54	22.54	21.60	20.66	19.72	17.84	18.78	18.31	18.31	18.31	18.31	18.31	18.31	18.31	18.31	18.31	18.31	18.31	18.31	18.31	18.78	16.43	10.80	10.80		35	32	32	33
28. *S. teres* SKS351	21.13	21.13	22.54	22.54	22.07	21.13	20.66	15.96	23.94	22.54	22.54	22.54	22.54	22.54	22.54	22.54	22.54	22.54	22.54	22.54	22.54	21.60	22.07	0.00	11.27	11.27	16.43		30	30	31
29. *S. yagiui* SKS071	19.72	19.72	21.60	21.60	20.66	19.25	19.72	14.55	21.60	20.19	20.19	20.19	20.19	20.19	20.19	20.19	20.19	20.19	20.19	20.19	20.19	22.07	22.07	14.08	12.21	12.21	15.02	0.1408		0	2
30. *S. yagiui* SKS977	19.72	19.72	21.60	21.60	20.66	19.25	19.72	14.55	21.60	20.19	20.19	20.19	20.19	20.19	20.19	20.19	20.19	20.19	20.19	20.19	20.19	22.07	22.07	14.08	12.21	12.21	15.02	14.08	0.00		2
31. *S. yagiui* SKS164	19.72	19.72	21.60	21.60	21.13	19.25	19.72	15.02	22.07	20.66	20.66	20.66	20.66	20.66	20.66	20.66	20.66	20.66	20.66	20.66	20.66	22.07	22.07	14.55	12.68	12.68	15.49	14.55	0.94	0.94	

Supplementary Table S19. Numbers of nucleotide differences (above diagonal) and pairwise distances (%, below diagonal) of the mitochondrial CO1 (nt) gene sequences (without insert) among 31 *Spirostomum* populations.

Taxon	1	2	3	4	5	6	7	8	9	10	11	12	13	14	15	16	17	18	19	20	21	22	23	24	25	26	27	28	29	30	31
1. *S. ambiguum* SKS363		0	51	51	43	4	21	65	63	63	63	63	63	63	63	63	63	63	63	63	63	59	58	58	59	60	67	58	64	61	58
2. *S. ambiguum* SKS949	0.00		51	51	43	4	21	65	63	63	63	63	63	63	63	63	63	63	63	63	63	59	58	58	59	60	67	58	64	61	58
3. *S. ambiguum* SKS167	14.29	14.29		0	36	50	48	58	66	67	67	67	67	67	67	67	67	67	67	67	67	67	65	59	53	53	61	59	59	57	52
4. *S. ambiguum* SKS254	14.29	14.29	0.00		36	50	48	58	66	67	67	67	67	67	67	67	67	67	67	67	67	67	65	59	53	53	61	59	59	57	52
5. *S. ambiguum* SKS779	12.04	12.04	10.08	10.08		43	46	57	61	59	59	59	59	59	59	59	59	59	59	59	59	62	61	54	53	54	57	54	55	54	51
6. *S. ambiguum* SKS185	1.12	1.12	14.01	14.01	12.04		20	67	65	65	65	65	65	65	65	65	65	65	65	65	65	61	60	61	61	61	67	61	63	62	57
7. *S. ambiguum* SKS989	5.88	5.88	13.45	13.45	12.89	5.60		63	63	63	64	64	63	64	63	64	64	64	64	64	64	61	59	62	59	59	67	62	61	61	59
8. *S. caudatum* SKS302	18.21	18.21	16.25	16.25	15.97	18.77	17.65		53	54	55	55	54	55	54	55	55	55	55	55	55	67	64	59	54	55	64	59	61	60	54
9. *S. minus* GuS1058	17.65	17.65	18.49	18.49	17.09	18.21	17.65	14.85		6	7	7	6	7	6	7	7	7	7	7	7	64	60	58	57	58	67	58	62	61	63
10. *S. minus* SKS161	17.65	17.65	18.77	18.77	16.53	18.21	17.65	15.13	1.68		1	1	0	1	0	1	1	1	1	1	1	61	57	54	53	54	65	54	60	57	60
11. *S. minus* SKS182	17.65	17.65	18.77	18.77	16.53	18.21	17.93	15.41	1.96	0.28		0	1	0	1	0	0	0	0	0	0	61	57	53	53	54	65	53	60	57	60
12. *S. minus* SKS214	17.65	17.65	18.77	18.77	16.53	18.21	17.93	15.41	1.96	0.28	0.00		1	0	1	0	0	0	0	0	0	61	57	53	53	54	65	53	60	57	60
13. *S. minus* SKS386	17.65	17.65	18.77	18.77	16.53	18.21	17.65	15.13	1.68	0.00	0.28	0.28		1	0	1	1	1	1	1	1	61	57	54	53	54	65	54	60	57	60
14. *S. minus* SKS731	17.65	17.65	18.77	18.77	16.53	18.21	17.93	15.41	1.96	0.28	0.00	0.00	0.28		1	0	0	0	0	0	0	61	57	53	53	54	65	53	60	57	60
15. *S. minus* SKS743	17.65	17.65	18.77	18.77	16.53	18.21	17.65	15.13	1.68	0.00	0.28	0.28	0.00	0.28		1	1	1	1	1	1	61	57	54	53	54	65	54	60	57	60
16. *S. minus* SKS794	17.65	17.65	18.77	18.77	16.53	18.21	17.93	15.41	1.96	0.28	0.00	0.00	0.28	0.00	0.28		0	0	0	0	0	61	57	53	53	54	65	53	60	57	60
17. *S. minus* SKS840	17.65	17.65	18.77	18.77	16.53	18.21	17.93	15.41	1.96	0.28	0.00	0.00	0.28	0.00	0.28	0.00		0	0	0	0	61	57	53	53	54	65	53	60	57	60
18. *S. minus* SKS1018	17.65	17.65	18.77	18.77	16.53	18.21	17.93	15.41	1.96	0.28	0.00	0.00	0.28	0.00	0.28	0.00	0.00		0	0	0	61	57	53	53	54	65	53	60	57	60
19. *S. minus* SKS1044	17.65	17.65	18.77	18.77	16.53	18.21	17.93	15.41	1.96	0.28	0.00	0.00	0.28	0.00	0.28	0.00	0.00	0.00		0	0	61	57	53	53	54	65	53	60	57	60
20. *S. minus* SKS1073	17.65	17.65	18.77	18.77	16.53	18.21	17.93	15.41	1.96	0.28	0.00	0.00	0.28	0.00	0.28	0.00	0.00	0.00	0.00		0	61	57	53	53	54	65	53	60	57	60
21. *S. minus* SKS939	17.65	17.65	18.77	18.77	16.53	18.21	17.93	15.41	1.96	0.28	0.00	0.00	0.28	0.00	0.28	0.00	0.00	0.00	0.00	0.00		61	57	53	53	54	65	53	60	57	60
22. *S. subtilis* SKS1056	16.53	16.53	18.77	18.77	17.37	17.09	17.09	18.77	17.93	17.09	17.09	17.09	17.09	17.09	17.09	17.09	17.09	17.09	17.09	17.09	17.09		7	64	61	60	72	64	65	62	62
23. *S. subtilis* SKS937	16.25	16.25	18.21	18.21	17.09	16.81	16.53	17.93	16.81	15.97	15.97	15.97	15.97	15.97	15.97	15.97	15.97	15.97	15.97	15.97	15.97	1.96		61	61	60	71	61	64	61	59
24. *S. teres* SKS026	16.25	16.25	16.53	16.53	15.13	17.09	17.37	16.53	16.25	15.13	14.85	14.85	15.13	14.85	15.13	14.85	14.85	14.85	14.85	14.85	14.85	17.93	17.09		39	40	57	0	56	54	50
25. *S. teres* SKS187	16.53	16.53	14.85	14.85	14.85	17.09	16.53	15.13	15.97	14.85	14.85	14.85	14.85	14.85	14.85	14.85	14.85	14.85	14.85	14.85	14.85	17.09	17.09	10.92		1	46	39	50	47	49
26. *S. teres* SKS85	16.81	16.81	14.85	14.85	15.13	17.09	16.53	15.41	16.25	15.13	15.13	15.13	15.13	15.13	15.13	15.13	15.13	15.13	15.13	15.13	15.13	16.81	16.81	11.20	0.28		47	40	50	47	49
27. *S. teres* SKS1045	18.77	18.77	17.09	17.09	15.97	18.77	18.77	17.93	18.77	18.21	18.21	18.21	18.21	18.21	18.21	18.21	18.21	18.21	18.21	18.21	18.21	20.17	19.89	15.97	12.89	13.17		57	60	59	49
28. *S. teres* SKS351	16.25	16.25	16.53	16.53	15.13	17.09	17.37	16.53	16.25	15.13	14.85	14.85	15.13	14.85	15.13	14.85	14.85	14.85	14.85	14.85	14.85	17.93	17.09	0.00	10.92	11.20	15.97		56	54	50
29. *S. yagiui* SKS071	17.93	17.93	16.53	16.53	15.41	17.65	17.09	17.09	17.37	16.81	16.81	16.81	16.81	16.81	16.81	16.81	16.81	16.81	16.81	16.81	16.81	18.21	17.93	15.69	14.01	14.01	16.81	15.69		4	22
30. *S. yagiui* SKS977	17.09	17.09	15.97	15.97	15.13	17.37	17.09	16.81	17.09	15.97	15.97	15.97	15.97	15.97	15.97	15.97	15.97	15.97	15.97	15.97	15.97	17.37	17.09	15.13	13.17	13.17	16.53	15.13	1.12		20
31. *S. yagiui* SKS164	16.25	16.25	14.57	14.57	14.29	15.97	16.53	15.13	17.65	16.81	16.81	16.81	16.81	16.81	16.81	16.81	16.81	16.81	16.81	16.81	16.81	17.37	16.53	14.01	13.73	13.73	13.73	14.01	6.16	5.60	

Supplementary Table S20. Numbers of amino acid differences (above diagonal) and pairwise distances (%, below diagonal) of the mitochondrial CO1 gene sequences (without insert) among 31 *Spirostomum* populations.

Taxon	1	2	3	4	5	6	7	8	9	10	11	12	13	14	15	16	17	18	19	20	21	22	23	24	25	26	27	28	29	30	31
1. *S. ambiguum* SKS363		0	1	1	1	0	0	5	3	3	3	3	3	3	3	3	3	3	3	3	3	4	4	6	5	5	4	6	6	6	6
2. *S. ambiguum* SKS949	0.00		1	1	1	0	0	5	3	3	3	3	3	3	3	3	3	3	3	3	3	4	4	6	5	5	4	6	6	6	6
3. *S. ambiguum* SKS167	0.85	0.85		0	0	1	1	6	2	2	2	2	2	2	2	2	2	2	2	2	2	5	5	5	4	4	3	5	5	5	5
4. *S. ambiguum* SKS254	0.85	0.85	0.00		0	1	1	6	2	2	2	2	2	2	2	2	2	2	2	2	2	5	5	5	4	4	3	5	5	5	5
5. *S. ambiguum* SKS779	0.85	0.85	0.00	0.00		1	1	6	2	2	2	2	2	2	2	2	2	2	2	2	2	5	5	5	4	4	3	5	5	5	5
6. *S. ambiguum* SKS185	0.00	0.00	0.85	0.85	0.85		0	5	3	3	3	3	3	3	3	3	3	3	3	3	3	4	4	6	5	5	4	6	6	6	6
7. *S. ambiguum* SKS989	0.00	0.00	0.85	0.85	0.85	0.00		5	3	3	3	3	3	3	3	3	3	3	3	3	3	4	4	6	5	5	4	6	6	6	6
8. *S. caudatum* SKS302	4.24	4.24	5.08	5.08	5.08	4.24	4.24		4	4	4	4	4	4	4	4	4	4	4	4	4	7	7	5	5	5	4	5	6	6	6
9. *S. minus* GuS1058	2.54	2.54	1.69	1.69	1.69	2.54	2.54	3.39		0	0	0	0	0	0	0	0	0	0	0	0	5	5	3	3	3	2	3	4	4	4
10. *S. minus* SKS161	2.54	2.54	1.69	1.69	1.69	2.54	2.54	3.39	0.00		0	0	0	0	0	0	0	0	0	0	0	5	5	3	3	3	2	3	4	4	4
11. *S. minus* SKS182	2.54	2.54	1.69	1.69	1.69	2.54	2.54	3.39	0.00	0.00		0	0	0	0	0	0	0	0	0	0	5	5	3	3	3	2	3	4	4	4
12. *S. minus* SKS214	2.54	2.54	1.69	1.69	1.69	2.54	2.54	3.39	0.00	0.00	0.00		0	0	0	0	0	0	0	0	0	5	5	3	3	3	2	3	4	4	4
13. *S. minus* SKS386	2.54	2.54	1.69	1.69	1.69	2.54	2.54	3.39	0.00	0.00	0.00	0.00		0	0	0	0	0	0	0	0	5	5	3	3	3	2	3	4	4	4
14. *S. minus* SKS731	2.54	2.54	1.69	1.69	1.69	2.54	2.54	3.39	0.00	0.00	0.00	0.00	0.00		0	0	0	0	0	0	0	5	5	3	3	3	2	3	4	4	4
15. *S. minus* SKS743	2.54	2.54	1.69	1.69	1.69	2.54	2.54	3.39	0.00	0.00	0.00	0.00	0.00	0.00		0	0	0	0	0	0	5	5	3	3	3	2	3	4	4	4
16. *S. minus* SKS794	2.54	2.54	1.69	1.69	1.69	2.54	2.54	3.39	0.00	0.00	0.00	0.00	0.00	0.00	0.00		0	0	0	0	0	5	5	3	3	3	2	3	4	4	4
17. *S. minus* SKS840	2.54	2.54	1.69	1.69	1.69	2.54	2.54	3.39	0.00	0.00	0.00	0.00	0.00	0.00	0.00	0.00		0	0	0	0	5	5	3	3	3	2	3	4	4	4
18. *S. minus* SKS1018	2.54	2.54	1.69	1.69	1.69	2.54	2.54	3.39	0.00	0.00	0.00	0.00	0.00	0.00	0.00	0.00	0.00		0	0	0	5	5	3	3	3	2	3	4	4	4
19. *S. minus* SKS1044	2.54	2.54	1.69	1.69	1.69	2.54	2.54	3.39	0.00	0.00	0.00	0.00	0.00	0.00	0.00	0.00	0.00	0.00		0	0	5	5	3	3	3	2	3	4	4	4
20. *S. minus* SKS1073	2.54	2.54	1.69	1.69	1.69	2.54	2.54	3.39	0.00	0.00	0.00	0.00	0.00	0.00	0.00	0.00	0.00	0.00	0.00		0	5	5	3	3	3	2	3	4	4	4
21. *S. minus* SKS939	2.54	2.54	1.69	1.69	1.69	2.54	2.54	3.39	0.00	0.00	0.00	0.00	0.00	0.00	0.00	0.00	0.00	0.00	0.00	0.00		5	5	3	3	3	2	3	4	4	4
22. *S. subtilis* SKS1056	3.39	3.39	4.24	4.24	4.24	3.39	3.39	5.93	4.24	4.24	4.24	4.24	4.24	4.24	4.24	4.24	4.24	4.24	4.24	4.24	4.24		0	8	7	7	6	8	8	8	8
23. *S. subtilis* SKS937	3.39	3.39	4.24	4.24	4.24	3.39	3.39	5.93	4.24	4.24	4.24	4.24	4.24	4.24	4.24	4.24	4.24	4.24	4.24	4.24	4.24	0.00		8	7	7	6	8	8	8	8
24. *S. teres* SKS026	5.08	5.08	4.24	4.24	4.24	5.08	5.08	4.24	2.54	2.54	2.54	2.54	2.54	2.54	2.54	2.54	2.54	2.54	2.54	2.54	2.54	6.78	6.78		4	4	3	0	5	5	5
25. *S. teres* SKS187	4.24	4.24	3.39	3.39	3.39	4.24	4.24	4.24	2.54	2.54	2.54	2.54	2.54	2.54	2.54	2.54	2.54	2.54	2.54	2.54	2.54	5.93	5.93	3.39		0	1	4	3	3	3
26. *S. teres* SKS85	4.24	4.24	3.39	3.39	3.39	4.24	4.24	4.24	2.54	2.54	2.54	2.54	2.54	2.54	2.54	2.54	2.54	2.54	2.54	2.54	2.54	5.93	5.93	3.39	0.00		1	4	3	3	3
27. *S. teres* SKS1045	3.39	3.39	2.54	2.54	2.54	3.39	3.39	3.39	1.69	1.69	1.69	1.69	1.69	1.69	1.69	1.69	1.69	1.69	1.69	1.69	1.69	5.08	5.08	2.54	0.85	0.85		3	2	2	2
28. *S. teres* SKS351	5.08	5.08	4.24	4.24	4.24	5.08	5.08	4.24	2.54	2.54	2.54	2.54	2.54	2.54	2.54	2.54	2.54	2.54	2.54	2.54	2.54	6.78	6.78	0.00	3.39	3.39	2.54		5	5	5
29. *S. yagiui* SKS071	5.08	5.08	4.24	4.24	4.24	5.08	5.08	5.08	3.39	3.39	3.39	3.39	3.39	3.39	3.39	3.39	3.39	3.39	3.39	3.39	3.39	6.78	6.78	4.24	2.54	2.54	1.69	4.24		0	0
30. *S. yagiui* SKS977	5.08	5.08	4.24	4.24	4.24	5.08	5.08	5.08	3.39	3.39	3.39	3.39	3.39	3.39	3.39	3.39	3.39	3.39	3.39	3.39	3.39	6.78	6.78	4.24	2.54	2.54	1.69	4.24	0.00		0
31. *S. yagiui* SKS164	5.08	5.08	4.24	4.24	4.24	5.08	5.08	5.08	3.39	3.39	3.39	3.39	3.39	3.39	3.39	3.39	3.39	3.39	3.39	3.39	3.39	6.78	6.78	4.24	2.54	2.54	1.69	4.24	0.00	0.00	

Supplementary Table S21. Log likelihoods (ln L) and *P*-values of AU (approximately unbiased), WKH (weighted Kishino-Hasegawa), and WSH (weighted Shimodaira-Hasegawa) tests for tree comparisons considering different topological scenarios. Significant differences (*P*-value < 0.05) between the best unconstrained and constrained topologies are in bold.

**Topology**	**Dataset**	**Log likely-hood (–ln L)**	**Δ (–ln L)**	**AU**	**WKH**	**WSH**	**Conclusion**
Best maximum likelihood tree (unconstrained)	CON-89	7833.0066	–	0.780	0.622	0.964	–
	CO1-38	4521.7946	–	0.122	0.102	0.183	–
	Tub-32	3813.3828	–	0.800	0.000	0.861	–
Monophyly of *Spirostomum* species with moniliform macronucleus	CO1-38	4526.7854	4.99	0.122	0.102	0.183	Not rejected
	Tub-32	3820.0138	6.63	0.178	0.160	0.231	Not rejected
Monophyly of *Spirostomum* species with compact macronucleus	CO1-38	4526.8669	5.07	0.062	0.096	0.205	Not rejected
	Tub-32	3815.6151	2.23	0.267	0.188	0.415	Not rejected
Monophyly of *Spirostomum minus* clades	CON-89	7844.4821	11.47	**0.036**	0.052	0.125	Not rejected
Monophyly of *Spirostomum teres* clades 1, 2, 3 and 4	CON-89	7870.7522	37.74	**0.004**	**0.016**	**0.034**	**Rejected**
Monophyly of *Spirostomum teres* clades 1 and 2	CON-89	7834.7035	1.69	0.572	0.378	0.856	Not rejected
	CO1-38	4521.8258	0.03	0.565	0.471	0.691	Not rejected
	Tub-32	3814.7539	1.37	0.436	0.336	0.533	Not rejected
Monophyly of *Spirostomum teres* clades 3 and 4	CON-89	7852.1695	19.16	**0.046**	0.050	0.134	Not rejected
Monophyly of *Spirostomum teres* clades 1, 2 and 3	CON-89	7838.3122	5.30	0.245	0.228	0.549	Not rejected
Monophyly of *Spirostomum teres* clades 1, 2 and 4	CON-89	7874.4004	41.39	**2e-04**	**0.006**	**0.017**	**Rejected**

Supplementary Table S22. Morphological characterization of *Spirostomum* clades delimited in phylogenetic analyses.

Clade designation	Body size in vivo (µm)	Length:width ratio	Shape of posterior body end	Peristome, % of body length	CV, % of body length	Zoochlorellae	Morphology of MA	No. of MA nodules	No. of MI	No. of CG rows	References
S. ambiguum	717–1491 × 80–140	7.6–18.6:1	Truncated	45–78	6–18	Absent	Moniliform	7–26	3–17	2–5	Boscaro *et al.* (2014), Fernandes *et al.* (2016), Gao *et al.* (2016), Schmidt *et al.* (2007), Shazib *et al.* (2014; 2016), Present study
S. caudatum	442–450 × 20–24	17–22:1	Tail-like	18–20	47–55	Absent	Ellipsoid	1	N.A.	2–3	Shazib *et al.* (2014), Present study
S. dharwarensis	327–519 × 28–51	8.4–14.8:1	Rounded	45–68	8–28	Absent	Filiform	1	1–6	3–4	Boscaro *et al.* (2014)
S. minus clade 1	342–735 × 29–61	7.4–19.1:1	N.A.	40–62	6–26	Absent	Moniliform	5–25	1–9	2–5	Boscaro *et al.* (2014), Schmidt *et al.* (2007), Present study
S. minus clade 2	358–997 × 28–78	6.5–16.4:1	Truncated	44–65	6–25	Absent	Moniliform	7–20	1–18	2–4	Boscaro *et al.* (2014), Shazib *et al.* (2016), Present study
S. semivirescens	630–1425 × 35	N.A.	N.A.	*ca.* 50	N.A.	Present	Moniliform	12	N.A.	N.A.	Esteban *et al.* (2009), Hines *et al.* (2018)
S. subtilis	630–1190 × 32–55	12.8–27.0:1	Truncated	28–61	10–39	Absent	Moniliform	9–29	1–6	1–4	Boscaro *et al.* (2014), Present study
S. teres clade 1	219–471 × 22–55	5.4–16.4:1	Truncated	31–56	7–31	Absent	Ellipsoid	1	1–4	2–6	Boscaro *et al.* (2014), Schmidt *et al.* (2007), Shazib *et al.* (2014; 2016), Present study
S. teres clade 2	191–327 × 25–41	5.4–10.7:1	Truncated	32–52	7–24	Absent	Ellipsoid	1	1–3	2–4	Boscaro *et al.* (2014), Fernandes *et al.* (2016), Present study
S. teres clade 3	305–430 × 32–45	8.2–11.1:1	N.A.	36–51	16–38	Absent	Ellipsoid	1	1–2	2	Boscaro *et al.* (2014)
S. teres clade 4	269–679 × 27–53	7.9–19.1:1	N.A.	39–56	10–26	Absent	Ellipsoid	1	1–3	3–5	Boscaro *et al.* (2014)
S. yagiui	200–630 × 20–50	5.9–15.8:1	Truncated	21–59	5–27	Absent	Ellipsoid, curved cylindroid	1	1–5	2–6	Boscaro *et al.* (2014), Chen *et al.* (2017), Present study

Notes: CG, cortical granules; CV, contractile vacuole; MA, macronucleus; MI, micronucleus; N.A., data not available.

References

Boscaro, V. *et al.* Focusing on genera to improve species identification: Revised systematics of the ciliate *Spirostomum*. *Protist* **165**, 527–541; 10.1016/j.protis.2014.05.004 (2014).

Chen, X., Kim, J. H., Shazib, S. U. A., Kwon, C. B. & Shin, M. K. Morphology and molecular phylogeny of three heterotrichid species (Ciliophora, Heterotrichea), including a new species of *Anigsteinia*. *Eur. J. Protistol*. **61**, 278–293; 10.1016/j.ejop.2017.06.005 (2017).

Esteban, G. F., Bradley, M. W., Finlay, B. J. A case-building *Spirostomum* (Ciliophora, Heterotrichida) with zoochlorellae. *Eur. J. Protistol.* **45**, 156–158; 10.1016/j.ejop.2009.01.002 (2009).

Fernandes, N. M., Paiva, T. da S., da Silva-Neto, I. D., Schlegel, M. & Schrago, C. G. Expanded phylogenetic analyses of the class Heterotrichea (Ciliophora, Postciliodesmatophora) using five molecular markers and morphological data. *Mol. Phylogenet. Evol*. **95**, 229–246; 10.1016/j.ympev.2015.10.030 (2016).

Gao, F. *et al.* The all-data-based evolutionary hypothesis of ciliated protists with a revised classification of the phylum Ciliophora (Eukaryota, Alveolata). *Sci. Rep*. **6**, 24874; 10.1038/srep24874 (2016).

Hines, H. N., Onsbring, H., Ettema, T. J. & Esteban, G. F. Molecular investigation of the ciliate *Spirostomum semivirescens*, with first transcriptome and new geographical records. *Protist* **169**, 875–886; 10.1016/j.protis.2018.08.001 (2018).

Schmidt, S. L., Treuner, T., Schlegel, M. & Bernhard, D. Multiplex PCR approach for species detection and differentiation within the genus *Spirostomum* (Ciliophora, Heterotrichea). *Protist* **158**, 139-145; 10.1016/j.protis.2006.11.005 (2007).

Shazib, S. U. A., Vďačný, P., Kim, J. H., Jang, S. W., Shin, M. K. Phylogenetic relationships of the ciliate class Heterotrichea (Protista, Ciliophora, Postciliodesmatophora) inferred from multiple molecular markers and multifaceted analysis strategy. *Mol. Phylogenet. Evol*. **78**, 118–135; 10.1016/j.ympev.2014.05.012 (2014).

Shazib, S. U. A., Vďačný, P., Kim, J. H., Jang, S. W., Shin, M. K. Molecular phylogeny and species delimitation within the ciliate genus *Spirostomum* (Ciliophora, Postciliodesmatophora, Heterotrichea), using the internal transcribed spacer region. *Mol. Phylogenet. Evol*. **102**, 128–144; 10.1016/j.ympev.2016.05.041 (2016).
